# HAPPENN is a novel tool for hemolytic activity prediction for therapeutic peptides which employs neural networks

**DOI:** 10.1038/s41598-020-67701-3

**Published:** 2020-07-02

**Authors:** Patrick Brendan Timmons, Chandralal M. Hewage

**Affiliations:** UCD School of Biomolecular and Biomedical Science, UCD Centre for Synthesis and Chemical Biology, UCD Conway Institute, University College Dublin, Dublin 4, Ireland

**Keywords:** Peptides, Cheminformatics, Drug discovery and development, Machine learning, Protein function predictions, Virtual drug screening, Drug development

## Abstract

The growing prevalence of resistance to antibiotics motivates the search for new antibacterial agents. Antimicrobial peptides are a diverse class of well-studied membrane-active peptides which function as part of the innate host defence system, and form a promising avenue in antibiotic drug research. Some antimicrobial peptides exhibit toxicity against eukaryotic membranes, typically characterised by hemolytic activity assays, but currently, the understanding of what differentiates hemolytic and non-hemolytic peptides is limited. This study leverages advances in machine learning research to produce a novel artificial neural network classifier for the prediction of hemolytic activity from a peptide’s primary sequence. The classifier achieves best-in-class performance, with cross-validated accuracy of $85.7\%$ and Matthews correlation coefficient of 0.71. This innovative classifier is available as a web server at https://research.timmons.eu/happenn, allowing the research community to utilise it for in silico screening of peptide drug candidates for high therapeutic efficacies.

## Introduction

All living organisms exist in an environment teeming with harmful microbes, which they can become exposed to through contact, ingestion or inhalation^1^. An important part of the host defence mechanisms that protect against these pathogens are antimicrobial peptides (AMPs).

The serious issue of pathogen resistance to multiple antibiotics is the motivation for the search of novel drugs that can be used without the development of resistance. AMPs are a class of compounds that are promising as novel antibiotics, due to good selectivity and only a limited number of cases of resistance, attributed to their relatively non-specific mechanism of action^2,3^.

Therapeutic peptides possess many advantages over traditional drugs. They are more efficacious, selective and specific than small molecules, and their products of degradation are amino acids, which present a reduced risk of drug-drug interactions. Additionally, their short half-life means a lower propensity for accumulation in tissues^4^. Their immediate response and potent activity against various pathogens, including bacteria, fungi, parasites and viruses, means that these compounds can be utilised both as substitutes and as part of a combination therapy with conventional antibiotics^5^. Furthermore, therapeutic peptides have been identified for other applications, such as cancer, immune disorders, cardiovascular diseases, gastrointestinal dysfunction, hemostasis and diabetes^6–9^.

Although therapeutic peptides were initially isolated from plants or animals that secrete them as part of their host defence mechanism^10^, they can now also be obtained from genetic^11^, recombinant^12^ and chemical^13^ libraries as well, which presents a largely unexplored chemical space, with only a limited number of peptide-based drugs currently available on the market. Among those are Enfuvirtide, Leuprolide, Bacitracin and Boceprevir, which act against HIV^14^, prostate cancer^15^, pneumonia^16^ and hepatitis-C^17^, respectively.

Many peptides never reach clinical trials because of a therapeutic potential that’s hindered by low metabolic stability, poor oral bioavailability, or a poor toxicity profile, which is typically assessed by measuring the activity that the peptide exerts against eukaryotic erythrocytes^18^. Peptide modifications such as substitution with D-amino acids have been proved to improve peptide stability to proteolysis^19^. Toxicity can be divided into three classes: immunotoxicity, cytotoxicity and hemotoxicity. It is the aim of this work to develop a method of predicting peptides’ hemotoxicity prior to their chemical synthesis.

**Figure 1:**
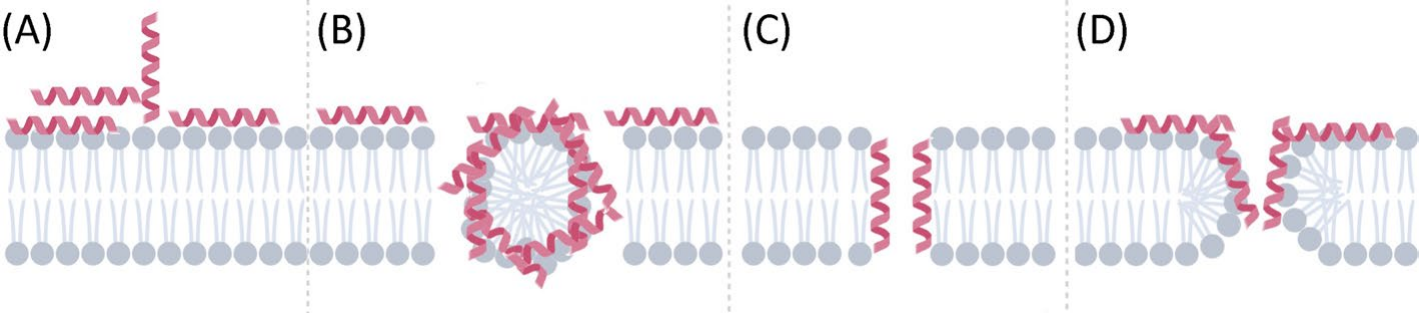
Schematic representation of the interactions between the peptides (red) and the lipid bilayer (grey) during (**A**) initial approach and binding, (**B**) carpet model, (**C**) barrel stave model and (**D**) toroidal pore model.

AMPs typically, but not exclusively, possess amphipathic structures with a positively charged and a hydrophobic interface, and primarily exert their activity at the charged surface of the bacterial plasma membrane. A number of mechanisms of action have been characterised to date; the most commonly observed mechanisms of action are the pore-forming barrel-stave and toroidal-pore models, and the non-pore-forming carpet model, which are illustrated in Fig. 1^20^. All three mechanisms share the development of an electrostatic attraction between the negatively charged membrane phospholipid headgroups and the positive charge of AMPs^21^ as their initial step. Once the AMP is in close proximity to the membrane, it establishes hydrophobic interactions between its hydrophobic face and the membrane’s hydrophobic interior. Interestingly, anionic AMPs have been identified despite the general requirement for cationicity; these peptides instead possess an increased hydrophobicity profile^22^. Barrel-stave model AMPs form a barrel shape in the cell membrane in which hydrophobic residues are juxtaposed with lipid chains and hydrophobic residues which line the central pore. AMPs which employ the toroidal pore model of action coerce the membrane lipids to bend in a manner such that the AMPs are positioned nearest to the phospholipid head groups which shape the pore diameter. The carpet model, meanwhile, requires for membrane thinning to occur between the anionic membrane phospholipids and the cationic AMPs. Following insertion, AMPs induce a change in the cell membrane polarization, which results in an increase in membrane permeability and a reduction of the transmembrane electrical potential^23^.

The basis for selectivity of AMPs lies in the different composition of the prokaryotic and eukaryotic cell membranes. Bacterial cell membranes are rich in phospholipids, including phosphatidylglycerol, phosphatidylserine, phosphoglycerol, gangliosides, phosphatidylcholine, sphingomyelin^24^, but do not contain cholesterol, like eukaryotic membranes. Classification of peptides as hemolytic or non-hemolytic is complicated as most antimicrobial peptides exert their activity at the plasma membrane. The differentiator between hemolytic and non-hemolytic peptides is whether or not they are active at the zwitterionic eukaryotic membrane as well as the anionic prokaryotic membrane.

In silico methods provide a more efficient avenue for the screening of the large chemical space and accelerate drug design by reducing the number of peptides that have their hemolytic activity screened experimentally. Deep learning has proven itself useful in other areas of bioinformatics, with numerous examples such as DeepPPISP for the prediction of protein-protein interaction sites^25^, SCLpred for protein subcellular localization prediction^26^ and CPPpred for prediction of cell-penetrating peptides^27^. As a number of databases exist that detail the biological activities of peptides, such as DBAASP^28^, CAMP^29^ and Hemolytik^30^, we have exploited the available data to train a deep neural network that classifies peptides as hemolytic or non-hemolytic based on their primary sequence. Herein we present a novel method for the prediction of the hemolytic activity of antimicrobial peptides.

## Methods

### Datasets

The HAPPENN dataset consists of 3,738 peptides sequences between 7–35 amino acids in length and their corresponding hemolytic activities. All sequences are composed of exclusively natural amino acids, and the only modifications included are N-terminal acetylation and C-terminal amidation. Secondary structure properties are not considered in the creation of the dataset. The dataset is available as supplementary material.

3,408 of these peptide sequences were extracted from the DBAASP database^28^ and 1,174 from the Hemolytik database^30^. 844 peptide sequences were present in both databases. Of the sequences extracted from DBAASP, 861 were ribosomally synthesised, while 2,547 were chemically synthesised.

The dataset consists of 1,543 experimentally validated hemolytic peptides and 2,195 experimentally validated non-hemolytic peptides, as determined by criteria detailed in Table 1.

#### Redundancy reduced dataset

The HAPPENN dataset was internally redundancy reduced using CD-HIT^31–33^, removing sequences so that no two sequences were $\ge 90\%$ similar to each other, which yielded the HAPPENN-RR90 dataset, which consists of 823 experimentally validated hemolytic peptides, and 1,100 experimentally validated non-hemolytic peptides.

#### Dataset for additional benchmarking

Discriminating compositionally similar peptides with different biological activities is one of the greatest challenges in developing prediction methods^34,35^. An additional dataset was created, HAPPENN-hard, wherein positive examples are the experimentally validated hemolytic peptides of HAPPENN-RR90, and the negative examples are experimentally validated non-hemolytic peptides exhibiting the greatest compositional similarity to the positive peptides. A negative sequence was deemed to be the most similar to a positive sequence if it possessed the minimum Euclidean distance to the positive sequence^36–38^.

**Table 1:** Criteria for designating a peptide as hemolytic or non-hemolytic. Peptides which satisfied neither or both of these criteria were excluded.

Hemolytic peptides		Non-hemolytic peptides	
Hemolytic activity (%)	Peptide conc. (μM)	Hemolytic activity (%)	Peptide conc. (μM)
50	$\le 300$	45	$>270$
55	$\le 330$	40	$>240$
60	$\le 360$	35	$>210$
65	$\le 390$	30	$>180$
70	$\le 420$	25	$>150$
75	$\le 450$	20	$>120$
80	$\le 480$	15	$>90$
85	$\le 510$	10	$>60$
90	$\le 540$	5	$>30$
95	$\le 570$	0	$>30$
100	$\le 600$		

### Model validation

It is critical that any classifier model created by machine learning is thoroughly validated. For that reason, tenfold cross-validations and validation by an external test set were employed to evaluate the performance of all models presented herein. The HAPPENN dataset was split into twelve parts, ten of which were used for cross-validation, whereby one of the subsets was selected for use in validation while the other nine were employed for training. The resultant models were ensembled and evaluated with an independent test set, which consists of the remaining two of the twelve parts. To avoid possible bias arising from the choice of a randomly selected test set, the procedure is repeated six times in total, allowing for a rigorous assessment of the model’s overall performance.

#### Validation comparison with HemoPI and HemoPred

The different dataset construction and validation procedure employed by HAPPENN compared to other available tools, namely HemoPI and HemoPred, prevents a direct comparison of their respective validation statistics. To facilitate a more direct comparison with the HemoPI and HemoPred classifiers, a model was trained and tested under equivalent conditions. An altered dataset, HAPPENN-HemoPI3-equiv was created, wherein all the peptide sequences present in the HAPPENN dataset that form part of the HemoPI-3 test dataset were set aside as the test dataset, and the remaining non-test set sequences were used for training and validation as part of a fivefold cross-validation.

### Amino acid composition analysis

An analysis of the amino acid composition of the hemolytic and non-hemolytic peptides was carried out, completed by an analysis of peptides randomly extracted from proteins in Swiss-Prot^39^. The analysis comprises the peptides’ full sequences, the 10 N-terminal residues, and the C-terminal 10 residues.

### Residue position preference analysis

Enrichment depletion logos (EDLogo)^40^ were created to identify preferences for certain amino acid residues at certain positions in the hemolytic peptides’ sequences. The logo plots were constructed using the experimentally validated non-hemolytic peptide sequences as the baseline.

### Motif analysis

Motif analysis was carried out on the HAPPENN dataset to identify motifs occurring exclusively in hemolytic and non-hemolytic peptides. Motifs with a length between 2–5 amino acids which occurred in at least ten peptides were considered.

### Features extraction

A large selection of features was extracted from the peptides’ primary sequences, which can be divided into two subcategories, amino acid composition based features and physicochemical descriptors.

#### Physicochemical descriptors

The modlAMP^41^, ChemoPy^42^ and RDKit packages were used for the calculation of global physicochemical descriptors, as well as amino acid scale-based descriptors.

Global physicochemical descriptors include sequence length, molecular formula, molecular weight, sequence charge, charge density, isoelectric point, instability index, aromaticity index^43^, aliphatic index^44^, Boman index^45^ and the hydrophobic ratio.

Meanwhile, amino acid scale-based descriptors include AASI^46^, ABHPRK^41^, hydrophobicity^47–51^, side-chain bulkiness^52^, amino acid charges, COUGAR^41^, Ez^53^, side-chain flexibility^54^, polarity^52,55^, ISAECI^56^, $\alpha$-helix propensity^57^, MSS^58^, MSW^59^, pepArc^41^, PPCALI^60^, refractivity^61^, t_scale^62^, transmembrane propensity^63^, z3^64^ and z5^65^.

Additionally, physicochemical descriptors were calculated from the amino acid properties in the AAindex^66^. Secondary structure related descriptors were calculated based on the turn^67^, helical^47, 68^, coil^69^ and amphiphilic^70^ propensities. The sequence hydrophobicity was quantified using the amino acids’ hydropathies^49,71^, hydrophobicities^72–76^, hydrophobic moments^77^, partition energies^78–80^ and retention coefficients in HPLC^81,82^. Similarly, the sequence hydrophilicity was characterised using properties based on the amino acids’ hydrophilicity^50^, charges^83^, polarities^84,85^, free energies of solution in water^77,86^, numbers of hydrogen bond donors^87^ and fractions of site occupied by water^88^. Descriptors relating to the amino acids’ sterics were calculated based on their residue volume^89–92^, residue flexibility^54^, steric hindrance^93^, bulkiness^52^, 8Å and 14Å contact numbers^94,95^, average reduced side-chain distance^96^ and accessible molar fractions ratio^97^ properties. As membrane interaction plays an important role in peptides’ hemolysis mechanism of action, features based on membrane-propensities^98^, membrane-buried preference parameters^47,99^ and the side-chain^100,101^ and electron-ion interactions^102,103^ were calculated. Descriptors were also calculated based on the number of full non-bonding orbitals^87^, SWIGM index^51^, and the IFH^104^ and z1^105^ scales.

#### Composition descriptors

Amino acid, dipeptide, and tripeptide compositions were calculated for the conventional 20-amino acid alphabet, as well as the reduced alphabets of Veltri et al.^106^, Thomas and Dill^107^, and the conjoint alphabet^108^. To account for the three-dimensional structure of the peptides, *g*-gap dipeptide and tripeptide compositions were calculated^109^. Finally, pseudo amino acid composition^110^, conjoint triad, composition, transition and distribution^111^ descriptors were also calculated.

### Machine learning approaches

Support vector machine (SVM)^112^, random forest (RF)^113^, principal component analysis (PCA)^114^, t-distributed Stochastic Neighbour Embedding (t-SNE)^115^ and dense fully connected neural networks^116^ are employed in this study.

Both a linear and non-linear (RBF) kernel were employed with SVMs. SVM and RF hyperparameters were tuned using a grid search in conjunction with the previously described cross-validation.

### Feature selection

Only features which were non-zero for at least 100 samples were retained. Furthermore, features were selected for retention by SVM and random forest.

Features importances were calculated individually for each of the splits during tenfold cross-validation using both support vector machines and random forests. Features which had SVM absolute weights near-zero ($< 0.05$) were excluded, as practised by Brank et al.^117^. Features which an ensemble of random forests decided were important (importance $> 0.0005$) were included.

### Neural network architecture

All input features are scaled to have minimum and maximum values of 0 and 1, respectively.

Both a randomized grid search and genetic algorithm were employed to identify the optimal neural network architecture and hyperparameters. The optimized neural network applies a Gaussian noise layer with a standard deviation of 0.03 to the input layers, which mitigates overfitting. The first hidden layer has 1024 nodes and the second hidden layer has 64 nodes. Batch normalization^118^ is applied before the ReLU activation function. Each hidden layer is followed by a Dropout layer, with a rate of 0.93, which aids in the prevention of overfitting^119^.

**Figure 2:**
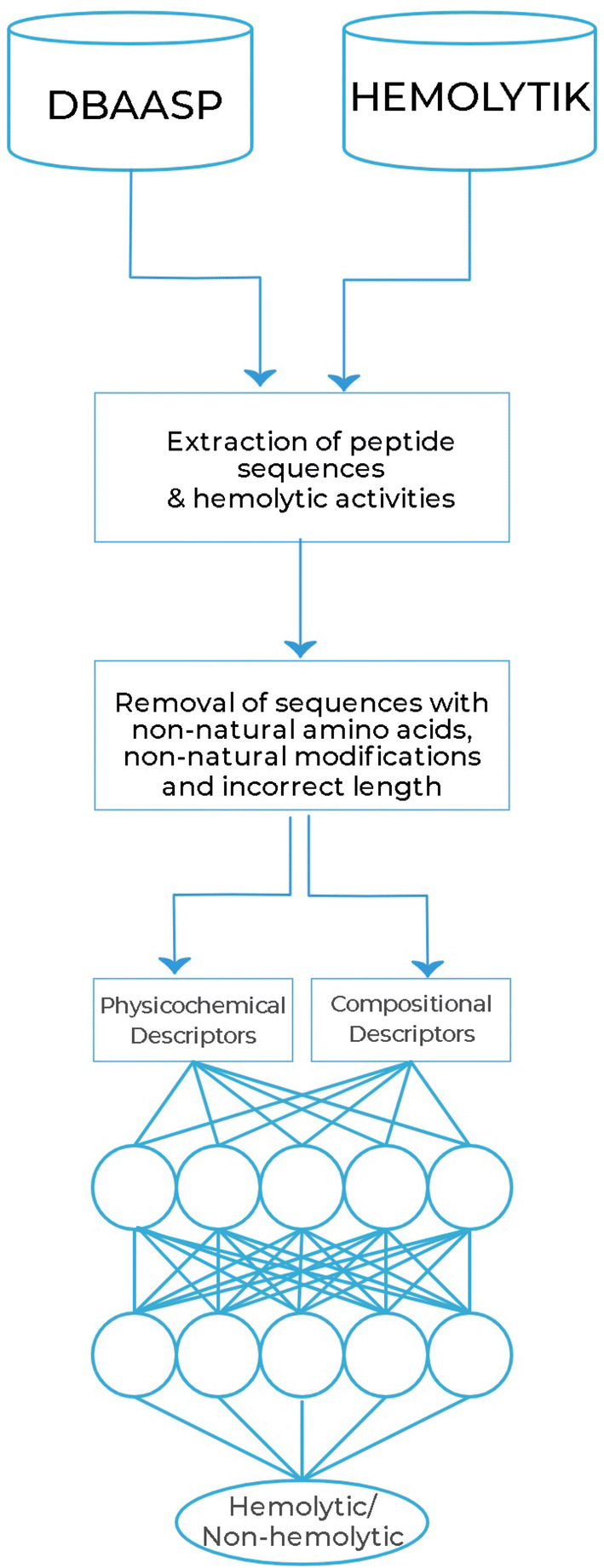
Summary of the model development architecture. Peptide sequences and their corresponding activities were extracted from databases, peptides outside the experiment’s scope were removed, and descriptors were calculated. The peptides’ descriptors are used as training input to a neural network with two hidden layers, which then predicts whether or not the peptide possesses hemolytic activity.

The final output layer consisted of a single node with a sigmoid activation function. A summary of the overall architecture described is shown in Fig. 2.

### Implementation

The neural network was implemented with Keras, a popular deep learning framework, using a Tensorflow^120^ back-end. The binary cross-entropy loss function was employed, which is defined as:

1 $$\begin{aligned} -\frac{1}{N}\sum _{i=1}^{N}[y_{i}\log (\hat{y_{i}}) + (1-y_{i})\log (1-\hat{y_{i}})] \end{aligned}$$

whereby $y_{i}$ is the true value of the $i^{th}$ sample, and $\hat{y_{i}}$ is the predicted value of the $i^{th}$ sample.

This loss function is commonly used in binary classification problems. As the predicted labels of all training data approach their respective true values, the value of the function approaches zero.

The optimizer employed is Adaptive Momentum (Adam), which updates the neural network weights according to the following formula^121^:

2 $$\begin{aligned} \Theta _{t+1} = \Theta _{t} - \frac{\eta {\hat{m}}_{t}}{\sqrt{{\hat{v}}_{t}} + \epsilon } \end{aligned}$$

whereby the $\hat{m_{t}}$ and $\hat{v_{t}}$ are the bias-corrected estimates of the mean and the variance of the gradients, respectively.

The neural network was trained for 600 epochs, without stopping criteria. The model with the highest validation accuracy encountered during training was saved for each of the cross-validation splits.

During training, the loss function was weighted to adjust for the slightly unequal number of positive and negative samples.

### Performance evaluation

The robustness of the predictor is evaluated by a number of standard parameters, namely accuracy (Acc), sensitivity (Sn), specificity (Sp), the Matthews correlation coefficient (MCC), and by the receiver operating characteristic (ROC) curve.

The first four of these are defined by the following equations:

3 $$\begin{aligned} Acc= \frac{TP + TN}{TP + TN + FP + FN} \times 100 \end{aligned}$$

4 $$\begin{aligned} Sn= \frac{TP}{TP + FN} \times 100 \end{aligned}$$

5 $$\begin{aligned} Sp= \frac{TN}{TN + FP} \times 100 \end{aligned}$$

6 $$\begin{aligned} MCC= \frac{TP \times TN - FP \times FN}{\sqrt{(TP + FP)(TP + FN)(TN + FP)(TN + FN)}} \end{aligned}$$

whereby

- TP = True positives: the number of correctly predicted positive (hemolytic) peptides.
- FP = False positives: the number of non-hemolytic peptides incorrectly predicted as being hemolytic.
- TN = True negatives: the number of correctly predicted negative (non-hemolytic) peptides.
- FN = False negatives: the number of hemolytic peptides incorrectly predicted as being non-hemolytic.

## Results

The HAPPENN dataset was constructed from peptide sequences whose hemolytic activity, or lack thereof, has previously been evaluated. Peptides were separated into a positive (hemolytic) class and a negative (non-hemolytic) class based on criteria outlined in Table 1. The peptide sequences were subjected to an amino acid composition analysis, residue position preference analysis and motif analysis. Peptides were then represented by feature vectors composed of physicochemical and compositional descriptors of the peptides. The feature vectors are visualised using principal component analysis (PCA) and t-stochastic neighbour embedding (t-SNE) plots, both of which show an incomplete separation of the positive and negative classes. Finally, the feature vectors are used to train support vector machine, random forest and neural network hemolytic activity classifiers, the prediction results of which are evaluated.

### Amino acid composition analysis

**Figure 3:**
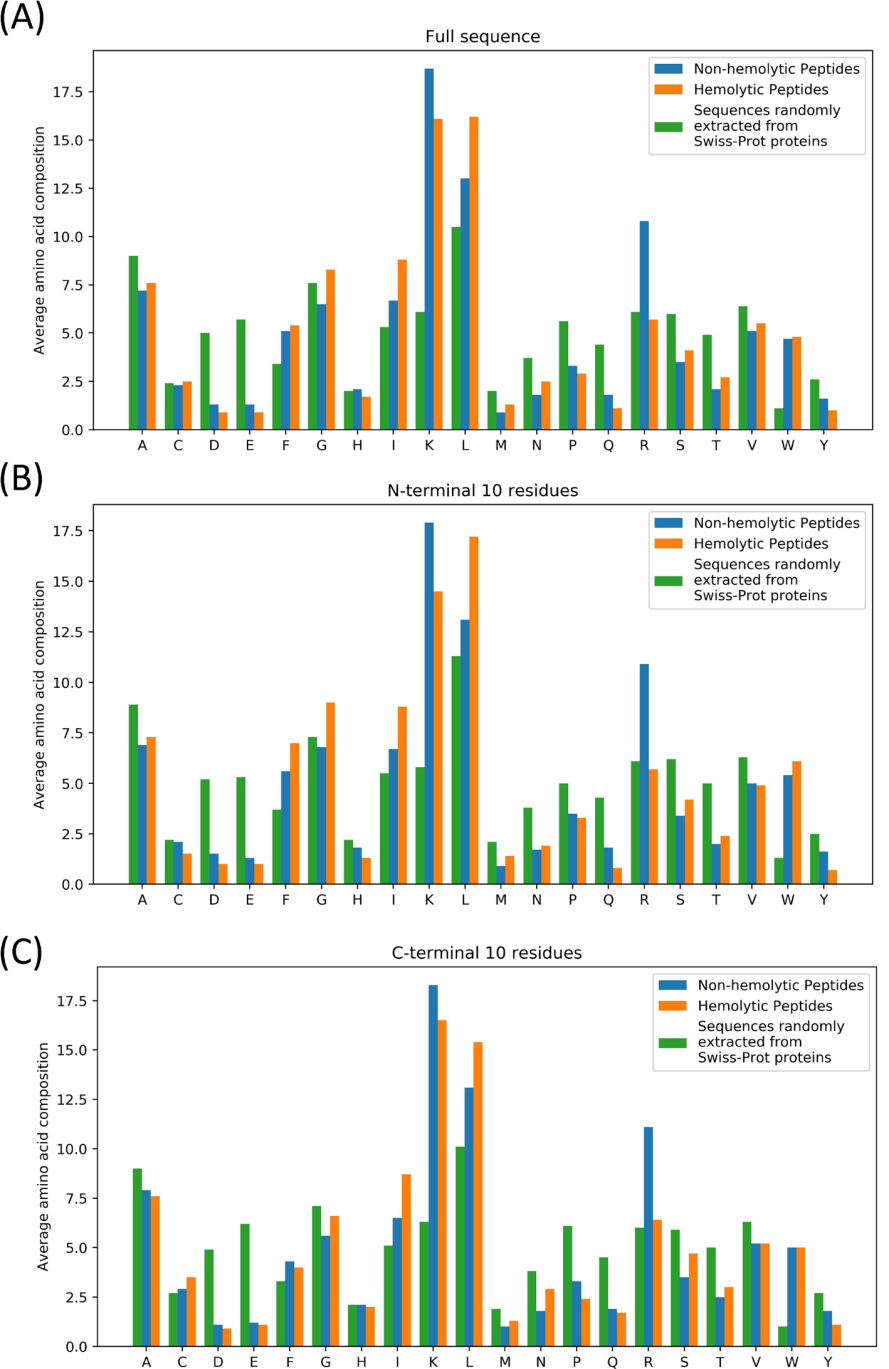
Percentage average amino acid residue composition of the (**A**) full sequences, (**B**) N-terminal 10 residues, and (**C**) C-terminal 10 residues of experimentally validated hemolytic peptides (orange), experimentally validated non-hemolytic peptides (blue) and peptide sequences randomly extracted from Swiss-Prot proteins (green).

To determine whether a preference exists for certain residues in hemolytic peptides compared to non-hemolytic peptides, an amino acid residue composition analysis was performed, the results of which are shown in Fig. 3. It is apparent that hemolytic peptides are most enriched in the hydrophobic leucine and isoleucine residues, and to a lesser extent phenylalanine, tryptophan and glycine. Meanwhile, non-hemolytic peptides are enriched in the positively charged lysine and arginine residues. Interestingly, both hemolytic and non-hemolytic peptides are depleted in the negatively charged aspartic and glutamic acid residues compared to the sequences randomly extracted from Swiss-Prot, with the hemolytic peptides exhibiting greater depletion.

### Residue position preference analysis

**Figure 4:**
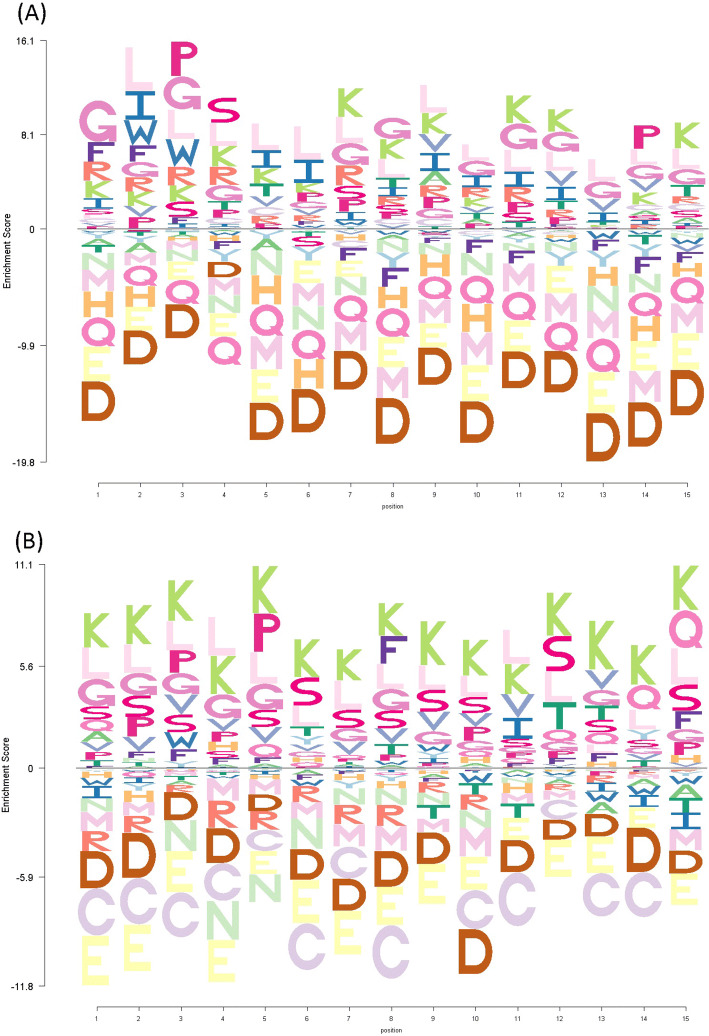
Enrichment-depletion logo plot of (**A**) N-terminal 15 residues and (**B**) C-terminal 15 residues of experimentally validated hemolytic peptides of the HAPPENN dataset. Data is scaled to account for the background probability of each amino acid, based on the experimentally validated non-hemolytic peptides.

To ascertain whether or not there exists a preference for certain residues at certain positions in the peptide sequence, an enrichment-depletion logo plot was created (Fig. 4) for the hemolytic peptide class, with the non-hemolytic peptide class serving as the baseline for the plot. Enriched residues, therefore, are those which are more common at that position in hemolytic peptides, relative to the non-hemolytic class, and depleted residues are those which are less common.

The first inspection of the EDlogo plot reveals information that is consistent with the amino acid composition analysis: hemolytic peptides are enriched in hydrophobic residues, and predominantly depleted in negatively charged residues. On further inspection, position-specific enrichments become apparent. Hemolytic peptides are enriched in the negatively charged aspartic acid residue at position 4, and at the last, third- and fourth- and eleventh-last position, despite being depleted in this residue for the remainder of the sequence. Hemolytic peptides are depleted in the positively charged arginine residue throughout the sequence, but enriched in lysine at positions 7, 8, 11, 12 and 15. A preference exists at the positions 2 and 3 for tryptophan, position 3 for proline, and positions 3 and 4 for serine. A notable preference exists at position 14 for proline, which is indeed a common feature in the brevinin-1 family of peptides, which do possess hemolytic activity^122^. Hemolytic peptides are also enriched in glutamine exclusively at their C-terminus, while being depleted in glutamine throughout the remainder of the sequence.

### Motif analysis

A motif analysis was undertaken on the HAPPENN dataset to identify any motifs present exclusively in hemolytic or non-hemolytic peptides. The top twenty motifs occurring exclusively in hemolytic peptides are ’LKHI’, ’KIIKV’, ’TLLKK’, ’VNWK’, ’GAIA’, ’VNWKK’, ’KKILG’, ’VLKAA’, ’LWKT’, ’ALWKT’, ’MAL’, ’KITK’, ’PKIF’, ’GKEV’, ’KIAS’, ’CKITK’, ’KHILK’, ’IKVV’, ’IKVA’, ’IASI’. The top twenty motifs occurring exclusively in non-hemolytic peptides are ’PRP’, ’RPRP’, ’AAAA’, ’PRPR’, ’PRPRP’, ’RPRPR’, ’AAAAA’, ’AFA’, ’AAFA’, ’AFAA’, ’LKYG’, ’WKI’, ’KYGK’, ’ILKYG’, ’LKYGK’, ’PRL’, ’RRKK’, ’AAFAA’, ’KPS’, ’RPG’.

### Data visualisation

#### Principal component analysis (PCA)

**Figure 5:**
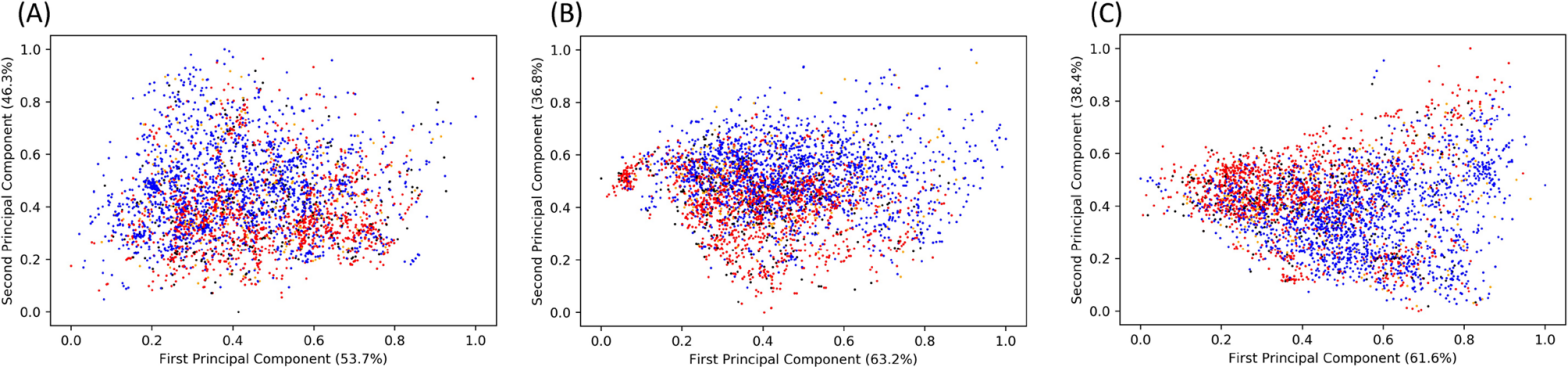
Principal component analysis of (**A**) all the computed descriptors, (**B**) only the physicochemical descriptors and (**C**) composition descriptors. Hemolytic peptides (positives) are coloured red, non-hemolytic peptides (negatives) are coloured blue, false-positives are coloured black, false-negatives are coloured orange.

Principal component analysis (PCA) was undertaken for the full computed dataset, the dataset with only the physicochemical features and the dataset with only composition descriptors (Fig. 5). Inspection of the results of all three indicates that while a separation exists between the hemolytic and non-hemolytic classes, the separation is not clear-cut and a significant overlap exists between the classes. The overlap between classes is most significant for the set consisting of only the physicochemical descriptors, while a greater separation between classes exists in the composition descriptor plot.

#### T-distributed stochastic neighbour embedding (t-SNE)

**Figure 6:**
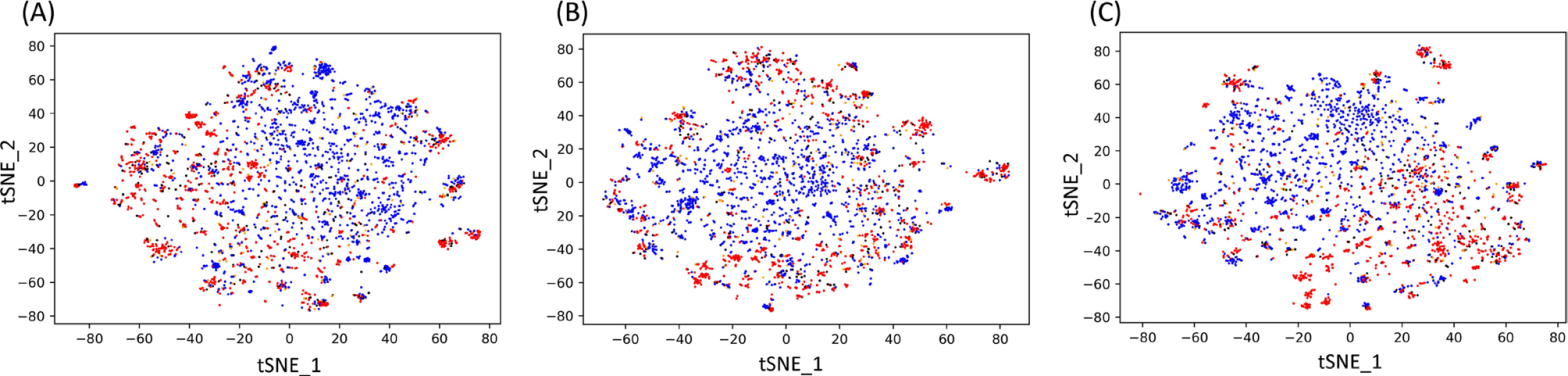
t-SNE visualisation of (**A**) all the computed descriptors, (**B**) only the physicochemical descriptors and (**C**) composition descriptors. Hemolytic peptides (positives) are coloured red, non-hemolytic peptides (negatives) are coloured blue, false-positives are coloured black, false-negatives are coloured orange.

Similarly to the aforementioned PCA analysis, a t-distributed Stochastic Neighbour Embedding (t-SNE) analysis was undertaken for the full computed dataset, the dataset with only the physiochemical features and the dataset with only composition descriptors (Fig. 6). As is the case with the PCA results, there exists an incomplete separation between the hemolytic and non-hemolytic classes in all three datasets. In many cases, positive and negatives peptides are near-coincident in the plots, and appear, therefore, to be physicochemically and compositionally similar.

### Hemolytic activity prediction

This novel study employed a number of popular machine learning classifiers for predicting peptides’ hemolytic activity on the basis of features calculated from their primary sequence. The predictive power was evaluated using tenfold cross-validation, and the final ensemble of ten neural networks was evaluated by means of external validation. Accuracy, sensitivity, specificity, Matthews correlation coefficient statistical parameters are reported with their confidence intervals. A receiver operating characteristic curve (ROC) with a calculated area under the curve (AUC) is also reported.

To the authors’ knowledge, three machine-learning based classifiers for the prediction of hemolytic activity peptides are described in the literature, namely HemoPI^123^, HemoPred^124^, and HemoPImod^125^. The former two predict the hemolytic activity of natural amino-acid-based peptides, while the latter specializes in predicting the hemolytic potency of chemically modified peptides. The results of the present study are compared to those of the former two classifiers, HemoPI and HemoPred. As HemoPImod specifically addresses chemically modified peptides, and therefore differs in its aims to HAPPENN, it is excluded from the comparisons.

#### Comparison of classifier methods

The prediction statistics achieved by support vector machine (SVM), random forest (RF) and neural network (NN) models are presented in Table 2.

**Table 2:** Validation and test results for the SVM, RF and NN models trained on the HAPPENN dataset.

Method	Acc	Sn	Sp	MCC
**Cross-validation**				
SVM (linear)	$81.22 \pm 2.58$	$76.87 \pm 4.06$	$84.26 \pm 3.13$	$0.61 \pm 0.05$
SVM (RBF)	$77.51 \pm 2.91$	$71.26 \pm 4.77$	$81.94 \pm 3.44$	$0.54 \pm 0.06$
RF	$83.30 \pm 2.35$	$77.68 \pm 4.10$	$87.26 \pm 2.39$	$0.65 \pm 0.05$
NN	$85.66 \pm 1.93$	$84.96 \pm 3.37$	$86.09 \pm 3.43$	$0.71 \pm 0.04$
**External validation**				
SVM (linear)	$81.49 \pm 1.80$	$76.77 \pm 2.46$	$84.85 \pm 2.13$	$0.62 \pm 0.04$
SVM (RBF)	$77.79 \pm 2.11$	$71.33 \pm 2.78$	$82.37 \pm 2.97$	$0.54 \pm 0.04$
RF	$84.06 \pm 1.38$	$78.56 \pm 2.69$	$87.96 \pm 1.75$	$0.67 \pm 0.03$
NN	$84.00 \pm 1.67$	$82.85 \pm 2.31$	$84.86 \pm 2.23$	$0.67 \pm 0.03$

The SVM hyperparameters were optimised using a grid search. The linear kernel SVM achieved its highest performance with the regularization parameter $C=0.1$. The non-linear RBF kernel SVM achieved its highest performance with the regularization parameter $C=10$ and the kernel coefficient $\gamma = 2 \times 10^{-4}$. Both the RBF and linear kernel SVM approaches achieve the worst level of performance of the three methods studied, with a validation accuracies of 78% and 81%, and MCCs of 0.54 and 0.61, respectively.

The RF hyperparameters were also optimised using a grid search. The highest performance, with an accuracy and MCC of 83% and 0.65 was achieved with the number of estimators set to be 1024, with unrestricted tree depth. The optimal value for max_features was found to be 70.

**Figure 7:**
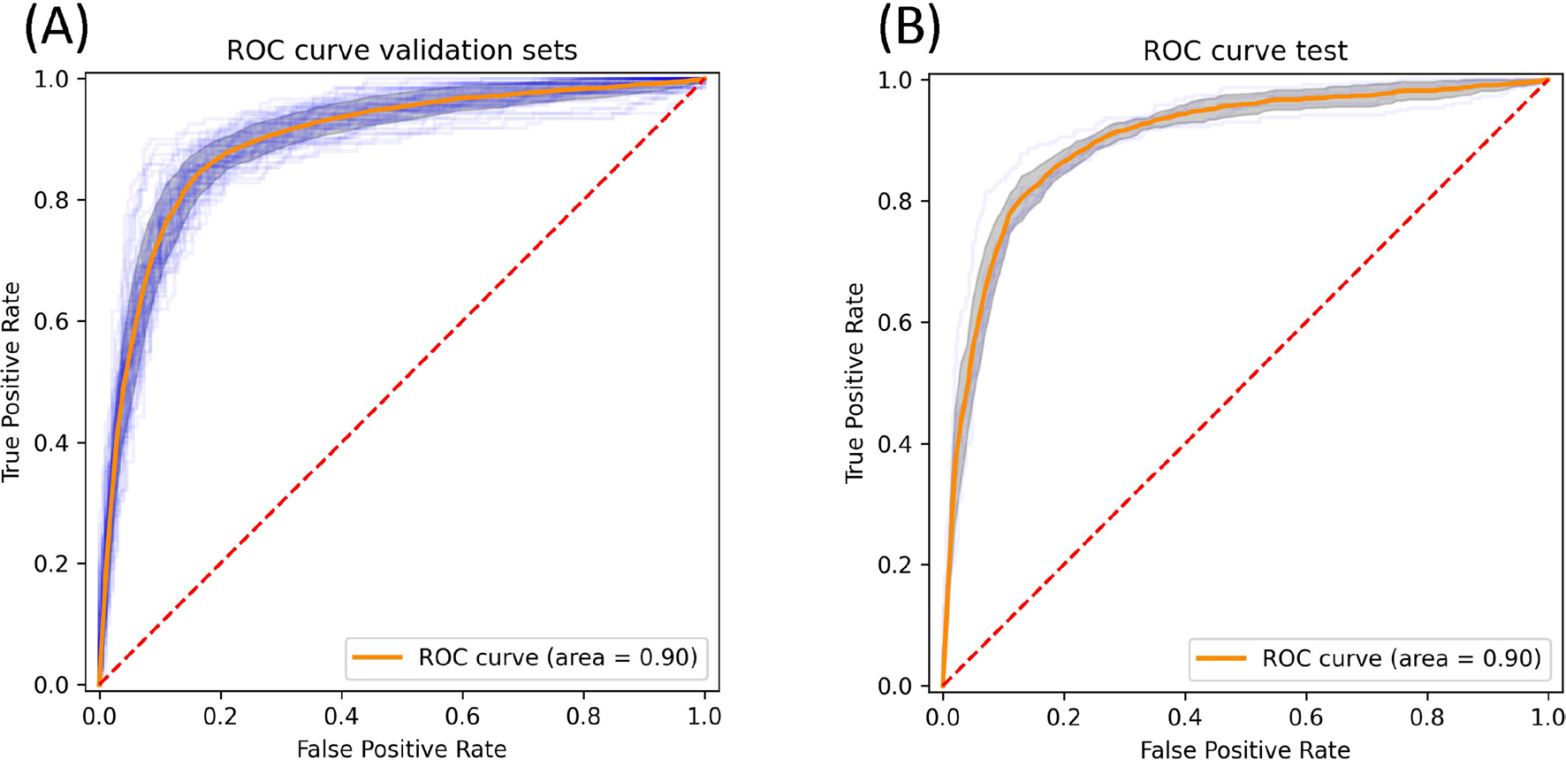
Receiver operating characteristic plot of HAPPENN performance on (**A**) the tenfold cross-validation sets and (**B**) the external validation.

The neural network approach, meanwhile, achieves the highest accuracy and MCC score, with scores of 86% and 0.71, respectively, marking it as the most capable predictor. Furthermore, the neural network approach achieves the best balance between sensitivity and specificity. As it was the most capable, the neural network approach was selected as the classifier of choice for the prediction of hemolytic activity. The predictive power of HAPPENN was further evaluated by means of the receiver operating characteristic (ROC) curve, and its associated area under the curve (AUC) (Fig. 7), which is equivalent to the probability that the predictor will rank a randomly selected positive instance higher than a negative one. We note that the performance is nearly excellent on both the validation and test sets, with both yielding an AUC of 0.90.

#### Comparison to HemoPI and HemoPred

HemoPI and HemoPred are in silico peptide hemolytic activity prediction models previously reported in the literature, to which the HAPPENN model is compared.

The former approach employs a support vector machine (SVM) trained on a combination of single residue-, dipeptide- and property-based features, while the latter employs a random forest (RF) trained on a combination of amino acid and dipeptide composition features. Both models achieve similar cross-validated accuracies and MCC scores not exceeding 78% and 0.56 when trained on the HemoPI-2 and HemoPI-3 datasets.

The HAPPENN model achieves good validation statistics, with a tenfold cross-validated accuracy of 85.66% and MCC value of 0.71. The HAPPENN approach achieves prediction performance that significantly exceeds that of both HemoPI and HemoPred (Table 3), although the cross-validation scheme and test set used differ.

In order to facilitate a more direct comparison, an altered dataset was created, termed HAPPENN-HemoPI3-equiv, wherein the test dataset consists exclusively of HAPPENN dataset peptides which also form part of the HemoPI-3 test set. The remaining non-test set peptides were used for training and validation. Using this altered dataset, a neural network sharing the architecture and hyperparameters of the main HAPPENN neural network was trained under fivefold cross-validation, achieving a test set accuracy of 84.96% and an MCC of 0.70. While these results again exceed those of the available classifiers, it should be noted that the test set in this case is not truly independent as its constituent peptides had been previously used in optimising the hyperparameters of the main HAPPENN neural network.

**Table 3:** Validation and test results for HemoPI, HemoPred and HAPPENN. HemoPI, HemoPred and HAPPENN-HemoPI3-Equiv datasets are subjected to fivefold cross-validation, while HAPPENN employs tenfold cross-validation for the HAPPENN, HAPPENN-RR90 and HAPPENN-hard datasets.

Dataset	Classifier	Acc (%)	Sn (%)	Sp (%)	MCC
**Cross-validation**					
HemoPI-2	HemoPI	78.0	78.3	77.6	0.56
	HemoPred	$76.18 \pm 0.40$	$76.57 \pm 0.34$	$75.66 \pm 0.53$	$0.52 \pm 0.01$
HemoPI-3	HemoPI	77.98	79.24	76.48	0.56
	HemoPred	$77.60 \pm 0.70$	$77.91 \pm 0.94$	$77.18 \pm 0.58$	$0.55 \pm 0.01$
HAPPENN	HAPPENN	$85.66 \pm 1.93$	$84.96 \pm 3.37$	$86.09 \pm 3.43$	$0.71 \pm 0.04$
HAPPENN-RR90	HAPPENN	$82.73 \pm 2.73$	$83.38 \pm 4.82$	$82.19 \pm 4.22$	$0.65 \pm 0.05$
HAPPENN-hard	HAPPENN	$77.54 \pm 3.31$	$82.12 \pm 6.41$	$72.45 \pm 7.74$	$0.55 \pm 0.06$
HAPPENN-HemoPI3-equiv	HAPPENN	$85.44 \pm 1.23$	$83.45 \pm 3.16$	$86.79 \pm 1.94$	$0.70 \pm 0.02$
**External validation**					
HemoPI-2	HemoPI	75.7	78.2	78.3	0.51
	HemoPred	$76.82 \pm 3.40$	$78.91 \pm 3.82$	$74.29 \pm 6.62$	$0.53 \pm 0.07$
HemoPI-3	HemoPI	77.16	81.92	71.43	0.54
	HemoPred	$79.91 \pm 0.68$	$85.20 \pm 2.09$	$73.33 \pm 1.76$	$0.59 \pm 0.01$
HAPPENN	HAPPENN	$84.00 \pm 1.67$	$82.85 \pm 2.31$	$84.86 \pm 2.23$	$0.67 \pm 0.03$
HAPPENN-RR90	HAPPENN	$80.65 \pm 2.41$	$81.75 \pm 4.30$	$79.84 \pm 1.96$	$0.61 \pm 0.05$
HAPPENN-hard	HAPPENN	$73.94 \pm 2.74$	$78.26 \pm 3.56$	$69.49 \pm 2.46$	$0.48 \pm 0.06$
HAPPENN-HemoPI3-Equiv	HAPPENN	$84.96 \pm 0.53$	$84.67 \pm 1.19$	$85.27 \pm 0.63$	$0.70 \pm 0.01$

#### Relationship between prediction and hemolytic activity values

Peptides were classified as hemolytic or non-hemolytic based on criteria given in Table 1, which relates hemolytic activity (H) to concentration (c). A peptide’s concentration can be expressed as a multiple (x) of the threshold concentration *c*. For instance, a peptide which exhibits 65% hemolytic activity at 195 μM can be said to have $x = 0.5$, as 0.5 × 390 μM = 195 μM. As *x* is less than 1, the concentration is lower than the threshold concentration (390 μM  for 65% hemolytic activity), and the peptide is considered hemolytic.

The neural network’s output is obtained from the sigmoid activation function of its final layer. As the sigmoid function produces values ranging between 0 and 1, these output values can be interpreted as the probability of a peptide being non-hemolytic (0) and hemolytic (1).

**Figure 8:**
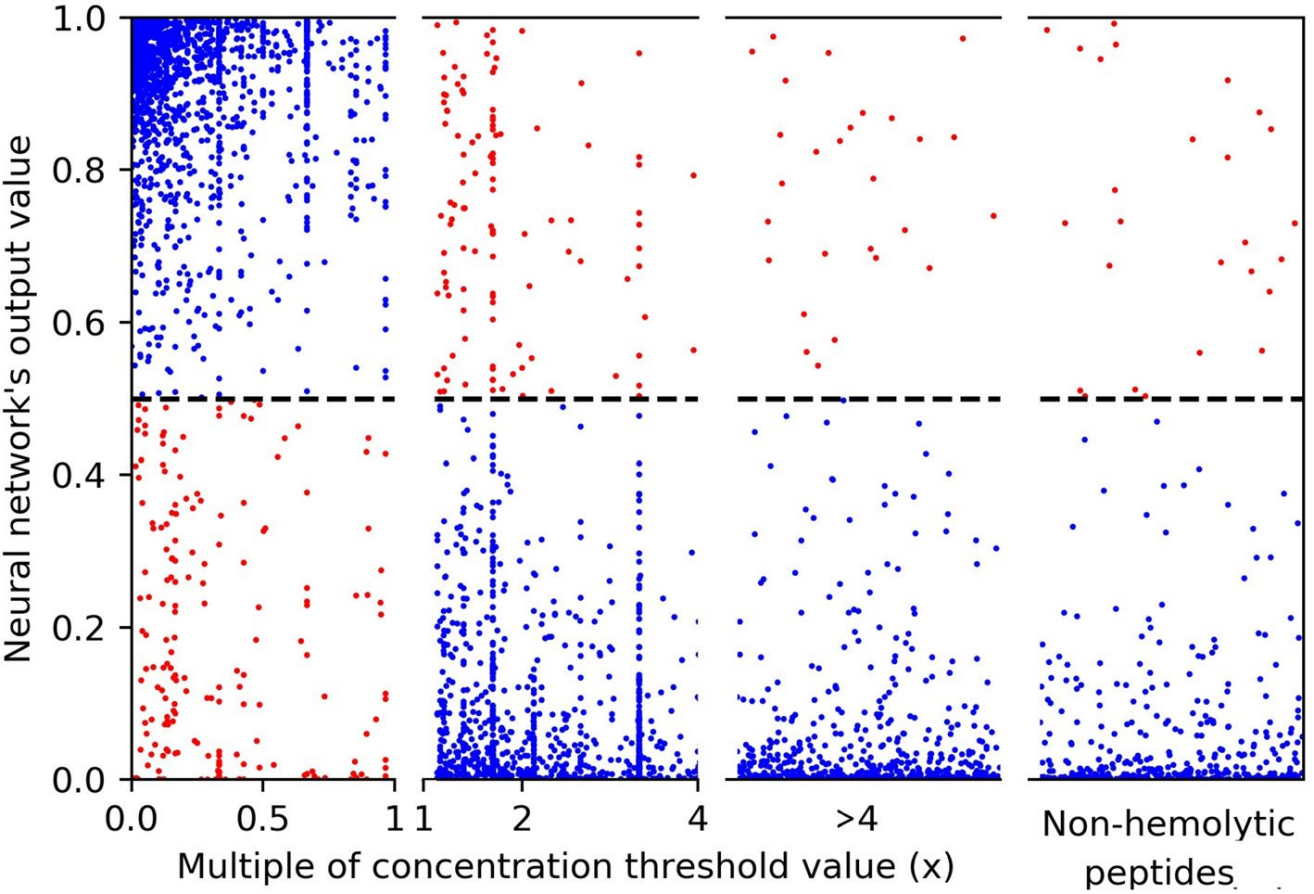
Plot of the neural network’s output values against *x*, the peptides’ multiple of the threshold concentration. Where $x < 4$, the values are presented to-scale. Where $x > 4$, the values are presented not-to-scale. Peptide’s which the literature states possess no hemolytic activity are presented separately, also not-to-scale. Correctly predicted peptides are coloured blue, incorrectly predicted peptides are coloured red.

The relationship between the neural network’s output values and *x*, the multiple of the threshold concentration, are shown in Fig. 8. The upper left quadrant shows the true positives, the lower right quadrant shows the true negatives, and the upper right and lower left quadrant show the false negatives and false positives, respectively.

It can be seen from Fig. 8, that not many peptides are found at the $y=0.5$ hemolytic-non-hemolytic prediction boundary. While there are many peptides at the $x=1.0$ activity boundary, most peptides are seen to be correctly predicted.

### Descriptor-set specific results

Several approaches were trialled for constructing the input feature space (Table 4), namely dipeptide and tripeptide composition, the corresponding *g*-gap compositions, N- and C-terminus composition and physicochemical features.

**Table 4:** Validation statistics achieved by neural networks trained with just single sets of descriptors.

Dataset	Features	Acc (%)	Sn (%)	Sp (%)	MCC
**Cross-validation**					
HAPPENN	Composition, dipeptide	$82.56 \pm 2.42$	$82.59 \pm 3.52$	$82.50 \pm 3.12$	$0.65 \pm 0.05$
	Composition, tripeptide	$82.62 \pm 1.74$	$80.46 \pm 4.45$	$84.06 \pm 2.27$	$0.64 \pm 0.04$
	g-gap composition, dipeptide	$84.02 \pm 2.38$	$83.87 \pm 3.14$	$84.13 \pm 3.20$	$0.68 \pm 0.05$
	g-gap composition, tripeptide	$83.55 \pm 1.85$	$82.07 \pm 3.06$	$84.54 \pm 2.86$	$0.66 \pm 0.04$
	Termini	$82.11 \pm 2.00$	$80.92 \pm 3.75$	$82.95 \pm 2.58$	$0.63 \pm 0.04$
	Physicochemical	$80.63 \pm 2.60$	$82.02 \pm 5.13$	$79.64 \pm 4.27$	$0.61 \pm 0.05$
HAPPENN-RR90	Composition, dipeptide	78.05 ± 2.57	81.25 ± 4.90	75.67 ± 3.86	0.56 ± 0.05
	Composition, tripeptide	$77.47 \pm 2.62$	$72.79 \pm 8.53$	$80.92 \pm 5.63$	$0.54 \pm 0.06$
	g-gap composition, dipeptide	$79.52 \pm 2.79$	$81.50 \pm 6.21$	$78.04 \pm 4.86$	$0.59 \pm 0.06$
	g-gap composition, tripeptide	$78.61 \pm 2.75$	$78.49 \pm 6.16$	$78.68 \pm 4.90$	$0.57 \pm 0.06$
	Termini	$79.38 \pm 3.01$	$80.39 \pm 6.52$	$78.63 \pm 3.02$	$0.59 \pm 0.06$
	Physicochemical	$79.02 \pm 4.42$	$81.70 \pm 6.77$	$77.01 \pm 5.75$	$0.58 \pm 0.09$
**External validation**					
HAPPENN	Composition, dipeptide	$81.42 \pm 1.61$	$81.25 \pm 1.51$	$81.58 \pm 2.49$	$0.62 \pm 0.03$
	Composition, tripeptide	$81.42 \pm 1.01$	$79.91 \pm 1.73$	$82.49 \pm 1.11$	$0.62 \pm 0.02$
	g-gap composition, dipeptide	$82.98 \pm 1.76$	$82.76 \pm 2.47$	$83.17 \pm 1.64$	$0.65 \pm 0.04$
	g-gap composition, tripeptide	$81.80 \pm 1.17$	$80.53 \pm 1.59$	$82.72 \pm 1.19$	$0.63 \pm 0.02$
	Termini	$81.34 \pm 1.15$	$80.02 \pm 3.18$	$82.33 \pm 1.26$	$0.62 \pm 0.02$
	Physicochemical	$79.30 \pm 1.82$	$80.93 \pm 3.89$	$78.21 \pm 2.27$	$0.58 \pm 0.04$
HAPPENN-RR90	Composition, dipeptide	$76.92 \pm 1.43$	$80.03 \pm 2.43$	$74.62 \pm 1.69$	$0.54 \pm 0.03$
	Composition, tripeptide	$76.44 \pm 0.96$	$74.63 \pm 4.15$	$77.79 \pm 2.57$	$0.52 \pm 0.02$
	g-gap composition, dipeptide	$78.14 \pm 1.82$	$79.00 \pm 2.75$	$77.47 \pm 3.37$	$0.56 \pm 0.03$
	g-gap composition, tripeptide	$77.16 \pm 1.80$	$77.06 \pm 3.52$	$77.28 \pm 1.17$	$0.54 \pm 0.04$
	Termini	$78.07 \pm 2.54$	$78.30 \pm 3.88$	$77.93 \pm 2.11$	$0.56 \pm 0.05$
	Physicochemical	$77.11 \pm 3.58$	$79.33 \pm 5.38$	$75.47 \pm 3.46$	$0.54 \pm 0.07$

#### Dipeptide and tripeptide composition

Dipeptide composition is defined as the proportion of a given dipeptide in the sequence, while similarly the tripeptide composition is defined as the proportion of a given tripeptide in the sequence. These composition features capture both the chemical nature of the peptide composition while retaining information about the local sequence order. The models achieved respectable accuracies of 82.56% and 82.62%, respectively, and MCC values of 0.65 and 0.64, respectively.

#### *g*-gap composition

*g*-gap dipeptide composition is described as the proportion of a given pair of amino acids separated by 1, 2 or 3 residues, which corresponds to residues which are adjacent in three-dimensional space, especially in regular secondary structures where such non-adjoining residues may be connected by hydrogen bonds. Interestingly, models trained on these features perform better than those trained on the more conventional dipeptide and tripeptide compositions. This can be attributed to these features better capturing the chemical environment that the peptide exposes to the membrane upon contact. For instance, the *g*-gap feature can represent the spatial adjacency separated by one turn of the $\alpha$-helix, which has a turn of 3.6 residues.

#### Termini

A feature set was created to capture the information on the residues specific location in the sequence. This feature set consists of a binary profile for the first and last 15 residues of each peptide. Each peptide is therefore represented by a number of vectors, the first of which represents the conventional 20 amino acid alphabet is of length $30\times 20$, and the remaining vectors represent the conjoint alphabet and the reduced alphabets of Veltri, Thomas and Dill. The model trained on this feature set achieved an accuracy of 82.11% and an MCC of 0.64.

#### Physicochemical

Interestingly, the worst-performing model is the model trained on the physicochemical features, achieving an accuracy of only 80.63% and an MCC of 0.61 on the non-redundancy reduced dataset.

Of the single feature-set approaches trialled, none outperform the model trained on the full feature set. The compositional descriptor-based models are seen to benefit from sequence similarity to an extent, and exhibit somewhat reduced performance on the redundancy-reduced dataset. The physicochemical descriptor-based model, conversely, maintains a comparable performance even on the redundancy-reduced dataset.

### Feature importance analysis

#### Random forest feature importance

Random forests have the advantage of being easily interpretable and provide an easy method of ranking the importance of input features. The most useful features as determined by cross-validated random forests are the Eisenberg direction of the hydrophobic moment (EISD860103)^77^, the Eisenberg normalized hydrophobicity scale (EISD840101)^73^, hydropathy (NADH010102, NADH010103, NADH010104)^71^, the hydrophobic parameter pi (FAUJ830101)^72^, the Boman index^45^, apparent partition energies (GUYH850105)^80^, membrane-propensity (PUNT030102)^98^, transmembrane propensity^63^, side-chain hydrophobicity values (BLAS910101)^76^ and Hopp-Woods hydrophobicity (HOPT810101)^50^.

Effectively all of these features directly or indirectly quantify hydrophobicity, which points to it being important for hemolytic activity.

#### Analysis of neural network weights using Garson’s method

In order to understand the basis for the neural network’s predictive power, we analysed the importance assigned to various input features by Garson’s method^126^, iteratively reducing the feature input space by approximately halving the number of input features, until  300 composition features were retained in each split. 24 features were identified as having large weights in each of the 10 splits.

The occurrences of the FS, LH, KIK, VAK, VLK dipeptides and tripeptides in the peptide sequence were found to be important. Additionally, the LLL Veltri reduced alphabet tripeptide and the RGV and VCR Thomas and Dill (length 3) reduced alphabet tripeptides were found to contribute strongly to the final classification.

Additionally, the occurrence of *g*-gap *i,i+3* residue pairs FG, FL, LK, WV, the occurrence of *g*-gap *i, i+4* residue pair FK and the occurrence of *g*-gap *i, i+2* residue pairs AR, FL, FS, LF were found to be meaningful.

Furthermore, the occurrence of the *g*-gap *i,i+3* Thomas and Dill (10) reduced alphabet residue pairs PS and WW and *g*-gap *i,i+3* Veltri reduced alphabet residue pair QQ were also important.

Finally, the network weights associated with the EstateVSA3 and EstateVSA4 (MOE-type descriptors using Estate indices and surface area contributions), Geary autocorrelation-lag8 weight by atomic polarizabilities, and the acetylation of the N-terminus inputs were also large.

Interestingly, the predictive power of the reduced feature space neural network is nearly as strong as the main network.

## Discussion

A decreasing number of drug approvals and a rising research and development cost base has contributed to a resurgence of interest in peptide therapeutics. An ideal peptide drug should possess a high therapeutic index, specifically high activity against the biological target and limited toxicity. The therapeutic potential of peptides, however, is highly dependent on it possessing little to no hemolytic activity. Minimizing hemolytic activity is important for improving the therapeutic index of a peptide.

Many research groups have studied the structures of natural peptides as well as engineered peptide analogues in order to characterise how their structure determines their biological activities^127–129^. A comprehensive understanding of the relationship between structure and function, however, remains elusive. A computational method that can provide information about a peptide’s biological activity from its primary structure prior to chemical synthesis, however, would allow for rapid and efficient exploration of the chemical space and present a significant cost and-time saving.

To accelerate the lead molecule design and optimization pipeline, this study aimed to create an in silico method for classifying therapeutic peptides as hemolytic or non-hemolytic based on their primary sequence. The prediction task is challenging, however, as it requires distinguishing between desirable activity at the peptide’s target, the prokaryotic plasma membrane in the case of most antimicrobial peptides, and activity at the membrane of eukaryotic erythrocytes. The task is further complicated by the varying extent to which many peptides display hemolytic activity, which makes arbitrarily classifying them as hemolytic or non-hemolytic challenging. Indeed, there is limited consensus on the most appropriate metric to quantify hemolysis, with many articles reporting only one metric recorded at a single concentration, which consequently precludes a regression approach instead of a classification approach. The topic is complicated further by a lack of consensus on the definition of a key metric, MHC. Most studies define it as the minimum hemolytic concentration, but differ on the specific criteria, with different studies defining it as the concentration at which 5%, 10%, 50% or even 100% hemolysis occurs. Some even define it as the maximum concentration that does not cause any hemolysis^130^. Ideally, studies would present the analysis of hemolytic activity as a series of measurements undertaken at several concentrations, which would allow for a fuller understanding of the toxicity-concentration profile. Until such a time, however, using the MHC values for training classifiers requires investigating its actual meaning on a case by case basis. In the course of verifying the activity of the sequences in our dataset, and comparing our dataset to the HemoPI dataset, we identified a number of instances of misclassified sequences, sequences whose hemolytic activities were not clear, and sequences whose presence in the literature we were unable to independently verify. These sequences were not included in the HAPPENN dataset.

The success and validity of a machine learning classifier are predicated on the correct definition of the problem at hand, which in this case encompasses the definition of positive and negative datasets. Both the positive and negative datasets consist of experimentally validated peptide sequences which exhibit antimicrobial or other biological activities. Unlike HemoPI and HemoPred, we chose not to conduct a machine learning experiment where the negative dataset consists of peptides randomly extracted from proteins in Swiss-Prot, as a machine learning classifier is most dependable when only one property of interest is varied. Using randomly extracted sequences as the negative dataset, and hemolytic antimicrobial peptides as the positive set, likely results in the classifier learning to predict general membrane activity, rather than specifically activity against eukaryotic erythrocytes. The authors believe that the HAPPENN dataset represents a major improvement on the HemoPI datasets, both in terms of size and reliability, as it contains 3738 peptides with confirmed biological activities, compared to the 904 and 1623 sequences present in the HemoPI-2 and HemoPI-3 datasets, and therefore has been made available for download both as supplementary information and on the server’s website.

Once a reliable dataset was constructed, the peptide sequences were translated into vectors of physicochemical and composition features, and a number of different machine learning approaches, namely support vector machines, random forests and neural networks, were trialled for relating the peptides’ features to their hemolytic activities. The neural network approach proved most promising, and was therefore retained, further optimised, and had its predictive power thoroughly evaluated by means of tenfold cross-validation and external validation on an independent test set.

The final neural network model achieved a tenfold cross-validated accuracy, sensitivity and specificity of 85.66%, an MCC of 0.71, and an AUC of 0.90. The validation statistics demonstrated that the model is capable of discriminating between hemolytic and non-hemolytic peptides, and that it exhibits minimal bias towards one class or another. The model performs very well compared to the existing methods, with a 35.3% decrease in cross-validated error relative to HemoPI and HemoPred. The model’s residual prediction error rate can likely be attributed to a limited sample size for neural networks to accurately learn from, as well as the fine boundary between the definition of hemolytic and non-hemolytic peptides combined with the margin of error associated with the experimental determination of hemolytic activity. Further improvements to the predictive power of the neural network approach are possible and indeed expected, as the number of peptides in the literature with characterised hemolytic activity increases.

The main HAPPENN dataset was not redundancy-reduced, as even a single amino acid substitution can affect a peptide’s bioactivity. Nonetheless, to determine to what extent sequence similarity contributes to the model’s performance, the experiments were repeated with a redundancy-reduced dataset, which achieved a cross-validated accuracy of 82.73% and MCC of 0.65. While these values are lower than the non-redundancy-reduced dataset, they illustrate that the majority of the predictive power of the model is not derived from sequence similarity, especially considering that neural networks perform best when trained with larger datasets, and redundancy reduction significantly reduces the amount of data available for training. Finally, to ascertain the model’s power in distinguishing between similar peptides with different hemolytic activities, a model was trained on the HAPPENN-hard dataset, which contains the positive examples from the HAPPENN-RR90 dataset, and for each positive example, the most compositionally similar non-hemolytic peptide, as measured by the Euclidean distance between their amino acid composition vectors. Despite the similarity between the positive and negative peptides, the model achieves a respectable accuracy of 77.54% and MCC of 0.55.

Interestingly, the results of training a neural network with reduced feature spaces are in close agreement with the unsupervised principal component analysis (PCA) (Fig. 5) and t-distributed Stochastic Neighbour Embedding (t-SNE) (Fig. 6) analysis. The neural network trained on just the physicochemical features had the least predictive power among the networks trained, which coincides with the physicochemical features’ PCA plot having the least separation between the hemolytic and non-hemolytic classes. The relatively lower predictive power of the physicochemical descriptors highlights the need for the development of novel, peptide-specific descriptors that account for their capacity to adopt complex three-dimensional structures. When trained on the redundancy-reduced dataset, the difference in predictive power between the compositional and physicochemical descriptors is reduced.

To ascertain the source of misclassification of the wrongly predicted peptides, the main model’s false positives and false negatives were highlighted on the PCA and t-SNE plots. It is apparent that many of the misclassifications occur due to the peptides’ compositional and/or physicochemical similarity to peptides with differing hemolytic activity. To gain further insight into the source of misclassification, the peptide with the most similar percentage amino acid composition but opposite hemolytic activity was identified for each wrongly predicted peptide. For 16% of misclassified peptides, a compositionally identical peptide with opposite hemolytic activity was identified, compared with just 5% for correctly classified peptides. Overall, misclassified peptides had a smaller Euclidean distance to their most compositionally similar opposite-activity peptide than correctly classified peptides did.

To gain insight into which features were most important for hemolytic activity, the importance assigned to features by random forests was investigated. Hydrophobicity, as quantified by a selection of different metrics, appears to be critical for hemolytic activity, with more hydrophobic sequences generally being found to be more hemolytic than less hydrophobic sequences. These findings are not surprising, and are consistent with the available literature^131,132^. A number of compositional descriptors were also found to be indicative of hemolytic propensity, with FS, LH, KIK, VAK and VLK being ranked as important.

HAPPENN’s power is demonstrated by an alanine scan applied to maximin 3, a non-hemolytic peptide^127,133^. Interestingly, the classifier predicts maximin 3 and all of its alanine scan mutants to be non-hemolytic, with the single exception of [E20A]maximin 3, which is consistent with the literature, which acknowledges the relationship between a peptide’s net charge and its hemolytic activity^134^.

This study presents a significant improvement in the area of in silico hemolytic activity classification, with its results forming the new state-of-the-art. The novel application of a neural network combined with the HAPPENN dataset’s superior data quality and quantity has facilitated a 35% decrease in classification error, compared to the results achieved by the best currently available tools.

To conclude, accurate prediction of hemolytic activity of antimicrobial peptides can facilitate in silico design of novel peptide-based therapeutics, thereby accelerating the design phase and reducing its cost. HAPPENN distinguishes itself from existing methods through its focus on antimicrobial peptides, more accurate prediction and incorporation of novel features.

Although HAPPENN displays advantages compared to competing methods, it is limited by the lower interpretability of the neural network’s hidden layers. Prediction of hemolytic activity from primary sequence remains a challenging problem, as it is characterised by a complex interplay between numerous features, which also contribute to the desirable antimicrobial activities. Nonetheless, HAPPENN possesses an error rate 35% lower than the most accurate existing classifiers, and we believe that this work will aid future studies focused on the identification and design of novel peptide therapeutics.

## Web server implementation

To best serve the scientific community, we have made the classifier algorithm available online at https://research.timmons.eu/happenn in the form of an easy to use web-server, which is available for free use by academic researchers. The web server is capable of predicting the hemolytic activity of peptides’ based on their primary sequence, as well as the presence or absence of N-terminal acetylation or C-terminal amidation modifications. Prediction is limited to peptides composed of the 20 natural amino acids; non-natural amino acids are not supported. The web server possesses many features. Neural network models trained on the HAPPENN, HAPPENN-RR90 and HAPPENN-hard datasets are available for prediction.

### Hemolytic activity prediction

Hemolytic activity prediction is available for both single and multiple sequences. The user should submit the peptide sequence or sequences in FASTA format, select the neural network model they wish to use for prediction, and the server will return the probability of the peptide being hemolytic, based on the neural network’s prediction. This probability is on a scale of 0–1, where 0 is most probably non-hemolytic and 1 is most probably hemolytic.

### Mutation analysis

Mutation analysis is available for single sequences, provided in FASTA format. After inputting the sequence, the user should select the mutation analysis option, input the residue number that they wish to mutate, and run the prediction. The server will predict the hemolytic activity of each of the peptide’s mutants attained by substituting the residue at the selected position with each of the other natural 20 amino acids.

### Residue scan

A residue scan, for instance an alanine-scan, is available for single sequences provided in FASTA format. After inputting the sequence, the user should select the residue scan option, choose the residue they wish to scan with and run the prediction. The server will predict the hemolytic activity of each of the peptide’s mutants attained by substituting successive residue positions with the selected residue.

## Supplementary information

Supplementary file1 (PDF 815 kb)

## Data Availability

All data generated or analysed during this study are included in this published article’s supplementary data sets.
