# Supplementary material for: HAPPENN is a novel tool for hemolytic activity prediction for therapeutic peptides which employs neural networks
Source: Sci Rep. 2020 Jul 2;10:10869. doi: 10.1038/s41598-020-67701-3 (PMC7331684; doi:10.1038/s41598-020-67701-3)
Supplement: Supplementary file 1 — Supplementary file1 (PDF 815 kb) [file 41598_2020_67701_MOESM1_ESM.pdf]

```
>lcl|2826|lcl|HAPPENN&HAPPENN-RR90&HAPPENN-hard|lcl|subdataset_9|lcl|hemolytic|lcl|cTer
YKLLKLLLPKLKGLLFKL
>lcl|1766|lcl|HAPPENN&HAPPENN-RR90|lcl|subdataset_11|lcl|non-hemolytic|lcl|cTer
PFWRRRIRIRR
>lcl|793|lcl|HAPPENN|lcl|subdataset_10|lcl|non-hemolytic
GEKLKKIGKKIKNFFQKL
>lcl|1730|lcl|HAPPENN&HAPPENN-RR90|lcl|subdataset_11|lcl|non-hemolytic|lcl|nTer|lcl|cTer
KWKSFLKTFKSAKKTSLHTALKAISS
>lcl|293|lcl|HAPPENN&HAPPENN-RR90&HAPPENN-hard|lcl|subdataset_9|lcl|hemolytic
PAICQRATATLGTVGSNTSGTTEIAACILL
>lcl|119|lcl|HAPPENN&HAPPENN-RR90|lcl|subdataset_4|lcl|non-hemolytic
RCYCRRRFCVCR
>lcl|283|lcl|HAPPENN&HAPPENN-RR90&HAPPENN-hard|lcl|subdataset_8|lcl|hemolytic|lcl|cTer
GFLSILKKVLPKVMAHMK
>lcl|117|lcl|HAPPENN|lcl|subdataset_8|lcl|hemolytic
RGGRLCYCRRRFCICV
>lcl|4303|lcl|HAPPENN&HAPPENN-hard|lcl|subdataset_10|lcl|non-hemolytic|lcl|nTer|lcl|cTer
KWKSFLKTFKSAKKTVLHTAAKAISS
>lcl|3635|lcl|HAPPENN&HAPPENN-RR90|lcl|subdataset_9|lcl|non-hemolytic|lcl|cTer
FKLFKKILKVL
>lcl|4440|lcl|HAPPENN&HAPPENN-RR90|lcl|subdataset_12|lcl|non-hemolytic|lcl|cTer
AKRHHGLNCAKGVLA
>lcl|2200|lcl|HAPPENN&HAPPENN-RR90|lcl|subdataset_12|lcl|non-hemolytic|lcl|cTer
RPAFRKAAFRVMRACV
>lcl|4679|lcl|HAPPENN|lcl|subdataset_8|lcl|non-hemolytic|lcl|cTer
IKIKIKIK
>lcl|1065|lcl|HAPPENN&HAPPENN-RR90&HAPPENN-hard|lcl|subdataset_4|lcl|hemolytic
RWCVYAYVRVRGVLVRYRRCW
>lcl|1250|lcl|HAPPENN|lcl|subdataset_4|lcl|hemolytic
KWKSFLKTFKSAVKTVLHTALKAISS
>lcl|3996|lcl|HAPPENN|lcl|subdataset_8|lcl|hemolytic|lcl|cTer
LKKVLKAAA
>lcl|2766|lcl|HAPPENN&HAPPENN-RR90&HAPPENN-hard|lcl|subdataset_12|lcl|non-hemolytic|lcl|cTer
GLPALISWIKRKRGG
>lcl|874|lcl|HAPPENN&HAPPENN-RR90&HAPPENN-hard|lcl|subdataset_4|lcl|non-hemolytic
CLRIGMRGRELMGGIGKTM
>lcl|1187|lcl|HAPPENN&HAPPENN-RR90|lcl|subdataset_7|lcl|non-hemolytic
GIGKFLHSAKKFGKAFVGEIMNSKKKKKKKKK
>lcl|4608|lcl|HAPPENN|lcl|subdataset_12|lcl|non-hemolytic|lcl|cTer
RKKRWWRRKK
>lcl|1075|lcl|HAPPENN&HAPPENN-RR90&HAPPENN-hard|lcl|subdataset_4|lcl|hemolytic|lcl|cTer
GMASKAGSVLGKITKIALGAL
>lcl|3255|lcl|HAPPENN&HAPPENN-RR90&HAPPENN-hard|lcl|subdataset_5|lcl|hemolytic|lcl|nTer
FLPIIAGIAAKFLPKIFCTISKKC
>lcl|1198|lcl|HAPPENN&HAPPENN-RR90&HAPPENN-hard|lcl|subdataset_11|lcl|hemolytic
KLLKLLLKLWLKLLKLLL
>lcl|1283|lcl|HAPPENN&HAPPENN-RR90&HAPPENN-hard|lcl|subdataset_5|lcl|hemolytic
FHPSLWVLIPQYIQLIRKILKSG
>lcl|2722|lcl|HAPPENN&HAPPENN-RR90&HAPPENN-hard|lcl|subdataset_12|lcl|hemolytic
GLKEVLHSTKKFAKGFITGLTGQ
>lcl|146|lcl|HAPPENN|lcl|subdataset_8|lcl|hemolytic
WCYCRRRFCVCVGR
>lcl|2933|lcl|HAPPENN|lcl|subdataset_11|lcl|non-hemolytic
KKLKKFKKLQ
>lcl|3280|lcl|HAPPENN&HAPPENN-RR90&HAPPENN-hard|lcl|subdataset_12|lcl|hemolytic
FLGMIPKLIKKLIKAFK
>lcl|225|lcl|HAPPENN&HAPPENN-hard|lcl|subdataset_12|lcl|non-hemolytic|lcl|cTer
LLKWLLK
>lcl|3099|lcl|HAPPENN&HAPPENN-RR90&HAPPENN-hard|lcl|subdataset_8|lcl|hemolytic|lcl|cTer
GFGMALKLLKKVL
>lcl|1243|lcl|HAPPENN&HAPPENN-RR90&HAPPENN-hard|lcl|subdataset_11|lcl|non-hemolytic|lcl|cTer
GVAKFGKAAAHFGKGWIKEMLNS
>lcl|4779|lcl|HAPPENN&HAPPENN-RR90|lcl|subdataset_4|lcl|non-hemolytic|lcl|cTer
WGRAFSAGVHRLARGGRG
>lcl|2514|lcl|HAPPENN&HAPPENN-RR90|lcl|subdataset_12|lcl|non-hemolytic|lcl|cTer
```

```
KKIGIWIGIKK
>lcl|2928|lcl|HAPPENN|lcl|subdataset_4|lcl|hemolytic
KLLLKFKKLQ
>lcl|2060|lcl|HAPPENN&HAPPENN-RR90&HAPPENN-hard|lcl|subdataset_12|lcl|hemolytic|lcl|cTer
YRMWRWRWRWR
>lcl|3189|lcl|HAPPENN&HAPPENN-RR90|lcl|subdataset_12|lcl|non-hemolytic|lcl|cTer
ILGPVLGLVSDTLDDVLGIL
>lcl|1536|lcl|HAPPENN&HAPPENN-RR90&HAPPENN-hard|lcl|subdataset_10|lcl|hemolytic
FSISASKVLDKFGKIVGKVLKQLKKVSAVAKV
>lcl|4736|lcl|HAPPENN&HAPPENN-RR90&HAPPENN-hard|lcl|subdataset_4|lcl|non-hemolytic
DLWNSIKDMAAAAGRAALNAVTGMVNQ
>lcl|221|lcl|HAPPENN|lcl|subdataset_6|lcl|non-hemolytic
RFGRFLRKIRRFRPK
>lcl|1197|lcl|HAPPENN&HAPPENN-RR90&HAPPENN-hard|lcl|subdataset_11|lcl|hemolytic
KLLKLLLKLWKKLLKLLK
>lcl|1744|lcl|HAPPENN&HAPPENN-RR90&HAPPENN-hard|lcl|subdataset_8|lcl|non-hemolytic|lcl|cTer
ILGAAWNGAKSLF
>lcl|2886|lcl|HAPPENN|lcl|subdataset_4|lcl|non-hemolytic
LKKKLFKLKQ
>lcl|886|lcl|HAPPENN&HAPPENN-RR90|lcl|subdataset_9|lcl|non-hemolytic|lcl|cTer
KWKSFIKKLTSKFLHLAKKF
>lcl|2490|lcl|HAPPENN&HAPPENN-RR90|lcl|subdataset_12|lcl|non-hemolytic|lcl|cTer
WWKAAAKAAAKWW
>lcl|4826|lcl|HAPPENN&HAPPENN-RR90&HAPPENN-hard|lcl|subdataset_12|lcl|hemolytic|lcl|cTer
FRIRVRVAKKFGKAFVGEIM
>lcl|2664|lcl|HAPPENN|lcl|subdataset_9|lcl|non-hemolytic|lcl|cTer
AHCLAIGRK
>lcl|4121|lcl|HAPPENN&HAPPENN-RR90&HAPPENN-hard|lcl|subdataset_7|lcl|non-hemolytic
GWGDTFGKVLKNFAKVAGVKAAK
>lcl|2977|lcl|HAPPENN|lcl|subdataset_4|lcl|hemolytic|lcl|cTer
GLLKRIKILL
>lcl|3131|lcl|HAPPENN&HAPPENN-RR90&HAPPENN-hard|lcl|subdataset_4|lcl|hemolytic
FLPMLAGLAASMVPKLVCLITKKC
>lcl|4746|lcl|HAPPENN|lcl|subdataset_12|lcl|non-hemolytic|lcl|cTer
IKWKRWWWR
>lcl|4048|lcl|HAPPENN&HAPPENN-RR90&HAPPENN-hard|lcl|subdataset_9|lcl|non-hemolytic
GVIPCGESCVFIPCISTLLGCSCKNKVCYRN
>lcl|1277|lcl|HAPPENN|lcl|subdataset_6|lcl|hemolytic|lcl|cTer
VLPKVMAHMK
>lcl|3025|lcl|HAPPENN&HAPPENN-RR90&HAPPENN-hard|lcl|subdataset_5|lcl|hemolytic|lcl|cTer
GMWKKILGHLIR
>lcl|4210|lcl|HAPPENN&HAPPENN-RR90&HAPPENN-hard|lcl|subdataset_4|lcl|hemolytic
FLPMLAGLAANFLPKIICKITKKC
>lcl|1740|lcl|HAPPENN&HAPPENN-RR90&HAPPENN-hard|lcl|subdataset_6|lcl|non-hemolytic|lcl|cTer
ILGKAWEGAKSLF
>lcl|2304|lcl|HAPPENN|lcl|subdataset_12|lcl|non-hemolytic
LQDLALQGAKERAHQQ
>lcl|558|lcl|HAPPENN&HAPPENN-hard|lcl|subdataset_5|lcl|non-hemolytic|lcl|cTer
LLKKLLKK
>lcl|518|lcl|HAPPENN&HAPPENN-RR90&HAPPENN-hard|lcl|subdataset_9|lcl|hemolytic|lcl|cTer
GLLGPLLKIAAKVGSKLL
>lcl|977|lcl|HAPPENN&HAPPENN-RR90&HAPPENN-hard|lcl|subdataset_7|lcl|hemolytic|lcl|cTer
FLGLLFHGVHHVGKWIHGLIHGHH
>lcl|2565|lcl|HAPPENN&HAPPENN-RR90&HAPPENN-hard|lcl|subdataset_12|lcl|hemolytic
FLPLLLAGLPLKLCFLFKKC
>lcl|1091|lcl|HAPPENN&HAPPENN-RR90&HAPPENN-hard|lcl|subdataset_11|lcl|hemolytic
GFLGPLLKLGLKGAAKLLPQLLPSRQQ
>lcl|963|lcl|HAPPENN&HAPPENN-RR90&HAPPENN-hard|lcl|subdataset_4|lcl|hemolytic|lcl|cTer
FLGALIKGAIHGGRFIHGMIQNHH
>lcl|4006|lcl|HAPPENN&HAPPENN-RR90|lcl|subdataset_4|lcl|non-hemolytic
GWFDVVKHIASAV
>lcl|2307|lcl|HAPPENN|lcl|subdataset_7|lcl|non-hemolytic
GAKERAHQ
>lcl|642|lcl|HAPPENN&HAPPENN-hard|lcl|subdataset_5|lcl|non-hemolytic|lcl|cTer
KWKLFKKIPKFLHL
>lcl|3738|lcl|HAPPENN|lcl|subdataset_12|lcl|hemolytic|lcl|nTer|lcl|cTer
RGLRRLGRKIAHGVKKYG
>lcl|3518|lcl|HAPPENN&HAPPENN-RR90|lcl|subdataset_12|lcl|non-hemolytic
```

```
IWDAIFHGAKHFLHRLVNPGGKDAVKDVQQKQ
>lcl|1329|lcl|HAPPENN&HAPPENN-RR90&HAPPENN-hard|lcl|subdataset_5|lcl|hemolytic|lcl|cTer
NLWAGILKHIIK
>lcl|2993|lcl|HAPPENN&HAPPENN-RR90&HAPPENN-hard|lcl|subdataset_4|lcl|hemolytic|lcl|cTer
SLLSLIRKLIT
>lcl|1491|lcl|HAPPENN&HAPPENN-RR90&HAPPENN-hard|lcl|subdataset_6|lcl|hemolytic
TCGETCFGGTCNTPGCTCDPWPICTDRGLP
>lcl|2984|lcl|HAPPENN|lcl|subdataset_7|lcl|hemolytic|lcl|cTer
KLLKFIKTLL
>lcl|126|lcl|HAPPENN&HAPPENN-hard|lcl|subdataset_10|lcl|non-hemolytic
RGGRLCYCRRRFCVC
>lcl|1061|lcl|HAPPENN&HAPPENN-RR90|lcl|subdataset_8|lcl|non-hemolytic|lcl|cTer
LLKWLKKWLKK
>lcl|4261|lcl|HAPPENN&HAPPENN-RR90&HAPPENN-hard|lcl|subdataset_10|lcl|hemolytic
FLPLILPSIVTALSSFLKQG
>lcl|4086|lcl|HAPPENN&HAPPENN-RR90|lcl|subdataset_7|lcl|non-hemolytic|lcl|cTer
GGHRAGLQFPVGRVHRLLRK
>lcl|3029|lcl|HAPPENN&HAPPENN-RR90&HAPPENN-hard|lcl|subdataset_5|lcl|hemolytic|lcl|cTer
GKWSKILGKLIR
>lcl|3605|lcl|HAPPENN|lcl|subdataset_4|lcl|hemolytic|lcl|cTer
FLGLLGSLL
>lcl|1812|lcl|HAPPENN&HAPPENN-hard|lcl|subdataset_8|lcl|non-hemolytic|lcl|cTer
FSEAIKKIIDFLG
>lcl|4549|lcl|HAPPENN|lcl|subdataset_12|lcl|non-hemolytic
SKVWRHWRRFW
>lcl|2857|lcl|HAPPENN&HAPPENN-RR90&HAPPENN-hard|lcl|subdataset_10|lcl|non-hemolytic|lcl|cTer
KLWKLWKKWLK
>lcl|2596|lcl|HAPPENN&HAPPENN-RR90&HAPPENN-hard|lcl|subdataset_6|lcl|non-hemolytic
CVHWQTNTARTSCIGP
>lcl|294|lcl|HAPPENN&HAPPENN-RR90&HAPPENN-hard|lcl|subdataset_8|lcl|hemolytic
PAICQRAEIEACILL
>lcl|4655|lcl|HAPPENN|lcl|subdataset_6|lcl|non-hemolytic|lcl|nTer|lcl|cTer
CRRWWRFC
>lcl|601|lcl|HAPPENN&HAPPENN-RR90&HAPPENN-hard|lcl|subdataset_4|lcl|hemolytic|lcl|cTer
GIWKSLFTKLLKG
>lcl|3553|lcl|HAPPENN&HAPPENN-RR90&HAPPENN-hard|lcl|subdataset_10|lcl|hemolytic
GLLSNVAGLLKQFAKGGVNAVLNPK
>lcl|715|lcl|HAPPENN|lcl|subdataset_5|lcl|non-hemolytic|lcl|cTer
GLPALISLIKRKRQQ
>lcl|3454|lcl|HAPPENN&HAPPENN-hard|lcl|subdataset_8|lcl|non-hemolytic|lcl|cTer
GIGAAILSAGKSALKGLAKGLAEHF
>lcl|2790|lcl|HAPPENN&HAPPENN-RR90|lcl|subdataset_12|lcl|non-hemolytic
FLKALFKALSKLL
>lcl|1637|lcl|HAPPENN&HAPPENN-RR90&HAPPENN-hard|lcl|subdataset_5|lcl|hemolytic
FLPAIIGMAAKVLPAFLCKITKKC
>lcl|4768|lcl|HAPPENN|lcl|subdataset_10|lcl|hemolytic
WWWLKRIW
>lcl|716|lcl|HAPPENN|lcl|subdataset_9|lcl|non-hemolytic|lcl|cTer
GLPALISSIKRKRQQ
>lcl|2589|lcl|HAPPENN&HAPPENN-RR90|lcl|subdataset_10|lcl|non-hemolytic
CLMARPNYRCKIFKQC
>lcl|857|lcl|HAPPENN&HAPPENN-RR90&HAPPENN-hard|lcl|subdataset_11|lcl|hemolytic|lcl|cTer
KWASLWNWFNITNWLWYIK
>lcl|4387|lcl|HAPPENN|lcl|subdataset_7|lcl|non-hemolytic
AGLQFPVGRVHRLLRK
>lcl|1992|lcl|HAPPENN&HAPPENN-RR90&HAPPENN-hard|lcl|subdataset_6|lcl|hemolytic|lcl|cTer
FWGALAKGALKLIPSLVSSFTKKD
>lcl|3833|lcl|HAPPENN&HAPPENN-RR90|lcl|subdataset_6|lcl|non-hemolytic|lcl|cTer
KLAGLAKKLAGLAKKLAGLAK
>lcl|533|lcl|HAPPENN&HAPPENN-RR90&HAPPENN-hard|lcl|subdataset_12|lcl|hemolytic
GLMDVFKGAAKNLLASALDKIRCKVTKC
>lcl|3703|lcl|HAPPENN&HAPPENN-RR90&HAPPENN-hard|lcl|subdataset_6|lcl|non-hemolytic|lcl|nTer|lcl|cTer
ILPWKWKFFPWRR
>lcl|1001|lcl|HAPPENN|lcl|subdataset_10|lcl|hemolytic|lcl|cTer
GIIKKIIKKIIKKI
>lcl|1855|lcl|HAPPENN&HAPPENN-RR90&HAPPENN-hard|lcl|subdataset_7|lcl|hemolytic|lcl|cTe
```

```
r
FLSLIPKIATGIAALAKHL
>lcl|2396|lcl|HAPPENN&HAPPENN-RR90&HAPPENN-hard|lcl|subdataset_5|lcl|hemolytic|lcl|cTer
FLSLIPKAISAVSALANHF
>lcl|2923|lcl|HAPPENN|lcl|subdataset_11|lcl|non-hemolytic
KLKLKFKKLQ
>lcl|3701|lcl|HAPPENN&HAPPENN-RR90|lcl|subdataset_8|lcl|non-hemolytic|lcl|nTer|lcl|cTer
RRRAAFAAWAAFAARRR
>lcl|2317|lcl|HAPPENN&HAPPENN-RR90&HAPPENN-hard|lcl|subdataset_10|lcl|hemolytic|lcl|cTer
FLSLIPHIASGIASLVKNF
>lcl|1076|lcl|HAPPENN&HAPPENN-RR90&HAPPENN-hard|lcl|subdataset_10|lcl|hemolytic
GLGSLLGKAFKFGLKTVGKMMGGAPREQ
>lcl|3071|lcl|HAPPENN&HAPPENN-RR90&HAPPENN-hard|lcl|subdataset_9|lcl|hemolytic|lcl|cTer
VNWKKVLAKIIKVAK
>lcl|2872|lcl|HAPPENN|lcl|subdataset_7|lcl|non-hemolytic
KLKKKFLKLQ
>lcl|3590|lcl|HAPPENN&HAPPENN-RR90&HAPPENN-hard|lcl|subdataset_8|lcl|non-hemolytic|lcl|cTer
RIVQRIKKWLLKWKKLGY
>lcl|3647|lcl|HAPPENN&HAPPENN-RR90&HAPPENN-hard|lcl|subdataset_6|lcl|hemolytic
FLPLIAGVAASILPKIFCFITKKC
>lcl|2089|lcl|HAPPENN&HAPPENN-RR90&HAPPENN-hard|lcl|subdataset_10|lcl|hemolytic|lcl|cTer
ALWKDILKNAGKAALNEINQIVQ
>lcl|4711|lcl|HAPPENN&HAPPENN-RR90&HAPPENN-hard|lcl|subdataset_5|lcl|hemolytic
FIFHIIKGLFHAGKMIHGLVTRRRH
>lcl|3912|lcl|HAPPENN|lcl|subdataset_6|lcl|non-hemolytic
RWKRWWRRKK
>lcl|1435|lcl|HAPPENN|lcl|subdataset_8|lcl|non-hemolytic|lcl|cTer
FKRLEKLFSKIWNWK
>lcl|4088|lcl|HAPPENN&HAPPENN-RR90|lcl|subdataset_4|lcl|non-hemolytic
GLFRALLRLLRSLWRLLLRA
>lcl|4289|lcl|HAPPENN&HAPPENN-RR90&HAPPENN-hard|lcl|subdataset_5|lcl|non-hemolytic
GVFDIIKGAGKQLIAHAMGKIAEKVGLNKDGN
>lcl|1290|lcl|HAPPENN&HAPPENN-RR90&HAPPENN-hard|lcl|subdataset_8|lcl|hemolytic
FLSLALAALPKFLCLVFKKC
>lcl|1570|lcl|HAPPENN|lcl|subdataset_5|lcl|hemolytic|lcl|cTer
ALHKTMLKKLGTMAL
>lcl|595|lcl|HAPPENN&HAPPENN-RR90&HAPPENN-hard|lcl|subdataset_4|lcl|hemolytic
LKIPGFVKDTLKKVAKGIFSAVAGAMTPS
>lcl|2432|lcl|HAPPENN&HAPPENN-hard|lcl|subdataset_5|lcl|non-hemolytic|lcl|cTer
RWKIFKKIEKVG
>lcl|1830|lcl|HAPPENN|lcl|subdataset_12|lcl|hemolytic|lcl|cTer
FLGGLIKWWPWRR
>lcl|1302|lcl|HAPPENN|lcl|subdataset_10|lcl|hemolytic
KFFKFFKFF
>lcl|2658|lcl|HAPPENN|lcl|subdataset_11|lcl|hemolytic
FLPMLAGLAANFLPKIVCKITKKC
>lcl|4658|lcl|HAPPENN&HAPPENN-RR90&HAPPENN-hard|lcl|subdataset_4|lcl|hemolytic|lcl|cTer
FLPAALAGIGGILGKLF
>lcl|42|lcl|HAPPENN&HAPPENN-RR90&HAPPENN-hard|lcl|subdataset_5|lcl|hemolytic
GIMSLFKGVLKTAGKHVAGSLVDQLKCKITGGC
>lcl|4731|lcl|HAPPENN&HAPPENN-RR90&HAPPENN-hard|lcl|subdataset_6|lcl|hemolytic
KWLRRVWRWWR
>lcl|1668|lcl|HAPPENN&HAPPENN-RR90|lcl|subdataset_11|lcl|non-hemolytic|lcl|cTer
KLPKLPWKPKK
>lcl|4422|lcl|HAPPENN|lcl|subdataset_9|lcl|non-hemolytic|lcl|cTer
GKLWLKG
>lcl|3774|lcl|HAPPENN&HAPPENN-RR90|lcl|subdataset_6|lcl|non-hemolytic
KKTWWKTWWTKWKK
>lcl|2298|lcl|HAPPENN&HAPPENN-RR90&HAPPENN-hard|lcl|subdataset_6|lcl|hemolytic|lcl|cTer
GIGKFIHSVKKWGKTFIGEIMNS
>lcl|3667|lcl|HAPPENN&HAPPENN-RR90|lcl|subdataset_5|lcl|non-hemolytic|lcl|cTer
GVVVRIGRVIVRGVRR
>lcl|4233|lcl|HAPPENN&HAPPENN-RR90|lcl|subdataset_8|lcl|non-hemolytic|lcl|cTer
WRWRCRRRFCRWRW
>lcl|4232|lcl|HAPPENN|lcl|subdataset_5|lcl|non-hemolytic|lcl|cTer
```

```
WRCRRRFCRW
>lcl|675|lcl|HAPPENN&HAPPENN-RR90|lcl|subdataset_8|lcl|non-hemolytic|lcl|cTer
RRRRRRAAFAAWAAFAA
>lcl|2989|lcl|HAPPENN&HAPPENN-RR90|lcl|subdataset_12|lcl|non-hemolytic|lcl|cTer
KKIGAIKKWGAIKKIGAI
>lcl|2296|lcl|HAPPENN|lcl|subdataset_7|lcl|non-hemolytic|lcl|cTer
QKALNEINQF
>lcl|3769|lcl|HAPPENN|lcl|subdataset_12|lcl|non-hemolytic|lcl|cTer
RWWRYWR
>lcl|4809|lcl|HAPPENN&HAPPENN-RR90&HAPPENN-hard|lcl|subdataset_12|lcl|hemolytic
KKYRYHLKPFSKK
>lcl|4277|lcl|HAPPENN&HAPPENN-RR90|lcl|subdataset_5|lcl|non-hemolytic
HLNKRVQRELIGWLDWLK
>lcl|3870|lcl|HAPPENN&HAPPENN-RR90&HAPPENN-hard|lcl|subdataset_11|lcl|non-hemolytic|lcl|cTer
LRFAKKALKKLF
>lcl|3693|lcl|HAPPENN&HAPPENN-RR90|lcl|subdataset_11|lcl|non-hemolytic|lcl|nTer|lcl|cTer
GIGKHVGKALKGLKGLLKGLGES
>lcl|684|lcl|HAPPENN&HAPPENN-RR90|lcl|subdataset_11|lcl|non-hemolytic
LRCKKRRWKCRV
>lcl|3569|lcl|HAPPENN&HAPPENN-hard|lcl|subdataset_7|lcl|non-hemolytic|lcl|cTer
IGGYCSWLRL
>lcl|3381|lcl|HAPPENN&HAPPENN-RR90&HAPPENN-hard|lcl|subdataset_11|lcl|hemolytic|lcl|cTer
ILPWKWPWWPWRA
>lcl|7|lcl|HAPPENN|lcl|subdataset_4|lcl|hemolytic
ALWKTLLKKVLKAAAKAALN
>lcl|900|lcl|HAPPENN&HAPPENN-RR90|lcl|subdataset_5|lcl|non-hemolytic|lcl|cTer
KWKSFIKKLTSKFLHSAKKN
>lcl|683|lcl|HAPPENN&HAPPENN-RR90|lcl|subdataset_11|lcl|non-hemolytic
LCLKKRRWKYCV
>lcl|4235|lcl|HAPPENN&HAPPENN-RR90|lcl|subdataset_10|lcl|non-hemolytic|lcl|cTer
PRPRCRRRFCRPRP
>lcl|3959|lcl|HAPPENN&HAPPENN-RR90&HAPPENN-hard|lcl|subdataset_4|lcl|non-hemolytic|lcl|cTer
FLPLIGKVLSGIL
>lcl|4338|lcl|HAPPENN&HAPPENN-RR90&HAPPENN-hard|lcl|subdataset_9|lcl|non-hemolytic|lcl|cTer
GIHDILKYGKPA
>lcl|2771|lcl|HAPPENN|lcl|subdataset_4|lcl|hemolytic
GLPICGETCVGGTCNTPGCSCSWPVCTRN
>lcl|2453|lcl|HAPPENN|lcl|subdataset_12|lcl|non-hemolytic
RWRRFWRR
>lcl|4560|lcl|HAPPENN&HAPPENN-RR90|lcl|subdataset_5|lcl|non-hemolytic
YPCKLNLKLGKVPFH
>lcl|1190|lcl|HAPPENN&HAPPENN-RR90|lcl|subdataset_5|lcl|non-hemolytic
KKKKKKKKKKGIGKLFLHAAKKFAKAFVAEKMNS
>lcl|3429|lcl|HAPPENN|lcl|subdataset_10|lcl|non-hemolytic|lcl|cTer
KISKKIMRTFLRR
>lcl|3373|lcl|HAPPENN&HAPPENN-RR90&HAPPENN-hard|lcl|subdataset_9|lcl|hemolytic|lcl|cTer
ILPAKWPWWPWRR
>lcl|3896|lcl|HAPPENN&HAPPENN-RR90|lcl|subdataset_12|lcl|non-hemolytic
DKPKKKPPPPAGPPPPPPPPPGPPPPGP
>lcl|73|lcl|HAPPENN&HAPPENN-RR90&HAPPENN-hard|lcl|subdataset_10|lcl|hemolytic|lcl|cTer
KILRGVCKKIMRTFLRRISKDILTGKK
>lcl|2756|lcl|HAPPENN&HAPPENN-RR90&HAPPENN-hard|lcl|subdataset_10|lcl|hemolytic
SMLKVLKNLGKVGLGFVACKINKQC
>lcl|2564|lcl|HAPPENN&HAPPENN-RR90&HAPPENN-hard|lcl|subdataset_11|lcl|hemolytic
VWPLGLVICKALKIC
>lcl|3699|lcl|HAPPENN&HAPPENN-RR90&HAPPENN-hard|lcl|subdataset_5|lcl|non-hemolytic|lcl|nTer|lcl|cTer
SWLSKTAKKLENSAKKRISEGIAIAIQGGPR
>lcl|2894|lcl|HAPPENN|lcl|subdataset_12|lcl|non-hemolytic
PQPRPPHPRL
>lcl|4114|lcl|HAPPENN&HAPPENN-RR90|lcl|subdataset_7|lcl|non-hemolytic|lcl|cTer
RFRRLRIKTRIRLKKI
>lcl|3780|lcl|HAPPENN|lcl|subdataset_9|lcl|non-hemolytic|lcl|cTer
GIKDILKYGKPS
>lcl|256|lcl|HAPPENN&HAPPENN-RR90&HAPPENN-hard|lcl|subdataset_6|lcl|hemolytic|lcl|cTer
ILPWKWPLWPWRR
>lcl|337|lcl|HAPPENN&HAPPENN-RR90&HAPPENN-hard|lcl|subdataset_11|lcl|hemolytic
```

```
GILSSFKGVAKGVAKNLAGKLLDELKCKITGC
>lcl|36|lcl|HAPPENN&HAPPENN-RR90&HAPPENN-hard|lcl|subdataset_5|lcl|non-hemolytic|lcl|cTer
RGLRRLGRKIAHGVKKYGPTVKRIKRKA
>lcl|297|lcl|HAPPENN&HAPPENN-hard|lcl|subdataset_9|lcl|non-hemolytic
MSVSDAVQLMLGFGTFVLMLLGLVVELIKNSNKK
>lcl|2293|lcl|HAPPENN|lcl|subdataset_5|lcl|non-hemolytic|lcl|cTer
SVAGRAQGM
>lcl|3154|lcl|HAPPENN&HAPPENN-RR90&HAPPENN-hard|lcl|subdataset_12|lcl|non-hemolytic|lcl|cTer
FLPFLIPALTSLISSL
>lcl|3918|lcl|HAPPENN&HAPPENN-RR90&HAPPENN-hard|lcl|subdataset_7|lcl|non-hemolytic|lcl|cTer
FKVTWKTKWWKG
>lcl|401|lcl|HAPPENN&HAPPENN-RR90&HAPPENN-hard|lcl|subdataset_4|lcl|non-hemolytic|lcl|cTer
VRRFAWWWPFLRR
>lcl|4482|lcl|HAPPENN&HAPPENN-RR90&HAPPENN-hard|lcl|subdataset_5|lcl|non-hemolytic|lcl|cTer
GILSSLWKKPKKIIAK
>lcl|3791|lcl|HAPPENN&HAPPENN-RR90|lcl|subdataset_4|lcl|non-hemolytic|lcl|cTer
GIRRILRYGRPS
>lcl|351|lcl|HAPPENN|lcl|subdataset_9|lcl|hemolytic
STIGDLVKWIIDTVNKFTKK
>lcl|1106|lcl|HAPPENN&HAPPENN-RR90&HAPPENN-hard|lcl|subdataset_9|lcl|hemolytic|lcl|cTer
ILGKLLSTAAGLLSKL
>lcl|914|lcl|HAPPENN&HAPPENN-RR90&HAPPENN-hard|lcl|subdataset_12|lcl|hemolytic|lcl|cTer
VNRKKILGKSIKVVK
>lcl|1539|lcl|HAPPENN|lcl|subdataset_6|lcl|non-hemolytic|lcl|cTer
MLKKFRGMF
>lcl|65|lcl|HAPPENN|lcl|subdataset_7|lcl|hemolytic
FFHHIFRAIVHVGKTIHRLVTG
>lcl|602|lcl|HAPPENN&HAPPENN-RR90&HAPPENN-hard|lcl|subdataset_12|lcl|non-hemolytic
GWGSFFKKAAHVGKHVGKAALTHYL
>lcl|723|lcl|HAPPENN&HAPPENN-hard|lcl|subdataset_7|lcl|non-hemolytic
SIGSALKKALPVAKKIGKIALPIAKAALP
>lcl|3623|lcl|HAPPENN&HAPPENN-RR90|lcl|subdataset_6|lcl|non-hemolytic|lcl|cTer
GRFKRFRKKAKKLFKKLSPVIPLLHL
>lcl|846|lcl|HAPPENN|lcl|subdataset_7|lcl|non-hemolytic|lcl|cTer
KWKWKWKW
>lcl|3044|lcl|HAPPENN&HAPPENN-RR90&HAPPENN-hard|lcl|subdataset_4|lcl|hemolytic|lcl|cTer
GKWMSLLKHWLK
>lcl|3818|lcl|HAPPENN&HAPPENN-RR90&HAPPENN-hard|lcl|subdataset_4|lcl|non-hemolytic|lcl|cTer
LLIRAAKKFIKK
>lcl|2071|lcl|HAPPENN&HAPPENN-RR90|lcl|subdataset_8|lcl|non-hemolytic
FFGRLKAVFRGARQGWKEHRY
>lcl|4047|lcl|HAPPENN|lcl|subdataset_11|lcl|non-hemolytic
RWRLLLLKKH
>lcl|1502|lcl|HAPPENN&HAPPENN-RR90&HAPPENN-hard|lcl|subdataset_5|lcl|non-hemolytic
FLGPIIKIATGILPTAICKILKKC
>lcl|2800|lcl|HAPPENN|lcl|subdataset_4|lcl|non-hemolytic
SIGRRGGYCAGIIKQTCTCYR
>lcl|700|lcl|HAPPENN&HAPPENN-RR90&HAPPENN-hard|lcl|subdataset_12|lcl|hemolytic
GIMDSVKGLAKNLAGKLLDSLKCKITGC
>lcl|1635|lcl|HAPPENN&HAPPENN-RR90&HAPPENN-hard|lcl|subdataset_11|lcl|hemolytic|lcl|cTer
FLPLLAGLAAKWF
>lcl|4433|lcl|HAPPENN&HAPPENN-RR90|lcl|subdataset_10|lcl|non-hemolytic
WKKILSKIKKLLK
>lcl|3072|lcl|HAPPENN|lcl|subdataset_12|lcl|hemolytic|lcl|cTer
VNWKKVLKKIIKVAK
>lcl|3338|lcl|HAPPENN|lcl|subdataset_10|lcl|non-hemolytic|lcl|cTer
EQHSSGKSDV
>lcl|1404|lcl|HAPPENN&HAPPENN-RR90&HAPPENN-hard|lcl|subdataset_7|lcl|hemolytic|lcl|cTer
INWSSIFESVSNLV
>lcl|3910|lcl|HAPPENN&HAPPENN-RR90|lcl|subdataset_12|lcl|non-hemolytic
RVVRQWPIGRVVRRVVRRVVRY
>lcl|4310|lcl|HAPPENN|lcl|subdataset_12|lcl|hemolytic
```

```
GIGKFLHSAKKFAKAFVGEIMNS
>lcl|3596|lcl|HAPPENN|lcl|subdataset_9|lcl|non-hemolytic
LVQRGRFGRFLRKIR
>lcl|3932|lcl|HAPPENN&HAPPENN-RR90|lcl|subdataset_8|lcl|non-hemolytic|lcl|cTer
KLLLKLKLKLLKGWKRKRFG
>lcl|71|lcl|HAPPENN|lcl|subdataset_11|lcl|hemolytic
FFHHIFRAIVHVPKTIHRLVTG
>lcl|1878|lcl|HAPPENN|lcl|subdataset_10|lcl|non-hemolytic
RVMFKWA
>lcl|3508|lcl|HAPPENN&HAPPENN-RR90&HAPPENN-hard|lcl|subdataset_7|lcl|non-hemolytic
INLKAIAPLAKKLL
>lcl|3331|lcl|HAPPENN&HAPPENN-RR90&HAPPENN-hard|lcl|subdataset_9|lcl|hemolytic|lcl|cTer
FVDLKKIANIINSIFGK
>lcl|4328|lcl|HAPPENN|lcl|subdataset_12|lcl|non-hemolytic|lcl|cTer
GIADILKYGKPS
>lcl|692|lcl|HAPPENN|lcl|subdataset_9|lcl|non-hemolytic
KKVADKVLLLKQLRIMRL
>lcl|1496|lcl|HAPPENN&HAPPENN-RR90&HAPPENN-hard|lcl|subdataset_10|lcl|non-hemolytic
CVISAGWNHKIRCKLTGNC
>lcl|3051|lcl|HAPPENN&HAPPENN-RR90&HAPPENN-hard|lcl|subdataset_9|lcl|hemolytic|lcl|cTer
GKWMSFLKHILK
>lcl|1161|lcl|HAPPENN&HAPPENN-RR90&HAPPENN-hard|lcl|subdataset_11|lcl|hemolytic|lcl|cTer
FIGSALKVLAGVLPSVISWVKQ
>lcl|417|lcl|HAPPENN&HAPPENN-RR90&HAPPENN-hard|lcl|subdataset_7|lcl|non-hemolytic|lcl|cTer
HFKGTLVNLAKKIL
>lcl|1274|lcl|HAPPENN|lcl|subdataset_5|lcl|hemolytic|lcl|cTer
KVMAHMK
>lcl|4390|lcl|HAPPENN&HAPPENN-RR90&HAPPENN-hard|lcl|subdataset_12|lcl|non-hemolytic
GEGFLGMLLHGVGHAIHGLIHGK
>lcl|1988|lcl|HAPPENN&HAPPENN-RR90&HAPPENN-hard|lcl|subdataset_10|lcl|non-hemolytic
GFLEKLKKGAKDFASALVNSIKGT
>lcl|2829|lcl|HAPPENN&HAPPENN-RR90&HAPPENN-hard|lcl|subdataset_7|lcl|hemolytic|lcl|cTer
YKLLKLLLPKLKPLLIKL
>lcl|1109|lcl|HAPPENN&HAPPENN-RR90&HAPPENN-hard|lcl|subdataset_12|lcl|hemolytic|lcl|cTer
ILGKLLSTWAGLLSNL
>lcl|1915|lcl|HAPPENN&HAPPENN-RR90|lcl|subdataset_9|lcl|non-hemolytic
PCNPDHDYRPFGNFRIAFTT
>lcl|2625|lcl|HAPPENN|lcl|subdataset_5|lcl|hemolytic|lcl|cTer
GIGAVLEVLTTGLPALISWIKRKRQQ
>lcl|3744|lcl|HAPPENN|lcl|subdataset_10|lcl|hemolytic
RPPQFTRAQWFAIQHISLN
>lcl|1718|lcl|HAPPENN&HAPPENN-RR90|lcl|subdataset_10|lcl|non-hemolytic
RWCVRARVRGVRYRRCW
>lcl|4563|lcl|HAPPENN&HAPPENN-RR90|lcl|subdataset_4|lcl|non-hemolytic
NYISFFRKCKNSQST
>lcl|3835|lcl|HAPPENN&HAPPENN-RR90&HAPPENN-hard|lcl|subdataset_5|lcl|hemolytic
GLNALKKVFKGIHEAIKLINNHVQ
>lcl|427|lcl|HAPPENN|lcl|subdataset_8|lcl|hemolytic|lcl|cTer
HFLLTLVNLAKKIL
>lcl|1229|lcl|HAPPENN&HAPPENN-RR90&HAPPENN-hard|lcl|subdataset_5|lcl|hemolytic|lcl|cTer
FLRFIGSVIHGIGHLVHHIGVAL
>lcl|2232|lcl|HAPPENN&HAPPENN-RR90&HAPPENN-hard|lcl|subdataset_11|lcl|non-hemolytic|lcl|cTer
LLGPVLGLVSNVLGGLL
>lcl|3785|lcl|HAPPENN&HAPPENN-RR90|lcl|subdataset_11|lcl|non-hemolytic|lcl|cTer
GIHDILKYGKRS
>lcl|657|lcl|HAPPENN&HAPPENN-RR90&HAPPENN-hard|lcl|subdataset_9|lcl|hemolytic
VRRFGWWWGFLRR
>lcl|2155|lcl|HAPPENN|lcl|subdataset_4|lcl|non-hemolytic|lcl|cTer
FLKLLKKAL
>lcl|2502|lcl|HAPPENN&HAPPENN-RR90&HAPPENN-hard|lcl|subdataset_8|lcl|non-hemolytic|lcl|cTer
ANWKGIAAMAKKLL
>lcl|1008|lcl|HAPPENN&HAPPENN-RR90|lcl|subdataset_7|lcl|non-hemolytic
GKPRPYSPRPTSHPRPIRV
>lcl|1576|lcl|HAPPENN|lcl|subdataset_6|lcl|hemolytic
```

```
SMLSVLKNLGKVGLGFVACKINKQC
>lcl|3463|lcl|HAPPENN&HAPPENN-RR90&HAPPENN-hard|lcl|subdataset_7|lcl|non-hemolytic|lcl|cTer
KRFKKFFKKVKKSVKKRLKKIFKKPMVIGVTIPF
>lcl|1342|lcl|HAPPENN&HAPPENN-RR90&HAPPENN-hard|lcl|subdataset_11|lcl|hemolytic|lcl|cTer
LNWGAVLKHVVK
>lcl|1238|lcl|HAPPENN&HAPPENN-RR90&HAPPENN-hard|lcl|subdataset_12|lcl|hemolytic|lcl|cTer
GIGAVLKVLTTGLKWKLFKKIEKVGQ
>lcl|4770|lcl|HAPPENN|lcl|subdataset_11|lcl|hemolytic
WWWLKKIW
>lcl|2945|lcl|HAPPENN&HAPPENN-hard|lcl|subdataset_5|lcl|non-hemolytic
GILDTLKQFAKGVGKDLVKGAAQGVLST
>lcl|4732|lcl|HAPPENN&HAPPENN-RR90&HAPPENN-hard|lcl|subdataset_7|lcl|hemolytic
GIFPIFAKLLGKVIKVASSLISKGRTK
>lcl|2322|lcl|HAPPENN&HAPPENN-RR90&HAPPENN-hard|lcl|subdataset_9|lcl|non-hemolytic
IETFLKQLRSAANKIVGL
>lcl|3451|lcl|HAPPENN&HAPPENN-RR90&HAPPENN-hard|lcl|subdataset_6|lcl|non-hemolytic|lcl|cTer
GIGSAILSAGKSALKGLAKGLAEHFAN
>lcl|2946|lcl|HAPPENN&HAPPENN-hard|lcl|subdataset_11|lcl|non-hemolytic
GILDTLKQFAKGVGKDLVKGAAQGVLS
>lcl|545|lcl|HAPPENN&HAPPENN-RR90&HAPPENN-hard|lcl|subdataset_10|lcl|hemolytic|lcl|cTer
LKLASIVSWAKKVL
>lcl|593|lcl|HAPPENN&HAPPENN-RR90&HAPPENN-hard|lcl|subdataset_4|lcl|hemolytic
FLFSLIPKAIGGLISAFK
>lcl|1012|lcl|HAPPENN&HAPPENN-RR90&HAPPENN-hard|lcl|subdataset_7|lcl|non-hemolytic|lcl|cTer
KLAKKLAKLAKLAKAL
>lcl|3426|lcl|HAPPENN&HAPPENN-RR90|lcl|subdataset_11|lcl|non-hemolytic|lcl|cTer
KILRGVSKKIMRRILTGKK
>lcl|1649|lcl|HAPPENN&HAPPENN-RR90|lcl|subdataset_5|lcl|non-hemolytic
CQWQFISPSRAGCIGP
>lcl|3965|lcl|HAPPENN|lcl|subdataset_8|lcl|non-hemolytic|lcl|cTer
GVVDILKGAAKDIAGHLASKVMNKA
>lcl|3369|lcl|HAPPENN|lcl|subdataset_4|lcl|hemolytic|lcl|cTer
WPWWPWRR
>lcl|551|lcl|HAPPENN&HAPPENN-RR90|lcl|subdataset_6|lcl|non-hemolytic|lcl|cTer
DSHAKRHHGYKRKFHEKHHSHRGY
>lcl|726|lcl|HAPPENN&HAPPENN-RR90|lcl|subdataset_8|lcl|non-hemolytic|lcl|nTer|lcl|cTer
CVKVRVKVGSGVKVRVKVC
>lcl|1552|lcl|HAPPENN&HAPPENN-RR90&HAPPENN-hard|lcl|subdataset_4|lcl|hemolytic|lcl|cTer
GLFDIWKWWRWRR
>lcl|4324|lcl|HAPPENN|lcl|subdataset_11|lcl|hemolytic|lcl|cTer
FLGLLGGLL
>lcl|3471|lcl|HAPPENN&HAPPENN-RR90&HAPPENN-hard|lcl|subdataset_12|lcl|hemolytic|lcl|cTer
KLSPSLGPVSKGKLLAGQR
>lcl|2683|lcl|HAPPENN&HAPPENN-RR90|lcl|subdataset_4|lcl|non-hemolytic|lcl|cTer
KILRGVSKKIMRRISKDILTGKK
>lcl|809|lcl|HAPPENN&HAPPENN-RR90|lcl|subdataset_7|lcl|non-hemolytic
GFCRCLCRRGVCRCLCTK
>lcl|3606|lcl|HAPPENN|lcl|subdataset_12|lcl|hemolytic|lcl|cTer
FLKLLKKLL
>lcl|438|lcl|HAPPENN|lcl|subdataset_8|lcl|non-hemolytic
FKRLEKLFSKIWNWK
>lcl|4036|lcl|HAPPENN&HAPPENN-RR90&HAPPENN-hard|lcl|subdataset_9|lcl|non-hemolytic
GIPCGESCVWIPCISAALGCSCKNKVCYRN
>lcl|3299|lcl|HAPPENN&HAPPENN-hard|lcl|subdataset_4|lcl|non-hemolytic
GLGKDLAKLGVDLVACKISKQC
>lcl|2316|lcl|HAPPENN&HAPPENN-RR90&HAPPENN-hard|lcl|subdataset_12|lcl|hemolytic|lcl|cTer
FLSLLPHIASGIASLVSKF
>lcl|2165|lcl|HAPPENN&HAPPENN-hard|lcl|subdataset_5|lcl|non-hemolytic|lcl|cTer
KWKVFKKIEKMIRNIRNKIVK
>lcl|2469|lcl|HAPPENN&HAPPENN-RR90|lcl|subdataset_11|lcl|non-hemolytic
PMARNKKLLKKLRLKIAFK
>lcl|1589|lcl|HAPPENN&HAPPENN-RR90|lcl|subdataset_11|lcl|non-hemolytic|lcl|cTer
KIAKKIAKIAKKIA
>lcl|3014|lcl|HAPPENN&HAPPENN-RR90&HAPPENN-hard|lcl|subdataset_5|lcl|non-hemolytic|lcl
```

```
|cTer
KLAGLKAKWAGLKAKLAGLKA
>lcl|3093|lcl|HAPPENN&HAPPENN-RR90&HAPPENN-hard|lcl|subdataset_12|lcl|hemolytic|lcl|cTer
KNWKKILKKIIKVVK
>lcl|4620|lcl|HAPPENN|lcl|subdataset_4|lcl|non-hemolytic|lcl|cTer
GRRPRPRPRP
>lcl|1398|lcl|HAPPENN&HAPPENN-RR90&HAPPENN-hard|lcl|subdataset_12|lcl|hemolytic|lcl|cTer
INWKKIFEKVKDLV
>lcl|3581|lcl|HAPPENN&HAPPENN-RR90&HAPPENN-hard|lcl|subdataset_4|lcl|non-hemolytic
GFLDIIKDTGKEFAVKILNNLKCKLAGGCPP
>lcl|4249|lcl|HAPPENN|lcl|subdataset_12|lcl|non-hemolytic|lcl|cTer
IGKFLKKAKKFG
>lcl|15|lcl|HAPPENN|lcl|subdataset_5|lcl|hemolytic|lcl|cTer
ALWKTLLKKVLKAAAK
>lcl|1598|lcl|HAPPENN|lcl|subdataset_6|lcl|non-hemolytic|lcl|cTer
KIAGKIAKIAGKIAK
>lcl|1108|lcl|HAPPENN&HAPPENN-RR90&HAPPENN-hard|lcl|subdataset_8|lcl|hemolytic|lcl|cTer
ILGKLLSWAAGLLSNL
>lcl|1077|lcl|HAPPENN|lcl|subdataset_10|lcl|hemolytic
GLGSLLGKAFKFGLKTVGKMMGGAPREE
>lcl|1433|lcl|HAPPENN&HAPPENN-RR90&HAPPENN-hard|lcl|subdataset_9|lcl|hemolytic|lcl|cTer
KNWKKIASIGKEVLKAL
>lcl|2821|lcl|HAPPENN|lcl|subdataset_8|lcl|non-hemolytic|lcl|cTer
ALLLAIRRR
>lcl|1039|lcl|HAPPENN|lcl|subdataset_5|lcl|hemolytic|lcl|cTer
KRAKKFFKKPK
>lcl|3760|lcl|HAPPENN&HAPPENN-RR90&HAPPENN-hard|lcl|subdataset_11|lcl|non-hemolytic
FLGALFKFASK
>lcl|1697|lcl|HAPPENN|lcl|subdataset_4|lcl|non-hemolytic|lcl|cTer
KKLFKKILKDL
>lcl|101|lcl|HAPPENN&HAPPENN-RR90&HAPPENN-hard|lcl|subdataset_9|lcl|non-hemolytic|lcl|cTer
KKKKKKALFALWLAFLA
>lcl|838|lcl|HAPPENN&HAPPENN-hard|lcl|subdataset_7|lcl|non-hemolytic|lcl|cTer
LKWLKKLLKKL
>lcl|2205|lcl|HAPPENN&HAPPENN-RR90|lcl|subdataset_10|lcl|non-hemolytic|lcl|cTer
KLVKTWLKVLK
>lcl|2241|lcl|HAPPENN&HAPPENN-RR90|lcl|subdataset_12|lcl|non-hemolytic|lcl|cTer
KRFIKWYKAWNKKRRVY
>lcl|1102|lcl|HAPPENN&HAPPENN-RR90&HAPPENN-hard|lcl|subdataset_10|lcl|hemolytic|lcl|cTer
ILKKLLSTAAGLLSNL
>lcl|3747|lcl|HAPPENN&HAPPENN-RR90&HAPPENN-hard|lcl|subdataset_5|lcl|non-hemolytic
FLGALFKWASK
>lcl|2715|lcl|HAPPENN|lcl|subdataset_6|lcl|hemolytic
GIGAVLTTGLPALISWIKRKRQQ
>lcl|88|lcl|HAPPENN&HAPPENN-RR90&HAPPENN-hard|lcl|subdataset_11|lcl|non-hemolytic|lcl|nTer|lcl|cTer
KWKEFLKTFKEAKKEVLHTALKAISS
>lcl|2192|lcl|HAPPENN&HAPPENN-RR90|lcl|subdataset_6|lcl|non-hemolytic
KWKKFKKIGAVLKKL
>lcl|1314|lcl|HAPPENN&HAPPENN-RR90&HAPPENN-hard|lcl|subdataset_10|lcl|non-hemolytic
SLFSLIKAGAKFLGKNLLKQGACYAACKASKQC
>lcl|3821|lcl|HAPPENN&HAPPENN-RR90&HAPPENN-hard|lcl|subdataset_4|lcl|hemolytic|lcl|cTer
LRFLKKILKHLF
>lcl|1162|lcl|HAPPENN|lcl|subdataset_7|lcl|hemolytic|lcl|cTer
LALILRKIVTAL
>lcl|3737|lcl|HAPPENN|lcl|subdataset_10|lcl|hemolytic|lcl|nTer|lcl|cTer
RGLRRLGRKIAHGVKKY
>lcl|824|lcl|HAPPENN|lcl|subdataset_11|lcl|non-hemolytic
WKSDVRRWRSRY
>lcl|2772|lcl|HAPPENN&HAPPENN-RR90&HAPPENN-hard|lcl|subdataset_6|lcl|hemolytic
GIPCGESCVWIPCITSAIGCSCKSKVCYRN
>lcl|2221|lcl|HAPPENN&HAPPENN-RR90|lcl|subdataset_10|lcl|non-hemolytic|lcl|cTer
DEMKLDGFNMHLE
>lcl|1698|lcl|HAPPENN|lcl|subdataset_5|lcl|non-hemolytic|lcl|cTer
KKLFKKILKRL
>lcl|3019|lcl|HAPPENN|lcl|subdataset_12|lcl|hemolytic|lcl|cTer
```

```
GMWSKILGHLI
>lcl|4342|lcl|HAPPENN&HAPPENN-RR90&HAPPENN-hard|lcl|subdataset_4|lcl|non-hemolytic|lcl|cTer
WGIHRILKYGKPS
>lcl|1825|lcl|HAPPENN&HAPPENN-RR90&HAPPENN-hard|lcl|subdataset_11|lcl|hemolytic
RIRWILRYWRWS
>lcl|2018|lcl|HAPPENN&HAPPENN-RR90|lcl|subdataset_9|lcl|non-hemolytic|lcl|cTer
FFGHIWHGARTLFRDVFA
>lcl|1594|lcl|HAPPENN|lcl|subdataset_11|lcl|hemolytic|lcl|cTer
KIAGKIASIAGKIAKIAGSIAKIAGK
>lcl|503|lcl|HAPPENN|lcl|subdataset_4|lcl|hemolytic|lcl|cTer
GIGKFLKKAKKFGKAFVKMKK
>lcl|1138|lcl|HAPPENN&HAPPENN-RR90|lcl|subdataset_8|lcl|non-hemolytic
RAGLQFPVGRLLRRLLRRLLR
>lcl|3083|lcl|HAPPENN&HAPPENN-RR90&HAPPENN-hard|lcl|subdataset_6|lcl|hemolytic|lcl|cTer
VNWKKILPKIIKVVK
>lcl|2093|lcl|HAPPENN&HAPPENN-RR90&HAPPENN-hard|lcl|subdataset_11|lcl|hemolytic|lcl|cTer
KITLKLAIKAWKLALKAA
>lcl|773|lcl|HAPPENN|lcl|subdataset_7|lcl|non-hemolytic
RIIDLLWRVRRPQKPKFVTVWVR
>lcl|676|lcl|HAPPENN&HAPPENN-RR90|lcl|subdataset_5|lcl|non-hemolytic|lcl|cTer
KKKKKKAAAAFWAAAAF
>lcl|39|lcl|HAPPENN&HAPPENN-RR90&HAPPENN-hard|lcl|subdataset_7|lcl|non-hemolytic|lcl|cTer
RKLRRLKRKIAHKVKKY
>lcl|2783|lcl|HAPPENN&HAPPENN-RR90&HAPPENN-hard|lcl|subdataset_10|lcl|non-hemolytic
FLGALFKALSKLV
>lcl|4739|lcl|HAPPENN|lcl|subdataset_12|lcl|non-hemolytic|lcl|cTer
RWKRWWRWI
>lcl|826|lcl|HAPPENN|lcl|subdataset_4|lcl|non-hemolytic
WKSYVRRWRSR
>lcl|3753|lcl|HAPPENN&HAPPENN-RR90&HAPPENN-hard|lcl|subdataset_4|lcl|hemolytic|lcl|cTer
KALWKTLLKKVLKA
>lcl|3076|lcl|HAPPENN|lcl|subdataset_5|lcl|hemolytic|lcl|cTer
VNWKKILAKIIKVAK
>lcl|4719|lcl|HAPPENN&HAPPENN-RR90&HAPPENN-hard|lcl|subdataset_12|lcl|non-hemolytic|lcl|cTer
FPVTWPTKWRKG
>lcl|2471|lcl|HAPPENN&HAPPENN-RR90&HAPPENN-hard|lcl|subdataset_10|lcl|non-hemolytic|lcl|nTer|lcl|cTer
KFLKLKWLKSWKNFK
>lcl|1079|lcl|HAPPENN|lcl|subdataset_5|lcl|non-hemolytic
LGRVDIHVWDGVYI
>lcl|2579|lcl|HAPPENN|lcl|subdataset_4|lcl|non-hemolytic
DEKGPKWKR
>lcl|4166|lcl|HAPPENN|lcl|subdataset_5|lcl|hemolytic
SKKLFKKILKYLAGPAKKLFKKILKYLKDEL
>lcl|4177|lcl|HAPPENN&HAPPENN-RR90&HAPPENN-hard|lcl|subdataset_4|lcl|hemolytic
KKLFKKILKYLTTGLPALISWKDEL
>lcl|3281|lcl|HAPPENN&HAPPENN-RR90|lcl|subdataset_7|lcl|non-hemolytic|lcl|cTer
RFRLPFRRPPIRIHPPPFYPPFRRFL
>lcl|2920|lcl|HAPPENN|lcl|subdataset_9|lcl|non-hemolytic
LLKLKFKKKQ
>lcl|3496|lcl|HAPPENN|lcl|subdataset_8|lcl|hemolytic
ALWMTLLKKVLKAAAKALNAVLVGANA
>lcl|3418|lcl|HAPPENN&HAPPENN-RR90&HAPPENN-hard|lcl|subdataset_4|lcl|non-hemolytic
GKLFKKILKIL
>lcl|1603|lcl|HAPPENN|lcl|subdataset_4|lcl|hemolytic|lcl|cTer
KIAGKIAKIAGKIAKIAGKIAKIAGK
>lcl|469|lcl|HAPPENN&HAPPENN-RR90|lcl|subdataset_8|lcl|non-hemolytic|lcl|nTer|lcl|cTer
LKDVEEAQQKIINIIRRL
>lcl|3045|lcl|HAPPENN&HAPPENN-RR90&HAPPENN-hard|lcl|subdataset_5|lcl|hemolytic|lcl|cTer
GKWMSLWKHILK
>lcl|3400|lcl|HAPPENN&HAPPENN-RR90&HAPPENN-hard|lcl|subdataset_9|lcl|non-hemolytic|lcl|cTer
ILKKWPWWPWRRK
>lcl|1685|lcl|HAPPENN&HAPPENN-RR90&HAPPENN-hard|lcl|subdataset_4|lcl|non-hemolytic
GLNALKKVFQPIHEAIKKINNHVQ
>lcl|1121|lcl|HAPPENN|lcl|subdataset_12|lcl|non-hemolytic
```

```
RRLMAAKAES
>lcl|2461|lcl|HAPPENN&HAPPENN-RR90&HAPPENN-hard|lcl|subdataset_12|lcl|hemolytic|lcl|cTer
INWLKIAAAVAGML
>lcl|742|lcl|HAPPENN&HAPPENN-RR90&HAPPENN-hard|lcl|subdataset_9|lcl|non-hemolytic|lcl|cTer
ILGKILKGIKKLF
>lcl|4630|lcl|HAPPENN&HAPPENN-RR90|lcl|subdataset_11|lcl|non-hemolytic
RRPRPRPRPFFFFF
>lcl|1355|lcl|HAPPENN|lcl|subdataset_12|lcl|non-hemolytic
INLKALAALAKKIL
>lcl|445|lcl|HAPPENN&HAPPENN-RR90&HAPPENN-hard|lcl|subdataset_12|lcl|hemolytic|lcl|cTer
LKLFKKILKFL
>lcl|2363|lcl|HAPPENN&HAPPENN-RR90&HAPPENN-hard|lcl|subdataset_11|lcl|hemolytic|lcl|cTer
RRIVQRIRDFLR
>lcl|1041|lcl|HAPPENN|lcl|subdataset_6|lcl|hemolytic|lcl|cTer
KRFKKFFKKPK
>lcl|2423|lcl|HAPPENN&HAPPENN-RR90&HAPPENN-hard|lcl|subdataset_7|lcl|non-hemolytic
GIGAVLKWLPALIKRKRQQ
>lcl|3735|lcl|HAPPENN|lcl|subdataset_6|lcl|hemolytic|lcl|nTer|lcl|cTer
KKTWWKTWWTKWSQPKKKRKV
>lcl|2069|lcl|HAPPENN&HAPPENN-RR90&HAPPENN-hard|lcl|subdataset_5|lcl|hemolytic|lcl|cTer
RKKRRQRRRLNLKALLAVAKKIL
>lcl|1639|lcl|HAPPENN&HAPPENN-RR90&HAPPENN-hard|lcl|subdataset_8|lcl|non-hemolytic
GAPKGCWTKSYPPQPCFGKK
>lcl|2747|lcl|HAPPENN&HAPPENN-RR90&HAPPENN-hard|lcl|subdataset_4|lcl|hemolytic
SMLSVLKNLGKVGLGFVASKINKQS
>lcl|2893|lcl|HAPPENN|lcl|subdataset_7|lcl|non-hemolytic
YIPQPRPPHPRL
>lcl|3427|lcl|HAPPENN&HAPPENN-RR90|lcl|subdataset_8|lcl|non-hemolytic|lcl|cTer
KILRGVSKRILTGKK
>lcl|4444|lcl|HAPPENN&HAPPENN-RR90|lcl|subdataset_11|lcl|non-hemolytic
IKKIWSKIKKLWK
>lcl|80|lcl|HAPPENN&HAPPENN-RR90&HAPPENN-hard|lcl|subdataset_4|lcl|hemolytic
FLPILAGLAAKIVPKLFCLATKKC
>lcl|760|lcl|HAPPENN&HAPPENN-RR90&HAPPENN-hard|lcl|subdataset_8|lcl|hemolytic|lcl|cTer
KWCFRVCYRGICYRRCR
>lcl|3684|lcl|HAPPENN&HAPPENN-RR90|lcl|subdataset_4|lcl|non-hemolytic|lcl|cTer
INLKAIAAFAKKLL
>lcl|3991|lcl|HAPPENN&HAPPENN-hard|lcl|subdataset_11|lcl|non-hemolytic|lcl|cTer
AVVDILKGAAKDIAGHLASKVMNKL
>lcl|4372|lcl|HAPPENN|lcl|subdataset_10|lcl|non-hemolytic|lcl|cTer
GLMKRIKTML
>lcl|4499|lcl|HAPPENN&HAPPENN-RR90|lcl|subdataset_8|lcl|non-hemolytic
RIKRFWPVVIRTVVAGYNLYRAI
>lcl|2954|lcl|HAPPENN|lcl|subdataset_6|lcl|non-hemolytic|lcl|cTer
GLAKRIKTLL
>lcl|232|lcl|HAPPENN&HAPPENN-RR90&HAPPENN-hard|lcl|subdataset_4|lcl|hemolytic|lcl|cTer
LKKLLKWLLKLLK
>lcl|4026|lcl|HAPPENN&HAPPENN-RR90|lcl|subdataset_5|lcl|non-hemolytic|lcl|nTer|lcl|cTer
NVMTYWWLDPPL
>lcl|2208|lcl|HAPPENN&HAPPENN-RR90|lcl|subdataset_4|lcl|non-hemolytic
TQQAFQKFLAAVTSALGKQYH
>lcl|891|lcl|HAPPENN&HAPPENN-RR90|lcl|subdataset_12|lcl|non-hemolytic|lcl|cTer
KWKSFIKKAKTSFLHSAKKF
>lcl|2481|lcl|HAPPENN&HAPPENN-RR90&HAPPENN-hard|lcl|subdataset_7|lcl|hemolytic|lcl|cTer
GIWKKWIKKWLNVLKNLF
>lcl|3289|lcl|HAPPENN&HAPPENN-RR90&HAPPENN-hard|lcl|subdataset_4|lcl|hemolytic
GLRSKIWLWVLLMIWQESNKFKKM
>lcl|1446|lcl|HAPPENN&HAPPENN-RR90&HAPPENN-hard|lcl|subdataset_12|lcl|non-hemolytic
SSLLEKGLDGAKKAVGGLGKLGKDAVEDL
>lcl|1144|lcl|HAPPENN|lcl|subdataset_6|lcl|hemolytic|lcl|cTer
LLAGLAANFLPKIFCKITKRC
>lcl|2240|lcl|HAPPENN&HAPPENN-RR90|lcl|subdataset_7|lcl|non-hemolytic|lcl|cTer
KRFIKWYNAWNEKRRVY
>lcl|3034|lcl|HAPPENN&HAPPENN-RR90&HAPPENN-hard|lcl|subdataset_6|lcl|hemolytic|lcl|cTer
GMWSKIPGHLIR
```

```
>lcl|2667|lcl|HAPPENN|lcl|subdataset_9|lcl|non-hemolytic|lcl|cTer
ALYLAIRKR
>lcl|4367|lcl|HAPPENN&HAPPENN-RR90&HAPPENN-hard|lcl|subdataset_9|lcl|hemolytic
AIPWSIWWRLLFKG
>lcl|3501|lcl|HAPPENN&HAPPENN-RR90&HAPPENN-hard|lcl|subdataset_4|lcl|hemolytic|lcl|cTer
FKRLKKLFKKIWNWK
>lcl|2420|lcl|HAPPENN&HAPPENN-RR90&HAPPENN-hard|lcl|subdataset_6|lcl|non-hemolytic
RTCIPIPLVMC
>lcl|379|lcl|HAPPENN|lcl|subdataset_12|lcl|hemolytic
KLKFKLKQ
>lcl|189|lcl|HAPPENN&HAPPENN-RR90&HAPPENN-hard|lcl|subdataset_5|lcl|hemolytic
PICKRNGLPVCGETCTLGTCYTQGCTCSW
>lcl|2671|lcl|HAPPENN&HAPPENN-hard|lcl|subdataset_11|lcl|non-hemolytic
SILDKIKNVALGVARGAGTGILKALLCKLDKSC
>lcl|1105|lcl|HAPPENN&HAPPENN-RR90&HAPPENN-hard|lcl|subdataset_7|lcl|hemolytic|lcl|cTer
ILGKLLSTAAGLLKNL
>lcl|1134|lcl|HAPPENN&HAPPENN-RR90&HAPPENN-hard|lcl|subdataset_8|lcl|non-hemolytic|lcl|cTer
GILGKLWEGFKSIV
>lcl|2997|lcl|HAPPENN&HAPPENN-RR90&HAPPENN-hard|lcl|subdataset_8|lcl|hemolytic|lcl|cTer
GLWSKIKAAGKEAAKAAAKAAGKAALNAVSEAV
>lcl|3428|lcl|HAPPENN&HAPPENN-hard|lcl|subdataset_9|lcl|non-hemolytic|lcl|cTer
KILGVSKRILTGKK
>lcl|4536|lcl|HAPPENN&HAPPENN-hard|lcl|subdataset_11|lcl|non-hemolytic|lcl|cTer
FFHHIFRGIVHVGKTAHRLVTG
>lcl|2427|lcl|HAPPENN&HAPPENN-RR90|lcl|subdataset_8|lcl|non-hemolytic|lcl|cTer
LRPLLRPLLRPLLRPLLRPLLRPLLRPL
>lcl|2759|lcl|HAPPENN|lcl|subdataset_11|lcl|hemolytic
SMLSVLKKLGKVGLGFVACKINKQC
>lcl|695|lcl|HAPPENN|lcl|subdataset_12|lcl|non-hemolytic|lcl|cTer
KPLLKKLLKKL
>lcl|3974|lcl|HAPPENN&HAPPENN-hard|lcl|subdataset_8|lcl|non-hemolytic|lcl|cTer
GVVDILKGAAKDIAGHASKVMNKL
>lcl|2233|lcl|HAPPENN&HAPPENN-RR90&HAPPENN-hard|lcl|subdataset_4|lcl|hemolytic|lcl|cTer
WKRIVRRIKRWLR
>lcl|1340|lcl|HAPPENN&HAPPENN-RR90&HAPPENN-hard|lcl|subdataset_7|lcl|hemolytic|lcl|cTer
KNWGKILKHIIK
>lcl|2333|lcl|HAPPENN|lcl|subdataset_9|lcl|non-hemolytic|lcl|nTer|lcl|cTer
KYTGKCYKKKNECK
>lcl|2971|lcl|HAPPENN|lcl|subdataset_10|lcl|hemolytic|lcl|cTer
GLLKFIKTLL
>lcl|915|lcl|HAPPENN&HAPPENN-RR90&HAPPENN-hard|lcl|subdataset_5|lcl|hemolytic|lcl|cTer
VNSKKISPKSIKVSK
>lcl|2922|lcl|HAPPENN|lcl|subdataset_10|lcl|non-hemolytic
KKLLKFKKLQ
>lcl|4403|lcl|HAPPENN&HAPPENN-RR90|lcl|subdataset_6|lcl|non-hemolytic
TLISWIKNKRKQRPRVSRRRRRRGGRRRR
>lcl|3107|lcl|HAPPENN&HAPPENN-RR90&HAPPENN-hard|lcl|subdataset_6|lcl|hemolytic|lcl|cTer
FLSTLWNAAKSIF
>lcl|1708|lcl|HAPPENN&HAPPENN-RR90&HAPPENN-hard|lcl|subdataset_11|lcl|hemolytic|lcl|cTer
TKPKGTKPKGTKPKGTKPKG
>lcl|3783|lcl|HAPPENN|lcl|subdataset_7|lcl|non-hemolytic|lcl|cTer
GIHRILKYGKPS
>lcl|1456|lcl|HAPPENN|lcl|subdataset_9|lcl|hemolytic|lcl|cTer
GLGSVFGRLARIGRVIPKV
>lcl|402|lcl|HAPPENN&HAPPENN-RR90&HAPPENN-hard|lcl|subdataset_8|lcl|hemolytic|lcl|cTer
VRRFPWWWAFLRR
>lcl|1011|lcl|HAPPENN&HAPPENN-hard|lcl|subdataset_7|lcl|non-hemolytic
KLAKKLAKLAKLAKAL
>lcl|732|lcl|HAPPENN&HAPPENN-RR90|lcl|subdataset_8|lcl|non-hemolytic|lcl|nTer|lcl|cTer
SADLVKKIWDNPAL
>lcl|553|lcl|HAPPENN|lcl|subdataset_5|lcl|non-hemolytic|lcl|cTer
DSHAKRHHGYKIKFHEKHHSLRGY
>lcl|2980|lcl|HAPPENN|lcl|subdataset_11|lcl|hemolytic|lcl|cTer
GLLKRIKWLL
>lcl|419|lcl|HAPPENN&HAPPENN-RR90&HAPPENN-hard|lcl|subdataset_12|lcl|hemolytic|lcl|cTe
```

r
HFLLKLVNLAKKIL
>lcl|4733|lcl|HAPPENN|lcl|subdataset_5|lcl|hemolytic|lcl|cTer
KIAKVALKALKIAKVALKAL
>lcl|1220|lcl|HAPPENN&HAPPENN-RR90&HAPPENN-hard|lcl|subdataset_11|lcl|hemolytic|lcl|cTer
LKLKSIVSWAKLVL
>lcl|4660|lcl|HAPPENN|lcl|subdataset_4|lcl|hemolytic|lcl|cTer
FFFLRRIF
>lcl|2818|lcl|HAPPENN&HAPPENN-hard|lcl|subdataset_5|lcl|non-hemolytic|lcl|cTer
HCLAIGRR
>lcl|2384|lcl|HAPPENN&HAPPENN-RR90|lcl|subdataset_4|lcl|non-hemolytic|lcl|cTer
RWWRRWRRWWR
>lcl|3003|lcl|HAPPENN|lcl|subdataset_8|lcl|non-hemolytic|lcl|cTer
KKLAGLAKKWAGLAKKLAGLA
>lcl|4108|lcl|HAPPENN|lcl|subdataset_12|lcl|hemolytic|lcl|cTer
RFRRLRKKKRKRLKKI
>lcl|494|lcl|HAPPENN&HAPPENN-hard|lcl|subdataset_6|lcl|non-hemolytic
KVFKRLEKLFSKI
>lcl|3101|lcl|HAPPENN|lcl|subdataset_11|lcl|hemolytic|lcl|cTer
AFGMALKLLKKVL
>lcl|3656|lcl|HAPPENN&HAPPENN-hard|lcl|subdataset_10|lcl|non-hemolytic
RRRRWFWF
>lcl|2679|lcl|HAPPENN&HAPPENN-RR90&HAPPENN-hard|lcl|subdataset_10|lcl|non-hemolytic
GLMSTLKDFGKTAAKEIAQSLLSTASCKLAKTC
>lcl|1662|lcl|HAPPENN&HAPPENN-RR90&HAPPENN-hard|lcl|subdataset_12|lcl|hemolytic
FFPGIIKVAGAILPTAICAITKRC
>lcl|4045|lcl|HAPPENN&HAPPENN-hard|lcl|subdataset_11|lcl|non-hemolytic|lcl|cTer
LLKKLLKKLLKL
>lcl|1057|lcl|HAPPENN|lcl|subdataset_6|lcl|non-hemolytic|lcl|cTer
LKWLKKWLK
>lcl|2900|lcl|HAPPENN|lcl|subdataset_10|lcl|non-hemolytic
LKKKLFLKKQ
>lcl|1676|lcl|HAPPENN&HAPPENN-hard|lcl|subdataset_10|lcl|non-hemolytic|lcl|cTer
FRRFFKWFRRPFKFF
>lcl|3831|lcl|HAPPENN&HAPPENN-RR90|lcl|subdataset_12|lcl|non-hemolytic|lcl|cTer
RKKKVKLLKRLV
>lcl|4001|lcl|HAPPENN&HAPPENN-hard|lcl|subdataset_6|lcl|non-hemolytic
FLPLIGRVLSGIL
>lcl|3467|lcl|HAPPENN|lcl|subdataset_4|lcl|non-hemolytic|lcl|cTer
ALLHHGLNCAKGVLA
>lcl|2204|lcl|HAPPENN&HAPPENN-RR90|lcl|subdataset_7|lcl|non-hemolytic|lcl|cTer
KLVKTQLKVLK
>lcl|1453|lcl|HAPPENN|lcl|subdataset_7|lcl|non-hemolytic|lcl|cTer
KWKKLLKKPLLKK
>lcl|917|lcl|HAPPENN&HAPPENN-RR90|lcl|subdataset_7|lcl|non-hemolytic|lcl|cTer
KSLVRRWRSRW
>lcl|4595|lcl|HAPPENN|lcl|subdataset_10|lcl|non-hemolytic
SGKLCCRRKK
>lcl|3858|lcl|HAPPENN|lcl|subdataset_6|lcl|non-hemolytic|lcl|cTer
RKKKFKFFKRFF
>lcl|4043|lcl|HAPPENN|lcl|subdataset_5|lcl|non-hemolytic|lcl|cTer
KLKKLLKKLLKK
>lcl|170|lcl|HAPPENN&HAPPENN-RR90&HAPPENN-hard|lcl|subdataset_10|lcl|hemolytic
LLLFLLKKRKKRKY
>lcl|1611|lcl|HAPPENN|lcl|subdataset_8|lcl|non-hemolytic|lcl|cTer
RRWWRHWRR
>lcl|2458|lcl|HAPPENN|lcl|subdataset_4|lcl|non-hemolytic
WRRFWRR
>lcl|49|lcl|HAPPENN&HAPPENN-RR90&HAPPENN-hard|lcl|subdataset_7|lcl|non-hemolytic
FVLPLVMCKILRKC
>lcl|110|lcl|HAPPENN&HAPPENN-RR90&HAPPENN-hard|lcl|subdataset_5|lcl|hemolytic
RGGRLCYCRGWICFCVGR
>lcl|3343|lcl|HAPPENN|lcl|subdataset_12|lcl|non-hemolytic|lcl|cTer
WKSYVRRWR
>lcl|908|lcl|HAPPENN|lcl|subdataset_10|lcl|non-hemolytic|lcl|cTer
GLPRKILAAIAKKK
>lcl|1683|lcl|HAPPENN&HAPPENN-hard|lcl|subdataset_6|lcl|non-hemolytic
GLNALKKVFQGIHKAIKKINNHVQ
>lcl|4443|lcl|HAPPENN&HAPPENN-RR90|lcl|subdataset_7|lcl|non-hemolytic
IKKIWSKIKKWLK
>lcl|3481|lcl|HAPPENN|lcl|subdataset_10|lcl|non-hemolytic
VEIHVWEGV

```
>lcl|2942|lcl|HAPPENN&HAPPENN-RR90&HAPPENN-hard|lcl|subdataset_8|lcl|non-hemolytic|lcl|cTer
ILKWKWKWWKWRR
>lcl|1375|lcl|HAPPENN&HAPPENN-RR90&HAPPENN-hard|lcl|subdataset_6|lcl|non-hemolytic
IIKVPLKKFKSMREVMRDHGIKAPVVDPATKY
>lcl|3070|lcl|HAPPENN&HAPPENN-RR90&HAPPENN-hard|lcl|subdataset_7|lcl|hemolytic|lcl|cTer
NVWKKVLGKIIKVAK
>lcl|1196|lcl|HAPPENN|lcl|subdataset_8|lcl|hemolytic
KLLKKLLKLWKKLLKKLK
>lcl|1578|lcl|HAPPENN|lcl|subdataset_12|lcl|hemolytic|lcl|cTer
ALLKTMLKKLGTMAL
>lcl|4500|lcl|HAPPENN|lcl|subdataset_12|lcl|non-hemolytic
RIKRFWPVVIRTVVAGYNLYRA
>lcl|854|lcl|HAPPENN&HAPPENN-RR90&HAPPENN-hard|lcl|subdataset_11|lcl|hemolytic|lcl|cTer
FSGGNCRGFRRRCFCTK
>lcl|144|lcl|HAPPENN|lcl|subdataset_7|lcl|hemolytic
ICYCRRRFCVCVGR
>lcl|1579|lcl|HAPPENN|lcl|subdataset_8|lcl|hemolytic|lcl|cTer
KALWKTMLKKLGTMAL
>lcl|3075|lcl|HAPPENN|lcl|subdataset_12|lcl|hemolytic|lcl|cTer
NVWKKILGKIIKVAK
>lcl|2862|lcl|HAPPENN&HAPPENN-RR90|lcl|subdataset_5|lcl|non-hemolytic|lcl|cTer
RLWRRWRRWRR
>lcl|1206|lcl|HAPPENN&HAPPENN-RR90&HAPPENN-hard|lcl|subdataset_10|lcl|hemolytic|lcl|cTer
IIGAIAAALPHVINAIKNTF
>lcl|1632|lcl|HAPPENN&HAPPENN-RR90|lcl|subdataset_4|lcl|non-hemolytic
CGYKYGCMVKVDR
>lcl|2823|lcl|HAPPENN|lcl|subdataset_8|lcl|non-hemolytic|lcl|cTer
ALWLAIRRR
>lcl|860|lcl|HAPPENN&HAPPENN-RR90&HAPPENN-hard|lcl|subdataset_6|lcl|hemolytic|lcl|cTer
KFAKKFKWFAKAAFKFFKK
>lcl|3546|lcl|HAPPENN&HAPPENN-RR90&HAPPENN-hard|lcl|subdataset_10|lcl|hemolytic
GVFGLLAKAALKGASKLIPHLLPSRQQ
>lcl|2728|lcl|HAPPENN&HAPPENN-hard|lcl|subdataset_11|lcl|non-hemolytic
FLGALFKALSKLL
>lcl|3290|lcl|HAPPENN&HAPPENN-RR90&HAPPENN-hard|lcl|subdataset_6|lcl|non-hemolytic|lcl|cTer
GVVDILKGAAKDLAGHLATKVMNKL
>lcl|2937|lcl|HAPPENN|lcl|subdataset_5|lcl|non-hemolytic
KKKKKFKKLQ
>lcl|228|lcl|HAPPENN&HAPPENN-hard|lcl|subdataset_10|lcl|non-hemolytic|lcl|cTer
KLLKWLLKL
>lcl|2855|lcl|HAPPENN&HAPPENN-RR90|lcl|subdataset_4|lcl|non-hemolytic|lcl|cTer
KKWKKWKKWKK
>lcl|3085|lcl|HAPPENN|lcl|subdataset_6|lcl|hemolytic|lcl|cTer
VNWKKILGKI
>lcl|3260|lcl|HAPPENN&HAPPENN-RR90&HAPPENN-hard|lcl|subdataset_12|lcl|hemolytic
FLPVIAGLAAKVLPKLFCAITKKC
>lcl|2159|lcl|HAPPENN&HAPPENN-RR90|lcl|subdataset_8|lcl|non-hemolytic|lcl|cTer
RIVQRIAKWAKKWYKAGK
>lcl|3033|lcl|HAPPENN&HAPPENN-RR90&HAPPENN-hard|lcl|subdataset_8|lcl|hemolytic|lcl|cTer
GMWSKILPHLIR
>lcl|4280|lcl|HAPPENN&HAPPENN-RR90|lcl|subdataset_10|lcl|non-hemolytic
ACPIFTKIQGTYRGKAKRIGRRIC
>lcl|4192|lcl|HAPPENN&HAPPENN-RR90&HAPPENN-hard|lcl|subdataset_7|lcl|non-hemolytic
KKLFKKILKYLGIGKFLHSAKKDEL
>lcl|2303|lcl|HAPPENN|lcl|subdataset_11|lcl|non-hemolytic
ERILSILRHQNLLKE
>lcl|1194|lcl|HAPPENN&HAPPENN-RR90|lcl|subdataset_9|lcl|non-hemolytic
KKLKKLKKKWKKLKKKLK
>lcl|2597|lcl|HAPPENN&HAPPENN-RR90&HAPPENN-hard|lcl|subdataset_4|lcl|hemolytic
KWKLFKKIGIGAVLKVLTTGLPALKLTK
>lcl|3421|lcl|HAPPENN|lcl|subdataset_11|lcl|non-hemolytic|lcl|cTer
KILRGVSKKIMRTFLRR
>lcl|4231|lcl|HAPPENN&HAPPENN-RR90|lcl|subdataset_8|lcl|non-hemolytic|lcl|cTer
FRFRCRRRFCRFRF
>lcl|2663|lcl|HAPPENN|lcl|subdataset_11|lcl|non-hemolytic|lcl|cTer
ALLLAIRKR
>lcl|4352|lcl|HAPPENN&HAPPENN-RR90&HAPPENN-hard|lcl|subdataset_6|lcl|hemolytic|lcl|cTe
```

```
r
GLFDVIKKLLKKIKGL
>lcl|3370|lcl|HAPPENN&HAPPENN-RR90&HAPPENN-hard|lcl|subdataset_4|lcl|hemolytic|lcl|cTer
ALPWKWPWWPWRR
>lcl|3016|lcl|HAPPENN&HAPPENN-RR90&HAPPENN-hard|lcl|subdataset_9|lcl|hemolytic|lcl|cTer
GMWSKILGHLIR
>lcl|1147|lcl|HAPPENN&HAPPENN-RR90|lcl|subdataset_9|lcl|non-hemolytic
CKTRTSTNTDHLWIAKSKARLSET
>lcl|3813|lcl|HAPPENN&HAPPENN-RR90|lcl|subdataset_7|lcl|non-hemolytic|lcl|cTer
RKRFARFAKRAV
>lcl|1405|lcl|HAPPENN&HAPPENN-RR90&HAPPENN-hard|lcl|subdataset_5|lcl|hemolytic|lcl|cTer
INWKKIFEKVSNLV
>lcl|721|lcl|HAPPENN&HAPPENN-RR90&HAPPENN-hard|lcl|subdataset_10|lcl|non-hemolytic
SLGGVISGAKKVAKVAIPIGKAVLPVVAKLVG
>lcl|1932|lcl|HAPPENN&HAPPENN-RR90&HAPPENN-hard|lcl|subdataset_6|lcl|non-hemolytic|lcl|cTer
AIGSILGRLAKGLPTLKSWIKNR
>lcl|968|lcl|HAPPENN&HAPPENN-RR90&HAPPENN-hard|lcl|subdataset_9|lcl|hemolytic|lcl|cTer
FFRLLFHGVHHVGKIKPRA
>lcl|331|lcl|HAPPENN&HAPPENN-RR90&HAPPENN-hard|lcl|subdataset_7|lcl|non-hemolytic
VIPFVASVAAEMMQHVYCAASRKC
>lcl|348|lcl|HAPPENN&HAPPENN-RR90&HAPPENN-hard|lcl|subdataset_9|lcl|hemolytic
MAQDIISTIGDLVKWIIDTVNKFTKK
>lcl|984|lcl|HAPPENN&HAPPENN-RR90&HAPPENN-hard|lcl|subdataset_9|lcl|hemolytic|lcl|cTer
GIRKWFKKAAHVGKKVGKVALNAYL
>lcl|4182|lcl|HAPPENN|lcl|subdataset_9|lcl|non-hemolytic
KKLFKKILKYLAGPAKFLHSAK
>lcl|955|lcl|HAPPENN&HAPPENN-hard|lcl|subdataset_5|lcl|non-hemolytic|lcl|cTer
FLPLIGRVLSGIA
>lcl|0|lcl|HAPPENN&HAPPENN-RR90&HAPPENN-hard|lcl|subdataset_6|lcl|hemolytic
ALWMTLLKKVLKAAAKAALNAVLVGANA
>lcl|3624|lcl|HAPPENN&HAPPENN-hard|lcl|subdataset_11|lcl|non-hemolytic|lcl|cTer
GRFKRFRKKFKKLFKKASPVIPLLHL
>lcl|3182|lcl|HAPPENN&HAPPENN-RR90&HAPPENN-hard|lcl|subdataset_4|lcl|non-hemolytic|lcl|cTer
LLGMIPVAISAISALSKL
>lcl|869|lcl|HAPPENN&HAPPENN-hard|lcl|subdataset_8|lcl|non-hemolytic
KKFFLKVLTKIRCKVAGGCRT
>lcl|2785|lcl|HAPPENN&HAPPENN-RR90&HAPPENN-hard|lcl|subdataset_8|lcl|non-hemolytic
FLGAVVKALSKLL
>lcl|4176|lcl|HAPPENN|lcl|subdataset_5|lcl|hemolytic
SKKLFKKILKYLAGPATTGLPALISW
>lcl|3004|lcl|HAPPENN&HAPPENN-RR90|lcl|subdataset_12|lcl|non-hemolytic|lcl|cTer
KLKAGLAKWKAGLAKLKAGLA
>lcl|1567|lcl|HAPPENN|lcl|subdataset_4|lcl|hemolytic|lcl|cTer
ALWKTMLKKLGTMALHAGKAALGAAADTI
>lcl|2248|lcl|HAPPENN|lcl|subdataset_5|lcl|non-hemolytic|lcl|cTer
YPCKLNLKLGKVPFH
>lcl|2926|lcl|HAPPENN|lcl|subdataset_7|lcl|non-hemolytic
LKLKKFKKLQ
>lcl|2623|lcl|HAPPENN|lcl|subdataset_9|lcl|hemolytic|lcl|cTer
GIGAVLAVLTTGLPALISWIKRKRQQ
>lcl|3604|lcl|HAPPENN&HAPPENN-RR90&HAPPENN-hard|lcl|subdataset_10|lcl|hemolytic|lcl|cTer
GIGGALLSAGKAALKGLAKGLAEHFAN
>lcl|3449|lcl|HAPPENN&HAPPENN-RR90&HAPPENN-hard|lcl|subdataset_4|lcl|hemolytic
GLPVCGETCFGGTCNTPGCICDPWPVCTRN
>lcl|4504|lcl|HAPPENN|lcl|subdataset_5|lcl|non-hemolytic
RIKRFWPVVIRTVVAGYNL
>lcl|3206|lcl|HAPPENN&HAPPENN-RR90&HAPPENN-hard|lcl|subdataset_10|lcl|non-hemolytic|lcl|cTer
GIGTKILGGVKAALKGALKELASTYVN
>lcl|4493|lcl|HAPPENN&HAPPENN-RR90&HAPPENN-hard|lcl|subdataset_12|lcl|hemolytic
GMASKLAKVLPHVVKLIK
>lcl|3474|lcl|HAPPENN|lcl|subdataset_6|lcl|non-hemolytic|lcl|cTer
LRPAILVRTK
>lcl|1854|lcl|HAPPENN&HAPPENN-RR90&HAPPENN-hard|lcl|subdataset_5|lcl|hemolytic
VLPLLETCSMTCWENNQTFGK
>lcl|2438|lcl|HAPPENN&HAPPENN-hard|lcl|subdataset_9|lcl|non-hemolytic
HLLKRVQRELIGWLDWLK
```

```
>lcl|2218|lcl|HAPPENN&HAPPENN-hard|lcl|subdataset_9|lcl|non-hemolytic|lcl|cTer
LLWIALRKK
>lcl|1643|lcl|HAPPENN&HAPPENN-RR90|lcl|subdataset_7|lcl|non-hemolytic
QYRPGSFGPLNQK
>lcl|3532|lcl|HAPPENN&HAPPENN-hard|lcl|subdataset_5|lcl|non-hemolytic|lcl|cTer
FITGLIGGLMKAL
>lcl|2310|lcl|HAPPENN&HAPPENN-RR90|lcl|subdataset_9|lcl|non-hemolytic
RCFRRRGKLTC
>lcl|3265|lcl|HAPPENN&HAPPENN-RR90&HAPPENN-hard|lcl|subdataset_7|lcl|hemolytic
GEFLKCGESCVQGECYTPGCSCDWPICKKN
>lcl|2834|lcl|HAPPENN|lcl|subdataset_11|lcl|hemolytic|lcl|cTer
FFPLVLGALGSILPKIF
>lcl|2327|lcl|HAPPENN&HAPPENN-RR90&HAPPENN-hard|lcl|subdataset_6|lcl|hemolytic|lcl|cTer
FFKLIPKLVKKLIKAFK
>lcl|672|lcl|HAPPENN&HAPPENN-RR90|lcl|subdataset_9|lcl|non-hemolytic|lcl|cTer
KKKAAAFAAWAAFAKKK
>lcl|3088|lcl|HAPPENN|lcl|subdataset_9|lcl|hemolytic|lcl|cTer
GKIIKVVK
>lcl|4038|lcl|HAPPENN&HAPPENN-RR90|lcl|subdataset_12|lcl|non-hemolytic
KGCALVKVRGLTLKVCK
>lcl|238|lcl|HAPPENN|lcl|subdataset_7|lcl|hemolytic
GKLIKKFGRKAISYAVKKARGKH
>lcl|1458|lcl|HAPPENN&HAPPENN-RR90&HAPPENN-hard|lcl|subdataset_6|lcl|non-hemolytic|lcl|cTer
VRRFKWWWKFLRR
>lcl|1128|lcl|HAPPENN|lcl|subdataset_11|lcl|hemolytic
FLGFLHHLF
>lcl|483|lcl|HAPPENN&HAPPENN-hard|lcl|subdataset_7|lcl|non-hemolytic
VTLASHLPSDFTPAVHASLDKFLANVSTVL
>lcl|4097|lcl|HAPPENN&HAPPENN-RR90|lcl|subdataset_5|lcl|non-hemolytic
SKVGRHLRRFLHRAHRKL
>lcl|1486|lcl|HAPPENN&HAPPENN-RR90&HAPPENN-hard|lcl|subdataset_8|lcl|hemolytic
CGESCVFIPCISTLLGCSCKNKVCYRNGVIP
>lcl|1834|lcl|HAPPENN&HAPPENN-RR90|lcl|subdataset_5|lcl|non-hemolytic|lcl|cTer
KVKVKVKVPPTKVKVKVK
>lcl|2320|lcl|HAPPENN&HAPPENN-RR90&HAPPENN-hard|lcl|subdataset_10|lcl|non-hemolytic
KIFGAIWPLALGALKNLIK
>lcl|4487|lcl|HAPPENN&HAPPENN-RR90&HAPPENN-hard|lcl|subdataset_11|lcl|non-hemolytic|lcl|cTer
INWLKAKKVAGMIL
>lcl|4714|lcl|HAPPENN&HAPPENN-RR90&HAPPENN-hard|lcl|subdataset_8|lcl|hemolytic
FFFHIIKGLFHAGRMIHGLVNRRRHRH
>lcl|184|lcl|HAPPENN&HAPPENN-RR90&HAPPENN-hard|lcl|subdataset_12|lcl|non-hemolytic|lcl|nTer|lcl|cTer
KWKKFKKIGKVLKVL
>lcl|3764|lcl|HAPPENN&HAPPENN-RR90|lcl|subdataset_4|lcl|non-hemolytic|lcl|cTer
RRWYRWAWRMR
>lcl|3654|lcl|HAPPENN&HAPPENN-RR90|lcl|subdataset_10|lcl|non-hemolytic|lcl|cTer
ILPWKKPKKPWRR
>lcl|4040|lcl|HAPPENN&HAPPENN-RR90|lcl|subdataset_6|lcl|non-hemolytic
HKCAKIKWRGVHVKYCA
>lcl|514|lcl|HAPPENN&HAPPENN-RR90&HAPPENN-hard|lcl|subdataset_5|lcl|non-hemolytic|lcl|cTer
GLLKPLLKIAAKVGSNLL
>lcl|3042|lcl|HAPPENN&HAPPENN-RR90&HAPPENN-hard|lcl|subdataset_11|lcl|hemolytic|lcl|cTer
KKWMSLLKHILK
>lcl|352|lcl|HAPPENN|lcl|subdataset_11|lcl|hemolytic
IGDLVKWIIDTVNKFTKK
>lcl|4420|lcl|HAPPENN&HAPPENN-hard|lcl|subdataset_8|lcl|non-hemolytic|lcl|cTer
FKRLKKLAKKIWNWK
>lcl|4735|lcl|HAPPENN&HAPPENN-RR90|lcl|subdataset_6|lcl|non-hemolytic
FFGRLKSVWSAVKHGWKAAKSR
>lcl|3266|lcl|HAPPENN|lcl|subdataset_9|lcl|hemolytic
GIPCGESCVFIPCITGAIGCSCKSKVCYRN
>lcl|1336|lcl|HAPPENN&HAPPENN-RR90&HAPPENN-hard|lcl|subdataset_6|lcl|hemolytic|lcl|cTer
LNWGAIKKHIIK
>lcl|4209|lcl|HAPPENN&HAPPENN-RR90&HAPPENN-hard|lcl|subdataset_8|lcl|hemolytic|lcl|cTer
FLPFVGKLLSGLL
>lcl|813|lcl|HAPPENN&HAPPENN-RR90|lcl|subdataset_8|lcl|non-hemolytic|lcl|cTer
```

```
RWRSFFKKAAHRGKHVGKRARTHYL
>lcl|2901|lcl|HAPPENN|lcl|subdataset_12|lcl|non-hemolytic
KKLLKFLKKQ
>lcl|3342|lcl|HAPPENN|lcl|subdataset_6|lcl|non-hemolytic|lcl|cTer
WWSYVRRWRSR
>lcl|3500|lcl|HAPPENN&HAPPENN-RR90|lcl|subdataset_10|lcl|non-hemolytic|lcl|cTer
AKRLKKLAKKIWNWK
>lcl|1278|lcl|HAPPENN|lcl|subdataset_4|lcl|hemolytic|lcl|cTer
GFLSILKKVLP
>lcl|3989|lcl|HAPPENN&HAPPENN-hard|lcl|subdataset_8|lcl|non-hemolytic|lcl|cTer
GAVDILKGAAKDIAGHLASKVMNKL
>lcl|4841|lcl|HAPPENN|lcl|subdataset_6|lcl|non-hemolytic|lcl|nTer|lcl|cTer
MLKKFRGMF
>lcl|1422|lcl|HAPPENN|lcl|subdataset_9|lcl|hemolytic|lcl|cTer
NWKKIASIGKEVLKAL
>lcl|1767|lcl|HAPPENN&HAPPENN-RR90|lcl|subdataset_9|lcl|non-hemolytic|lcl|cTer
RRRYIGRYVRFWK
>lcl|392|lcl|HAPPENN&HAPPENN-hard|lcl|subdataset_5|lcl|non-hemolytic|lcl|cTer
KWKKLLKKPLKLLKKLL
>lcl|3168|lcl|HAPPENN&HAPPENN-RR90&HAPPENN-hard|lcl|subdataset_7|lcl|hemolytic|lcl|cTer
FLPGLIAGIAKML
>lcl|3903|lcl|HAPPENN&HAPPENN-RR90&HAPPENN-hard|lcl|subdataset_10|lcl|hemolytic|lcl|cTer
GIWKTIKSMGKVFAGAIKQNL
>lcl|2944|lcl|HAPPENN&HAPPENN-hard|lcl|subdataset_7|lcl|non-hemolytic
GILDTLKQFAKGVGKDLVKGAAQGVLSTV
>lcl|3740|lcl|HAPPENN|lcl|subdataset_7|lcl|hemolytic|lcl|nTer|lcl|cTer
RKLRRLKRKIAHKVKKY
>lcl|3795|lcl|HAPPENN&HAPPENN-RR90&HAPPENN-hard|lcl|subdataset_9|lcl|non-hemolytic|lcl|cTer
KRIVQRIKKWLR
>lcl|251|lcl|HAPPENN&HAPPENN-RR90&HAPPENN-hard|lcl|subdataset_5|lcl|hemolytic|lcl|cTer
INWLKLGKMVIDAL
>lcl|3911|lcl|HAPPENN&HAPPENN-RR90&HAPPENN-hard|lcl|subdataset_5|lcl|hemolytic|lcl|cTer
VQLRIRVAVIRA
>lcl|2733|lcl|HAPPENN&HAPPENN-RR90&HAPPENN-hard|lcl|subdataset_9|lcl|non-hemolytic
GIKIAKKAITIAKKIAKIYW
>lcl|3425|lcl|HAPPENN|lcl|subdataset_7|lcl|non-hemolytic|lcl|cTer
KILGVSKKIMRRISKDILTGKK
>lcl|4799|lcl|HAPPENN|lcl|subdataset_11|lcl|non-hemolytic
KKWMQKVIDRFGG
>lcl|456|lcl|HAPPENN|lcl|subdataset_11|lcl|non-hemolytic
FKCRRWQWRMKKLG
>lcl|3010|lcl|HAPPENN&HAPPENN-hard|lcl|subdataset_5|lcl|non-hemolytic
FLPGLLAGLL
>lcl|4622|lcl|HAPPENN&HAPPENN-RR90&HAPPENN-hard|lcl|subdataset_5|lcl|non-hemolytic|lcl|cTer
GRRPRPRPRPWWWW
>lcl|3390|lcl|HAPPENN&HAPPENN-RR90&HAPPENN-hard|lcl|subdataset_8|lcl|hemolytic|lcl|cTer
ILGWKWGWWGWRR
>lcl|2140|lcl|HAPPENN&HAPPENN-RR90|lcl|subdataset_11|lcl|non-hemolytic
EVASFDKSKLK
>lcl|741|lcl|HAPPENN&HAPPENN-RR90&HAPPENN-hard|lcl|subdataset_9|lcl|hemolytic|lcl|cTer
ILGKIWKGIKSLF
>lcl|3333|lcl|HAPPENN&HAPPENN-hard|lcl|subdataset_7|lcl|non-hemolytic|lcl|cTer
LRRLWLRANRL
>lcl|4814|lcl|HAPPENN&HAPPENN-RR90&HAPPENN-hard|lcl|subdataset_4|lcl|hemolytic|lcl|cTer
FPFLLSLIPSAISAIKRL
>lcl|1653|lcl|HAPPENN&HAPPENN-RR90&HAPPENN-hard|lcl|subdataset_5|lcl|non-hemolytic
ASVVNKLTGGVAGLLK
>lcl|472|lcl|HAPPENN|lcl|subdataset_4|lcl|non-hemolytic
QAKIRVRLSA
>lcl|43|lcl|HAPPENN&HAPPENN-RR90&HAPPENN-hard|lcl|subdataset_5|lcl|hemolytic|lcl|cTer
FLGSIVGALASALPSLISKIRN
>lcl|962|lcl|HAPPENN&HAPPENN-RR90&HAPPENN-hard|lcl|subdataset_6|lcl|hemolytic|lcl|cTer
GRRKRKWLRRIGKGVKIIGGAALDHL
>lcl|2399|lcl|HAPPENN&HAPPENN-RR90&HAPPENN-hard|lcl|subdataset_8|lcl|hemolytic|lcl|cTer
FLSLIPKIISAISALINHF
```

```
>lcl|4244|lcl|HAPPENN&HAPPENN-RR90&HAPPENN-hard|lcl|subdataset_9|lcl|non-hemolytic
LRLKSIVSYAKKVL
>lcl|4288|lcl|HAPPENN&HAPPENN-hard|lcl|subdataset_11|lcl|non-hemolytic
GVFDIIKGAGKQLIAHAMEKIAEKVGLNKDGN
>lcl|1349|lcl|HAPPENN&HAPPENN-RR90&HAPPENN-hard|lcl|subdataset_11|lcl|hemolytic
LDVKKIICVACKIRPNPACKKICPK
>lcl|2364|lcl|HAPPENN|lcl|subdataset_12|lcl|hemolytic
FLFSLIRKAIGGLISAFK
>lcl|983|lcl|HAPPENN&HAPPENN-RR90&HAPPENN-hard|lcl|subdataset_12|lcl|hemolytic|lcl|cTer
GCKKWFKKAAHVGKNVGKVALNAYL
>lcl|1430|lcl|HAPPENN&HAPPENN-RR90&HAPPENN-hard|lcl|subdataset_8|lcl|hemolytic|lcl|cTer
IWNKIAKSIGKVLEKAL
>lcl|939|lcl|HAPPENN&HAPPENN-RR90&HAPPENN-hard|lcl|subdataset_12|lcl|hemolytic|lcl|cTer
FVQWFSKFLGKIL
>lcl|3964|lcl|HAPPENN&HAPPENN-hard|lcl|subdataset_12|lcl|non-hemolytic|lcl|cTer
GFGKALKLLKKVL
>lcl|86|lcl|HAPPENN&HAPPENN-RR90|lcl|subdataset_5|lcl|non-hemolytic|lcl|nTer|lcl|cTer
EWESFLETFESAKETVLHTALEAISS
>lcl|1588|lcl|HAPPENN&HAPPENN-RR90&HAPPENN-hard|lcl|subdataset_4|lcl|hemolytic|lcl|cTer
KIAGKIAAIAGKIAKIAGAIAKIAGKIA
>lcl|4162|lcl|HAPPENN&HAPPENN-RR90&HAPPENN-hard|lcl|subdataset_11|lcl|hemolytic
KKLFKKILKYLKKLFKKILKYLKDEL
>lcl|3645|lcl|HAPPENN&HAPPENN-RR90&HAPPENN-hard|lcl|subdataset_9|lcl|hemolytic
FLPLIAGVAANFLPKIFCLISKKC
>lcl|1754|lcl|HAPPENN&HAPPENN-RR90|lcl|subdataset_7|lcl|non-hemolytic
GRKKRRQRRRGGWMWVTNLRTD
>lcl|202|lcl|HAPPENN&HAPPENN-RR90&HAPPENN-hard|lcl|subdataset_5|lcl|non-hemolytic|lcl|cTer
GRILSFIKGLAEHL
>lcl|4462|lcl|HAPPENN&HAPPENN-RR90&HAPPENN-hard|lcl|subdataset_10|lcl|non-hemolytic|lcl|cTer
GIMSSLMKKLAKHIAK
>lcl|1421|lcl|HAPPENN&HAPPENN-RR90&HAPPENN-hard|lcl|subdataset_9|lcl|hemolytic|lcl|cTer
INWKKIASIGKEVLKAI
>lcl|3962|lcl|HAPPENN&HAPPENN-hard|lcl|subdataset_5|lcl|non-hemolytic|lcl|cTer
IKKIVSKIKKLLK
>lcl|143|lcl|HAPPENN|lcl|subdataset_12|lcl|hemolytic
VCYCRRRFCVCVGR
>lcl|3902|lcl|HAPPENN|lcl|subdataset_4|lcl|hemolytic|lcl|cTer
GIWKTIKSMGKVFAGAILQNL
>lcl|4024|lcl|HAPPENN&HAPPENN-RR90|lcl|subdataset_9|lcl|non-hemolytic|lcl|nTer|lcl|cTer
SYSMEHFRWGKPV
>lcl|3259|lcl|HAPPENN|lcl|subdataset_12|lcl|hemolytic
FLPIIAGVAAKVLPKLFCAITKKC
>lcl|3844|lcl|HAPPENN|lcl|subdataset_4|lcl|hemolytic
GLNALKKFFQGIHEAIKLINNHVQ
>lcl|875|lcl|HAPPENN|lcl|subdataset_4|lcl|non-hemolytic
NWCKRGRKQCKTHPH
>lcl|3279|lcl|HAPPENN&HAPPENN-RR90&HAPPENN-hard|lcl|subdataset_9|lcl|hemolytic
FLSLIPKLVKKIIKAFK
>lcl|2419|lcl|HAPPENN&HAPPENN-RR90|lcl|subdataset_8|lcl|non-hemolytic
RICTPIPFPMCY
>lcl|2019|lcl|HAPPENN&HAPPENN-RR90|lcl|subdataset_10|lcl|non-hemolytic|lcl|cTer
FFYNVIKIYGNMAGRISK
>lcl|266|lcl|HAPPENN&HAPPENN-hard|lcl|subdataset_7|lcl|non-hemolytic|lcl|cTer
ILPWKWPWLPLRR
>lcl|4255|lcl|HAPPENN&HAPPENN-RR90&HAPPENN-hard|lcl|subdataset_12|lcl|non-hemolytic
KFFKRLLKSVRRAVKKFRKKPRLIGLSTLL
>lcl|2054|lcl|HAPPENN&HAPPENN-RR90|lcl|subdataset_11|lcl|non-hemolytic
YKARRWAWRMK
>lcl|3731|lcl|HAPPENN|lcl|subdataset_9|lcl|hemolytic|lcl|nTer|lcl|cTer
RGLRRLGRKIAHGVKKGGPTVLRIIRIAG
>lcl|2650|lcl|HAPPENN&HAPPENN-RR90&HAPPENN-hard|lcl|subdataset_6|lcl|hemolytic|lcl|cTer
FLPILGNLLNGLL
>lcl|2918|lcl|HAPPENN|lcl|subdataset_5|lcl|non-hemolytic
KLLLKFKKKQ
```

```
>lcl|364|lcl|HAPPENN&HAPPENN-RR90&HAPPENN-hard|lcl|subdataset_11|lcl|non-hemolytic
VSAVAKVAMKKGAALLKKMGVKISPLK
>lcl|1774|lcl|HAPPENN&HAPPENN-RR90|lcl|subdataset_5|lcl|non-hemolytic
RLCRIVVIRVCR
>lcl|1334|lcl|HAPPENN|lcl|subdataset_7|lcl|hemolytic|lcl|cTer
LNWGKILKHIIK
>lcl|1860|lcl|HAPPENN|lcl|subdataset_7|lcl|non-hemolytic
AIGHCLGATL
>lcl|4489|lcl|HAPPENN&HAPPENN-RR90&HAPPENN-hard|lcl|subdataset_8|lcl|non-hemolytic|lcl|cTer
AKKVFKRLEKLFSKIWNDK
>lcl|3011|lcl|HAPPENN|lcl|subdataset_4|lcl|non-hemolytic
RAGLKFPVGRVHRLLR
>lcl|82|lcl|HAPPENN&HAPPENN-RR90&HAPPENN-hard|lcl|subdataset_10|lcl|hemolytic|lcl|cTer
FLPIIGKLLSGLL
>lcl|4046|lcl|HAPPENN|lcl|subdataset_8|lcl|hemolytic|lcl|cTer
LLKKLLKLLLKL
>lcl|4742|lcl|HAPPENN|lcl|subdataset_9|lcl|non-hemolytic|lcl|cTer
KKRWWWWWR
>lcl|4750|lcl|HAPPENN|lcl|subdataset_7|lcl|non-hemolytic
LRKRLRKFRNKIKEKLKKIGQKIQGFVPKL
>lcl|1358|lcl|HAPPENN&HAPPENN-RR90&HAPPENN-hard|lcl|subdataset_9|lcl|hemolytic|lcl|cTer
SAPRGCWTKSYPPKPCK
>lcl|247|lcl|HAPPENN&HAPPENN-RR90&HAPPENN-hard|lcl|subdataset_6|lcl|hemolytic
MQFITDLIKKAVDFFKGLFGNK
>lcl|3916|lcl|HAPPENN&HAPPENN-hard|lcl|subdataset_9|lcl|non-hemolytic|lcl|cTer
FRVTWRTKWWKG
>lcl|1275|lcl|HAPPENN|lcl|subdataset_9|lcl|hemolytic|lcl|cTer
PKVMAHMK
>lcl|1642|lcl|HAPPENN&HAPPENN-RR90&HAPPENN-hard|lcl|subdataset_11|lcl|hemolytic|lcl|cTer
FFPLVLGALGSILPKVFGK
>lcl|2103|lcl|HAPPENN&HAPPENN-RR90|lcl|subdataset_5|lcl|non-hemolytic
CVKGGKKYKRQGKGHRMRRYRNNH
>lcl|415|lcl|HAPPENN&HAPPENN-RR90&HAPPENN-hard|lcl|subdataset_7|lcl|non-hemolytic|lcl|cTer
KFLGTLVNLAKKIL
>lcl|2261|lcl|HAPPENN|lcl|subdataset_5|lcl|hemolytic
GLPVCGETCVGGTCYTPGCTCSWPVCTRN
>lcl|4569|lcl|HAPPENN&HAPPENN-RR90|lcl|subdataset_12|lcl|non-hemolytic
RMKLNAKKLSFC
>lcl|651|lcl|HAPPENN&HAPPENN-hard|lcl|subdataset_12|lcl|non-hemolytic
ILGKIWEGIKS
>lcl|1868|lcl|HAPPENN|lcl|subdataset_7|lcl|non-hemolytic|lcl|cTer
IKKIIKKIIKKI
>lcl|1383|lcl|HAPPENN&HAPPENN-RR90&HAPPENN-hard|lcl|subdataset_7|lcl|hemolytic
PKLLKTFLSKWKKIG
>lcl|2688|lcl|HAPPENN&HAPPENN-RR90&HAPPENN-hard|lcl|subdataset_6|lcl|non-hemolytic
LKIAKGIIKSL
>lcl|534|lcl|HAPPENN&HAPPENN-RR90&HAPPENN-hard|lcl|subdataset_4|lcl|hemolytic
FLSLALAALPKLFCLIFKKC
>lcl|1344|lcl|HAPPENN&HAPPENN-RR90&HAPPENN-hard|lcl|subdataset_6|lcl|hemolytic|lcl|cTer
LNWGAFLKHFFK
>lcl|2332|lcl|HAPPENN&HAPPENN-RR90|lcl|subdataset_5|lcl|non-hemolytic|lcl|nTer|lcl|cTer
KYTGKCTKSKNECK
>lcl|807|lcl|HAPPENN&HAPPENN-RR90&HAPPENN-hard|lcl|subdataset_4|lcl|hemolytic
CKLLLKWLLKLLKC
>lcl|4123|lcl|HAPPENN|lcl|subdataset_6|lcl|non-hemolytic
RKSKEKIGKEFKRIVQRIKDF
>lcl|1395|lcl|HAPPENN&HAPPENN-RR90&HAPPENN-hard|lcl|subdataset_10|lcl|hemolytic|lcl|cTer
FLPLVTMLLGKLF
>lcl|4419|lcl|HAPPENN&HAPPENN-hard|lcl|subdataset_6|lcl|non-hemolytic|lcl|cTer
AKRLKKLFKKIWNWK
>lcl|1360|lcl|HAPPENN&HAPPENN-hard|lcl|subdataset_11|lcl|non-hemolytic
GMASKAGSIVGKIKIALGAL
>lcl|845|lcl|HAPPENN|lcl|subdataset_6|lcl|non-hemolytic|lcl|cTer
LKKLLKKLLKW
>lcl|1064|lcl|HAPPENN&HAPPENN-RR90&HAPPENN-hard|lcl|subdataset_12|lcl|non-hemolytic
GKLNLFLSRLEILKLFVGAL
```

```
>lcl|3277|lcl|HAPPENN&HAPPENN-RR90&HAPPENN-hard|lcl|subdataset_8|lcl|non-hemolytic
FLSLIPSLVGGSISAFK
>lcl|1381|lcl|HAPPENN&HAPPENN-RR90&HAPPENN-hard|lcl|subdataset_6|lcl|hemolytic
LKLLLLKLLKLKLWK
>lcl|122|lcl|HAPPENN&HAPPENN-RR90|lcl|subdataset_8|lcl|non-hemolytic
RCYARRRFAVCR
>lcl|3755|lcl|HAPPENN|lcl|subdataset_11|lcl|hemolytic|lcl|cTer
ALWMTLLKKVLKA
>lcl|859|lcl|HAPPENN&HAPPENN-RR90&HAPPENN-hard|lcl|subdataset_12|lcl|hemolytic|lcl|cTer
KWAKKWKWFAKAAWKWYKK
>lcl|487|lcl|HAPPENN|lcl|subdataset_10|lcl|non-hemolytic
AKKVFKRLEKLFSKIQNDK
>lcl|629|lcl|HAPPENN|lcl|subdataset_5|lcl|non-hemolytic|lcl|cTer
KWFKKIPKFLHLAKKF
>lcl|4757|lcl|HAPPENN&HAPPENN-RR90&HAPPENN-hard|lcl|subdataset_10|lcl|hemolytic|lcl|cTer
FLGALWKVAKSVF
>lcl|2138|lcl|HAPPENN&HAPPENN-RR90|lcl|subdataset_11|lcl|non-hemolytic|lcl|cTer
GRKKRRQRRRGALWKSLLKNVGKA
>lcl|4836|lcl|HAPPENN&HAPPENN-RR90&HAPPENN-hard|lcl|subdataset_4|lcl|hemolytic|lcl|cTer
FFHHIFRGIVHVGKKIHRLVKG
>lcl|4217|lcl|HAPPENN&HAPPENN-RR90&HAPPENN-hard|lcl|subdataset_5|lcl|non-hemolytic
AVIPPLRCKAAFC
>lcl|2702|lcl|HAPPENN&HAPPENN-RR90&HAPPENN-hard|lcl|subdataset_7|lcl|hemolytic|lcl|cTer
FLSGIVGMLGKLF
>lcl|754|lcl|HAPPENN|lcl|subdataset_7|lcl|non-hemolytic
RLYRKVYG
>lcl|3665|lcl|HAPPENN|lcl|subdataset_11|lcl|non-hemolytic|lcl|cTer
GVVVRIGRVVVRGVRR
>lcl|3639|lcl|HAPPENN&HAPPENN-hard|lcl|subdataset_6|lcl|non-hemolytic
FKCWRWQWRWKKLGA
>lcl|1367|lcl|HAPPENN|lcl|subdataset_12|lcl|hemolytic|lcl|cTer
GFFALIAKIISSPLFKTL
>lcl|3388|lcl|HAPPENN&HAPPENN-RR90&HAPPENN-hard|lcl|subdataset_7|lcl|hemolytic|lcl|cTer
ILPWRWPWWPWRR
>lcl|1181|lcl|HAPPENN&HAPPENN-RR90&HAPPENN-hard|lcl|subdataset_7|lcl|hemolytic|lcl|cTer
ILGPVLGLVGNALGGLIKKI
>lcl|578|lcl|HAPPENN&HAPPENN-RR90&HAPPENN-hard|lcl|subdataset_7|lcl|hemolytic
LRRLWLRANRLLRRLWLRANRL
>lcl|952|lcl|HAPPENN|lcl|subdataset_6|lcl|hemolytic|lcl|cTer
FLPLIGRVLAGIL
>lcl|821|lcl|HAPPENN&HAPPENN-RR90&HAPPENN-hard|lcl|subdataset_10|lcl|non-hemolytic
FKKLKKIANIINSIFKK
>lcl|4705|lcl|HAPPENN|lcl|subdataset_4|lcl|non-hemolytic|lcl|nTer|lcl|cTer
KWRRAVRAI
>lcl|2007|lcl|HAPPENN&HAPPENN-RR90|lcl|subdataset_11|lcl|non-hemolytic|lcl|cTer
IKLRNVLKYLFRIDVIKEDIL
>lcl|1174|lcl|HAPPENN|lcl|subdataset_4|lcl|hemolytic
FFHHIFRGIVHVGKTIHKLVTG
>lcl|4535|lcl|HAPPENN|lcl|subdataset_9|lcl|hemolytic|lcl|cTer
FFHHIFRGAVHVGKTIHRLVTG
>lcl|4223|lcl|HAPPENN&HAPPENN-RR90&HAPPENN-hard|lcl|subdataset_11|lcl|non-hemolytic
LALERRSGWLRLFGLKPRRKH
>lcl|2251|lcl|HAPPENN|lcl|subdataset_5|lcl|non-hemolytic|lcl|cTer
HKKHKKHKKHKKHKKHKK
>lcl|858|lcl|HAPPENN&HAPPENN-RR90&HAPPENN-hard|lcl|subdataset_9|lcl|hemolytic|lcl|cTer
KWARLWRWFRITRWLWYIK
>lcl|1339|lcl|HAPPENN&HAPPENN-RR90&HAPPENN-hard|lcl|subdataset_5|lcl|hemolytic|lcl|cTer
LNWGAILKHIKK
>lcl|1652|lcl|HAPPENN&HAPPENN-RR90|lcl|subdataset_9|lcl|non-hemolytic
ILPSKLCRLLGNC
>lcl|4605|lcl|HAPPENN|lcl|subdataset_7|lcl|non-hemolytic|lcl|cTer
SGKRWWRRKK
>lcl|522|lcl|HAPPENN&HAPPENN-RR90&HAPPENN-hard|lcl|subdataset_5|lcl|hemolytic|lcl|cTer
AVGIGALFLGFLGAAGSTMGARS
>lcl|3178|lcl|HAPPENN|lcl|subdataset_4|lcl|hemolytic|lcl|cTer
FLFSLIPKAIGGLISAFK
```

```
>lcl|2051|lcl|HAPPENN&HAPPENN-RR90|lcl|subdataset_7|lcl|non-hemolytic
RGGCLCYCRRRFCVCVCR
>lcl|1223|lcl|HAPPENN&HAPPENN-RR90&HAPPENN-hard|lcl|subdataset_8|lcl|non-hemolytic|lcl|cTer
GLGKFLHSAKRFGKAFVGEAMNS
>lcl|1232|lcl|HAPPENN|lcl|subdataset_4|lcl|non-hemolytic
ALLHHGLNCAKGVLA
>lcl|113|lcl|HAPPENN&HAPPENN-RR90|lcl|subdataset_9|lcl|non-hemolytic
RGGRLCYARRRFAVCVGR
>lcl|4663|lcl|HAPPENN|lcl|subdataset_6|lcl|non-hemolytic|lcl|cTer
IRIKIRIK
>lcl|1447|lcl|HAPPENN&HAPPENN-hard|lcl|subdataset_8|lcl|non-hemolytic
SSLLEKGLDGAKKAVGGLGKLGKDA
>lcl|3648|lcl|HAPPENN|lcl|subdataset_5|lcl|non-hemolytic|lcl|cTer
GLWSKIKEVGKEAAKAAAKAAGK
>lcl|2395|lcl|HAPPENN&HAPPENN-RR90&HAPPENN-hard|lcl|subdataset_9|lcl|hemolytic|lcl|cTer
RRLIWLILRLLR
>lcl|130|lcl|HAPPENN&HAPPENN-RR90|lcl|subdataset_7|lcl|non-hemolytic
LCYTFRPRFVCV
>lcl|4321|lcl|HAPPENN|lcl|subdataset_4|lcl|hemolytic
FWGFLGKLAMKAVPSLIGGNKK
>lcl|557|lcl|HAPPENN&HAPPENN-hard|lcl|subdataset_11|lcl|non-hemolytic|lcl|cTer
LLRRLLRR
>lcl|686|lcl|HAPPENN&HAPPENN-RR90&HAPPENN-hard|lcl|subdataset_9|lcl|hemolytic|lcl|cTer
FFGWLIRGAIHAGKAIHGLIHRRRH
>lcl|1513|lcl|HAPPENN&HAPPENN-RR90&HAPPENN-hard|lcl|subdataset_7|lcl|hemolytic|lcl|cTer
FLPILGKLLSGIL
>lcl|1389|lcl|HAPPENN&HAPPENN-RR90&HAPPENN-hard|lcl|subdataset_10|lcl|hemolytic|lcl|cTer
GLKKLISWIKRAAQQG
>lcl|3420|lcl|HAPPENN&HAPPENN-hard|lcl|subdataset_8|lcl|non-hemolytic
KILRGVSKKIMRTFLRRISKDILTGKK
>lcl|3901|lcl|HAPPENN|lcl|subdataset_12|lcl|hemolytic|lcl|cTer
GIWKTIKSMGKVFAGKIKQNL
>lcl|2043|lcl|HAPPENN&HAPPENN-hard|lcl|subdataset_6|lcl|non-hemolytic|lcl|cTer
FKRFIGSVKHGIGHLVHHIGVAL
>lcl|3689|lcl|HAPPENN&HAPPENN-hard|lcl|subdataset_11|lcl|non-hemolytic
WLGALFKVASK
>lcl|3337|lcl|HAPPENN|lcl|subdataset_5|lcl|non-hemolytic|lcl|cTer
SGIQPEQHSS
>lcl|112|lcl|HAPPENN&HAPPENN-RR90|lcl|subdataset_6|lcl|non-hemolytic
RGGRLAYCRRRFCVAVGR
>lcl|3353|lcl|HAPPENN&HAPPENN-RR90&HAPPENN-hard|lcl|subdataset_4|lcl|non-hemolytic
KVVKQWPIGKVVKKVVKKVVK
>lcl|3310|lcl|HAPPENN&HAPPENN-RR90&HAPPENN-hard|lcl|subdataset_5|lcl|hemolytic|lcl|cTer
IKLSPETKKNLKKVLKGAIKGAIAVAKMV
>lcl|3219|lcl|HAPPENN&HAPPENN-RR90&HAPPENN-hard|lcl|subdataset_11|lcl|non-hemolytic|lcl|cTer
ILGPVIKTIGGVIGGLLKNL
>lcl|135|lcl|HAPPENN|lcl|subdataset_12|lcl|non-hemolytic
CYCFRRFCVC
>lcl|669|lcl|HAPPENN&HAPPENN-RR90|lcl|subdataset_11|lcl|non-hemolytic|lcl|cTer
KKKKKKAAAFAAAAAFAAWAAFAAA
>lcl|1200|lcl|HAPPENN&HAPPENN-RR90&HAPPENN-hard|lcl|subdataset_5|lcl|non-hemolytic|lcl|cTer
FLPLILRKIVTAL
>lcl|3205|lcl|HAPPENN&HAPPENN-RR90&HAPPENN-hard|lcl|subdataset_9|lcl|hemolytic
MAADIISTIGDLVKLIINTVKKFQK
>lcl|2408|lcl|HAPPENN&HAPPENN-RR90&HAPPENN-hard|lcl|subdataset_7|lcl|non-hemolytic
GKSNKPALTLIQARILKHKT
>lcl|2814|lcl|HAPPENN&HAPPENN-RR90|lcl|subdataset_11|lcl|non-hemolytic|lcl|cTer
LRAAHRLAIGRR
>lcl|2680|lcl|HAPPENN&HAPPENN-RR90&HAPPENN-hard|lcl|subdataset_9|lcl|non-hemolytic
GILDTLKEFGKTAAKGIAQSLLSTASCKLAKTC
>lcl|1466|lcl|HAPPENN&HAPPENN-RR90|lcl|subdataset_10|lcl|non-hemolytic|lcl|cTer
KAKLFKKIPKFLHLWKKF
>lcl|4649|lcl|HAPPENN|lcl|subdataset_5|lcl|hemolytic|lcl|nTer|lcl|cTer
IFGAIWPLALGALKNLIK
>lcl|3396|lcl|HAPPENN&HAPPENN-RR90|lcl|subdataset_11|lcl|non-hemolytic
KWCRCVCRRGICYCRCRG
```

```
>lcl|3000|lcl|HAPPENN|lcl|subdataset_4|lcl|hemolytic
ALWKNMLKGIGKLAGQAALGAVKTLVGAES
>lcl|3018|lcl|HAPPENN|lcl|subdataset_8|lcl|hemolytic|lcl|cTer
WSKILGHLIR
>lcl|81|lcl|HAPPENN|lcl|subdataset_8|lcl|hemolytic|lcl|cTer
FLPIVGKLLSGLL
>lcl|2994|lcl|HAPPENN&HAPPENN-RR90|lcl|subdataset_4|lcl|non-hemolytic|lcl|cTer
KIGKAIKWGKAIKIGKAI
>lcl|28|lcl|HAPPENN&HAPPENN-RR90|lcl|subdataset_8|lcl|non-hemolytic|lcl|cTer
ILPWKLPLLPLRR
>lcl|1636|lcl|HAPPENN&HAPPENN-RR90&HAPPENN-hard|lcl|subdataset_10|lcl|hemolytic|lcl|cTer
FLPFLAGLFGKIF
>lcl|2015|lcl|HAPPENN&HAPPENN-RR90|lcl|subdataset_12|lcl|non-hemolytic|lcl|cTer
GLYNFIKVLGRTVFGLYKQF
>lcl|2806|lcl|HAPPENN|lcl|subdataset_7|lcl|non-hemolytic|lcl|cTer
VVQGACRAIRHIPRRIR
>lcl|2730|lcl|HAPPENN&HAPPENN-RR90|lcl|subdataset_9|lcl|non-hemolytic
GANLAKKFYTYINKFINYAW
>lcl|4395|lcl|HAPPENN&HAPPENN-RR90|lcl|subdataset_6|lcl|non-hemolytic
LLRRVARLLKRFAK
>lcl|748|lcl|HAPPENN|lcl|subdataset_12|lcl|non-hemolytic
RLYRRLYR
>lcl|4571|lcl|HAPPENN|lcl|subdataset_4|lcl|hemolytic|lcl|cTer
LKLFKKILKVL
>lcl|4565|lcl|HAPPENN|lcl|subdataset_6|lcl|hemolytic|lcl|cTer
WKLFKKILKYL
>lcl|4472|lcl|HAPPENN&HAPPENN-RR90&HAPPENN-hard|lcl|subdataset_6|lcl|hemolytic|lcl|cTer
GILSSLLKKLKKIIAK
>lcl|1664|lcl|HAPPENN&HAPPENN-RR90&HAPPENN-hard|lcl|subdataset_9|lcl|non-hemolytic
NILSSIVNGINRALSFFG
>lcl|3746|lcl|HAPPENN|lcl|subdataset_9|lcl|hemolytic
AQWFAIQHISLNPPRSTIAMRAINNYRWR
>lcl|3120|lcl|HAPPENN&HAPPENN-RR90&HAPPENN-hard|lcl|subdataset_7|lcl|hemolytic
FLSTIWNGIKSLL
>lcl|790|lcl|HAPPENN|lcl|subdataset_4|lcl|hemolytic|lcl|cTer
GIGKFLHAAKKFAKAFVAEIMNS
>lcl|3082|lcl|HAPPENN&HAPPENN-RR90&HAPPENN-hard|lcl|subdataset_9|lcl|hemolytic|lcl|cTer
VNWKKILGKIIKKVVK
>lcl|111|lcl|HAPPENN|lcl|subdataset_4|lcl|hemolytic
RGGRLCYCRPRFCVCVGR
>lcl|1615|lcl|HAPPENN|lcl|subdataset_6|lcl|non-hemolytic|lcl|cTer
RRWHRHWRR
>lcl|1043|lcl|HAPPENN|lcl|subdataset_8|lcl|non-hemolytic|lcl|cTer
DVNDLKNLCAKTHNLLPMCAMF
>lcl|1060|lcl|HAPPENN|lcl|subdataset_8|lcl|non-hemolytic|lcl|cTer
LKKWLKKWLKK
>lcl|54|lcl|HAPPENN|lcl|subdataset_6|lcl|hemolytic|lcl|cTer
RWRWRWRWRW
>lcl|1244|lcl|HAPPENN&HAPPENN-RR90&HAPPENN-hard|lcl|subdataset_7|lcl|non-hemolytic|lcl|cTer
GIAKFGKAAAHFGKKWVGELMNS
>lcl|2151|lcl|HAPPENN|lcl|subdataset_4|lcl|non-hemolytic|lcl|cTer
FLKALKKLL
>lcl|2148|lcl|HAPPENN&HAPPENN-hard|lcl|subdataset_12|lcl|non-hemolytic|lcl|cTer
ALKLLKKLL
>lcl|2836|lcl|HAPPENN&HAPPENN-RR90&HAPPENN-hard|lcl|subdataset_6|lcl|hemolytic|lcl|cTer
SLSRFLSFLKIVYPPAF
>lcl|507|lcl|HAPPENN&HAPPENN-RR90&HAPPENN-hard|lcl|subdataset_12|lcl|hemolytic|lcl|cTer
GFKLLLKGAAKALVKTVLF
>lcl|1524|lcl|HAPPENN&HAPPENN-RR90&HAPPENN-hard|lcl|subdataset_12|lcl|non-hemolytic
GWASKIGQTLGKIAKVGLKELIQPK
>lcl|1795|lcl|HAPPENN|lcl|subdataset_9|lcl|hemolytic|lcl|cTer
WWRLRRIW
>lcl|1475|lcl|HAPPENN&HAPPENN-RR90&HAPPENN-hard|lcl|subdataset_4|lcl|hemolytic
CGESCVWIPCISSAIGCSCKNKVCYRNGIP
>lcl|3405|lcl|HAPPENN&HAPPENN-RR90&HAPPENN-hard|lcl|subdataset_12|lcl|non-hemolytic
CIAKGNGCQPSGVQGNCCSGHCHKEPGWVAGYCK
>lcl|1889|lcl|HAPPENN&HAPPENN-RR90&HAPPENN-hard|lcl|subdataset_11|lcl|hemolytic|lcl|cT
```

```
er
KLALKAALKAWKAAAKLA
>lcl|205|lcl|HAPPENN|lcl|subdataset_4|lcl|non-hemolytic
GIHDILKYGKPS
>lcl|3440|lcl|HAPPENN&HAPPENN-RR90|lcl|subdataset_7|lcl|non-hemolytic
YGAAKKAAKAAKKAAKAA
>lcl|444|lcl|HAPPENN|lcl|subdataset_4|lcl|non-hemolytic|lcl|nTer|lcl|cTer
KKLFKKILKFL
>lcl|1622|lcl|HAPPENN&HAPPENN-RR90&HAPPENN-hard|lcl|subdataset_10|lcl|hemolytic|lcl|cTer
KFWSLLKKALRLWANVL
>lcl|843|lcl|HAPPENN&HAPPENN-RR90|lcl|subdataset_9|lcl|non-hemolytic|lcl|cTer
KLLKKLLKWLK
>lcl|3502|lcl|HAPPENN|lcl|subdataset_8|lcl|non-hemolytic|lcl|cTer
AKRLKKLAKKIWKWK
>lcl|4394|lcl|HAPPENN&HAPPENN-RR90&HAPPENN-hard|lcl|subdataset_12|lcl|non-hemolytic
LLRRVAGLLKQFAK
>lcl|1700|lcl|HAPPENN&HAPPENN-RR90|lcl|subdataset_8|lcl|non-hemolytic|lcl|cTer
RRLFRRILRRL
>lcl|4596|lcl|HAPPENN&HAPPENN-RR90&HAPPENN-hard|lcl|subdataset_4|lcl|hemolytic
FLGGLIKIVPAMICAVTKKCHHHHHH
>lcl|3718|lcl|HAPPENN&HAPPENN-RR90&HAPPENN-hard|lcl|subdataset_8|lcl|hemolytic|lcl|nTer|lcl|cTer
GFWKKVGSAAWGGVKAAAKGAAVGGLNALAKHIQ
>lcl|140|lcl|HAPPENN|lcl|subdataset_5|lcl|hemolytic
YCYCRRRFCVCVGR
>lcl|4064|lcl|HAPPENN&HAPPENN-RR90&HAPPENN-hard|lcl|subdataset_4|lcl|hemolytic|lcl|cTer
GIGKFLKKAKKFGKAFVKILKK
>lcl|783|lcl|HAPPENN&HAPPENN-RR90&HAPPENN-hard|lcl|subdataset_12|lcl|hemolytic|lcl|cTer
GVVTDLLNTAGGLLGNLVGSLSG
>lcl|4287|lcl|HAPPENN&HAPPENN-RR90&HAPPENN-hard|lcl|subdataset_8|lcl|non-hemolytic|lcl|cTer
GVFDIIKDAGRQLVAHAMGKIAEKV
>lcl|1904|lcl|HAPPENN|lcl|subdataset_11|lcl|non-hemolytic
GKPKPYSPRPTSHPRPIRV
>lcl|1835|lcl|HAPPENN&HAPPENN-RR90&HAPPENN-hard|lcl|subdataset_5|lcl|hemolytic
FFGPLIKIATGVLPNLICKALGKC
>lcl|1813|lcl|HAPPENN&HAPPENN-RR90&HAPPENN-hard|lcl|subdataset_4|lcl|non-hemolytic|lcl|cTer
LVKLVAGIKKFLKWK
>lcl|182|lcl|HAPPENN|lcl|subdataset_4|lcl|hemolytic|lcl|nTer|lcl|cTer
KWKLFKKIGKVLKVL
>lcl|784|lcl|HAPPENN&HAPPENN-RR90&HAPPENN-hard|lcl|subdataset_4|lcl|hemolytic|lcl|cTer
GLVTGLLKTAGKLLGDLFGSLSG
>lcl|3397|lcl|HAPPENN|lcl|subdataset_9|lcl|non-hemolytic
KWCRCVCRRGICRCRCRG
>lcl|145|lcl|HAPPENN|lcl|subdataset_5|lcl|hemolytic
FCYCRRRFCVCVGR
>lcl|4227|lcl|HAPPENN&HAPPENN-RR90&HAPPENN-hard|lcl|subdataset_4|lcl|hemolytic
GLWDSIKNFGKTIALNVMDKIKCKIGGGCPP
>lcl|2215|lcl|HAPPENN&HAPPENN-hard|lcl|subdataset_6|lcl|non-hemolytic|lcl|cTer
LLCKALRKI
>lcl|2885|lcl|HAPPENN|lcl|subdataset_9|lcl|non-hemolytic
KLKKLFKLKQ
>lcl|16|lcl|HAPPENN|lcl|subdataset_12|lcl|hemolytic|lcl|cTer
ALWKTLLKKVLKAAA
>lcl|1063|lcl|HAPPENN&HAPPENN-RR90&HAPPENN-hard|lcl|subdataset_7|lcl|hemolytic|lcl|cTer
LLKWLLKWLLK
>lcl|1215|lcl|HAPPENN|lcl|subdataset_11|lcl|hemolytic|lcl|cTer
LKLNSIVSWAKKVL
>lcl|3841|lcl|HAPPENN|lcl|subdataset_8|lcl|hemolytic
GLKALKKVFKGIHKAIKLINKHVQ
>lcl|437|lcl|HAPPENN&HAPPENN-hard|lcl|subdataset_5|lcl|non-hemolytic
FKRLKKLFSKIWNWK
>lcl|194|lcl|HAPPENN&HAPPENN-RR90&HAPPENN-hard|lcl|subdataset_11|lcl|hemolytic
TQTVYEWCGVATQLLAAYILL
>lcl|2605|lcl|HAPPENN&HAPPENN-RR90&HAPPENN-hard|lcl|subdataset_12|lcl|hemolytic|lcl|cTer
FFPLLFGALSSHLPKLF
>lcl|1853|lcl|HAPPENN&HAPPENN-RR90|lcl|subdataset_5|lcl|non-hemolytic
```

```
RRRLCPIVIRVCRR
>lcl|2768|lcl|HAPPENN|lcl|subdataset_4|lcl|non-hemolytic|lcl|cTer
GLPALISWIKRKRL
>lcl|811|lcl|HAPPENN&HAPPENN-RR90&HAPPENN-hard|lcl|subdataset_4|lcl|hemolytic
LFGFLIPLLPHIIGAIPQVIGAIR
>lcl|2717|lcl|HAPPENN|lcl|subdataset_11|lcl|hemolytic
PRRRRSSSRP
>lcl|2784|lcl|HAPPENN&HAPPENN-RR90&HAPPENN-hard|lcl|subdataset_9|lcl|non-hemolytic
FLGALVKALSKLL
>lcl|2654|lcl|HAPPENN&HAPPENN-RR90&HAPPENN-hard|lcl|subdataset_8|lcl|hemolytic
FLSTALKVAANVVPTLFCKITKKC
>lcl|4528|lcl|HAPPENN|lcl|subdataset_11|lcl|non-hemolytic
AGYNLYRAIKKK
>lcl|1981|lcl|HAPPENN&HAPPENN-RR90&HAPPENN-hard|lcl|subdataset_6|lcl|hemolytic|lcl|cTer
FLFSLIPSAISGLISAFKGRRKRDLN
>lcl|4564|lcl|HAPPENN&HAPPENN-RR90|lcl|subdataset_9|lcl|non-hemolytic
GTKRGKLCRISRLAL
>lcl|3002|lcl|HAPPENN|lcl|subdataset_7|lcl|non-hemolytic|lcl|cTer
KIGAIKKWGAIKKIGAIK
>lcl|4558|lcl|HAPPENN|lcl|subdataset_4|lcl|hemolytic|lcl|cTer
WKLFKKILKFL
>lcl|1|lcl|HAPPENN|lcl|subdataset_6|lcl|hemolytic
ALWMTLLKKVLKAAAKAALDAVLVGANA
>lcl|3162|lcl|HAPPENN&HAPPENN-RR90&HAPPENN-hard|lcl|subdataset_11|lcl|hemolytic
GIKEFAHSLGKFGKAFVGGILNQ
>lcl|941|lcl|HAPPENN&HAPPENN-RR90&HAPPENN-hard|lcl|subdataset_4|lcl|hemolytic|lcl|cTer
FVRWFSKFLGRIL
>lcl|3204|lcl|HAPPENN&HAPPENN-RR90|lcl|subdataset_5|lcl|non-hemolytic
GLLGGLLGPLLGGGGGGGGGLL
>lcl|3269|lcl|HAPPENN&HAPPENN-RR90&HAPPENN-hard|lcl|subdataset_7|lcl|hemolytic
GDACGETCFTGICFTAGCSCNPWPTCTRN
>lcl|2540|lcl|HAPPENN&HAPPENN-hard|lcl|subdataset_9|lcl|non-hemolytic|lcl|cTer
PFKISIHL
>lcl|99|lcl|HAPPENN|lcl|subdataset_8|lcl|non-hemolytic|lcl|cTer
KKKKKKALFALWLAFAA
>lcl|2096|lcl|HAPPENN&HAPPENN-RR90&HAPPENN-hard|lcl|subdataset_10|lcl|hemolytic
GNPLKLFLPSTWVHFFKFLR
>lcl|2091|lcl|HAPPENN|lcl|subdataset_10|lcl|hemolytic|lcl|cTer
ALWKDILKNLLKAALNEINQIVQ
>lcl|4095|lcl|HAPPENN&HAPPENN-RR90|lcl|subdataset_6|lcl|non-hemolytic
SKVGRHGRRFGHRAHRKL
>lcl|2400|lcl|HAPPENN|lcl|subdataset_6|lcl|hemolytic|lcl|cTer
FLSLIPKIISAISALIKHF
>lcl|691|lcl|HAPPENN|lcl|subdataset_8|lcl|non-hemolytic
HVDKKVADKVLLLKQLRIMRLLTRL
>lcl|4651|lcl|HAPPENN|lcl|subdataset_8|lcl|non-hemolytic
RKWVAWRNR
>lcl|661|lcl|HAPPENN|lcl|subdataset_4|lcl|non-hemolytic
GSKKPVPIIYCNRRTGKCQRM
>lcl|4585|lcl|HAPPENN&HAPPENN-RR90|lcl|subdataset_4|lcl|non-hemolytic|lcl|cTer
GMWSKIKETAMAAAKEAAKAAGKTISDMIKQ
>lcl|3165|lcl|HAPPENN&HAPPENN-RR90&HAPPENN-hard|lcl|subdataset_9|lcl|hemolytic|lcl|cTer
GMASTAGSVLGKLAKAVAIGAL
>lcl|4623|lcl|HAPPENN|lcl|subdataset_10|lcl|non-hemolytic
GRRPRPRPRPFFF
>lcl|1675|lcl|HAPPENN&HAPPENN-hard|lcl|subdataset_12|lcl|non-hemolytic|lcl|cTer
FRRFFKWPRRFFKFF
>lcl|1780|lcl|HAPPENN&HAPPENN-RR90&HAPPENN-hard|lcl|subdataset_12|lcl|hemolytic
GCRRLCYKQRCVTWCRGR
>lcl|4251|lcl|HAPPENN|lcl|subdataset_10|lcl|hemolytic|lcl|cTer
FLFSLIPSVIAGLVSAIRN
>lcl|1371|lcl|HAPPENN&HAPPENN-RR90|lcl|subdataset_9|lcl|non-hemolytic
RQRVEELSKFSKKGAAARRRK
>lcl|5|lcl|HAPPENN|lcl|subdataset_11|lcl|hemolytic
ALWKTLLKKVLKAAAKAALNAVLVGANA
>lcl|4273|lcl|HAPPENN|lcl|subdataset_4|lcl|hemolytic
KWKLFKKIFKRIVQRIKDFLR
>lcl|755|lcl|HAPPENN|lcl|subdataset_8|lcl|non-hemolytic
RLYRKVYGRLYRKVYGK
>lcl|4792|lcl|HAPPENN&HAPPENN-RR90&HAPPENN-hard|lcl|subdataset_4|lcl|hemolytic|lcl|cTer
```

```
KKKALFALWLAFLAKKK
>lcl|1055|lcl|HAPPENN&HAPPENN-hard|lcl|subdataset_6|lcl|non-hemolytic|lcl|cTer
KWLLKWL
>lcl|4805|lcl|HAPPENN&HAPPENN-RR90|lcl|subdataset_6|lcl|non-hemolytic
GKYGFYTHVFRLKKWMQKVIDRFGG
>lcl|1808|lcl|HAPPENN&HAPPENN-RR90&HAPPENN-hard|lcl|subdataset_7|lcl|hemolytic|lcl|cTer
GVFRVLRKVTRVVLKVIGKVLKWI
>lcl|4586|lcl|HAPPENN&HAPPENN-RR90&HAPPENN-hard|lcl|subdataset_9|lcl|non-hemolytic
FLGALFKVASKVLPSVFCAITKKC
>lcl|2998|lcl|HAPPENN&HAPPENN-RR90&HAPPENN-hard|lcl|subdataset_10|lcl|hemolytic
ALWKTLLKNVGKAAGKAALNAVTDMVNQ
>lcl|3797|lcl|HAPPENN&HAPPENN-RR90&HAPPENN-hard|lcl|subdataset_6|lcl|non-hemolytic|lcl|cTer
KRIVKLIKKWLR
>lcl|4003|lcl|HAPPENN&HAPPENN-RR90|lcl|subdataset_7|lcl|non-hemolytic
VAIALKAAHYHTHKE
>lcl|2911|lcl|HAPPENN|lcl|subdataset_11|lcl|non-hemolytic
LLKKKFLKKQ
>lcl|1548|lcl|HAPPENN&HAPPENN-RR90|lcl|subdataset_7|lcl|non-hemolytic|lcl|cTer
RFRRLFRIRVRVLKKI
>lcl|4087|lcl|HAPPENN&HAPPENN-RR90|lcl|subdataset_4|lcl|non-hemolytic|lcl|cTer
VIHRAGLQFPVGRVHRLLRK
>lcl|2887|lcl|HAPPENN|lcl|subdataset_8|lcl|non-hemolytic
KKLLKFKLKQ
>lcl|3041|lcl|HAPPENN&HAPPENN-RR90&HAPPENN-hard|lcl|subdataset_4|lcl|hemolytic|lcl|cTer
GKWKSLLKHILK
>lcl|659|lcl|HAPPENN&HAPPENN-RR90|lcl|subdataset_7|lcl|non-hemolytic
VRRFPFFFPFLRR
>lcl|2839|lcl|HAPPENN&HAPPENN-RR90&HAPPENN-hard|lcl|subdataset_9|lcl|hemolytic
INWLKLGKKMMSAL
>lcl|511|lcl|HAPPENN&HAPPENN-RR90&HAPPENN-hard|lcl|subdataset_8|lcl|hemolytic|lcl|cTer
GFKDLLKGAAKALVKAVLF
>lcl|4823|lcl|HAPPENN&HAPPENN-hard|lcl|subdataset_6|lcl|non-hemolytic
FLFSLIPSAIGGLISAFK
>lcl|259|lcl|HAPPENN|lcl|subdataset_5|lcl|hemolytic|lcl|cTer
ILPLKLPLLPLRR
>lcl|3927|lcl|HAPPENN&HAPPENN-RR90&HAPPENN-hard|lcl|subdataset_6|lcl|non-hemolytic|lcl|cTer
RFRRLRWKTRKRLWKI
>lcl|3349|lcl|HAPPENN&HAPPENN-RR90|lcl|subdataset_6|lcl|non-hemolytic
ACQFWSCNSSCISRGYRQGYCWGIQYKYCQCQ
>lcl|4438|lcl|HAPPENN&HAPPENN-RR90|lcl|subdataset_9|lcl|non-hemolytic|lcl|cTer
WLNALLHHGYKRKFH
>lcl|3252|lcl|HAPPENN&HAPPENN-RR90&HAPPENN-hard|lcl|subdataset_11|lcl|hemolytic
FFPIIAGMAAKVICAITKKC
>lcl|2094|lcl|HAPPENN|lcl|subdataset_8|lcl|non-hemolytic
GNNRPVYIPQPRPPHPRL
>lcl|3136|lcl|HAPPENN&HAPPENN-RR90|lcl|subdataset_5|lcl|non-hemolytic
VAIYGRDDRSDVCRQVQHNWLVCDTY
>lcl|1986|lcl|HAPPENN&HAPPENN-hard|lcl|subdataset_7|lcl|non-hemolytic
NVLKKLNRLKEKNKAKNSKENN
>lcl|3584|lcl|HAPPENN&HAPPENN-RR90|lcl|subdataset_10|lcl|non-hemolytic
FWGLKGLKKFSKKL
>lcl|3479|lcl|HAPPENN|lcl|subdataset_10|lcl|non-hemolytic
VDIHVWSGV
>lcl|4096|lcl|HAPPENN|lcl|subdataset_11|lcl|non-hemolytic
SKVGRHLRRFGHRAHRKL
>lcl|662|lcl|HAPPENN|lcl|subdataset_10|lcl|non-hemolytic
GSKKPVPIIYCNRRGKCQRM
>lcl|1452|lcl|HAPPENN&HAPPENN-RR90&HAPPENN-hard|lcl|subdataset_11|lcl|non-hemolytic
GLLSGILGAGKHIVCGLTGCAKA
>lcl|2374|lcl|HAPPENN|lcl|subdataset_12|lcl|non-hemolytic|lcl|cTer
RWRWWWRWR
>lcl|2421|lcl|HAPPENN&HAPPENN-RR90|lcl|subdataset_12|lcl|non-hemolytic
RICTAIPLPMCL
>lcl|1090|lcl|HAPPENN&HAPPENN-RR90&HAPPENN-hard|lcl|subdataset_5|lcl|hemolytic
GFLGPLLKLGLKGVAKVIPHLIPSRQQ
>lcl|1419|lcl|HAPPENN|lcl|subdataset_9|lcl|hemolytic|lcl|nTer|lcl|cTer
INWLKLGKKMMSAL
>lcl|3790|lcl|HAPPENN|lcl|subdataset_5|lcl|non-hemolytic|lcl|nTer|lcl|cTer
GIRRILKYGKRS
```

```
>lcl|4589|lcl|HAPPENN|lcl|subdataset_7|lcl|non-hemolytic
RGSALTHLP
>lcl|2974|lcl|HAPPENN|lcl|subdataset_4|lcl|non-hemolytic|lcl|cTer
GLLKKIKTLL
>lcl|4016|lcl|HAPPENN|lcl|subdataset_8|lcl|hemolytic|lcl|cTer
GLLKFIKWLL
>lcl|339|lcl|HAPPENN&HAPPENN-RR90&HAPPENN-hard|lcl|subdataset_7|lcl|hemolytic
FLPILAGLAANILPKVFCSITKKC
>lcl|4716|lcl|HAPPENN&HAPPENN-RR90&HAPPENN-hard|lcl|subdataset_4|lcl|hemolytic|lcl|cTer
FPVTWGWWKWWKG
>lcl|1249|lcl|HAPPENN&HAPPENN-RR90&HAPPENN-hard|lcl|subdataset_10|lcl|hemolytic
KWKSFIKKLTSAAKKVVTTAKPLISS
>lcl|3676|lcl|HAPPENN&HAPPENN-RR90&HAPPENN-hard|lcl|subdataset_9|lcl|non-hemolytic
RRRSVGQQNAIPSHIQVNKFFLRKPAKQHI
>lcl|2050|lcl|HAPPENN&HAPPENN-RR90|lcl|subdataset_9|lcl|non-hemolytic|lcl|cTer
GLFRRLRDSIRRGQQKILEKARRIGERIKDIFR
>lcl|899|lcl|HAPPENN&HAPPENN-RR90|lcl|subdataset_6|lcl|non-hemolytic|lcl|cTer
KWKSFIKKLTSKFLHSANKF
>lcl|4037|lcl|HAPPENN&HAPPENN-RR90|lcl|subdataset_9|lcl|non-hemolytic
EWFKARRWGWRMKKLQA
>lcl|3358|lcl|HAPPENN|lcl|subdataset_5|lcl|non-hemolytic|lcl|nTer|lcl|cTer
KKAAHVGKHVGKAALTHYL
>lcl|1839|lcl|HAPPENN&HAPPENN-RR90&HAPPENN-hard|lcl|subdataset_9|lcl|hemolytic
NPEKALEPLIAIQIAIKGMLNGWFTGVGFRRKR
>lcl|3595|lcl|HAPPENN&HAPPENN-hard|lcl|subdataset_8|lcl|non-hemolytic
LVQRGRFGRFLRKI
>lcl|2035|lcl|HAPPENN&HAPPENN-RR90|lcl|subdataset_9|lcl|non-hemolytic
GSAQPYKQLHKVVNWDPYG
>lcl|3258|lcl|HAPPENN&HAPPENN-RR90&HAPPENN-hard|lcl|subdataset_8|lcl|hemolytic
GLMDTIKGVAKTVAASWLDKLKCKITGC
>lcl|4753|lcl|HAPPENN&HAPPENN-RR90&HAPPENN-hard|lcl|subdataset_10|lcl|non-hemolytic|lcl|cTer
FLSLIPHAINAVGVHAKHF
>lcl|3193|lcl|HAPPENN|lcl|subdataset_10|lcl|hemolytic
IIGAIAAALPHVINAIKNTF
>lcl|3139|lcl|HAPPENN&HAPPENN-RR90|lcl|subdataset_12|lcl|non-hemolytic
VVKCSYRQGSPDSR
>lcl|751|lcl|HAPPENN|lcl|subdataset_6|lcl|non-hemolytic
RLYRRLYRK
>lcl|3840|lcl|HAPPENN&HAPPENN-hard|lcl|subdataset_12|lcl|non-hemolytic
GLKALKKVFKGIHKAIKLINNHVQ
>lcl|2896|lcl|HAPPENN|lcl|subdataset_4|lcl|non-hemolytic
LLKKKFKLKQ
>lcl|3078|lcl|HAPPENN|lcl|subdataset_12|lcl|hemolytic|lcl|cTer
VNWKKILPKIIKVAK
>lcl|936|lcl|HAPPENN|lcl|subdataset_9|lcl|hemolytic|lcl|cTer
FVPWFSKFLGRIL
>lcl|1487|lcl|HAPPENN&HAPPENN-RR90&HAPPENN-hard|lcl|subdataset_8|lcl|hemolytic
CGESCVFIPCVTALLGCSCKSKVCYKNSIP
>lcl|1101|lcl|HAPPENN|lcl|subdataset_7|lcl|hemolytic|lcl|cTer
ILGKLLSTAAGLLSNL
>lcl|460|lcl|HAPPENN&HAPPENN-RR90|lcl|subdataset_4|lcl|non-hemolytic
FKCKKVVISLRRY
>lcl|2072|lcl|HAPPENN&HAPPENN-RR90&HAPPENN-hard|lcl|subdataset_7|lcl|non-hemolytic
KFKKLFKKLSPVIGKEFKRIVERIKRFLR
>lcl|2915|lcl|HAPPENN|lcl|subdataset_11|lcl|non-hemolytic
KLLKLFKKKQ
>lcl|451|lcl|HAPPENN|lcl|subdataset_5|lcl|hemolytic
FFGWLIKGAIHAGKAIHGLIHRRRH
>lcl|1454|lcl|HAPPENN|lcl|subdataset_7|lcl|non-hemolytic|lcl|cTer
KWKKPLLKKLLKKL
>lcl|1007|lcl|HAPPENN&HAPPENN-RR90|lcl|subdataset_12|lcl|non-hemolytic
GRPNPVNNKPTPHPRL
>lcl|3278|lcl|HAPPENN&HAPPENN-RR90&HAPPENN-hard|lcl|subdataset_11|lcl|hemolytic
FLGMIPGLIGGLISAFK
>lcl|2184|lcl|HAPPENN|lcl|subdataset_4|lcl|hemolytic
KWKLFKKIGKVLKVL
>lcl|1337|lcl|HAPPENN&HAPPENN-RR90&HAPPENN-hard|lcl|subdataset_4|lcl|hemolytic|lcl|cTer
LNWGAILKKIIK
>lcl|3677|lcl|HAPPENN&HAPPENN-RR90|lcl|subdataset_5|lcl|non-hemolytic|lcl|cTer
INLKAIAALAKKLF
```

```
>lcl|2440|lcl|HAPPENN&HAPPENN-RR90&HAPPENN-hard|lcl|subdataset_8|lcl|non-hemolytic|lcl|cTer
RGWRRWGRKWAHGWKKYG
>lcl|1881|lcl|HAPPENN&HAPPENN-RR90&HAPPENN-hard|lcl|subdataset_8|lcl|non-hemolytic
HLRRINKLLTRIGLYRHAFG
>lcl|3031|lcl|HAPPENN&HAPPENN-RR90&HAPPENN-hard|lcl|subdataset_10|lcl|hemolytic|lcl|cTer
GKWKKILGKLIR
>lcl|1537|lcl|HAPPENN|lcl|subdataset_11|lcl|non-hemolytic|lcl|cTer
IKQVKKLFKK
>lcl|354|lcl|HAPPENN|lcl|subdataset_4|lcl|hemolytic|lcl|cTer
GLGSVFGRLARILGRVIPKV
>lcl|1650|lcl|HAPPENN&HAPPENN-RR90|lcl|subdataset_12|lcl|non-hemolytic
FFPLIPGVRCKILRTC
>lcl|4526|lcl|HAPPENN|lcl|subdataset_10|lcl|non-hemolytic
VVAGYNLYRAIKKK
>lcl|1301|lcl|HAPPENN&HAPPENN-RR90&HAPPENN-hard|lcl|subdataset_6|lcl|hemolytic|lcl|cTer
ILAWKWAWWAWRR
>lcl|3183|lcl|HAPPENN&HAPPENN-RR90&HAPPENN-hard|lcl|subdataset_7|lcl|non-hemolytic|lcl|cTer
LLGMIPLAISAISSLSKL
>lcl|1856|lcl|HAPPENN&HAPPENN-RR90&HAPPENN-hard|lcl|subdataset_5|lcl|hemolytic|lcl|cTer
FFSMIPKIAGGIASLVKNL
>lcl|1684|lcl|HAPPENN&HAPPENN-hard|lcl|subdataset_10|lcl|non-hemolytic
GLNALKKVFQPIHEAIKLINNHVQ
>lcl|785|lcl|HAPPENN|lcl|subdataset_4|lcl|hemolytic|lcl|cTer
GVVTDLLKTAGKLLGNLVGSLSG
>lcl|3777|lcl|HAPPENN&HAPPENN-hard|lcl|subdataset_12|lcl|non-hemolytic|lcl|cTer
GIGKHVGKALKGLKGLLKGLGEC
>lcl|2214|lcl|HAPPENN&HAPPENN-RR90|lcl|subdataset_6|lcl|non-hemolytic
LRLKKYKVPQL
>lcl|1532|lcl|HAPPENN&HAPPENN-RR90&HAPPENN-hard|lcl|subdataset_6|lcl|non-hemolytic
AKKVFKRLGIGAVLKVLKKG
>lcl|3483|lcl|HAPPENN&HAPPENN-RR90|lcl|subdataset_8|lcl|non-hemolytic
IKSIASKVANTVQKLKRKAKNAVA
>lcl|1420|lcl|HAPPENN|lcl|subdataset_5|lcl|hemolytic|lcl|cTer
INWKKIASIGKEVLKAL
>lcl|1982|lcl|HAPPENN|lcl|subdataset_8|lcl|hemolytic|lcl|cTer
FLFSLIPSAISGLINAFK
>lcl|241|lcl|HAPPENN|lcl|subdataset_7|lcl|hemolytic|lcl|cTer
GKMKEYFKKFGASFKRRFANLKKRL
>lcl|1376|lcl|HAPPENN&HAPPENN-hard|lcl|subdataset_9|lcl|non-hemolytic
AVVKVPLKKFKSIRE
>lcl|4741|lcl|HAPPENN|lcl|subdataset_7|lcl|non-hemolytic|lcl|cTer
RWKKWWRWL
>lcl|2996|lcl|HAPPENN&HAPPENN-RR90|lcl|subdataset_5|lcl|non-hemolytic|lcl|cTer
KIGAKIKWGAKIKIGAKI
>lcl|4325|lcl|HAPPENN|lcl|subdataset_8|lcl|hemolytic|lcl|cTer
FLGLIGSLL
>lcl|4834|lcl|HAPPENN|lcl|subdataset_10|lcl|non-hemolytic
GLFKKLRRKIKKGFKKIFKRL
>lcl|1913|lcl|HAPPENN&HAPPENN-RR90|lcl|subdataset_6|lcl|non-hemolytic
IHRDQQHESFLDARPEPGLTE
>lcl|4326|lcl|HAPPENN&HAPPENN-RR90&HAPPENN-hard|lcl|subdataset_5|lcl|non-hemolytic|lcl|cTer
AIHDILKYGKPS
>lcl|1807|lcl|HAPPENN&HAPPENN-RR90&HAPPENN-hard|lcl|subdataset_9|lcl|hemolytic|lcl|cTer
GVFRRLRKVTRKVLKKIGKVLKWI
>lcl|641|lcl|HAPPENN|lcl|subdataset_10|lcl|non-hemolytic|lcl|cTer
KWKLFKKIPLAKKF
>lcl|1080|lcl|HAPPENN&HAPPENN-RR90|lcl|subdataset_9|lcl|non-hemolytic
VDIHVWDGVVDIHVWDGV
>lcl|4107|lcl|HAPPENN|lcl|subdataset_7|lcl|hemolytic|lcl|cTer
RFRRLRKKIRKRLKKI
>lcl|3410|lcl|HAPPENN|lcl|subdataset_6|lcl|hemolytic|lcl|cTer
HFLGTLVKLAKKIL
>lcl|537|lcl|HAPPENN|lcl|subdataset_9|lcl|hemolytic|lcl|cTer
FVDLKKIANIINSIF
>lcl|1877|lcl|HAPPENN&HAPPENN-RR90|lcl|subdataset_9|lcl|non-hemolytic
RLCRIVPVIRVCR
```

```
>lcl|3627|lcl|HAPPENN&HAPPENN-RR90&HAPPENN-hard|lcl|subdataset_6|lcl|non-hemolytic|lcl|cTer
FRFKRFRKKGKKLFKKVSPPILLLHL
>lcl|749|lcl|HAPPENN|lcl|subdataset_12|lcl|non-hemolytic
RLYRRLYRRLYRRLYR
>lcl|4811|lcl|HAPPENN|lcl|subdataset_12|lcl|hemolytic
KKYRYHLKPFCKK
>lcl|4405|lcl|HAPPENN&HAPPENN-RR90&HAPPENN-hard|lcl|subdataset_4|lcl|hemolytic|lcl|cTer
RQIKIWFQNRRLKWKK
>lcl|2507|lcl|HAPPENN&HAPPENN-RR90|lcl|subdataset_7|lcl|non-hemolytic|lcl|cTer
KKLLGWGLLKK
>lcl|128|lcl|HAPPENN&HAPPENN-RR90|lcl|subdataset_6|lcl|non-hemolytic
LTYCRRRFCVTV
>lcl|1911|lcl|HAPPENN&HAPPENN-RR90|lcl|subdataset_11|lcl|non-hemolytic
YYNPLPHDCGRDNNTDICSR
>lcl|3157|lcl|HAPPENN&HAPPENN-RR90&HAPPENN-hard|lcl|subdataset_5|lcl|hemolytic
GVGKFLHSAKKFGQALVSEIMKS
>lcl|2832|lcl|HAPPENN&HAPPENN-RR90&HAPPENN-hard|lcl|subdataset_11|lcl|hemolytic|lcl|cTer
YKLLKKLLKKLKKLLKKL
>lcl|9|lcl|HAPPENN|lcl|subdataset_4|lcl|hemolytic
ALWMTLLKKVLK
>lcl|1897|lcl|HAPPENN&HAPPENN-RR90&HAPPENN-hard|lcl|subdataset_7|lcl|non-hemolytic
RRGFSLKLALAKDGWALMLRLGYGRR
>lcl|879|lcl|HAPPENN|lcl|subdataset_8|lcl|hemolytic
GIGAILKVLATGLPTLISWI
>lcl|3924|lcl|HAPPENN&HAPPENN-RR90|lcl|subdataset_5|lcl|non-hemolytic|lcl|cTer
RFRRLRWWTRKRLKKI
>lcl|2536|lcl|HAPPENN&HAPPENN-RR90&HAPPENN-hard|lcl|subdataset_6|lcl|non-hemolytic
LKKWWKTSKGLLGGLLGKVTSVIK
>lcl|2462|lcl|HAPPENN&HAPPENN-hard|lcl|subdataset_8|lcl|non-hemolytic|lcl|cTer
INWLAIAAAVAGML
>lcl|4248|lcl|HAPPENN|lcl|subdataset_12|lcl|non-hemolytic|lcl|cTer
GIGKFLKKAKKF
>lcl|3586|lcl|HAPPENN&HAPPENN-RR90|lcl|subdataset_10|lcl|non-hemolytic|lcl|cTer
RKDVYRRRRRR
>lcl|377|lcl|HAPPENN&HAPPENN-RR90&HAPPENN-hard|lcl|subdataset_4|lcl|hemolytic
GVLGTVKNLLIGAGKSAAQSVLKTLSCKLSNDC
>lcl|4575|lcl|HAPPENN|lcl|subdataset_6|lcl|non-hemolytic
VWRHWRRFWH
>lcl|829|lcl|HAPPENN|lcl|subdataset_7|lcl|non-hemolytic
KKSYVRRWRS
>lcl|3971|lcl|HAPPENN&HAPPENN-hard|lcl|subdataset_8|lcl|non-hemolytic|lcl|cTer
GVVDILKGAAKDIAGHLASAVMNKL
>lcl|677|lcl|HAPPENN&HAPPENN-RR90|lcl|subdataset_12|lcl|non-hemolytic|lcl|cTer
KKKKKKAAFAAFAAFAA
>lcl|4163|lcl|HAPPENN|lcl|subdataset_12|lcl|hemolytic
KKLFKKILKYLAGPAKKLFKKILKYLKDEL
>lcl|4237|lcl|HAPPENN|lcl|subdataset_8|lcl|hemolytic
LLSLALAALPKLFCLIFKKC
>lcl|2480|lcl|HAPPENN&HAPPENN-RR90|lcl|subdataset_9|lcl|non-hemolytic
AVKKLLRWWSRWWKK
>lcl|4206|lcl|HAPPENN|lcl|subdataset_4|lcl|hemolytic
FLPGLIKAAVGIGSTIFCKISRKC
>lcl|1455|lcl|HAPPENN|lcl|subdataset_6|lcl|non-hemolytic
KKTWWKTWWTKWSQPKKKRKV
>lcl|1974|lcl|HAPPENN&HAPPENN-RR90&HAPPENN-hard|lcl|subdataset_5|lcl|non-hemolytic|lcl|cTer
GKEFKRIVKWPWWPWRR
>lcl|2170|lcl|HAPPENN&HAPPENN-RR90&HAPPENN-hard|lcl|subdataset_7|lcl|hemolytic|lcl|cTer
GIKKWVKGVAKGVAKDLAKKIL
>lcl|3362|lcl|HAPPENN|lcl|subdataset_12|lcl|hemolytic|lcl|cTer
ILPWKWPWWPW
>lcl|1426|lcl|HAPPENN|lcl|subdataset_8|lcl|hemolytic|lcl|cTer
INWKKIASIGKEVLKA
>lcl|4196|lcl|HAPPENN&HAPPENN-RR90&HAPPENN-hard|lcl|subdataset_7|lcl|non-hemolytic
AVAVVGQATQIAKKKLFKKILKYLKDEL
>lcl|3350|lcl|HAPPENN|lcl|subdataset_11|lcl|non-hemolytic
RAGLQWPIGRLLRRLLRRLLR
>lcl|975|lcl|HAPPENN&HAPPENN-RR90&HAPPENN-hard|lcl|subdataset_7|lcl|hemolytic
GWKKWLRKGAKHLGQAAIKGLAS
```

```
>lcl|2024|lcl|HAPPENN&HAPPENN-RR90|lcl|subdataset_11|lcl|non-hemolytic|lcl|cTer
IARRNLCASLRARHTIPQCKKFGRR
>lcl|638|lcl|HAPPENN&HAPPENN-hard|lcl|subdataset_10|lcl|non-hemolytic|lcl|cTer
KWKLFKKIPKFLHLAK
>lcl|290|lcl|HAPPENN|lcl|subdataset_7|lcl|hemolytic
PAIAQRATATLGTVGSNTSGTTEIEACILL
>lcl|2699|lcl|HAPPENN&HAPPENN-RR90|lcl|subdataset_7|lcl|non-hemolytic
GSNTGFNFKTLDKE
>lcl|851|lcl|HAPPENN&HAPPENN-RR90&HAPPENN-hard|lcl|subdataset_5|lcl|hemolytic
FIHHIIGGLFSAGKAIHRLIRRRRR
>lcl|50|lcl|HAPPENN&HAPPENN-RR90&HAPPENN-hard|lcl|subdataset_4|lcl|non-hemolytic
GWANTLKNVAGGLCKITGAA
>lcl|3080|lcl|HAPPENN|lcl|subdataset_11|lcl|hemolytic|lcl|cTer
NVWKKILGKIIKVVK
>lcl|3147|lcl|HAPPENN&HAPPENN-RR90|lcl|subdataset_11|lcl|non-hemolytic
AAPRGGKGFFCKLFKDC
>lcl|1205|lcl|HAPPENN|lcl|subdataset_11|lcl|hemolytic|lcl|cTer
FVGAIAAALPHVISAIKNAL
>lcl|4094|lcl|HAPPENN&HAPPENN-RR90|lcl|subdataset_8|lcl|non-hemolytic
SAVLRHLRRFLLRKHRKH
>lcl|766|lcl|HAPPENN|lcl|subdataset_5|lcl|non-hemolytic
SWLSKTAKKLENSAKKRISEGIAIAIQGGPR
>lcl|4760|lcl|HAPPENN&HAPPENN-RR90&HAPPENN-hard|lcl|subdataset_12|lcl|hemolytic|lcl|cTer
FLGALWEVAKSVF
>lcl|452|lcl|HAPPENN|lcl|subdataset_5|lcl|non-hemolytic|lcl|cTer
RLRLRIGRR
>lcl|1408|lcl|HAPPENN&HAPPENN-RR90&HAPPENN-hard|lcl|subdataset_5|lcl|hemolytic|lcl|cTer
NIWKKIFEKVKNLV
>lcl|3210|lcl|HAPPENN&HAPPENN-RR90&HAPPENN-hard|lcl|subdataset_5|lcl|non-hemolytic|lcl|cTer
GIGTKFLGGVKTALKGALKELAFTYVN
>lcl|1062|lcl|HAPPENN|lcl|subdataset_4|lcl|hemolytic|lcl|cTer
LLKWLLKWLKK
>lcl|4593|lcl|HAPPENN&HAPPENN-hard|lcl|subdataset_10|lcl|non-hemolytic|lcl|cTer
CLLKKLLKK
>lcl|4616|lcl|HAPPENN|lcl|subdataset_4|lcl|non-hemolytic
GRRPRPRPRP
>lcl|201|lcl|HAPPENN&HAPPENN-RR90&HAPPENN-hard|lcl|subdataset_9|lcl|hemolytic
FWGALAKGALKLIGVGSLFSSFSKKD
>lcl|2875|lcl|HAPPENN|lcl|subdataset_9|lcl|non-hemolytic
KKLKLFKKLQ
>lcl|2665|lcl|HAPPENN&HAPPENN-hard|lcl|subdataset_10|lcl|non-hemolytic|lcl|cTer
ALRLAIRKR
>lcl|2227|lcl|HAPPENN&HAPPENN-RR90|lcl|subdataset_9|lcl|non-hemolytic|lcl|cTer
IHKFWRGGRWFKHI
>lcl|2134|lcl|HAPPENN&HAPPENN-RR90|lcl|subdataset_6|lcl|non-hemolytic
KCRRRKVHGPMIRIRKK
>lcl|3972|lcl|HAPPENN|lcl|subdataset_7|lcl|non-hemolytic|lcl|cTer
GVVDILKGAAKDIAGHLAAKVMNKL
>lcl|3618|lcl|HAPPENN|lcl|subdataset_4|lcl|hemolytic|lcl|cTer
RLWLAIKRR
>lcl|3028|lcl|HAPPENN|lcl|subdataset_6|lcl|hemolytic|lcl|cTer
GMWKKILGKLIR
>lcl|1153|lcl|HAPPENN&HAPPENN-RR90&HAPPENN-hard|lcl|subdataset_9|lcl|non-hemolytic|lcl|nTer
QQRKRKGIGAVLKVLTTGLPALISWI
>lcl|90|lcl|HAPPENN|lcl|subdataset_4|lcl|non-hemolytic|lcl|nTer|lcl|cTer
KWKSFLKTFKSAKKKVLHTALKAISS
>lcl|340|lcl|HAPPENN&HAPPENN-RR90&HAPPENN-hard|lcl|subdataset_12|lcl|hemolytic|lcl|cTer
FLPIIGQLLSGLL
>lcl|2835|lcl|HAPPENN&HAPPENN-RR90&HAPPENN-hard|lcl|subdataset_7|lcl|hemolytic|lcl|cTer
FIITGLVRGLTKLF
>lcl|1346|lcl|HAPPENN&HAPPENN-RR90&HAPPENN-hard|lcl|subdataset_11|lcl|hemolytic|lcl|cTer
LNWGALLKHLLK
>lcl|4754|lcl|HAPPENN&HAPPENN-hard|lcl|subdataset_10|lcl|non-hemolytic|lcl|cTer
FLSLIPHAIKAVGVHAKHF
>lcl|1873|lcl|HAPPENN&HAPPENN-RR90|lcl|subdataset_11|lcl|non-hemolytic|lcl|cTer
RLCPRVRIRVCR
```

```
>lcl|223|lcl|HAPPENN|lcl|subdataset_8|lcl|non-hemolytic|lcl|cTer
LKKWLKK
>lcl|2512|lcl|HAPPENN&HAPPENN-RR90|lcl|subdataset_6|lcl|non-hemolytic|lcl|cTer
KLLGKWKGLLK
>lcl|3536|lcl|HAPPENN&HAPPENN-RR90&HAPPENN-hard|lcl|subdataset_4|lcl|non-hemolytic
GFLNTAMNTVTNLAGTLMDKAKCKIRGC
>lcl|2477|lcl|HAPPENN&HAPPENN-RR90&HAPPENN-hard|lcl|subdataset_10|lcl|hemolytic|lcl|cTer
GIWKKWIKKVVNVLKNLF
>lcl|4550|lcl|HAPPENN|lcl|subdataset_4|lcl|non-hemolytic
SKVWRHWRRFWHR
>lcl|736|lcl|HAPPENN&HAPPENN-RR90|lcl|subdataset_8|lcl|non-hemolytic
KPTFRRLKWKYK
>lcl|1619|lcl|HAPPENN|lcl|subdataset_4|lcl|non-hemolytic|lcl|cTer
HRWWRWWRH
>lcl|1113|lcl|HAPPENN|lcl|subdataset_8|lcl|non-hemolytic
WFRKQLKW
>lcl|856|lcl|HAPPENN&HAPPENN-RR90&HAPPENN-hard|lcl|subdataset_7|lcl|hemolytic
FFGSLLSLGSKLLPSVFKLFQRKKE
>lcl|1416|lcl|HAPPENN&HAPPENN-RR90&HAPPENN-hard|lcl|subdataset_6|lcl|hemolytic|lcl|nTer|lcl|cTer
INWLKLGKKLLSAL
>lcl|4178|lcl|HAPPENN&HAPPENN-RR90&HAPPENN-hard|lcl|subdataset_5|lcl|non-hemolytic
KKLFKKILKYLAGPATTGLPALISWKDEL
>lcl|2188|lcl|HAPPENN&HAPPENN-hard|lcl|subdataset_6|lcl|non-hemolytic|lcl|cTer
LNALKKVFQGIHEAIKKI
>lcl|2434|lcl|HAPPENN&HAPPENN-hard|lcl|subdataset_4|lcl|non-hemolytic|lcl|cTer
RWKIFKKVVKKG
>lcl|2729|lcl|HAPPENN&HAPPENN-RR90&HAPPENN-hard|lcl|subdataset_9|lcl|non-hemolytic
GIWGTLAKIGIKAVPRVISMLKKKQ
>lcl|2819|lcl|HAPPENN|lcl|subdataset_12|lcl|non-hemolytic|lcl|cTer
CLAIGRR
>lcl|866|lcl|HAPPENN|lcl|subdataset_9|lcl|hemolytic|lcl|cTer
IWRIFRRIFRIF
>lcl|1464|lcl|HAPPENN&HAPPENN-RR90|lcl|subdataset_11|lcl|non-hemolytic|lcl|cTer
KKKLFWKIPKFLHLAKKF
>lcl|4747|lcl|HAPPENN|lcl|subdataset_12|lcl|non-hemolytic|lcl|cTer
KWWKIWRWR
>lcl|2743|lcl|HAPPENN&HAPPENN-hard|lcl|subdataset_6|lcl|non-hemolytic|lcl|cTer
GIGKFLHSAKKWGKAFVGEIMNS
>lcl|495|lcl|HAPPENN&HAPPENN-hard|lcl|subdataset_12|lcl|non-hemolytic
KVFKRLEKLFS
>lcl|4669|lcl|HAPPENN|lcl|subdataset_11|lcl|hemolytic|lcl|cTer
FFSMIPKIATGIASLVKNL
>lcl|3792|lcl|HAPPENN|lcl|subdataset_11|lcl|non-hemolytic|lcl|cTer
GIRRILRYGRRS
>lcl|2719|lcl|HAPPENN|lcl|subdataset_9|lcl|hemolytic
VSRRRRRRGGRRRR
>lcl|1378|lcl|HAPPENN|lcl|subdataset_7|lcl|non-hemolytic|lcl|cTer
KKVVFKVKFK
>lcl|4706|lcl|HAPPENN|lcl|subdataset_7|lcl|non-hemolytic|lcl|nTer|lcl|cTer
KARRWVRAI
>lcl|2380|lcl|HAPPENN&HAPPENN-RR90|lcl|subdataset_9|lcl|non-hemolytic|lcl|cTer
RFFRRFRRFFR
>lcl|2838|lcl|HAPPENN&HAPPENN-RR90&HAPPENN-hard|lcl|subdataset_4|lcl|hemolytic|lcl|cTer
INWSKIFEKVKNLV
>lcl|2675|lcl|HAPPENN|lcl|subdataset_11|lcl|hemolytic
SLWENFKNAGKQFILNILDKIRCRVAGGCRT
>lcl|3213|lcl|HAPPENN&HAPPENN-RR90&HAPPENN-hard|lcl|subdataset_10|lcl|non-hemolytic|lcl|cTer
GIGGVLLSAGKAALKGLTKVLAEKYAN
>lcl|4203|lcl|HAPPENN&HAPPENN-RR90&HAPPENN-hard|lcl|subdataset_12|lcl|hemolytic|lcl|cTer
FLPLLVGAISSILPKIF
>lcl|3415|lcl|HAPPENN|lcl|subdataset_7|lcl|hemolytic
KWRRWVRWI
>lcl|3670|lcl|HAPPENN&HAPPENN-RR90|lcl|subdataset_6|lcl|non-hemolytic|lcl|cTer
GVVVRWGRVWVRGVRR
>lcl|409|lcl|HAPPENN|lcl|subdataset_9|lcl|hemolytic|lcl|cTer
INWLKLGKKILGAI
>lcl|4276|lcl|HAPPENN&HAPPENN-RR90&HAPPENN-hard|lcl|subdataset_12|lcl|hemolytic
GIGKFLHSAKKFFKRIVQRIKDFLR
```

```
>lcl|3140|lcl|HAPPENN&HAPPENN-RR90|lcl|subdataset_7|lcl|non-hemolytic
RFIYMKGFGKPRFGKR
>lcl|2196|lcl|HAPPENN&HAPPENN-RR90|lcl|subdataset_12|lcl|non-hemolytic
TPFLLVGTQIDLR
>lcl|2137|lcl|HAPPENN&HAPPENN-hard|lcl|subdataset_8|lcl|non-hemolytic|lcl|cTer
ALWKSLLKNVGKA
>lcl|3967|lcl|HAPPENN&HAPPENN-RR90&HAPPENN-hard|lcl|subdataset_7|lcl|hemolytic
KRLFKELKFSLRKY
>lcl|4629|lcl|HAPPENN|lcl|subdataset_8|lcl|non-hemolytic|lcl|cTer
RRPRPRPRPWWW
>lcl|1601|lcl|HAPPENN|lcl|subdataset_8|lcl|hemolytic|lcl|cTer
KIAGKIAKIAGKIAKIAGKIAK
>lcl|4602|lcl|HAPPENN|lcl|subdataset_10|lcl|non-hemolytic|lcl|cTer
SGKLCCRRKK
>lcl|1296|lcl|HAPPENN&HAPPENN-RR90&HAPPENN-hard|lcl|subdataset_7|lcl|hemolytic
GLLGKILGVEKKVLCGLSGMC
>lcl|4007|lcl|HAPPENN&HAPPENN-RR90|lcl|subdataset_8|lcl|non-hemolytic
SKGKKANKDVELARG
>lcl|981|lcl|HAPPENN&HAPPENN-RR90&HAPPENN-hard|lcl|subdataset_6|lcl|hemolytic|lcl|cTer
GLKKWFKKAVHVGKKVGKVALNAYL
>lcl|3313|lcl|HAPPENN|lcl|subdataset_9|lcl|hemolytic|lcl|cTer
IKLSPKTKKNLKKVLKGAIKGAIAVAKMV
>lcl|2941|lcl|HAPPENN&HAPPENN-RR90&HAPPENN-hard|lcl|subdataset_7|lcl|non-hemolytic|lcl|cTer
ILKWKWPWWKWRR
>lcl|278|lcl|HAPPENN&HAPPENN-RR90&HAPPENN-hard|lcl|subdataset_5|lcl|non-hemolytic|lcl|cTer
ILPWKWPWAPARR
>lcl|1670|lcl|HAPPENN&HAPPENN-RR90|lcl|subdataset_10|lcl|non-hemolytic|lcl|cTer
KKPKLPWLPLK
>lcl|2003|lcl|HAPPENN&HAPPENN-RR90&HAPPENN-hard|lcl|subdataset_12|lcl|non-hemolytic|lcl|cTer
GIKDLLKGAAKALVKTVLK
>lcl|685|lcl|HAPPENN|lcl|subdataset_5|lcl|hemolytic|lcl|cTer
FFGWLIKGAIHAGKAIHGLIHRRRH
>lcl|1646|lcl|HAPPENN&HAPPENN-RR90&HAPPENN-hard|lcl|subdataset_11|lcl|hemolytic
SFLDKFKDVAIGVAKGAGTGVLKALLCKLDNSC
>lcl|2939|lcl|HAPPENN|lcl|subdataset_8|lcl|hemolytic|lcl|cTer
ILKWKWKWWPWRR
>lcl|4748|lcl|HAPPENN&HAPPENN-hard|lcl|subdataset_9|lcl|non-hemolytic|lcl|cTer
RLWKRWWIR
>lcl|3832|lcl|HAPPENN|lcl|subdataset_12|lcl|non-hemolytic|lcl|cTer
KIAGKIAKIAGKIAKIAGKIA
>lcl|3787|lcl|HAPPENN|lcl|subdataset_5|lcl|non-hemolytic|lcl|cTer
GIRRILKYGKRS
>lcl|4029|lcl|HAPPENN|lcl|subdataset_4|lcl|non-hemolytic
GFCWNVCVYRNGVRVCHRRCN
>lcl|400|lcl|HAPPENN|lcl|subdataset_7|lcl|non-hemolytic|lcl|cTer
VRRFPFFFPFLRR
>lcl|2026|lcl|HAPPENN&HAPPENN-RR90|lcl|subdataset_5|lcl|non-hemolytic
ELDRIGYGTARRKKR
>lcl|3952|lcl|HAPPENN&HAPPENN-RR90&HAPPENN-hard|lcl|subdataset_11|lcl|non-hemolytic
GFFLNALKNFAKTAGKRLKSLLNHASCKLSGQC
>lcl|1476|lcl|HAPPENN&HAPPENN-RR90&HAPPENN-hard|lcl|subdataset_10|lcl|hemolytic
CGESCVWIPCISSAVGCSCKNKVCYKNGTP
>lcl|3886|lcl|HAPPENN&HAPPENN-RR90&HAPPENN-hard|lcl|subdataset_5|lcl|non-hemolytic|lcl|cTer
KRRKAIKILKLIAKLIRKKR
>lcl|4098|lcl|HAPPENN|lcl|subdataset_5|lcl|non-hemolytic
SKVLRHLRRFLHRAHRKL
>lcl|3722|lcl|HAPPENN&HAPPENN-hard|lcl|subdataset_12|lcl|non-hemolytic|lcl|nTer|lcl|cTer
GWGSFFKKAAHVGKHVGKAALTHYL
>lcl|1602|lcl|HAPPENN|lcl|subdataset_9|lcl|hemolytic|lcl|cTer
KIAGKIAKIAGKIAKIAGKIAKIA
>lcl|4238|lcl|HAPPENN&HAPPENN-RR90&HAPPENN-hard|lcl|subdataset_8|lcl|non-hemolytic|lcl|cTer
ILPILAPLIGGLL
>lcl|2245|lcl|HAPPENN|lcl|subdataset_5|lcl|hemolytic
GIPCGESCVWIPCISAAIGCSCKSKVCYRN
>lcl|4490|lcl|HAPPENN&HAPPENN-hard|lcl|subdataset_5|lcl|non-hemolytic|lcl|cTer
AKKVFKRLEKLFSKIWNWK
>lcl|3477|lcl|HAPPENN|lcl|subdataset_4|lcl|non-hemolytic|lcl|cTer
```

```
FLHFLHHLF
>lcl|3713|lcl|HAPPENN&HAPPENN-hard|lcl|subdataset_6|lcl|non-hemolytic|lcl|nTer|lcl|cTer
RRWYRWWR
>lcl|3957|lcl|HAPPENN|lcl|subdataset_10|lcl|non-hemolytic
FFHHIKRGIKHVGKTIHRLVTG
>lcl|550|lcl|HAPPENN&HAPPENN-RR90|lcl|subdataset_4|lcl|non-hemolytic|lcl|cTer
KWKLFKKIGPGKFLHSAKKF
>lcl|284|lcl|HAPPENN&HAPPENN-RR90&HAPPENN-hard|lcl|subdataset_11|lcl|hemolytic
GTPCGESCVYIPCISGVIGCSCTDKVCYLN
>lcl|1790|lcl|HAPPENN&HAPPENN-RR90&HAPPENN-hard|lcl|subdataset_4|lcl|hemolytic
WIRRIKKWIRRVHK
>lcl|779|lcl|HAPPENN&HAPPENN-RR90&HAPPENN-hard|lcl|subdataset_12|lcl|hemolytic
GFISTVKNLATNVAGTVIDTIKCKVTGGC
>lcl|1403|lcl|HAPPENN|lcl|subdataset_7|lcl|hemolytic|lcl|cTer
INWSSIFESVKNLV
>lcl|4780|lcl|HAPPENN&HAPPENN-RR90&HAPPENN-hard|lcl|subdataset_8|lcl|non-hemolytic|lcl|cTer
WGRAFSRGVRRLARGGRG
>lcl|3779|lcl|HAPPENN|lcl|subdataset_4|lcl|non-hemolytic|lcl|cTer
GIHDILKYGKPS
>lcl|3540|lcl|HAPPENN&HAPPENN-RR90&HAPPENN-hard|lcl|subdataset_9|lcl|hemolytic
GLRDKIKNVAIDVAKGAGTGVLKALLCQLDKSC
>lcl|4112|lcl|HAPPENN&HAPPENN-RR90|lcl|subdataset_11|lcl|non-hemolytic|lcl|cTer
RFRRLRCKTRCRLKKI
>lcl|2509|lcl|HAPPENN|lcl|subdataset_11|lcl|non-hemolytic|lcl|cTer
KKLGLWLGLKK
>lcl|1595|lcl|HAPPENN|lcl|subdataset_6|lcl|hemolytic|lcl|cTer
KIAGKIASIAGKIAKIAGSIAKIAGKIA
>lcl|847|lcl|HAPPENN|lcl|subdataset_11|lcl|hemolytic|lcl|cTer
KWKWKWKWKW
>lcl|2073|lcl|HAPPENN&HAPPENN-RR90&HAPPENN-hard|lcl|subdataset_7|lcl|hemolytic|lcl|cTer
KALKKLLAKWLAAAKALL
>lcl|3302|lcl|HAPPENN|lcl|subdataset_10|lcl|hemolytic
GIFPIFAKLLGKVIKVASSLISKGRTE
>lcl|4544|lcl|HAPPENN|lcl|subdataset_5|lcl|hemolytic|lcl|nTer|lcl|cTer
WKLFKKILKVL
>lcl|2190|lcl|HAPPENN&HAPPENN-hard|lcl|subdataset_9|lcl|non-hemolytic
KWKLFKKIGAVLKKL
>lcl|3480|lcl|HAPPENN|lcl|subdataset_6|lcl|non-hemolytic
VDIHVWEGV
>lcl|183|lcl|HAPPENN&HAPPENN-hard|lcl|subdataset_9|lcl|non-hemolytic|lcl|nTer|lcl|cTer
KWKLFKKIGAVLKKL
>lcl|4818|lcl|HAPPENN|lcl|subdataset_11|lcl|hemolytic|lcl|cTer
FLSTIWNGIKSLF
>lcl|1136|lcl|HAPPENN|lcl|subdataset_8|lcl|non-hemolytic
RAGLQFPVGRLLR
>lcl|1140|lcl|HAPPENN&HAPPENN-RR90|lcl|subdataset_12|lcl|non-hemolytic
MASRAAGLAARLARLALRAL
>lcl|2921|lcl|HAPPENN|lcl|subdataset_5|lcl|non-hemolytic
LLLKKFKKKQ
>lcl|3257|lcl|HAPPENN|lcl|subdataset_10|lcl|hemolytic
FLPIIAGAAAKVVEKIFCAISKKC
>lcl|383|lcl|HAPPENN&HAPPENN-RR90&HAPPENN-hard|lcl|subdataset_8|lcl|non-hemolytic
LNLKGIFKKVASLLT
>lcl|2822|lcl|HAPPENN|lcl|subdataset_11|lcl|non-hemolytic|lcl|cTer
AWLLAIRRR
>lcl|4437|lcl|HAPPENN&HAPPENN-RR90|lcl|subdataset_7|lcl|non-hemolytic
IKKILSKIKKLWK
>lcl|910|lcl|HAPPENN&HAPPENN-RR90&HAPPENN-hard|lcl|subdataset_10|lcl|hemolytic|lcl|cTer
VNWKKSLGKSIKVVK
>lcl|2614|lcl|HAPPENN|lcl|subdataset_6|lcl|hemolytic|lcl|cTer
PWWPWRR
>lcl|3878|lcl|HAPPENN&HAPPENN-RR90|lcl|subdataset_9|lcl|non-hemolytic|lcl|cTer
LRKAKKIAKKLF
>lcl|2075|lcl|HAPPENN&HAPPENN-RR90&HAPPENN-hard|lcl|subdataset_7|lcl|hemolytic
FFRNLWKGAKAAFRAGHAAWRA
>lcl|2929|lcl|HAPPENN|lcl|subdataset_10|lcl|non-hemolytic
KKKLKFKKLQ
>lcl|3931|lcl|HAPPENN&HAPPENN-RR90&HAPPENN-hard|lcl|subdataset_5|lcl|non-hemolytic|lcl|cTer
```

```
FLPLIGRVLSGILGWKRKRFG
>lcl|3920|lcl|HAPPENN&HAPPENN-RR90|lcl|subdataset_5|lcl|non-hemolytic
RVVRPVVQVVKQKVR
>lcl|4790|lcl|HAPPENN&HAPPENN-RR90|lcl|subdataset_4|lcl|non-hemolytic
GKYGFYTHVFRLKKWIQKVIDQFGE
>lcl|3036|lcl|HAPPENN&HAPPENN-RR90&HAPPENN-hard|lcl|subdataset_4|lcl|hemolytic|lcl|cTer
GMWSKLLGHLLR
>lcl|1457|lcl|HAPPENN&HAPPENN-RR90|lcl|subdataset_4|lcl|non-hemolytic
HNKQEGRDHDKSKGHFHRVVIHHKGGKAH
>lcl|1852|lcl|HAPPENN&HAPPENN-RR90&HAPPENN-hard|lcl|subdataset_7|lcl|hemolytic
VLPLVGNLLNDLLGK
>lcl|4246|lcl|HAPPENN|lcl|subdataset_8|lcl|non-hemolytic|lcl|cTer
WLLKRWKKLL
>lcl|2608|lcl|HAPPENN&HAPPENN-RR90|lcl|subdataset_8|lcl|non-hemolytic
ATCYCRTGRCATRESLSGVCEISGRLYRLCCR
>lcl|3977|lcl|HAPPENN|lcl|subdataset_6|lcl|hemolytic|lcl|cTer
GVVDILKGAAKDILGHLASKVMNKL
>lcl|504|lcl|HAPPENN&HAPPENN-RR90|lcl|subdataset_10|lcl|non-hemolytic
GCASRCKAKCAGRRCKGWASASFRGRCYCKCFRC
>lcl|4463|lcl|HAPPENN&HAPPENN-RR90&HAPPENN-hard|lcl|subdataset_5|lcl|non-hemolytic|lcl|cTer
GIMSSLMKKLAAHIKK
>lcl|3159|lcl|HAPPENN&HAPPENN-RR90&HAPPENN-hard|lcl|subdataset_7|lcl|hemolytic
GLGSLLGKALKFGLKAAGKFMGGEPQQ
>lcl|4374|lcl|HAPPENN&HAPPENN-hard|lcl|subdataset_6|lcl|non-hemolytic|lcl|cTer
GMLKRIKTLL
>lcl|2924|lcl|HAPPENN|lcl|subdataset_9|lcl|non-hemolytic
LKKLKFKKLQ
>lcl|4254|lcl|HAPPENN&HAPPENN-RR90&HAPPENN-hard|lcl|subdataset_10|lcl|hemolytic|lcl|cTer
FLFKLIPKAIKGLVKAIRK
>lcl|1199|lcl|HAPPENN&HAPPENN-hard|lcl|subdataset_12|lcl|non-hemolytic|lcl|cTer
GLLKRIKTLL
>lcl|380|lcl|HAPPENN|lcl|subdataset_7|lcl|hemolytic
KLKLKFKLKQ
>lcl|1132|lcl|HAPPENN&HAPPENN-RR90&HAPPENN-hard|lcl|subdataset_6|lcl|hemolytic|lcl|cTer
GKGALKKFLAKKVAKTVAKQAAKQGAKYVVNKQME
>lcl|728|lcl|HAPPENN&HAPPENN-RR90|lcl|subdataset_9|lcl|non-hemolytic|lcl|nTer|lcl|cTer
CAKAKAKAGSGAKAKAKAC
>lcl|3167|lcl|HAPPENN&HAPPENN-RR90&HAPPENN-hard|lcl|subdataset_4|lcl|hemolytic|lcl|cTer
FLPIALKALGSIFPKIL
>lcl|922|lcl|HAPPENN|lcl|subdataset_12|lcl|non-hemolytic|lcl|cTer
SFFKKAAHVGKHVGKAALTHYL
>lcl|1604|lcl|HAPPENN|lcl|subdataset_6|lcl|hemolytic|lcl|cTer
KIAGKIAKIAGKIAKIAGKIAKIAGKIA
>lcl|4377|lcl|HAPPENN|lcl|subdataset_4|lcl|non-hemolytic
RGRKVVRRKK
>lcl|3412|lcl|HAPPENN&HAPPENN-RR90&HAPPENN-hard|lcl|subdataset_10|lcl|non-hemolytic|lcl|cTer
HFLGTLVNLKKKIL
>lcl|668|lcl|HAPPENN&HAPPENN-RR90|lcl|subdataset_6|lcl|non-hemolytic|lcl|cTer
KKAAAFAAAAAFAAWAAFAAAKKKK
>lcl|3490|lcl|HAPPENN&HAPPENN-RR90&HAPPENN-hard|lcl|subdataset_6|lcl|non-hemolytic|lcl|cTer
ILQKAVLDCLKAAGSSLSKAAITAIYNKIT
>lcl|1257|lcl|HAPPENN&HAPPENN-RR90&HAPPENN-hard|lcl|subdataset_11|lcl|hemolytic
KWKSFLKTFKSPARTVLHTALKPISS
>lcl|3103|lcl|HAPPENN|lcl|subdataset_5|lcl|hemolytic|lcl|cTer
GFKMALKLLKKVL
>lcl|155|lcl|HAPPENN&HAPPENN-hard|lcl|subdataset_5|lcl|non-hemolytic
RGGRLCYCRRRFCVCE
>lcl|3558|lcl|HAPPENN&HAPPENN-RR90&HAPPENN-hard|lcl|subdataset_5|lcl|hemolytic|lcl|cTer
FLPLKKLRFGLL
>lcl|4626|lcl|HAPPENN|lcl|subdataset_10|lcl|non-hemolytic|lcl|cTer
GRRPRPRPRPFFF
>lcl|3680|lcl|HAPPENN|lcl|subdataset_5|lcl|non-hemolytic|lcl|cTer
GATAIKQVKKLFKKKGG
>lcl|3363|lcl|HAPPENN|lcl|subdataset_8|lcl|hemolytic|lcl|cTer
ILPWKWPWWP
```

```
>lcl|4032|lcl|HAPPENN&HAPPENN-RR90&HAPPENN-hard|lcl|subdataset_11|lcl|non-hemolytic
TISQPEWFKARRWQWRMKKLGA
>lcl|3079|lcl|HAPPENN|lcl|subdataset_12|lcl|hemolytic|lcl|cTer
VNWKKILAKIIKVVK
>lcl|2217|lcl|HAPPENN|lcl|subdataset_4|lcl|non-hemolytic|lcl|cTer
LLLIALRKK
>lcl|4081|lcl|HAPPENN&HAPPENN-RR90|lcl|subdataset_12|lcl|non-hemolytic
KAGLAFPVGRVHRLLRK
>lcl|286|lcl|HAPPENN&HAPPENN-RR90&HAPPENN-hard|lcl|subdataset_8|lcl|hemolytic|lcl|cTer
KLKKLLKKWLKLLKKLLK
>lcl|1514|lcl|HAPPENN&HAPPENN-hard|lcl|subdataset_6|lcl|non-hemolytic|lcl|cTer
IPHAVKAISDLI
>lcl|4035|lcl|HAPPENN|lcl|subdataset_12|lcl|non-hemolytic
EWFKARRWQWRMKKLGA
>lcl|4062|lcl|HAPPENN&HAPPENN-RR90|lcl|subdataset_4|lcl|non-hemolytic|lcl|cTer
DAHGLLKRIKTLL
>lcl|3450|lcl|HAPPENN&HAPPENN-hard|lcl|subdataset_7|lcl|non-hemolytic|lcl|cTer
GIGASILSAGKSALKGLAKGLAEHFAN
>lcl|1739|lcl|HAPPENN&HAPPENN-hard|lcl|subdataset_7|lcl|non-hemolytic|lcl|cTer
FWQRNIRIRR
>lcl|1922|lcl|HAPPENN&HAPPENN-RR90|lcl|subdataset_7|lcl|non-hemolytic
SDDAQRCYPHNRTPFTYTYI
>lcl|2899|lcl|HAPPENN|lcl|subdataset_12|lcl|non-hemolytic
KLKKLFLKKQ
>lcl|157|lcl|HAPPENN&HAPPENN-RR90|lcl|subdataset_8|lcl|non-hemolytic
LCYTRGRFTVCVR
>lcl|3615|lcl|HAPPENN&HAPPENN-RR90&HAPPENN-hard|lcl|subdataset_7|lcl|non-hemolytic
GVLDILKGAAKDLAGHVATKVI
>lcl|3906|lcl|HAPPENN&HAPPENN-hard|lcl|subdataset_7|lcl|non-hemolytic|lcl|nTer|lcl|cTer
KWRRWVRWI
>lcl|1681|lcl|HAPPENN&HAPPENN-RR90&HAPPENN-hard|lcl|subdataset_5|lcl|hemolytic|lcl|cTer
LLGMIKVAITAISALSKL
>lcl|2428|lcl|HAPPENN&HAPPENN-hard|lcl|subdataset_7|lcl|non-hemolytic
FLGGLIKIVPAMICAVTKKC
>lcl|3287|lcl|HAPPENN&HAPPENN-RR90|lcl|subdataset_4|lcl|non-hemolytic
AWNTYDYMKREHSLVKPYQG
>lcl|2021|lcl|HAPPENN&HAPPENN-RR90&HAPPENN-hard|lcl|subdataset_6|lcl|hemolytic|lcl|cTer
ANRLLEAYKMLLKFLGNLR
>lcl|3673|lcl|HAPPENN&HAPPENN-RR90|lcl|subdataset_11|lcl|non-hemolytic
RRRFVVQQDTISPRLEVDERFLPNSVQEQI
>lcl|1999|lcl|HAPPENN&HAPPENN-RR90&HAPPENN-hard|lcl|subdataset_4|lcl|hemolytic|lcl|cTer
KIALKALKALKALGKALKAL
>lcl|4494|lcl|HAPPENN&HAPPENN-RR90&HAPPENN-hard|lcl|subdataset_10|lcl|hemolytic
GMASLLAKVLPKVVKLIK
>lcl|1347|lcl|HAPPENN&HAPPENN-RR90&HAPPENN-hard|lcl|subdataset_9|lcl|hemolytic|lcl|cTer
LNWGAWLKHWWK
>lcl|2562|lcl|HAPPENN&HAPPENN-RR90&HAPPENN-hard|lcl|subdataset_12|lcl|hemolytic
FLIGMTQGLICLITRKC
>lcl|3102|lcl|HAPPENN|lcl|subdataset_9|lcl|hemolytic|lcl|cTer
LFGMALKLLKKVL
>lcl|4775|lcl|HAPPENN|lcl|subdataset_11|lcl|hemolytic|lcl|cTer
FLGMIPGLIGGLISAFK
>lcl|3961|lcl|HAPPENN|lcl|subdataset_4|lcl|non-hemolytic|lcl|cTer
IKKIVSKIKKLL
>lcl|95|lcl|HAPPENN&HAPPENN-RR90|lcl|subdataset_8|lcl|non-hemolytic|lcl|cTer
KKKKKKAAFALWLAFAA
>lcl|2701|lcl|HAPPENN|lcl|subdataset_9|lcl|hemolytic
GLLDTFKNLALNAAKSAGVSVLNSLSCKLSKTC
>lcl|3250|lcl|HAPPENN&HAPPENN-RR90&HAPPENN-hard|lcl|subdataset_8|lcl|hemolytic
FFPIIAGMAAKLIPSLFCKITKKC
>lcl|4224|lcl|HAPPENN&HAPPENN-RR90&HAPPENN-hard|lcl|subdataset_12|lcl|non-hemolytic
NFAEIFAAVNKLIKQGVVKG
>lcl|3087|lcl|HAPPENN|lcl|subdataset_8|lcl|hemolytic|lcl|cTer
VNWKKILG
>lcl|4191|lcl|HAPPENN&HAPPENN-hard|lcl|subdataset_12|lcl|non-hemolytic
SKKLFKKILKYLAGPAGIGKFLHSAK
>lcl|3504|lcl|HAPPENN|lcl|subdataset_12|lcl|non-hemolytic|lcl|cTer
RLYRRLYRRLYRRLYR
```

>lcl|1081|lcl|HAPPENN|lcl|subdataset_5|lcl|non-hemolytic
VDIHVWDGV
>lcl|2986|lcl|HAPPENN|lcl|subdataset_6|lcl|hemolytic|lcl|cTer
KLLKVIKKLL
>lcl|4034|lcl|HAPPENN|lcl|subdataset_11|lcl|non-hemolytic
TASQAEWFKARRWQWRMKKLGA
>lcl|1731|lcl|HAPPENN|lcl|subdataset_8|lcl|non-hemolytic|lcl|nTer|lcl|cTer
KWKSFLKTFKSAKKTELHTALKAISS
>lcl|169|lcl|HAPPENN&HAPPENN-RR90|lcl|subdataset_9|lcl|non-hemolytic
TGRAKRRMQYNRR
>lcl|2861|lcl|HAPPENN|lcl|subdataset_12|lcl|non-hemolytic|lcl|cTer
RRWRRWRRWRR
>lcl|3318|lcl|HAPPENN&HAPPENN-RR90|lcl|subdataset_10|lcl|non-hemolytic
KVALGVAQNYLNPQQ
>lcl|19|lcl|HAPPENN|lcl|subdataset_11|lcl|hemolytic
GLPVCGETCVGGTCNTPGCTCSWPVCTRN
>lcl|2474|lcl|HAPPENN&HAPPENN-RR90&HAPPENN-hard|lcl|subdataset_6|lcl|non-hemolytic
KLKNFAKGVAQSLLNKASCKLSGQC
>lcl|114|lcl|HAPPENN|lcl|subdataset_7|lcl|hemolytic
LCYCRRRFCVCVGR
>lcl|4591|lcl|HAPPENN&HAPPENN-hard|lcl|subdataset_6|lcl|non-hemolytic|lcl|cTer
LLKKLLKKM
>lcl|2179|lcl|HAPPENN&HAPPENN-RR90&HAPPENN-hard|lcl|subdataset_11|lcl|hemolytic|lcl|cTer
WLNALKKVFQGIHEAIKLIWNHVQ
>lcl|830|lcl|HAPPENN|lcl|subdataset_12|lcl|non-hemolytic
WKSYVRRWR
>lcl|4039|lcl|HAPPENN&HAPPENN-RR90|lcl|subdataset_6|lcl|non-hemolytic
KWCRKWQWRGVKFIKCV
>lcl|2064|lcl|HAPPENN&HAPPENN-RR90&HAPPENN-hard|lcl|subdataset_12|lcl|non-hemolytic|lcl|cTer
IIGLVSKGTCVLVKTVCKKVLKQ
>lcl|3192|lcl|HAPPENN|lcl|subdataset_11|lcl|hemolytic
FVGAIAAALPHVISAIKNAL
>lcl|3839|lcl|HAPPENN&HAPPENN-RR90&HAPPENN-hard|lcl|subdataset_9|lcl|hemolytic
GLKALKKVFQGIHKAIKLINNHVQ
>lcl|165|lcl|HAPPENN|lcl|subdataset_7|lcl|hemolytic
LCYCRRRFCWCV
>lcl|3263|lcl|HAPPENN&HAPPENN-RR90&HAPPENN-hard|lcl|subdataset_12|lcl|hemolytic
GLMDTIKGVAKNVAASLLEKLKCKVTGC
>lcl|1400|lcl|HAPPENN|lcl|subdataset_8|lcl|hemolytic|lcl|cTer
IDWKKIFEKVKDLV
>lcl|2972|lcl|HAPPENN&HAPPENN-hard|lcl|subdataset_9|lcl|non-hemolytic|lcl|cTer
GLLKNIKTLL
>lcl|1427|lcl|HAPPENN|lcl|subdataset_4|lcl|hemolytic|lcl|cTer
INWKKIASIGKEVLK
>lcl|498|lcl|HAPPENN&HAPPENN-RR90&HAPPENN-hard|lcl|subdataset_9|lcl|non-hemolytic
AKKVFKRLEKSFSKIQNDK
>lcl|4327|lcl|HAPPENN&HAPPENN-RR90|lcl|subdataset_11|lcl|non-hemolytic|lcl|cTer
GAHDILKYGKPS
>lcl|3653|lcl|HAPPENN&HAPPENN-RR90&HAPPENN-hard|lcl|subdataset_12|lcl|non-hemolytic|lcl|cTer
ILPWKWPKKPWRR
>lcl|3146|lcl|HAPPENN|lcl|subdataset_11|lcl|non-hemolytic
FFSTSCRSGC
>lcl|1669|lcl|HAPPENN|lcl|subdataset_9|lcl|non-hemolytic|lcl|cTer
KKPKLPWLPKK
>lcl|3416|lcl|HAPPENN|lcl|subdataset_8|lcl|hemolytic
KWRRWIRWL
>lcl|1269|lcl|HAPPENN&HAPPENN-hard|lcl|subdataset_8|lcl|non-hemolytic
GFWKKVGSAAWGGVKAAAKGAAVGGLNALAKHIQ
>lcl|4111|lcl|HAPPENN|lcl|subdataset_5|lcl|hemolytic|lcl|cTer
GLFDIIKKIAESF
>lcl|2487|lcl|HAPPENN&HAPPENN-RR90|lcl|subdataset_7|lcl|non-hemolytic|lcl|cTer
WVKAAAKAAAKVW
>lcl|197|lcl|HAPPENN|lcl|subdataset_9|lcl|hemolytic
FWGALAKGALKLIPSLFSSFSKKD
>lcl|1880|lcl|HAPPENN&HAPPENN-RR90|lcl|subdataset_8|lcl|non-hemolytic
LPRRNRWSKIWKKVVTVFS
>lcl|219|lcl|HAPPENN|lcl|subdataset_11|lcl|hemolytic|lcl|cTer
RFGRFLRKIRRFRPKVTITIQGSARF
>lcl|4159|lcl|HAPPENN|lcl|subdataset_7|lcl|hemolytic
GKKLFKKILKYLAGPAKKLFKKILKYL

```
>lcl|4385|lcl|HAPPENN&HAPPENN-RR90|lcl|subdataset_5|lcl|non-hemolytic
ACDFQQCWVTCQRQYSINFISARCNGDSCVCTFRT
>lcl|2534|lcl|HAPPENN&HAPPENN-RR90|lcl|subdataset_6|lcl|non-hemolytic
VRFKLLSHSLLVTLASHL
>lcl|3141|lcl|HAPPENN&HAPPENN-RR90&HAPPENN-hard|lcl|subdataset_8|lcl|non-hemolytic
SADQTGMNKAALSPIRFISKSV
>lcl|1665|lcl|HAPPENN&HAPPENN-RR90&HAPPENN-hard|lcl|subdataset_6|lcl|non-hemolytic
NVLSSVANGINRALSFFG
>lcl|1028|lcl|HAPPENN&HAPPENN-RR90&HAPPENN-hard|lcl|subdataset_8|lcl|non-hemolytic
FIFPKKNIINSLFGR
>lcl|3160|lcl|HAPPENN&HAPPENN-RR90&HAPPENN-hard|lcl|subdataset_5|lcl|non-hemolytic|lcl|cTer
GMASKAGSVAGKIAKFALGAL
>lcl|1097|lcl|HAPPENN&HAPPENN-RR90&HAPPENN-hard|lcl|subdataset_8|lcl|hemolytic
GLADYWRTAFRANFANLGPGIRCKSARC
>lcl|4789|lcl|HAPPENN|lcl|subdataset_11|lcl|hemolytic|lcl|cTer
GVVVRVARVVVRWVRR
>lcl|1479|lcl|HAPPENN&HAPPENN-RR90&HAPPENN-hard|lcl|subdataset_6|lcl|hemolytic
CESCVWIPCISSVVGCSCKSKVCYKNGTLP
>lcl|4483|lcl|HAPPENN&HAPPENN-RR90|lcl|subdataset_7|lcl|non-hemolytic|lcl|cTer
AKKVFKRLEKLFSKIQNWK
>lcl|2466|lcl|HAPPENN&HAPPENN-RR90&HAPPENN-hard|lcl|subdataset_5|lcl|non-hemolytic|lcl|cTer
INWKKIKKKVAGML
>lcl|1089|lcl|HAPPENN&HAPPENN-RR90&HAPPENN-hard|lcl|subdataset_8|lcl|hemolytic
GVWTTILGGLKKFAKGGLEALTNPK
>lcl|32|lcl|HAPPENN&HAPPENN-RR90&HAPPENN-hard|lcl|subdataset_11|lcl|hemolytic|lcl|cTer
RRWCFRVCYRGRFCYRKCR
>lcl|999|lcl|HAPPENN&HAPPENN-RR90|lcl|subdataset_11|lcl|non-hemolytic
SWLRDIWDWKCEVLSDFK
>lcl|2287|lcl|HAPPENN&HAPPENN-RR90|lcl|subdataset_4|lcl|non-hemolytic|lcl|cTer
QKDDEEESRFFFNFIFSAE
>lcl|1869|lcl|HAPPENN|lcl|subdataset_8|lcl|non-hemolytic|lcl|cTer
WKKWWKKW
>lcl|3046|lcl|HAPPENN&HAPPENN-RR90&HAPPENN-hard|lcl|subdataset_9|lcl|hemolytic|lcl|cTer
GKWMSWLKHILK
>lcl|3644|lcl|HAPPENN&HAPPENN-RR90&HAPPENN-hard|lcl|subdataset_8|lcl|hemolytic
FLGAIAGVAAKFLPKVFCFITKKC
>lcl|3013|lcl|HAPPENN|lcl|subdataset_5|lcl|non-hemolytic|lcl|cTer
KIAKVALKAL
>lcl|123|lcl|HAPPENN|lcl|subdataset_5|lcl|non-hemolytic
LAYCRRRFCVAV
>lcl|4607|lcl|HAPPENN|lcl|subdataset_6|lcl|non-hemolytic|lcl|cTer
RWKRWWRRKK
>lcl|933|lcl|HAPPENN&HAPPENN-RR90&HAPPENN-hard|lcl|subdataset_7|lcl|hemolytic|lcl|cTer
LLQWLSKLLGRLL
>lcl|1035|lcl|HAPPENN|lcl|subdataset_12|lcl|hemolytic
SVSNAATRVARTGRSRWRDVARNFMR
>lcl|309|lcl|HAPPENN&HAPPENN-RR90|lcl|subdataset_12|lcl|non-hemolytic|lcl|cTer
FLGWLFKWAKK
>lcl|3351|lcl|HAPPENN|lcl|subdataset_6|lcl|non-hemolytic
RVVRQWPIGRVVRRVVRRVVR
>lcl|473|lcl|HAPPENN|lcl|subdataset_4|lcl|non-hemolytic
KIRVRLSA
>lcl|1291|lcl|HAPPENN&HAPPENN-RR90&HAPPENN-hard|lcl|subdataset_4|lcl|hemolytic
FLPLLLAGLPKLLCLFFKKC
>lcl|4519|lcl|HAPPENN&HAPPENN-hard|lcl|subdataset_10|lcl|non-hemolytic
WPVVIRTVVAGYNLYRAIKKK
>lcl|3166|lcl|HAPPENN&HAPPENN-RR90&HAPPENN-hard|lcl|subdataset_5|lcl|hemolytic|lcl|cTer
GLGSVLGKALKIGANLL
>lcl|4427|lcl|HAPPENN&HAPPENN-RR90|lcl|subdataset_6|lcl|non-hemolytic|lcl|cTer
GLWKGKLGLWKGKLGLWKGKL
>lcl|198|lcl|HAPPENN|lcl|subdataset_9|lcl|hemolytic
FWGALAKGALKLIVSLFSSFSKKD
>lcl|304|lcl|HAPPENN&HAPPENN-hard|lcl|subdataset_7|lcl|non-hemolytic
MESQSDAVQLMLGFGTFVLMLLGLVVELIKNSNKK
>lcl|138|lcl|HAPPENN&HAPPENN-RR90|lcl|subdataset_9|lcl|non-hemolytic
LCYCRFRRCVCV
>lcl|240|lcl|HAPPENN|lcl|subdataset_12|lcl|hemolytic|lcl|cTer
GFFGKRKEYFKKFGASFKRRFANLKKRL
>lcl|2000|lcl|HAPPENN&HAPPENN-RR90&HAPPENN-hard|lcl|subdataset_8|lcl|hemolytic|lcl|cTe
```

```
r
GWKDLLKGAAKALVKTVF
>lcl|3815|lcl|HAPPENN&HAPPENN-RR90|lcl|subdataset_10|lcl|non-hemolytic|lcl|cTer
LLIAAFKKLVKK
>lcl|693|lcl|HAPPENN&HAPPENN-RR90|lcl|subdataset_12|lcl|non-hemolytic|lcl|cTer
KWKLKPLLKKLLKKL
>lcl|4221|lcl|HAPPENN&HAPPENN-RR90&HAPPENN-hard|lcl|subdataset_9|lcl|hemolytic
GILDTLKQLGKAAAQSLLSKAACKLAKTC
>lcl|689|lcl|HAPPENN&HAPPENN-RR90|lcl|subdataset_9|lcl|non-hemolytic
VKKFPWWWPFLKK
>lcl|4661|lcl|HAPPENN|lcl|subdataset_4|lcl|hemolytic
FFFLRRIF
>lcl|2045|lcl|HAPPENN&HAPPENN-RR90|lcl|subdataset_11|lcl|non-hemolytic
KNVNSIVKIQAFFRARKAQDDYR
>lcl|3217|lcl|HAPPENN&HAPPENN-hard|lcl|subdataset_8|lcl|non-hemolytic|lcl|cTer
GIGGALLSAGKSALKGLAKGLAEHL
>lcl|1351|lcl|HAPPENN&HAPPENN-RR90&HAPPENN-hard|lcl|subdataset_11|lcl|non-hemolytic|lcl|cTer
KAYSTPRCKGLFRALMCWL
>lcl|624|lcl|HAPPENN&HAPPENN-RR90|lcl|subdataset_7|lcl|non-hemolytic|lcl|cTer
KWKFKKIPKFLHLAKKF
>lcl|3197|lcl|HAPPENN&HAPPENN-RR90&HAPPENN-hard|lcl|subdataset_12|lcl|hemolytic
GIFGKILGAGKKVLCGLSGLC
>lcl|597|lcl|HAPPENN&HAPPENN-RR90&HAPPENN-hard|lcl|subdataset_6|lcl|hemolytic
IKIPAFVKDTLKKVAKGVISAVAGALTQ
>lcl|1695|lcl|HAPPENN|lcl|subdataset_11|lcl|non-hemolytic|lcl|cTer
KKLFKKILKQL
>lcl|1311|lcl|HAPPENN&HAPPENN-RR90&HAPPENN-hard|lcl|subdataset_4|lcl|hemolytic
GLWSKIKTAGKSVAKAAAKAAVKAVTNAV
>lcl|4218|lcl|HAPPENN&HAPPENN-RR90&HAPPENN-hard|lcl|subdataset_8|lcl|non-hemolytic
FLPLFLPKIICEITKKC
>lcl|3778|lcl|HAPPENN&HAPPENN-RR90&HAPPENN-hard|lcl|subdataset_7|lcl|non-hemolytic|lcl|cTer
GLKGLLGKALKGIGKHIGKAQGC
>lcl|1473|lcl|HAPPENN|lcl|subdataset_7|lcl|hemolytic
CGESCVWIPCISSAIGCSCKSKVCYRNGIP
>lcl|3314|lcl|HAPPENN&HAPPENN-RR90&HAPPENN-hard|lcl|subdataset_8|lcl|hemolytic|lcl|cTer
RVCSWIPLPICH
>lcl|1909|lcl|HAPPENN&HAPPENN-RR90|lcl|subdataset_6|lcl|non-hemolytic
TCRTNRPCFYDLDLNVCRCS
>lcl|4639|lcl|HAPPENN&HAPPENN-RR90&HAPPENN-hard|lcl|subdataset_8|lcl|non-hemolytic
AVNIPFKVHLRCKAAFC
>lcl|1159|lcl|HAPPENN&HAPPENN-RR90&HAPPENN-hard|lcl|subdataset_5|lcl|hemolytic
MLQDWLSSLGDLLKSLLDTVNKFTKK
>lcl|4362|lcl|HAPPENN&HAPPENN-RR90&HAPPENN-hard|lcl|subdataset_7|lcl|non-hemolytic
GLPWILLRWLFFRG
>lcl|3236|lcl|HAPPENN&HAPPENN-RR90&HAPPENN-hard|lcl|subdataset_5|lcl|hemolytic|lcl|cTer
GVVTDLLKTAGKLLGNLFGSLSG
>lcl|3340|lcl|HAPPENN|lcl|subdataset_11|lcl|non-hemolytic|lcl|cTer
WKSDVRRWRSRY
>lcl|867|lcl|HAPPENN&HAPPENN-RR90&HAPPENN-hard|lcl|subdataset_4|lcl|hemolytic|lcl|cTer
IWRIFRRIFRIFIRF
>lcl|4320|lcl|HAPPENN&HAPPENN-RR90&HAPPENN-hard|lcl|subdataset_5|lcl|hemolytic
FWGFLGKLAMKAVPSLIGGNKSSSK
>lcl|1363|lcl|HAPPENN&HAPPENN-RR90&HAPPENN-hard|lcl|subdataset_12|lcl|non-hemolytic
SFHVFPPWMCKSLKKC
>lcl|770|lcl|HAPPENN|lcl|subdataset_11|lcl|hemolytic
ILPWKWPWWPWRR
>lcl|3651|lcl|HAPPENN&HAPPENN-RR90|lcl|subdataset_10|lcl|non-hemolytic
RCPGHTRQIGTSFGPRVKSCRKW
>lcl|3949|lcl|HAPPENN&HAPPENN-RR90&HAPPENN-hard|lcl|subdataset_8|lcl|non-hemolytic|lcl|nTer|lcl|cTer
GWLQLALHLLQLGLHLLQLALQLRRR
>lcl|3898|lcl|HAPPENN|lcl|subdataset_10|lcl|hemolytic|lcl|cTer
KIFGAIWPLALGALKNLIK
>lcl|4379|lcl|HAPPENN|lcl|subdataset_7|lcl|non-hemolytic
RGRKGGRRKK
>lcl|3244|lcl|HAPPENN&HAPPENN-RR90&HAPPENN-hard|lcl|subdataset_9|lcl|hemolytic|lcl|cTer
GECIWDAIFHGAKHFLHRLVNP
>lcl|2381|lcl|HAPPENN&HAPPENN-RR90|lcl|subdataset_7|lcl|non-hemolytic|lcl|cTer
```

```
RRIRIIIRIRR
>lcl|4156|lcl|HAPPENN&HAPPENN-hard|lcl|subdataset_4|lcl|non-hemolytic
KKLFKKILKYLKDEL
>lcl|4590|lcl|HAPPENN&HAPPENN-RR90&HAPPENN-hard|lcl|subdataset_4|lcl|non-hemolytic
NKKAGLFVVQFPKKY
>lcl|1690|lcl|HAPPENN|lcl|subdataset_8|lcl|non-hemolytic|lcl|cTer
KKLFKKILKAL
>lcl|3853|lcl|HAPPENN&HAPPENN-RR90|lcl|subdataset_9|lcl|non-hemolytic|lcl|cTer
RKKKIKIIKKII
>lcl|2189|lcl|HAPPENN&HAPPENN-hard|lcl|subdataset_9|lcl|non-hemolytic|lcl|cTer
LNALKKVFQKIHEAIKKI
>lcl|2634|lcl|HAPPENN&HAPPENN-RR90&HAPPENN-hard|lcl|subdataset_9|lcl|non-hemolytic|lcl|cTer
LGGIVSAVKKIVDFLG
>lcl|4181|lcl|HAPPENN&HAPPENN-RR90&HAPPENN-hard|lcl|subdataset_12|lcl|hemolytic
KKLFKKILKYLKFLHSAK
>lcl|1541|lcl|HAPPENN|lcl|subdataset_8|lcl|non-hemolytic|lcl|nTer|lcl|cTer
WMLKKFRGMF
>lcl|3365|lcl|HAPPENN|lcl|subdataset_5|lcl|hemolytic|lcl|cTer
ILPWKWPW
>lcl|1705|lcl|HAPPENN&HAPPENN-RR90&HAPPENN-hard|lcl|subdataset_8|lcl|hemolytic|lcl|cTer
VCRTGRSRWRDVCRNFMRRYQSR
>lcl|3838|lcl|HAPPENN&HAPPENN-hard|lcl|subdataset_11|lcl|non-hemolytic
GLNALKKVFQGIHEAIKLINNHVK
>lcl|1410|lcl|HAPPENN&HAPPENN-RR90&HAPPENN-hard|lcl|subdataset_11|lcl|hemolytic|lcl|cTer
INWLRLGRRILGAL
>lcl|1127|lcl|HAPPENN|lcl|subdataset_6|lcl|hemolytic
FLGFLKNLF
>lcl|1693|lcl|HAPPENN|lcl|subdataset_8|lcl|non-hemolytic|lcl|cTer
KKLFKKILKGL
>lcl|905|lcl|HAPPENN&HAPPENN-RR90|lcl|subdataset_6|lcl|non-hemolytic
GLPRKILAAIAKKKGKAKGPLKLVAKA
>lcl|3300|lcl|HAPPENN&HAPPENN-RR90&HAPPENN-hard|lcl|subdataset_5|lcl|hemolytic|lcl|cTer
ILGAILPLVSGLLSNKL
>lcl|4451|lcl|HAPPENN&HAPPENN-RR90&HAPPENN-hard|lcl|subdataset_9|lcl|non-hemolytic|lcl|cTer
LILGKLWKGVKSIF
>lcl|4120|lcl|HAPPENN&HAPPENN-RR90&HAPPENN-hard|lcl|subdataset_12|lcl|non-hemolytic
FVKKFWGGVKAIFKGARKGLK
>lcl|1306|lcl|HAPPENN&HAPPENN-hard|lcl|subdataset_9|lcl|non-hemolytic
GFFALIPKIIS
>lcl|2863|lcl|HAPPENN|lcl|subdataset_4|lcl|non-hemolytic
KKKKKFLLLQ
>lcl|1184|lcl|HAPPENN&HAPPENN-RR90&HAPPENN-hard|lcl|subdataset_8|lcl|non-hemolytic
AAAAAAAAAAGIGKFLHSAKKFGKAFVGEIMNS
>lcl|3443|lcl|HAPPENN&HAPPENN-RR90|lcl|subdataset_7|lcl|non-hemolytic
TKCFQWQRNMRKVRGPPVSCIKR
>lcl|4421|lcl|HAPPENN&HAPPENN-RR90|lcl|subdataset_7|lcl|non-hemolytic|lcl|cTer
AKRLKKLAKKIWKWL
>lcl|231|lcl|HAPPENN|lcl|subdataset_9|lcl|hemolytic|lcl|cTer
LKLLKWLLKLL
>lcl|959|lcl|HAPPENN|lcl|subdataset_6|lcl|non-hemolytic
RRWQWRMKK
>lcl|4304|lcl|HAPPENN|lcl|subdataset_12|lcl|non-hemolytic|lcl|nTer|lcl|cTer
KWKSFLKTFKSAKKTVAHTALKAISS
>lcl|4674|lcl|HAPPENN&HAPPENN-RR90|lcl|subdataset_11|lcl|non-hemolytic|lcl|cTer
KKWRWWLKALAKK
>lcl|2833|lcl|HAPPENN&HAPPENN-RR90&HAPPENN-hard|lcl|subdataset_8|lcl|hemolytic|lcl|cTer
YKLLKWLLLKLKALLEKL
>lcl|4353|lcl|HAPPENN&HAPPENN-RR90&HAPPENN-hard|lcl|subdataset_5|lcl|hemolytic|lcl|cTer
GLLKRIKKLLKKIKKLL
>lcl|4838|lcl|HAPPENN&HAPPENN-RR90|lcl|subdataset_6|lcl|non-hemolytic
SAVWRHWRRFWLRKHRKH
>lcl|2867|lcl|HAPPENN|lcl|subdataset_7|lcl|non-hemolytic
KLKKKFKLLQ
>lcl|3706|lcl|HAPPENN&HAPPENN-RR90|lcl|subdataset_10|lcl|non-hemolytic|lcl|nTer|lcl|cTer
KLWKKWKKWLK
```

```
>lcl|3854|lcl|HAPPENN&HAPPENN-RR90|lcl|subdataset_12|lcl|non-hemolytic|lcl|cTer
RKKKAKIIKKII
>lcl|1129|lcl|HAPPENN|lcl|subdataset_4|lcl|non-hemolytic
FLHFLHHLF
>lcl|2178|lcl|HAPPENN|lcl|subdataset_9|lcl|hemolytic|lcl|cTer
WLNALKKVFQGIHEAIKLINNHVQ
>lcl|3710|lcl|HAPPENN&HAPPENN-RR90&HAPPENN-hard|lcl|subdataset_12|lcl|hemolytic|lcl|nTer|lcl|cTer
FKRIVQRIKDFLRGIGKFLHSAKKF
>lcl|2330|lcl|HAPPENN&HAPPENN-RR90|lcl|subdataset_12|lcl|non-hemolytic
KLLKHKLLVTLA
>lcl|93|lcl|HAPPENN&HAPPENN-RR90|lcl|subdataset_4|lcl|non-hemolytic|lcl|cTer
KKKKKKAAFAAWAAFDA
>lcl|14|lcl|HAPPENN|lcl|subdataset_5|lcl|hemolytic
ALWKTLLKKVLKAAAK
>lcl|4842|lcl|HAPPENN|lcl|subdataset_5|lcl|non-hemolytic
CKYLLKWR
>lcl|521|lcl|HAPPENN&HAPPENN-RR90&HAPPENN-hard|lcl|subdataset_8|lcl|hemolytic|lcl|cTer
GLLKKLLKIAAKVGKKLL
>lcl|4525|lcl|HAPPENN|lcl|subdataset_6|lcl|non-hemolytic
TVVAGYNLYRAIKKK
>lcl|4033|lcl|HAPPENN|lcl|subdataset_5|lcl|non-hemolytic
TISQAEWFKARRWQWRMKKLGA
>lcl|1424|lcl|HAPPENN|lcl|subdataset_12|lcl|hemolytic|lcl|cTer
KKIASIGKEVLKAL
>lcl|3800|lcl|HAPPENN|lcl|subdataset_11|lcl|non-hemolytic|lcl|cTer
KRIRKRIKKWLR
>lcl|1538|lcl|HAPPENN&HAPPENN-hard|lcl|subdataset_11|lcl|non-hemolytic|lcl|cTer
GVVDILKGAAKDIAGHLASKVMN
>lcl|1546|lcl|HAPPENN&HAPPENN-RR90&HAPPENN-hard|lcl|subdataset_11|lcl|hemolytic
RRWQWRLCYCRRRFCVCVG
>lcl|3199|lcl|HAPPENN&HAPPENN-hard|lcl|subdataset_4|lcl|non-hemolytic
GLLSKVLGVGKKVLCGVSGLC
>lcl|3922|lcl|HAPPENN&HAPPENN-RR90|lcl|subdataset_6|lcl|non-hemolytic
RLLRPLLQLLKQKLR
>lcl|554|lcl|HAPPENN&HAPPENN-RR90|lcl|subdataset_10|lcl|non-hemolytic|lcl|cTer
EGRERDHELRHRRHHHQSPK
>lcl|4551|lcl|HAPPENN&HAPPENN-RR90&HAPPENN-hard|lcl|subdataset_9|lcl|non-hemolytic|lcl|cTer
FLKGIKGMLGKLF
>lcl|3066|lcl|HAPPENN&HAPPENN-RR90&HAPPENN-hard|lcl|subdataset_7|lcl|non-hemolytic|lcl|cTer
FLPVLAGVLSRA
>lcl|2912|lcl|HAPPENN|lcl|subdataset_4|lcl|non-hemolytic
KKLLLFKKKQ
>lcl|2439|lcl|HAPPENN&HAPPENN-hard|lcl|subdataset_12|lcl|non-hemolytic|lcl|cTer
RGLRRLGRKIAHGVKKYG
>lcl|1933|lcl|HAPPENN&HAPPENN-hard|lcl|subdataset_5|lcl|non-hemolytic|lcl|cTer
RIGSILGRLAKGLPTLKSWIKNR
>lcl|3060|lcl|HAPPENN&HAPPENN-RR90&HAPPENN-hard|lcl|subdataset_6|lcl|hemolytic|lcl|cTer
IFKAIWSGIKSLF
>lcl|4676|lcl|HAPPENN&HAPPENN-RR90&HAPPENN-hard|lcl|subdataset_7|lcl|non-hemolytic|lcl|cTer
LKLKAIAALAKKKW
>lcl|299|lcl|HAPPENN|lcl|subdataset_4|lcl|non-hemolytic
MLSVSDAVQLMLGFGTFVLMLLGLVVELIKNSNKK
>lcl|892|lcl|HAPPENN|lcl|subdataset_4|lcl|non-hemolytic|lcl|cTer
KWKSFIKKLLSKFLHSAKKF
>lcl|12|lcl|HAPPENN|lcl|subdataset_4|lcl|hemolytic
KVLKAAAKAALNAVLVGANA
>lcl|3674|lcl|HAPPENN&HAPPENN-RR90&HAPPENN-hard|lcl|subdataset_10|lcl|non-hemolytic
RRRFVVQQNTISPRLQVNQRFLPNSVQQQI
>lcl|3721|lcl|HAPPENN|lcl|subdataset_6|lcl|non-hemolytic|lcl|nTer|lcl|cTer
KKTWWKTWWTKWKK
>lcl|1893|lcl|HAPPENN&HAPPENN-RR90&HAPPENN-hard|lcl|subdataset_9|lcl|hemolytic|lcl|cTer
FFGSTIGALANFLPSLISKIRN
>lcl|236|lcl|HAPPENN&HAPPENN-RR90&HAPPENN-hard|lcl|subdataset_5|lcl|hemolytic
GLFGKLIKKKGRKAISYAVKKARGKH
>lcl|3402|lcl|HAPPENN|lcl|subdataset_11|lcl|hemolytic
KRLFKELLFSLRKY
>lcl|2877|lcl|HAPPENN|lcl|subdataset_11|lcl|non-hemolytic
```

```
LKKKLFKKLQ
>lcl|227|lcl|HAPPENN&HAPPENN-hard|lcl|subdataset_7|lcl|non-hemolytic|lcl|cTer
KLLKWLLK
>lcl|1824|lcl|HAPPENN&HAPPENN-RR90|lcl|subdataset_9|lcl|non-hemolytic
KIKWILKYWKWS
>lcl|2398|lcl|HAPPENN|lcl|subdataset_7|lcl|hemolytic|lcl|cTer
FLSLIPKAISAISALINHF
>lcl|476|lcl|HAPPENN&HAPPENN-RR90|lcl|subdataset_12|lcl|non-hemolytic|lcl|cTer
KWKKLLKKPLLKKLLKKL
>lcl|3715|lcl|HAPPENN&HAPPENN-RR90&HAPPENN-hard|lcl|subdataset_10|lcl|hemolytic|lcl|nTer|lcl|cTer
KNIRRIIRKIIHIIKKYG
>lcl|4284|lcl|HAPPENN&HAPPENN-hard|lcl|subdataset_6|lcl|non-hemolytic|lcl|cTer
GVFDIIKDAGKQLVAHATGKIAEKV
>lcl|2372|lcl|HAPPENN|lcl|subdataset_6|lcl|non-hemolytic|lcl|cTer
RIRIIIRIR
>lcl|4115|lcl|HAPPENN&HAPPENN-RR90|lcl|subdataset_12|lcl|non-hemolytic|lcl|cTer
RFRRLRPKTRPRLKKI
>lcl|828|lcl|HAPPENN|lcl|subdataset_8|lcl|non-hemolytic
WKSYVRRWRS
>lcl|2243|lcl|HAPPENN&HAPPENN-RR90|lcl|subdataset_10|lcl|non-hemolytic|lcl|cTer
KRFKKWYKAWRKKWRKY
>lcl|3956|lcl|HAPPENN|lcl|subdataset_4|lcl|non-hemolytic
FKHHIFRGIKHVGKTIHRLVTG
>lcl|335|lcl|HAPPENN&HAPPENN-RR90&HAPPENN-hard|lcl|subdataset_9|lcl|hemolytic|lcl|cTer
GLFGVLAKVAAHVVAAIAEHF
>lcl|1013|lcl|HAPPENN&HAPPENN-RR90|lcl|subdataset_6|lcl|non-hemolytic
FKCRRWQWRWKKLGAKPVPIIYCNRRTGKCQRM
>lcl|894|lcl|HAPPENN&HAPPENN-RR90|lcl|subdataset_9|lcl|non-hemolytic|lcl|cTer
KWKSFIKKLTDKFLHSAKKF
>lcl|3555|lcl|HAPPENN&HAPPENN-RR90&HAPPENN-hard|lcl|subdataset_9|lcl|hemolytic|lcl|cTer
FIGALLRPALKLLAGK
>lcl|3383|lcl|HAPPENN&HAPPENN-RR90&HAPPENN-hard|lcl|subdataset_8|lcl|hemolytic|lcl|cTer
ILPIKIPIIPIRR
>lcl|4416|lcl|HAPPENN&HAPPENN-RR90|lcl|subdataset_4|lcl|non-hemolytic
VARGWKRKCPLFGKGG
>lcl|547|lcl|HAPPENN|lcl|subdataset_12|lcl|hemolytic|lcl|cTer
LKLKSIVSWAKAVL
>lcl|2609|lcl|HAPPENN&HAPPENN-RR90&HAPPENN-hard|lcl|subdataset_11|lcl|non-hemolytic|lcl|cTer
RRWKIVVIRWRR
>lcl|4599|lcl|HAPPENN|lcl|subdataset_7|lcl|non-hemolytic
SGKRWWRRKK
>lcl|4797|lcl|HAPPENN&HAPPENN-RR90|lcl|subdataset_12|lcl|non-hemolytic|lcl|cTer
WRNGRWWRNGRWWRNGRW
>lcl|2464|lcl|HAPPENN&HAPPENN-hard|lcl|subdataset_9|lcl|non-hemolytic|lcl|cTer
INWLKIKKKVAGML
>lcl|1256|lcl|HAPPENN|lcl|subdataset_11|lcl|hemolytic
KWKSFLKTFKSPARTVLYTALKPISS
>lcl|1906|lcl|HAPPENN|lcl|subdataset_11|lcl|non-hemolytic
GKPRPYSPRPTSHPKPIRV
>lcl|484|lcl|HAPPENN&HAPPENN-RR90|lcl|subdataset_12|lcl|non-hemolytic
QADFQKVVAGVANALAHRYH
>lcl|4265|lcl|HAPPENN|lcl|subdataset_8|lcl|non-hemolytic
PCRGRSCGPRLRGGYTLIGRPVKNQNRPKYMWV
>lcl|1472|lcl|HAPPENN&HAPPENN-RR90&HAPPENN-hard|lcl|subdataset_8|lcl|hemolytic
CAESCVYIPCTVTALLGCSCSNRVCYNGIP
>lcl|4505|lcl|HAPPENN|lcl|subdataset_8|lcl|non-hemolytic
RIKRFWPVVIRTVVAGYN
>lcl|46|lcl|HAPPENN|lcl|subdataset_9|lcl|hemolytic
FLPVIAGAANFLPKLFCAISKKC
>lcl|1236|lcl|HAPPENN&HAPPENN-RR90&HAPPENN-hard|lcl|subdataset_11|lcl|hemolytic|lcl|cTer
KWKLFKKIEKVGQGIGAVLKVLTTGL
>lcl|3620|lcl|HAPPENN|lcl|subdataset_8|lcl|hemolytic|lcl|cTer
RLWLAIGRG
>lcl|3867|lcl|HAPPENN&HAPPENN-RR90|lcl|subdataset_10|lcl|non-hemolytic|lcl|cTer
KKKKKKIIKKII
>lcl|27|lcl|HAPPENN|lcl|subdataset_11|lcl|hemolytic|lcl|cTer
ILPWKWPWWPWRR
>lcl|2727|lcl|HAPPENN&HAPPENN-RR90&HAPPENN-hard|lcl|subdataset_12|lcl|hemolytic
```

```
GIKDWIKGAAKKLIKTVASHIANQ
>lcl|575|lcl|HAPPENN&HAPPENN-RR90|lcl|subdataset_12|lcl|non-hemolytic
VHVPPICSHRECRK
>lcl|4511|lcl|HAPPENN|lcl|subdataset_11|lcl|non-hemolytic
RIKRFWPVVIRTV
>lcl|3297|lcl|HAPPENN&HAPPENN-hard|lcl|subdataset_11|lcl|non-hemolytic
KFASKGLGKDLAKLGVDLVACKISKQC
>lcl|4803|lcl|HAPPENN&HAPPENN-hard|lcl|subdataset_4|lcl|non-hemolytic
FYTHVFRLKKWMQKVIDRFGG
>lcl|4601|lcl|HAPPENN|lcl|subdataset_12|lcl|non-hemolytic
RKKRWWRRKK
>lcl|1660|lcl|HAPPENN|lcl|subdataset_10|lcl|hemolytic
FFPGIIKVASAILPTAICAITKRC
>lcl|718|lcl|HAPPENN&HAPPENN-RR90&HAPPENN-hard|lcl|subdataset_5|lcl|non-hemolytic|lcl|cTer
FLSLIPHAINAVSAIAKHN
>lcl|791|lcl|HAPPENN|lcl|subdataset_6|lcl|non-hemolytic
GEKLKKIGQKIKNFFQKL
>lcl|1380|lcl|HAPPENN|lcl|subdataset_7|lcl|hemolytic
KLLKLLLKLLLKLLK
>lcl|2716|lcl|HAPPENN|lcl|subdataset_8|lcl|hemolytic
KWCFRVCYRGICYRRCR
>lcl|1053|lcl|HAPPENN&HAPPENN-hard|lcl|subdataset_12|lcl|non-hemolytic|lcl|cTer
CIAKGNGCQPSGVQGNCCSGHCHKEPGWVAGYCK
>lcl|1879|lcl|HAPPENN|lcl|subdataset_6|lcl|non-hemolytic
RLCRIVWVIRVCR
>lcl|2473|lcl|HAPPENN&HAPPENN-RR90|lcl|subdataset_8|lcl|non-hemolytic|lcl|nTer|lcl|cTer
LFKKNKLWFKSKKWL
>lcl|789|lcl|HAPPENN|lcl|subdataset_11|lcl|non-hemolytic
GWGSFFKKAAHVGKHVAKAALTHYL
>lcl|549|lcl|HAPPENN|lcl|subdataset_9|lcl|non-hemolytic|lcl|cTer
KWKLFKKIPKFLHSAKKF
>lcl|2443|lcl|HAPPENN|lcl|subdataset_7|lcl|non-hemolytic
HLNKRVQRELRGWLDWLK
>lcl|3860|lcl|HAPPENN&HAPPENN-hard|lcl|subdataset_12|lcl|non-hemolytic|lcl|cTer
KRKKLLKRLL
>lcl|1861|lcl|HAPPENN&HAPPENN-hard|lcl|subdataset_8|lcl|non-hemolytic
GLTRLFSVIK
>lcl|1165|lcl|HAPPENN&HAPPENN-RR90&HAPPENN-hard|lcl|subdataset_4|lcl|hemolytic|lcl|cTer
IWLTALKFLGKHAAKHLAKQQLSKL
>lcl|2403|lcl|HAPPENN&HAPPENN-RR90|lcl|subdataset_12|lcl|non-hemolytic
FIFHIIRFFNFRVFRI
>lcl|37|lcl|HAPPENN|lcl|subdataset_4|lcl|hemolytic|lcl|cTer
RGLRRLGRKIAHGVKKYGATVLRIIRIA
>lcl|3196|lcl|HAPPENN&HAPPENN-RR90&HAPPENN-hard|lcl|subdataset_5|lcl|hemolytic
FLPAVIRVAANVLPTVFCAISKKC
>lcl|763|lcl|HAPPENN&HAPPENN-RR90|lcl|subdataset_4|lcl|non-hemolytic|lcl|cTer
RGGRLAYARRRFAVAVGR
>lcl|3985|lcl|HAPPENN|lcl|subdataset_9|lcl|non-hemolytic|lcl|cTer
GVVDILAGAAKDIAGHLASKVMNKL
>lcl|148|lcl|HAPPENN|lcl|subdataset_8|lcl|hemolytic
RGGRLCYCRRRFCVCY
>lcl|2828|lcl|HAPPENN|lcl|subdataset_5|lcl|hemolytic|lcl|cTer
YKLLKLLLPKLKGLLIKL
>lcl|4465|lcl|HAPPENN&HAPPENN-RR90&HAPPENN-hard|lcl|subdataset_6|lcl|non-hemolytic|lcl|cTer
GIMISLMKKLAAHIAK
>lcl|2191|lcl|HAPPENN|lcl|subdataset_12|lcl|non-hemolytic
KWKKFKKIGKVLKVL
>lcl|366|lcl|HAPPENN&HAPPENN-RR90&HAPPENN-hard|lcl|subdataset_6|lcl|non-hemolytic
VGKVLKQLKKVSAVAKVAMKKGAALLK
>lcl|242|lcl|HAPPENN&HAPPENN-RR90&HAPPENN-hard|lcl|subdataset_10|lcl|hemolytic|lcl|cTer
ILPWKWKWWPWRR
>lcl|2705|lcl|HAPPENN&HAPPENN-RR90&HAPPENN-hard|lcl|subdataset_9|lcl|hemolytic
FLPAVLRVAAQVVPTVFCAISKKC
>lcl|1176|lcl|HAPPENN|lcl|subdataset_4|lcl|hemolytic|lcl|cTer
FFHHIFRGIVHVGKTIHKLVTG
>lcl|3091|lcl|HAPPENN&HAPPENN-RR90&HAPPENN-hard|lcl|subdataset_5|lcl|hemolytic|lcl|cTer
VNFKKLLGKLLKVVK
```

```
>lcl|1666|lcl|HAPPENN&HAPPENN-RR90|lcl|subdataset_9|lcl|non-hemolytic|lcl|cTer
KKPKKPWKPKK
>lcl|646|lcl|HAPPENN&HAPPENN-hard|lcl|subdataset_5|lcl|non-hemolytic
KNLRRIIRKGIHIIKKYG
>lcl|2904|lcl|HAPPENN|lcl|subdataset_11|lcl|non-hemolytic
KLLKKFLKKQ
>lcl|2726|lcl|HAPPENN&HAPPENN-RR90&HAPPENN-hard|lcl|subdataset_11|lcl|hemolytic|lcl|cTer
GFRDVLKGAAKAFVKTVAGHIAN
>lcl|3767|lcl|HAPPENN|lcl|subdataset_6|lcl|non-hemolytic|lcl|cTer
RRWYRWWR
>lcl|4296|lcl|HAPPENN&HAPPENN-RR90&HAPPENN-hard|lcl|subdataset_8|lcl|hemolytic|lcl|cTer
FIGALLGPLLNLLK
>lcl|4219|lcl|HAPPENN&HAPPENN-RR90&HAPPENN-hard|lcl|subdataset_12|lcl|hemolytic
GLWNTIKEAGKKFALNLLDKIRCGIAGGCKG
>lcl|4579|lcl|HAPPENN|lcl|subdataset_8|lcl|non-hemolytic
RRFWHRAH
>lcl|4448|lcl|HAPPENN&HAPPENN-RR90&HAPPENN-hard|lcl|subdataset_10|lcl|non-hemolytic|lcl|nTer|lcl|cTer
IDGLKAIWKKVADLLKNT
>lcl|3475|lcl|HAPPENN|lcl|subdataset_6|lcl|hemolytic|lcl|cTer
FLGFLKNLF
>lcl|3933|lcl|HAPPENN|lcl|subdataset_9|lcl|non-hemolytic|lcl|cTer
IKKILSKIKKLLK
>lcl|447|lcl|HAPPENN|lcl|subdataset_8|lcl|hemolytic|lcl|cTer
LKLFKKILKYL
>lcl|3232|lcl|HAPPENN&HAPPENN-RR90|lcl|subdataset_12|lcl|non-hemolytic
GAARKSIRLHRLYTWKATIYTR
>lcl|1110|lcl|HAPPENN&HAPPENN-RR90&HAPPENN-hard|lcl|subdataset_12|lcl|hemolytic|lcl|cTer
ILGKLLSTAWGLLSNL
>lcl|3866|lcl|HAPPENN&HAPPENN-RR90|lcl|subdataset_7|lcl|non-hemolytic|lcl|cTer
RKKKKKIIKKLI
>lcl|4786|lcl|HAPPENN|lcl|subdataset_8|lcl|hemolytic|lcl|cTer
GVVVRVGRVVVRWVRR
>lcl|4523|lcl|HAPPENN|lcl|subdataset_12|lcl|non-hemolytic
IRTVVAGYNLYRAIKKK
>lcl|1050|lcl|HAPPENN&HAPPENN-RR90&HAPPENN-hard|lcl|subdataset_8|lcl|non-hemolytic
FSPQMLQDIIEAATAIL
>lcl|4014|lcl|HAPPENN|lcl|subdataset_4|lcl|hemolytic|lcl|cTer
GLLKWIKKLL
>lcl|1418|lcl|HAPPENN|lcl|subdataset_4|lcl|hemolytic|lcl|cTer
SNWLKLGKKMMSAL
>lcl|2630|lcl|HAPPENN|lcl|subdataset_6|lcl|hemolytic|lcl|cTer
GIGAVLWVLTTGLPALISWIKRKRQQ
>lcl|4662|lcl|HAPPENN|lcl|subdataset_6|lcl|non-hemolytic
RYPAVGYT
>lcl|3253|lcl|HAPPENN&HAPPENN-RR90&HAPPENN-hard|lcl|subdataset_5|lcl|hemolytic|lcl|cTer
FLSAITSLLGKLL
>lcl|3599|lcl|HAPPENN|lcl|subdataset_7|lcl|non-hemolytic
PKVTITIQGSARF
>lcl|4587|lcl|HAPPENN&HAPPENN-RR90&HAPPENN-hard|lcl|subdataset_4|lcl|non-hemolytic|lcl|cTer
GMWSKIKNAGKAAAKAAAKAAGKAALDAVSEAI
>lcl|3683|lcl|HAPPENN&HAPPENN-RR90&HAPPENN-hard|lcl|subdataset_4|lcl|hemolytic
FLKALFKVALKVL
>lcl|2890|lcl|HAPPENN|lcl|subdataset_4|lcl|non-hemolytic
KLLKKFKLKQ
>lcl|1120|lcl|HAPPENN|lcl|subdataset_10|lcl|non-hemolytic
RRLMAAKAESR
>lcl|2295|lcl|HAPPENN|lcl|subdataset_12|lcl|hemolytic|lcl|cTer
YCSYTMEA
>lcl|2657|lcl|HAPPENN&HAPPENN-RR90&HAPPENN-hard|lcl|subdataset_4|lcl|hemolytic
FLSTLLNVASKVVPTLFCKITKKC
>lcl|2002|lcl|HAPPENN&HAPPENN-hard|lcl|subdataset_5|lcl|non-hemolytic|lcl|cTer
GFKDLLKGAAKALVKTVLK
>lcl|3121|lcl|HAPPENN&HAPPENN-RR90&HAPPENN-hard|lcl|subdataset_7|lcl|hemolytic|lcl|cTer
AILTTLANWARKFL
>lcl|1183|lcl|HAPPENN&HAPPENN-RR90|lcl|subdataset_6|lcl|non-hemolytic
KKKKKKKKKGIGKFLHSAKKFGKAFVGEIMNS
```

```
>lcl|1219|lcl|HAPPENN&HAPPENN-RR90&HAPPENN-hard|lcl|subdataset_11|lcl|hemolytic|lcl|cTer
LKLKSIVSWALKVL
>lcl|3875|lcl|HAPPENN&HAPPENN-RR90&HAPPENN-hard|lcl|subdataset_10|lcl|hemolytic|lcl|cTer
LRFLRRILRRLL
>lcl|1142|lcl|HAPPENN&HAPPENN-RR90|lcl|subdataset_12|lcl|non-hemolytic
MAARAARLAARLARLALRAL
>lcl|787|lcl|HAPPENN&HAPPENN-RR90|lcl|subdataset_6|lcl|non-hemolytic|lcl|cTer
KWKKLPKKLLKLL
>lcl|1082|lcl|HAPPENN|lcl|subdataset_11|lcl|non-hemolytic
LGRVDIHVWDAVYIRGR
>lcl|2183|lcl|HAPPENN&HAPPENN-RR90|lcl|subdataset_5|lcl|non-hemolytic|lcl|cTer
GLNALKKVSQGIHESIKLINNHVQ
>lcl|3728|lcl|HAPPENN|lcl|subdataset_10|lcl|hemolytic|lcl|nTer|lcl|cTer
RGLRRLGRKIAHGVKKYGPTVLRIIRTAT
>lcl|4213|lcl|HAPPENN&HAPPENN-hard|lcl|subdataset_10|lcl|non-hemolytic
NILSSIANGINRALSFFG
>lcl|2992|lcl|HAPPENN&HAPPENN-hard|lcl|subdataset_4|lcl|non-hemolytic
SLLSLIRKLIT
>lcl|4745|lcl|HAPPENN|lcl|subdataset_6|lcl|non-hemolytic|lcl|cTer
RIWKIWWKR
>lcl|3322|lcl|HAPPENN&HAPPENN-RR90&HAPPENN-hard|lcl|subdataset_5|lcl|hemolytic|lcl|cTer
SGTSEKERESGRLLGVVKRLIVCFRSPFP
>lcl|849|lcl|HAPPENN|lcl|subdataset_9|lcl|hemolytic
GECIWDAIFHGAKHFLHRLVNP
>lcl|565|lcl|HAPPENN&HAPPENN-RR90&HAPPENN-hard|lcl|subdataset_8|lcl|hemolytic|lcl|cTer
LLKKLLKKLLKKLLKK
>lcl|255|lcl|HAPPENN&HAPPENN-RR90&HAPPENN-hard|lcl|subdataset_10|lcl|hemolytic|lcl|cTer
ILPWKLPWWPWRR
>lcl|1680|lcl|HAPPENN|lcl|subdataset_11|lcl|hemolytic|lcl|cTer
LLGMIPVAIKAISALSKL
>lcl|1691|lcl|HAPPENN|lcl|subdataset_11|lcl|non-hemolytic|lcl|cTer
KKLFKKILKLL
>lcl|1715|lcl|HAPPENN&HAPPENN-RR90|lcl|subdataset_11|lcl|non-hemolytic
RILTMTKRVKMPQLYKQIVCRLFKTC
>lcl|4306|lcl|HAPPENN|lcl|subdataset_9|lcl|non-hemolytic|lcl|nTer|lcl|cTer
KWKSFAKTFKSAKKTVAHTALKAISS
>lcl|129|lcl|HAPPENN&HAPPENN-RR90|lcl|subdataset_9|lcl|non-hemolytic
LCYTRPRFTVCV
>lcl|605|lcl|HAPPENN&HAPPENN-RR90&HAPPENN-hard|lcl|subdataset_9|lcl|hemolytic
AWASFFKKAAHVAKHVAKAALTHYL
>lcl|2456|lcl|HAPPENN&HAPPENN-RR90|lcl|subdataset_11|lcl|non-hemolytic|lcl|nTer|lcl|cTer
LRRLKPLIRPWLRPLRRWWW
>lcl|4099|lcl|HAPPENN|lcl|subdataset_6|lcl|hemolytic|lcl|cTer
GWLDVAKKIGKAAFNVAKNFI
>lcl|932|lcl|HAPPENN|lcl|subdataset_10|lcl|hemolytic|lcl|cTer
FVQWFSKFLGRIL
>lcl|2365|lcl|HAPPENN|lcl|subdataset_8|lcl|non-hemolytic|lcl|nTer|lcl|cTer
KKWRKWLAKK
>lcl|2880|lcl|HAPPENN|lcl|subdataset_9|lcl|non-hemolytic
KKLKKFLLKQ
>lcl|4486|lcl|HAPPENN&HAPPENN-RR90&HAPPENN-hard|lcl|subdataset_11|lcl|hemolytic|lcl|cTer
INWKKMAATALKMI
>lcl|1260|lcl|HAPPENN&HAPPENN-RR90&HAPPENN-hard|lcl|subdataset_9|lcl|hemolytic
IISTIGKLVKWIIKTV
>lcl|2390|lcl|HAPPENN&HAPPENN-RR90&HAPPENN-hard|lcl|subdataset_10|lcl|non-hemolytic
LSVKAFTGIQLRGVCGIEVKARG
>lcl|4190|lcl|HAPPENN&HAPPENN-hard|lcl|subdataset_12|lcl|non-hemolytic
GKKLFKKILKYLAGPAGIGKFLHSAK
>lcl|448|lcl|HAPPENN|lcl|subdataset_11|lcl|non-hemolytic|lcl|cTer
KKLFKKILKKL
>lcl|1356|lcl|HAPPENN|lcl|subdataset_10|lcl|non-hemolytic|lcl|cTer
KKVVFKVKFKK
>lcl|68|lcl|HAPPENN|lcl|subdataset_11|lcl|hemolytic
FFHHIFRPIVHVGKTIHRLVTG
>lcl|2305|lcl|HAPPENN&HAPPENN-hard|lcl|subdataset_10|lcl|non-hemolytic|lcl|cTer
PFKLSLHL
>lcl|4839|lcl|HAPPENN&HAPPENN-hard|lcl|subdataset_7|lcl|non-hemolytic
```

```
SAVWRWRRFWLRKRK
>lcl|1377|lcl|HAPPENN&HAPPENN-RR90&HAPPENN-hard|lcl|subdataset_7|lcl|non-hemolytic
AVVKVPLKKFKSIRETMKEKGLLEDF
>lcl|1609|lcl|HAPPENN&HAPPENN-RR90&HAPPENN-hard|lcl|subdataset_11|lcl|non-hemolytic
FKAFRWAWRWKKLAAPS
>lcl|4496|lcl|HAPPENN|lcl|subdataset_9|lcl|non-hemolytic|lcl|cTer
AKKVFKRLEKSFSKIQNDK
>lcl|2811|lcl|HAPPENN&HAPPENN-RR90|lcl|subdataset_5|lcl|non-hemolytic|lcl|cTer
LCAAHCLAILRR
>lcl|3707|lcl|HAPPENN|lcl|subdataset_9|lcl|non-hemolytic|lcl|nTer|lcl|cTer
KLWKKWKKWKK
>lcl|3172|lcl|HAPPENN&HAPPENN-hard|lcl|subdataset_7|lcl|non-hemolytic
FLGPIIKMATGILPTAICKGLKKC
>lcl|4570|lcl|HAPPENN&HAPPENN-RR90|lcl|subdataset_5|lcl|non-hemolytic
HNSSKQWSHWLWHNGIRI
>lcl|2515|lcl|HAPPENN&HAPPENN-RR90|lcl|subdataset_8|lcl|non-hemolytic|lcl|cTer
RRLGLWLGLRR
>lcl|2048|lcl|HAPPENN&HAPPENN-RR90|lcl|subdataset_5|lcl|non-hemolytic|lcl|cTer
GLLRRLRDFLKKIGEKFKKIGY
>lcl|1644|lcl|HAPPENN&HAPPENN-RR90&HAPPENN-hard|lcl|subdataset_7|lcl|non-hemolytic
DYSIRTRLHQESSRNVF
>lcl|3064|lcl|HAPPENN|lcl|subdataset_11|lcl|non-hemolytic|lcl|cTer
GIGKHVGKALKGLKGLLKGLGES
>lcl|3814|lcl|HAPPENN&HAPPENN-RR90|lcl|subdataset_8|lcl|non-hemolytic|lcl|cTer
KKKAARALKRAL
>lcl|1497|lcl|HAPPENN&HAPPENN-RR90|lcl|subdataset_4|lcl|non-hemolytic
FKTWKRPPFQTSCSGIIKE
>lcl|4212|lcl|HAPPENN&HAPPENN-RR90|lcl|subdataset_5|lcl|non-hemolytic
FLPPSPWKETFRTT
>lcl|1528|lcl|HAPPENN&HAPPENN-RR90&HAPPENN-hard|lcl|subdataset_8|lcl|hemolytic|lcl|cTer
INLKALAALAKALL
>lcl|1613|lcl|HAPPENN|lcl|subdataset_7|lcl|non-hemolytic|lcl|cTer
RRWHRWWRR
>lcl|1844|lcl|HAPPENN&HAPPENN-RR90|lcl|subdataset_5|lcl|non-hemolytic
VDNANDLLSKVKKDKSD
>lcl|4456|lcl|HAPPENN&HAPPENN-RR90|lcl|subdataset_9|lcl|non-hemolytic
GWWRRTVDKVRNAGRKVAGFASKACGALGH
>lcl|3422|lcl|HAPPENN|lcl|subdataset_10|lcl|non-hemolytic|lcl|cTer
KKIMRTFLRR
>lcl|3180|lcl|HAPPENN&HAPPENN-RR90|lcl|subdataset_12|lcl|non-hemolytic
NALSSPRNKCDRASSCFG
>lcl|4632|lcl|HAPPENN|lcl|subdataset_4|lcl|non-hemolytic|lcl|cTer
RRPRPRPRPFFFF
>lcl|4672|lcl|HAPPENN&HAPPENN-RR90&HAPPENN-hard|lcl|subdataset_6|lcl|non-hemolytic|lcl|cTer
FLSMIPHIAGGIASLVKNL
>lcl|1651|lcl|HAPPENN&HAPPENN-RR90&HAPPENN-hard|lcl|subdataset_10|lcl|non-hemolytic
FFLLPIPNDVKCKVLGICKS
>lcl|3602|lcl|HAPPENN&HAPPENN-RR90|lcl|subdataset_12|lcl|non-hemolytic
SSMKLSFRARAYGFRGPGPQL
>lcl|3597|lcl|HAPPENN|lcl|subdataset_12|lcl|non-hemolytic
LVQRGRFGRFLRKIRRFR
>lcl|548|lcl|HAPPENN&HAPPENN-RR90&HAPPENN-hard|lcl|subdataset_6|lcl|non-hemolytic|lcl|cTer
KWKLFKKIKFLHSAKKF
>lcl|1765|lcl|HAPPENN&HAPPENN-RR90&HAPPENN-hard|lcl|subdataset_6|lcl|hemolytic
GLPTCGETCFKGKCYTPGCSCSYPICKKN
>lcl|440|lcl|HAPPENN&HAPPENN-hard|lcl|subdataset_5|lcl|non-hemolytic
AKKVFKRLEKLFSKIWNWK
>lcl|2027|lcl|HAPPENN&HAPPENN-RR90&HAPPENN-hard|lcl|subdataset_9|lcl|hemolytic|lcl|cTer
KLLAKAALKWLLKALKAA
>lcl|1152|lcl|HAPPENN&HAPPENN-hard|lcl|subdataset_9|lcl|non-hemolytic|lcl|cTer
QQRKRKGIGAVLKVLTTGLPALISWI
>lcl|4488|lcl|HAPPENN&HAPPENN-RR90|lcl|subdataset_7|lcl|non-hemolytic
GLPGPLGPAGPK
>lcl|199|lcl|HAPPENN|lcl|subdataset_12|lcl|hemolytic
FWGALAKGALKLIGVSLFSSFSKKD
>lcl|2868|lcl|HAPPENN|lcl|subdataset_10|lcl|non-hemolytic
LKKKKFKLLQ
>lcl|2389|lcl|HAPPENN|lcl|subdataset_4|lcl|non-hemolytic|lcl|cTer
RGRRSSRRKK
```

```
>lcl|2884|lcl|HAPPENN|lcl|subdataset_4|lcl|non-hemolytic
KKLKLFKLKQ
>lcl|3403|lcl|HAPPENN&HAPPENN-hard|lcl|subdataset_4|lcl|non-hemolytic
FKRLEKLF
>lcl|1610|lcl|HAPPENN&HAPPENN-RR90&HAPPENN-hard|lcl|subdataset_8|lcl|non-hemolytic|lcl|cTer
IFSAIAGLLSNLL
>lcl|368|lcl|HAPPENN|lcl|subdataset_5|lcl|hemolytic
GKVLDKFGKIVGKVLKQLKKVSAVAKV
>lcl|4724|lcl|HAPPENN&HAPPENN-RR90|lcl|subdataset_10|lcl|non-hemolytic|lcl|cTer
FPLTWPTKWWKG
>lcl|3658|lcl|HAPPENN&HAPPENN-RR90&HAPPENN-hard|lcl|subdataset_11|lcl|hemolytic
RRRRRRWFWFWF
>lcl|2042|lcl|HAPPENN&HAPPENN-RR90&HAPPENN-hard|lcl|subdataset_7|lcl|non-hemolytic
ANLDAIIKIQAWARMWAARRQYL
>lcl|2040|lcl|HAPPENN&HAPPENN-RR90|lcl|subdataset_10|lcl|non-hemolytic
ANVGFVIQLQARLRGFLVRQKFA
>lcl|2366|lcl|HAPPENN|lcl|subdataset_8|lcl|non-hemolytic|lcl|nTer|lcl|cTer
FKKLKKLFSKLWNWK
>lcl|1712|lcl|HAPPENN|lcl|subdataset_9|lcl|hemolytic|lcl|cTer
FASGIAGMAGKLF
>lcl|2905|lcl|HAPPENN|lcl|subdataset_8|lcl|non-hemolytic
RPPHPRL
>lcl|1573|lcl|HAPPENN|lcl|subdataset_8|lcl|hemolytic|lcl|cTer
ALWKTMLKKLGT
>lcl|396|lcl|HAPPENN|lcl|subdataset_9|lcl|non-hemolytic|lcl|cTer
VKKFPWWWPFLKK
>lcl|577|lcl|HAPPENN|lcl|subdataset_7|lcl|non-hemolytic
LRRLWLRANRL
>lcl|1015|lcl|HAPPENN|lcl|subdataset_5|lcl|non-hemolytic
ELSKAVRLIK
>lcl|501|lcl|HAPPENN&HAPPENN-hard|lcl|subdataset_5|lcl|non-hemolytic
KWCFRVCYRGICYRRC
>lcl|2575|lcl|HAPPENN|lcl|subdataset_6|lcl|hemolytic
DSHAKRHHGYKRKFHEKHHSHRGY
>lcl|434|lcl|HAPPENN&HAPPENN-RR90|lcl|subdataset_12|lcl|non-hemolytic
AKKVFKRLRKLFKKI
>lcl|1414|lcl|HAPPENN|lcl|subdataset_9|lcl|hemolytic|lcl|cTer
INWLKLGKKMMSAL
>lcl|4817|lcl|HAPPENN|lcl|subdataset_6|lcl|hemolytic|lcl|cTer
IFGAIWSGIKSLF
>lcl|4573|lcl|HAPPENN|lcl|subdataset_8|lcl|non-hemolytic
WRHWRRFWHR
>lcl|2873|lcl|HAPPENN|lcl|subdataset_11|lcl|non-hemolytic
LKKKKFLKLQ
>lcl|2484|lcl|HAPPENN&HAPPENN-RR90&HAPPENN-hard|lcl|subdataset_6|lcl|non-hemolytic|lcl|cTer
LLKAAAKAAAKLL
>lcl|1393|lcl|HAPPENN&HAPPENN-RR90&HAPPENN-hard|lcl|subdataset_12|lcl|hemolytic|lcl|cTer
FLSSIGKILGNLL
>lcl|1902|lcl|HAPPENN&HAPPENN-RR90|lcl|subdataset_9|lcl|non-hemolytic
GRPNPVPNPRPPHPRL
>lcl|3865|lcl|HAPPENN&HAPPENN-RR90|lcl|subdataset_7|lcl|non-hemolytic|lcl|cTer
RKKRKKLLKRLI
>lcl|4109|lcl|HAPPENN|lcl|subdataset_10|lcl|hemolytic|lcl|cTer
RFRRLRKKFRKRLKKI
>lcl|1800|lcl|HAPPENN|lcl|subdataset_10|lcl|non-hemolytic|lcl|cTer
RKKTRKRLKKIGKVLKWI
>lcl|4172|lcl|HAPPENN|lcl|subdataset_7|lcl|non-hemolytic
KDWEHLKDWEHLKDWEHLKDEL
>lcl|3359|lcl|HAPPENN&HAPPENN-RR90|lcl|subdataset_6|lcl|non-hemolytic|lcl|cTer
KKAAASAAAAASAAWAASAAAKKKK
>lcl|1978|lcl|HAPPENN&HAPPENN-RR90&HAPPENN-hard|lcl|subdataset_7|lcl|hemolytic
FFGHLFKLATKIIPSLFQRKKE
>lcl|124|lcl|HAPPENN&HAPPENN-RR90|lcl|subdataset_9|lcl|non-hemolytic
RAYCRRRFCVAR
>lcl|2995|lcl|HAPPENN&HAPPENN-RR90&HAPPENN-hard|lcl|subdataset_6|lcl|non-hemolytic|lcl|cTer
FLKLIPRKIVTAL
>lcl|2865|lcl|HAPPENN|lcl|subdataset_11|lcl|non-hemolytic
KKKLKFKLLQ
>lcl|1084|lcl|HAPPENN|lcl|subdataset_12|lcl|hemolytic
```

```
GKLEVLHSTKKFAKGFITGLTGQ
>lcl|2362|lcl|HAPPENN&HAPPENN-RR90|lcl|subdataset_4|lcl|non-hemolytic|lcl|cTer
KKIVQKIKDFLK
>lcl|4554|lcl|HAPPENN|lcl|subdataset_9|lcl|hemolytic|lcl|cTer
FLKGIVGMLGKLL
>lcl|332|lcl|HAPPENN|lcl|subdataset_7|lcl|hemolytic|lcl|cTer
GLFGVLAKVAAHVVPAIAEHF
>lcl|1382|lcl|HAPPENN&HAPPENN-RR90&HAPPENN-hard|lcl|subdataset_4|lcl|hemolytic
PKLLKKFLKKWIG
>lcl|3538|lcl|HAPPENN&HAPPENN-RR90&HAPPENN-hard|lcl|subdataset_11|lcl|hemolytic
FLPAVLKVAAHILPTAICAISRRC
>lcl|2991|lcl|HAPPENN&HAPPENN-RR90|lcl|subdataset_12|lcl|non-hemolytic|lcl|cTer
KIKGAIKWKGAIKIKGAI
>lcl|2378|lcl|HAPPENN&HAPPENN-RR90&HAPPENN-hard|lcl|subdataset_6|lcl|non-hemolytic|lcl|cTer
RLLRRLRRLLR
>lcl|2058|lcl|HAPPENN&HAPPENN-RR90|lcl|subdataset_7|lcl|non-hemolytic|lcl|cTer
YRAWRWAWRWR
>lcl|4253|lcl|HAPPENN|lcl|subdataset_4|lcl|hemolytic|lcl|cTer
FLFKLIPKVIKGLVKAIRK
>lcl|2516|lcl|HAPPENN&HAPPENN-RR90|lcl|subdataset_6|lcl|non-hemolytic|lcl|cTer
KKLTLWLTLKK
>lcl|4080|lcl|HAPPENN&HAPPENN-RR90&HAPPENN-hard|lcl|subdataset_4|lcl|non-hemolytic
FLAKAVAKAAAKALAKAL
>lcl|3476|lcl|HAPPENN|lcl|subdataset_11|lcl|hemolytic|lcl|cTer
FLGFLHHLF
>lcl|2782|lcl|HAPPENN&HAPPENN-RR90&HAPPENN-hard|lcl|subdataset_7|lcl|non-hemolytic
FLGALFKALSKVL
>lcl|3678|lcl|HAPPENN&HAPPENN-RR90&HAPPENN-hard|lcl|subdataset_10|lcl|non-hemolytic|lcl|cTer
IKWKAILDAVKKVI
>lcl|4010|lcl|HAPPENN&HAPPENN-RR90|lcl|subdataset_8|lcl|non-hemolytic|lcl|nTer|lcl|cTer
MKWKIGKKIGIGKFLHSAKKFN
>lcl|1417|lcl|HAPPENN|lcl|subdataset_5|lcl|hemolytic|lcl|cTer
INWLKLGKKMMSAI
>lcl|923|lcl|HAPPENN|lcl|subdataset_5|lcl|non-hemolytic|lcl|cTer
KKAAHVGKHVGKAALTHYL
>lcl|2957|lcl|HAPPENN&HAPPENN-hard|lcl|subdataset_11|lcl|non-hemolytic|lcl|cTer
GLLKRAKTLL
>lcl|3151|lcl|HAPPENN&HAPPENN-RR90|lcl|subdataset_11|lcl|non-hemolytic
DAAVEPELYHWGKVWLPN
>lcl|508|lcl|HAPPENN&HAPPENN-RR90&HAPPENN-hard|lcl|subdataset_10|lcl|hemolytic|lcl|cTer
GFKDLLKKAAKALVKTVLF
>lcl|4445|lcl|HAPPENN|lcl|subdataset_12|lcl|non-hemolytic
IKKILSKIKKWWK
>lcl|4161|lcl|HAPPENN|lcl|subdataset_12|lcl|hemolytic
KKLFKKILKYLAGPAKKLFKKILKYLAGPA
>lcl|3617|lcl|HAPPENN&HAPPENN-RR90&HAPPENN-hard|lcl|subdataset_8|lcl|hemolytic|lcl|cTer
IWLTALKFLGKNLGKHLAKQQLAKL
>lcl|2407|lcl|HAPPENN&HAPPENN-RR90&HAPPENN-hard|lcl|subdataset_6|lcl|non-hemolytic
AKAFKKAFEKLAAVVPFGGT
>lcl|3393|lcl|HAPPENN&HAPPENN-RR90&HAPPENN-hard|lcl|subdataset_10|lcl|hemolytic|lcl|cTer
RRFPFFPFKFPLI
>lcl|1411|lcl|HAPPENN|lcl|subdataset_6|lcl|hemolytic|lcl|cTer
INFLKLGKKILGAL
>lcl|1908|lcl|HAPPENN&HAPPENN-RR90|lcl|subdataset_5|lcl|non-hemolytic
TTSIRRRYQVSLIRRHRGKR
>lcl|3659|lcl|HAPPENN&HAPPENN-RR90&HAPPENN-hard|lcl|subdataset_11|lcl|hemolytic|lcl|cTer
KLALKLALKAWKLALKAA
>lcl|513|lcl|HAPPENN&HAPPENN-RR90&HAPPENN-hard|lcl|subdataset_8|lcl|non-hemolytic|lcl|cTer
KLLGPLLKIAAKVGSNLL
>lcl|3695|lcl|HAPPENN|lcl|subdataset_4|lcl|hemolytic|lcl|nTer|lcl|cTer
KWKLFKKIFKRIVQRIKDFLR
>lcl|2825|lcl|HAPPENN|lcl|subdataset_7|lcl|non-hemolytic|lcl|cTer
YCNGKRVCVCR
>lcl|2723|lcl|HAPPENN&HAPPENN-RR90&HAPPENN-hard|lcl|subdataset_12|lcl|hemolytic|lcl|cTer
```

```
GLLGLLGSVVSHVVPAIVGHF
>lcl|4480|lcl|HAPPENN|lcl|subdataset_11|lcl|hemolytic|lcl|cTer
GILSSLLKKLKKWIAK
>lcl|3897|lcl|HAPPENN|lcl|subdataset_12|lcl|hemolytic|lcl|cTer
GIWDTIKSMGKVFAGLILQNL
>lcl|4652|lcl|HAPPENN|lcl|subdataset_4|lcl|non-hemolytic|lcl|cTer
CRRWQWRC
>lcl|2259|lcl|HAPPENN&HAPPENN-RR90|lcl|subdataset_10|lcl|non-hemolytic
YYHFWHRGVTKRSLSPHRPRHSRLQR
>lcl|1478|lcl|HAPPENN&HAPPENN-RR90&HAPPENN-hard|lcl|subdataset_10|lcl|hemolytic
CGESCVWIPCTITALAGCKCKSKVCYNSIP
>lcl|3315|lcl|HAPPENN&HAPPENN-RR90|lcl|subdataset_4|lcl|non-hemolytic
APAHRSSTFPKWVTKTERGRQPLRS
>lcl|3144|lcl|HAPPENN|lcl|subdataset_6|lcl|non-hemolytic
FLPGLECVW
>lcl|969|lcl|HAPPENN&HAPPENN-RR90&HAPPENN-hard|lcl|subdataset_12|lcl|hemolytic|lcl|cTer
GWKSVFRKAKKVGKTVGGLALDHYL
>lcl|2909|lcl|HAPPENN&HAPPENN-RR90|lcl|subdataset_10|lcl|non-hemolytic
PRPPHPPRPPHPPRPPHPPRPPHPRL
>lcl|4157|lcl|HAPPENN|lcl|subdataset_6|lcl|hemolytic
KKLFKKILKYLKKLFKKILKYL
>lcl|3938|lcl|HAPPENN&HAPPENN-RR90&HAPPENN-hard|lcl|subdataset_12|lcl|hemolytic
WLRRIKAWLRRIKA
>lcl|2083|lcl|HAPPENN&HAPPENN-hard|lcl|subdataset_10|lcl|non-hemolytic|lcl|cTer
LRWLRRL
>lcl|1480|lcl|HAPPENN|lcl|subdataset_5|lcl|hemolytic
CGESCVWIPCLTSAVGCSCKSKVCYRNGIP
>lcl|4774|lcl|HAPPENN&HAPPENN-RR90&HAPPENN-hard|lcl|subdataset_7|lcl|hemolytic
FFGTLFKLGSKLIPGVMKLFSKKKER
>lcl|4689|lcl|HAPPENN&HAPPENN-hard|lcl|subdataset_8|lcl|non-hemolytic|lcl|cTer
FRFKFRFK
>lcl|1228|lcl|HAPPENN|lcl|subdataset_9|lcl|non-hemolytic
QKAVLDCLKAAGSSLSKAAITAI
>lcl|1189|lcl|HAPPENN&HAPPENN-hard|lcl|subdataset_9|lcl|non-hemolytic
GIGKLFLHAAKKFAKAFVAEKMNS
>lcl|2220|lcl|HAPPENN&HAPPENN-RR90&HAPPENN-hard|lcl|subdataset_9|lcl|non-hemolytic
RNCTWLFSTKLKLPA
>lcl|2252|lcl|HAPPENN&HAPPENN-RR90|lcl|subdataset_6|lcl|non-hemolytic|lcl|cTer
HKKHKKHKKHKKHKKHKKHKKHKK
>lcl|265|lcl|HAPPENN&HAPPENN-RR90&HAPPENN-hard|lcl|subdataset_8|lcl|non-hemolytic|lcl|cTer
ILPWKWPKLPWRR
>lcl|1309|lcl|HAPPENN|lcl|subdataset_10|lcl|hemolytic
ALWKTMLKKLGTMALHAGKAALGAAANTISQGTQ
>lcl|359|lcl|HAPPENN&HAPPENN-RR90&HAPPENN-hard|lcl|subdataset_7|lcl|hemolytic|lcl|nTer|lcl|cTer
KQLIRFLKRLDRNLWGLA
>lcl|707|lcl|HAPPENN|lcl|subdataset_11|lcl|non-hemolytic|lcl|cTer
LLPALISWIKRKRQQ
>lcl|3771|lcl|HAPPENN|lcl|subdataset_10|lcl|non-hemolytic
KKTWWKTWWTKWSQPKK
>lcl|3554|lcl|HAPPENN&HAPPENN-RR90&HAPPENN-hard|lcl|subdataset_7|lcl|hemolytic
GFMSKVANFAKKFAKGGVNAIMNQK
>lcl|4546|lcl|HAPPENN&HAPPENN-RR90&HAPPENN-hard|lcl|subdataset_6|lcl|hemolytic
FFVLKFLLKWAGKVGLEHLACKFKNWC
>lcl|1169|lcl|HAPPENN&HAPPENN-RR90&HAPPENN-hard|lcl|subdataset_7|lcl|non-hemolytic
CTCSWPVCTRNGLPVCGETCVGGTCNTPG
>lcl|2506|lcl|HAPPENN&HAPPENN-RR90|lcl|subdataset_12|lcl|non-hemolytic|lcl|cTer
GKKLGLWLGLKKG
>lcl|878|lcl|HAPPENN&HAPPENN-RR90&HAPPENN-hard|lcl|subdataset_10|lcl|hemolytic
GIGAILKVLATGLPTLISWIKNKRKQ
>lcl|4656|lcl|HAPPENN&HAPPENN-RR90&HAPPENN-hard|lcl|subdataset_11|lcl|hemolytic|lcl|cTer
FLSLIPHIVSGVASLAKHF
>lcl|1585|lcl|HAPPENN|lcl|subdataset_10|lcl|hemolytic|lcl|cTer
KIAGKIAAIAGKIAKIAGKIAKIA
>lcl|4184|lcl|HAPPENN&HAPPENN-hard|lcl|subdataset_9|lcl|non-hemolytic
SKKLFKKILKYLAGPAKFLHSAK
>lcl|4138|lcl|HAPPENN|lcl|subdataset_4|lcl|non-hemolytic
RWCVYAYRRVRGVLVRYRRCW
>lcl|4800|lcl|HAPPENN|lcl|subdataset_7|lcl|non-hemolytic
RLKKWMQKVIDRFGG
```

```
>lcl|3327|lcl|HAPPENN|lcl|subdataset_10|lcl|non-hemolytic|lcl|cTer
FKKLKKIANIINSIFKK
>lcl|3517|lcl|HAPPENN&HAPPENN-RR90&HAPPENN-hard|lcl|subdataset_12|lcl|non-hemolytic
FIHHIIGGLFSVGKHIHGLIHGH
>lcl|4618|lcl|HAPPENN&HAPPENN-hard|lcl|subdataset_5|lcl|non-hemolytic
GRRPRPRPRPWWWW
>lcl|2168|lcl|HAPPENN|lcl|subdataset_6|lcl|hemolytic|lcl|cTer
KWKVFKKIEKMARNIRNKIVK
>lcl|2914|lcl|HAPPENN|lcl|subdataset_6|lcl|non-hemolytic
LKKLLFKKKQ
>lcl|4013|lcl|HAPPENN|lcl|subdataset_6|lcl|hemolytic|lcl|cTer
GLLKKIKWLL
>lcl|628|lcl|HAPPENN&HAPPENN-RR90|lcl|subdataset_11|lcl|non-hemolytic|lcl|cTer
FKLFKKIPKFLHLAKKF
>lcl|3256|lcl|HAPPENN&HAPPENN-RR90&HAPPENN-hard|lcl|subdataset_11|lcl|hemolytic
FLPVIAGVAANFLPKLFCAISKKC
>lcl|3850|lcl|HAPPENN&HAPPENN-RR90|lcl|subdataset_9|lcl|non-hemolytic|lcl|cTer
RKKKLKVVKRLV
>lcl|1483|lcl|HAPPENN|lcl|subdataset_7|lcl|hemolytic
CGESCVYIPCLTSAIGCSCKSKVCYRNGIP
>lcl|966|lcl|HAPPENN&HAPPENN-RR90&HAPPENN-hard|lcl|subdataset_8|lcl|hemolytic|lcl|cTer
RSTEDIIKSISGGGFLNAMNA
>lcl|275|lcl|HAPPENN&HAPPENN-RR90|lcl|subdataset_10|lcl|non-hemolytic|lcl|cTer
RRWPLKPKKWPLI
>lcl|3829|lcl|HAPPENN&HAPPENN-RR90&HAPPENN-hard|lcl|subdataset_10|lcl|non-hemolytic|lcl|cTer
RKKRLKLLKRLL
>lcl|142|lcl|HAPPENN|lcl|subdataset_9|lcl|hemolytic
ACYCRRRFCVCVGR
>lcl|2488|lcl|HAPPENN&HAPPENN-RR90|lcl|subdataset_8|lcl|non-hemolytic|lcl|cTer
WIKAAAKAAAKIW
>lcl|4307|lcl|HAPPENN|lcl|subdataset_6|lcl|hemolytic
GIGKALHSAKKFGKAFVGEIMNS
>lcl|4286|lcl|HAPPENN&HAPPENN-hard|lcl|subdataset_10|lcl|non-hemolytic|lcl|cTer
GVFDIIKGAGKQLIAHAMGKIAEKV
>lcl|1663|lcl|HAPPENN|lcl|subdataset_12|lcl|hemolytic|lcl|cTer
FFPGIIKVAGAILPTAICAITKRC
>lcl|234|lcl|HAPPENN|lcl|subdataset_7|lcl|hemolytic
GLFGKLIKKFGRKAISYAVKKARGKH
>lcl|3687|lcl|HAPPENN&HAPPENN-hard|lcl|subdataset_6|lcl|non-hemolytic
ALGALFKVASKVL
>lcl|2869|lcl|HAPPENN|lcl|subdataset_11|lcl|non-hemolytic
KKKKLFLKLQ
>lcl|1444|lcl|HAPPENN&HAPPENN-hard|lcl|subdataset_6|lcl|non-hemolytic
GVVDILKGAAKDLAGHLATKVMNKL
>lcl|1831|lcl|HAPPENN&HAPPENN-RR90&HAPPENN-hard|lcl|subdataset_4|lcl|hemolytic
MMRVMRRKTKVIWEKKDFIGLYSID
>lcl|1439|lcl|HAPPENN|lcl|subdataset_11|lcl|hemolytic
RVKRVWPLVIRTVIAGYNLYRAI
>lcl|3048|lcl|HAPPENN&HAPPENN-RR90&HAPPENN-hard|lcl|subdataset_9|lcl|hemolytic|lcl|cTer
GKAMSLLKHILK
>lcl|4458|lcl|HAPPENN&HAPPENN-RR90&HAPPENN-hard|lcl|subdataset_11|lcl|hemolytic
WGHKLRSSWNKVKHAVKKGAGYASGACRVLGH
>lcl|1210|lcl|HAPPENN|lcl|subdataset_6|lcl|hemolytic|lcl|cTer
VLPLVGNLLNDLL
>lcl|3472|lcl|HAPPENN&HAPPENN-RR90&HAPPENN-hard|lcl|subdataset_6|lcl|hemolytic|lcl|cTer
RLGTALPALLKTLLAGLNG
>lcl|3228|lcl|HAPPENN&HAPPENN-hard|lcl|subdataset_9|lcl|non-hemolytic|lcl|cTer
ILGPVLGLDGNALGGLIKKI
>lcl|327|lcl|HAPPENN&HAPPENN-RR90&HAPPENN-hard|lcl|subdataset_11|lcl|hemolytic|lcl|cTer
INWLKLGKAIIDAL
>lcl|3161|lcl|HAPPENN&HAPPENN-RR90&HAPPENN-hard|lcl|subdataset_11|lcl|hemolytic
GFGSFLGSLFKTGLKIIPKLLPSIQQ
>lcl|3993|lcl|HAPPENN|lcl|subdataset_4|lcl|hemolytic|lcl|cTer
AKRHHGYKRKFH
>lcl|1203|lcl|HAPPENN&HAPPENN-RR90&HAPPENN-hard|lcl|subdataset_4|lcl|hemolytic|lcl|cTer
FLGAIAAALPHVINAVTNAL
>lcl|3498|lcl|HAPPENN|lcl|subdataset_10|lcl|hemolytic|lcl|cTer
ALWKNMLKGI
```

```
>lcl|1738|lcl|HAPPENN&HAPPENN-RR90&HAPPENN-hard|lcl|subdataset_8|lcl|non-hemolytic
RLLRKFFRKLKKSV
>lcl|338|lcl|HAPPENN&HAPPENN-RR90&HAPPENN-hard|lcl|subdataset_12|lcl|hemolytic
FLPILAGLAAKLVPKVFCSITKKC
>lcl|4356|lcl|HAPPENN&HAPPENN-hard|lcl|subdataset_4|lcl|non-hemolytic|lcl|cTer
GLLKRIKSLL
>lcl|3225|lcl|HAPPENN&HAPPENN-hard|lcl|subdataset_12|lcl|non-hemolytic|lcl|cTer
ISGPVLGLVGNALGGLIKKI
>lcl|4471|lcl|HAPPENN&HAPPENN-RR90&HAPPENN-hard|lcl|subdataset_12|lcl|non-hemolytic|lcl|cTer
GIMSSLMKKLKKIIKK
>lcl|4386|lcl|HAPPENN&HAPPENN-hard|lcl|subdataset_5|lcl|non-hemolytic
RAGLQFPVGRVHRLLRK
>lcl|3053|lcl|HAPPENN&HAPPENN-RR90&HAPPENN-hard|lcl|subdataset_4|lcl|hemolytic|lcl|cTer
GKWMHLLKHILK
>lcl|3128|lcl|HAPPENN&HAPPENN-RR90&HAPPENN-hard|lcl|subdataset_11|lcl|hemolytic
GIMNTVKDVATGVATHLLNMVKCKITGC
>lcl|1507|lcl|HAPPENN&HAPPENN-RR90&HAPPENN-hard|lcl|subdataset_11|lcl|hemolytic|lcl|cTer
FLPVILPVIGKLLSGIL
>lcl|3308|lcl|HAPPENN|lcl|subdataset_4|lcl|hemolytic|lcl|cTer
IKLSKETKDNLKKVLKGAIKGAIAVAKMV
>lcl|1171|lcl|HAPPENN&HAPPENN-RR90&HAPPENN-hard|lcl|subdataset_6|lcl|non-hemolytic
CSCKNKVCYRNGVIPCGESCVFIPCISTLLG
>lcl|782|lcl|HAPPENN&HAPPENN-RR90&HAPPENN-hard|lcl|subdataset_4|lcl|hemolytic
GLVTSLIKGAGKLLGGLFGSVTGGQS
>lcl|2649|lcl|HAPPENN&HAPPENN-RR90&HAPPENN-hard|lcl|subdataset_7|lcl|hemolytic|lcl|cTer
FLPIVGKLLSGLF
>lcl|4479|lcl|HAPPENN&HAPPENN-hard|lcl|subdataset_11|lcl|non-hemolytic|lcl|cTer
GILSSLLKKWKKIIAK
>lcl|3869|lcl|HAPPENN&HAPPENN-RR90&HAPPENN-hard|lcl|subdataset_10|lcl|hemolytic|lcl|cTer
LRFLKKALKKLF
>lcl|171|lcl|HAPPENN|lcl|subdataset_11|lcl|non-hemolytic|lcl|cTer
LRQSQFVGSR
>lcl|825|lcl|HAPPENN&HAPPENN-RR90|lcl|subdataset_8|lcl|non-hemolytic
WKSYVRRWRSRY
>lcl|3756|lcl|HAPPENN|lcl|subdataset_11|lcl|hemolytic|lcl|nTer|lcl|cTer
ALWMTLLKKVLKA
>lcl|3830|lcl|HAPPENN&HAPPENN-RR90|lcl|subdataset_7|lcl|non-hemolytic|lcl|cTer
RKKRVKLLKRLV
>lcl|4609|lcl|HAPPENN&HAPPENN-RR90|lcl|subdataset_6|lcl|non-hemolytic
GRKKRRQRRRPPQ
>lcl|4654|lcl|HAPPENN|lcl|subdataset_6|lcl|non-hemolytic|lcl|cTer
CRRWWRFC
>lcl|1903|lcl|HAPPENN|lcl|subdataset_5|lcl|non-hemolytic
GNNRPVYIPQPKPPHPRL
>lcl|2236|lcl|HAPPENN|lcl|subdataset_4|lcl|hemolytic|lcl|cTer
FKRWVQRWKRFLR
>lcl|3669|lcl|HAPPENN|lcl|subdataset_5|lcl|non-hemolytic|lcl|cTer
GVVVRWGRVIVRGVRR
>lcl|3863|lcl|HAPPENN|lcl|subdataset_8|lcl|non-hemolytic|lcl|cTer
RKKRKKLLKRLL
>lcl|2383|lcl|HAPPENN&HAPPENN-RR90|lcl|subdataset_9|lcl|non-hemolytic|lcl|cTer
RRWRWWWRWRR
>lcl|3341|lcl|HAPPENN|lcl|subdataset_8|lcl|non-hemolytic|lcl|cTer
WKSYVRRWRSRY
>lcl|3655|lcl|HAPPENN|lcl|subdataset_10|lcl|non-hemolytic|lcl|cTer
ILPWKKPKKPKRR
>lcl|1412|lcl|HAPPENN|lcl|subdataset_5|lcl|hemolytic|lcl|cTer
INWKKLGKKILGAL
>lcl|4442|lcl|HAPPENN&HAPPENN-RR90&HAPPENN-hard|lcl|subdataset_12|lcl|non-hemolytic|lcl|cTer
WLNAKRHHGYKRKFH
>lcl|1529|lcl|HAPPENN&HAPPENN-RR90&HAPPENN-hard|lcl|subdataset_11|lcl|hemolytic
GLPVCGETCTLGTVYTQGCTCSWPICKRN
>lcl|2603|lcl|HAPPENN&HAPPENN-RR90&HAPPENN-hard|lcl|subdataset_11|lcl|non-hemolytic
SIRDKIKTIAIDLAKSAGTGVLKTLICKLNKSC
>lcl|4423|lcl|HAPPENN|lcl|subdataset_9|lcl|non-hemolytic|lcl|cTer
GKLWLKGGKLWLKG
>lcl|3997|lcl|HAPPENN|lcl|subdataset_7|lcl|hemolytic|lcl|cTer
```

```
KKVLKAAA
>lcl|1599|lcl|HAPPENN|lcl|subdataset_6|lcl|non-hemolytic|lcl|cTer
KIAGKIAKIAGKIAKIA
>lcl|2269|lcl|HAPPENN&HAPPENN-RR90&HAPPENN-hard|lcl|subdataset_9|lcl|hemolytic
GLPVCGETCVGGTCNTPGCTCSWWPVCTRN
>lcl|2228|lcl|HAPPENN&HAPPENN-RR90|lcl|subdataset_10|lcl|non-hemolytic|lcl|cTer
IHKFWRRWFKHI
>lcl|1477|lcl|HAPPENN&HAPPENN-RR90&HAPPENN-hard|lcl|subdataset_5|lcl|hemolytic
CGESCVWIPCISAAVGCSCKSKVCYKNGTLP
>lcl|2737|lcl|HAPPENN&HAPPENN-RR90|lcl|subdataset_11|lcl|non-hemolytic
GATYAKKIIKTITKIATTAW
>lcl|4473|lcl|HAPPENN&HAPPENN-hard|lcl|subdataset_12|lcl|non-hemolytic|lcl|cTer
GILKSLLKKLKKIIAK
>lcl|4830|lcl|HAPPENN&HAPPENN-RR90&HAPPENN-hard|lcl|subdataset_5|lcl|non-hemolytic|lcl|cTer
FFWHHIGHALDAAKRVHGMLSG
>lcl|1706|lcl|HAPPENN&HAPPENN-RR90&HAPPENN-hard|lcl|subdataset_11|lcl|hemolytic|lcl|cTer
YKQCHKKGGKKGSG
>lcl|2337|lcl|HAPPENN&HAPPENN-RR90&HAPPENN-hard|lcl|subdataset_6|lcl|non-hemolytic|lcl|cTer
FLPFFAACAITRKCGK
>lcl|6|lcl|HAPPENN|lcl|subdataset_5|lcl|hemolytic
ALWKTLLKKVLKAAAKAALKAVLVGANA
>lcl|3251|lcl|HAPPENN|lcl|subdataset_8|lcl|hemolytic
FLPIIAGMAAKVICAITKKC
>lcl|2551|lcl|HAPPENN&HAPPENN-hard|lcl|subdataset_12|lcl|non-hemolytic|lcl|nTer|lcl|cTer
KWKSFLKTFKSASKTVLHTALKAISS
>lcl|4761|lcl|HAPPENN&HAPPENN-RR90&HAPPENN-hard|lcl|subdataset_6|lcl|hemolytic|lcl|cTer
FLGALWNVWKSVF
>lcl|4582|lcl|HAPPENN&HAPPENN-RR90&HAPPENN-hard|lcl|subdataset_11|lcl|non-hemolytic
VWRKWRRFWKR
>lcl|973|lcl|HAPPENN&HAPPENN-RR90&HAPPENN-hard|lcl|subdataset_9|lcl|hemolytic|lcl|cTer
GFWGKLFKLGLHGIGLLHLHL
>lcl|455|lcl|HAPPENN|lcl|subdataset_7|lcl|non-hemolytic
DSHEKRHHGYKRKFHEKHHSHRGY
>lcl|4281|lcl|HAPPENN&HAPPENN-hard|lcl|subdataset_4|lcl|non-hemolytic
KGIRGYKGGYCKGAFKQTCKCY
>lcl|3935|lcl|HAPPENN&HAPPENN-RR90|lcl|subdataset_7|lcl|non-hemolytic|lcl|cTer
IKKWWSKIKKLLK
>lcl|883|lcl|HAPPENN&HAPPENN-RR90&HAPPENN-hard|lcl|subdataset_10|lcl|hemolytic
VGTIIKIIKAIIDIFAK
>lcl|4712|lcl|HAPPENN|lcl|subdataset_11|lcl|hemolytic
FIFHIIKGLFHAGKMIHGLVT
>lcl|2969|lcl|HAPPENN|lcl|subdataset_7|lcl|hemolytic|lcl|cTer
GLLKLIKTLL
>lcl|3909|lcl|HAPPENN|lcl|subdataset_4|lcl|non-hemolytic
RVVRQWPIGRVVRRVVRRVVRF
>lcl|4452|lcl|HAPPENN&HAPPENN-hard|lcl|subdataset_10|lcl|non-hemolytic|lcl|cTer
SILGKLWKGVKSIF
>lcl|1935|lcl|HAPPENN&HAPPENN-RR90&HAPPENN-hard|lcl|subdataset_4|lcl|hemolytic|lcl|cTer
KLALKAAAKAWKAAAKAA
>lcl|3788|lcl|HAPPENN&HAPPENN-RR90|lcl|subdataset_5|lcl|non-hemolytic|lcl|cTer
WGIRRILKYGKRS
>lcl|3006|lcl|HAPPENN&HAPPENN-RR90|lcl|subdataset_9|lcl|non-hemolytic
RCVCRRGVCRCVCRRGVC
>lcl|598|lcl|HAPPENN&HAPPENN-RR90&HAPPENN-hard|lcl|subdataset_4|lcl|non-hemolytic
IKIPPIVKDTLKKVAKGVLSTIAGALST
>lcl|4204|lcl|HAPPENN&HAPPENN-RR90|lcl|subdataset_5|lcl|non-hemolytic
TLIWEFYHQILDEYNKENKG
>lcl|1305|lcl|HAPPENN&HAPPENN-hard|lcl|subdataset_4|lcl|non-hemolytic
ISSPLFKTLLSAVGSALSSSGGQE
>lcl|919|lcl|HAPPENN|lcl|subdataset_6|lcl|hemolytic|lcl|cTer
KWLRRVWRWWR
>lcl|3267|lcl|HAPPENN&HAPPENN-RR90&HAPPENN-hard|lcl|subdataset_8|lcl|hemolytic
GLPTCGETCTLGTCYVPDCSCSWPICMKN
>lcl|3837|lcl|HAPPENN|lcl|subdataset_12|lcl|hemolytic
GLNALKKVFQGIHEAIKLINKHVQ
>lcl|1531|lcl|HAPPENN&HAPPENN-hard|lcl|subdataset_12|lcl|non-hemolytic
AKKVFKRLGIGAVLKVLTWG
```

```
>lcl|2056|lcl|HAPPENN&HAPPENN-RR90&HAPPENN-hard|lcl|subdataset_5|lcl|non-hemolytic
YKAWRWAWRWK
>lcl|218|lcl|HAPPENN|lcl|subdataset_6|lcl|hemolytic
RFGRFLRKIRRFRPKVTITIQGSARFG
>lcl|3541|lcl|HAPPENN|lcl|subdataset_8|lcl|hemolytic
GVLDTLKNVAIGVAKGAGTGVLKALLCQLDKSC
>lcl|109|lcl|HAPPENN|lcl|subdataset_11|lcl|hemolytic
RGGGLCYCRRRFCVCVGR
>lcl|1847|lcl|HAPPENN&HAPPENN-RR90|lcl|subdataset_4|lcl|non-hemolytic
KKFGGGKTERARKAMAVE
>lcl|3291|lcl|HAPPENN|lcl|subdataset_7|lcl|non-hemolytic|lcl|cTer
GVVDILKGAAKDIAGHLASKVM
>lcl|3447|lcl|HAPPENN|lcl|subdataset_4|lcl|hemolytic
GLPVCGETCTLGTCYTQGCTCSWPICKRN
>lcl|343|lcl|HAPPENN&HAPPENN-RR90&HAPPENN-hard|lcl|subdataset_12|lcl|hemolytic
QRSVSNAATRVCRTGRSRWRDVCRNFMRR
>lcl|2975|lcl|HAPPENN|lcl|subdataset_9|lcl|hemolytic|lcl|cTer
GLLKRIKVLL
>lcl|4318|lcl|HAPPENN&HAPPENN-RR90&HAPPENN-hard|lcl|subdataset_7|lcl|hemolytic|lcl|cTer
LPFFLLSLIPSAISAIKKI
>lcl|2913|lcl|HAPPENN|lcl|subdataset_10|lcl|non-hemolytic
KLKLLFKKKQ
>lcl|4343|lcl|HAPPENN|lcl|subdataset_4|lcl|non-hemolytic
WGIHRILKYGKPS
>lcl|1520|lcl|HAPPENN&HAPPENN-hard|lcl|subdataset_8|lcl|non-hemolytic
GFMKYIGPLIPHAVKAISDLI
>lcl|4434|lcl|HAPPENN&HAPPENN-RR90|lcl|subdataset_4|lcl|non-hemolytic
IKKWLSKIKKLLK
>lcl|2626|lcl|HAPPENN|lcl|subdataset_9|lcl|hemolytic|lcl|cTer
GIGAVLGVLTTGLPALISWIKRKRQQ
>lcl|3708|lcl|HAPPENN|lcl|subdataset_4|lcl|non-hemolytic|lcl|nTer|lcl|cTer
KKWKKWKKWKK
>lcl|4686|lcl|HAPPENN|lcl|subdataset_9|lcl|non-hemolytic|lcl|cTer
IIRKIIRK
>lcl|817|lcl|HAPPENN|lcl|subdataset_9|lcl|hemolytic
FVDLKKIANIINSIFGK
>lcl|663|lcl|HAPPENN|lcl|subdataset_10|lcl|non-hemolytic
GSKKPVPIIYCNRRTKCQRM
>lcl|2160|lcl|HAPPENN&HAPPENN-RR90&HAPPENN-hard|lcl|subdataset_8|lcl|hemolytic|lcl|cTer
GFKRIVQKIRDFLRNLV
>lcl|1564|lcl|HAPPENN&HAPPENN-RR90&HAPPENN-hard|lcl|subdataset_7|lcl|hemolytic|lcl|cTer
MLLKKLLKKLLKKM
>lcl|3979|lcl|HAPPENN|lcl|subdataset_9|lcl|non-hemolytic|lcl|cTer
GVVDILKGAAKDAAGHLASKVMNKL
>lcl|3175|lcl|HAPPENN&HAPPENN-RR90|lcl|subdataset_11|lcl|non-hemolytic
GVCRCVCRRGVCRCVCRR
>lcl|4347|lcl|HAPPENN&HAPPENN-RR90|lcl|subdataset_9|lcl|non-hemolytic|lcl|cTer
KKKKWKLFKKIGIGKFL
>lcl|1921|lcl|HAPPENN&HAPPENN-RR90|lcl|subdataset_9|lcl|non-hemolytic
DATPHAALFFTVKDHTAGDN
>lcl|1267|lcl|HAPPENN|lcl|subdataset_4|lcl|hemolytic
FFGHLFKLATKIIPSLFQ
>lcl|4164|lcl|HAPPENN&HAPPENN-RR90&HAPPENN-hard|lcl|subdataset_11|lcl|hemolytic
KKLFKKILKYLAGPALYKLIKKFLKKKDEL
>lcl|4777|lcl|HAPPENN&HAPPENN-RR90|lcl|subdataset_5|lcl|non-hemolytic|lcl|cTer
WGEAFSAGVHRLANGGNG
>lcl|958|lcl|HAPPENN|lcl|subdataset_4|lcl|non-hemolytic
RRWQWRMKKLG
>lcl|1819|lcl|HAPPENN|lcl|subdataset_8|lcl|hemolytic
GIMDTLKNLAKTAGKGALQSLLNKASCKLSGQC
>lcl|2947|lcl|HAPPENN&HAPPENN-hard|lcl|subdataset_10|lcl|non-hemolytic
GILDTLKQFAKGVGKDLVKGAAQGVL
>lcl|3609|lcl|HAPPENN|lcl|subdataset_4|lcl|non-hemolytic
KPYLKRFQVYYKGRMWC
>lcl|1492|lcl|HAPPENN&HAPPENN-RR90&HAPPENN-hard|lcl|subdataset_11|lcl|hemolytic
VCGETCVGGTCNTPGCTCSWPVCTRDGLP
>lcl|3352|lcl|HAPPENN&HAPPENN-RR90|lcl|subdataset_12|lcl|non-hemolytic
KLLKQWPIGKLLKKLLKKLLK
>lcl|4364|lcl|HAPPENN&HAPPENN-RR90&HAPPENN-hard|lcl|subdataset_5|lcl|hemolytic
RIRFPWPWRWPWWRRVRG
```

```
>lcl|3347|lcl|HAPPENN|lcl|subdataset_10|lcl|hemolytic
GIIKKIIKKIIKKI
>lcl|740|lcl|HAPPENN&HAPPENN-RR90&HAPPENN-hard|lcl|subdataset_6|lcl|non-hemolytic|lcl|cTer
ILGKILEGIKSLF
>lcl|664|lcl|HAPPENN|lcl|subdataset_12|lcl|non-hemolytic
GSKKPVPIIYCNRRKCQRM
>lcl|3407|lcl|HAPPENN|lcl|subdataset_9|lcl|hemolytic|lcl|cTer
HFLGKLVNLAKKIL
>lcl|3945|lcl|HAPPENN|lcl|subdataset_4|lcl|non-hemolytic
FFHHIKRGIVHVGKTIHRLVTG
>lcl|4315|lcl|HAPPENN&HAPPENN-RR90|lcl|subdataset_5|lcl|non-hemolytic|lcl|cTer
GKPYGNAYLGLRPKYSRWL
>lcl|1379|lcl|HAPPENN&HAPPENN-RR90|lcl|subdataset_9|lcl|non-hemolytic
KGRGKQGGKVRAKAKTRSS
>lcl|3150|lcl|HAPPENN&HAPPENN-RR90|lcl|subdataset_8|lcl|non-hemolytic
VFLGNIVSMGKKI
>lcl|3022|lcl|HAPPENN|lcl|subdataset_4|lcl|hemolytic|lcl|cTer
GMWSKILGHLKR
>lcl|3694|lcl|HAPPENN&HAPPENN-RR90|lcl|subdataset_11|lcl|non-hemolytic|lcl|nTer|lcl|cTer
RRIRPRPPRLPRPRPRPLPFPRPGPRPIPRPLPFP
>lcl|316|lcl|HAPPENN|lcl|subdataset_8|lcl|hemolytic|lcl|cTer
KWKKLLKKLLKLLKKLLK
>lcl|4337|lcl|HAPPENN|lcl|subdataset_6|lcl|non-hemolytic|lcl|cTer
GIHDILKYGKAS
>lcl|4538|lcl|HAPPENN|lcl|subdataset_7|lcl|hemolytic|lcl|cTer
FFHHIFRGVVHVGKTIHRLVTG
>lcl|786|lcl|HAPPENN|lcl|subdataset_11|lcl|hemolytic|lcl|cTer
KWKKLLKKLLKLL
>lcl|1399|lcl|HAPPENN&HAPPENN-RR90&HAPPENN-hard|lcl|subdataset_12|lcl|hemolytic|lcl|cTer
IDWKKIFEKVKNLV
>lcl|877|lcl|HAPPENN|lcl|subdataset_12|lcl|hemolytic
GIGAVLKVLTTGLPALISWI
>lcl|3309|lcl|HAPPENN|lcl|subdataset_6|lcl|hemolytic|lcl|cTer
IKLSPKTKDNLKKVLKGAIKGAIAVAKMV
>lcl|3305|lcl|HAPPENN&HAPPENN-hard|lcl|subdataset_6|lcl|non-hemolytic|lcl|cTer
GLKKIFKAGLGSLKKGIAAHVAS
>lcl|3525|lcl|HAPPENN&HAPPENN-RR90|lcl|subdataset_9|lcl|non-hemolytic
YSKSLPLSVLNP
>lcl|3550|lcl|HAPPENN&HAPPENN-RR90&HAPPENN-hard|lcl|subdataset_5|lcl|hemolytic
GWASSIGSILGKFAKGGAQAFLQPK
>lcl|3081|lcl|HAPPENN|lcl|subdataset_10|lcl|hemolytic|lcl|cTer
VNWKKILKKIIKVVK
>lcl|2807|lcl|HAPPENN|lcl|subdataset_8|lcl|non-hemolytic|lcl|cTer
GACRAIRHIPRRIR
>lcl|523|lcl|HAPPENN&HAPPENN-RR90&HAPPENN-hard|lcl|subdataset_5|lcl|hemolytic|lcl|cTer
AAGLAMLFLGILSAAGSTMGARA
>lcl|3486|lcl|HAPPENN|lcl|subdataset_6|lcl|non-hemolytic
KVANTVQKLKRKAKNAV
>lcl|3275|lcl|HAPPENN&HAPPENN-RR90&HAPPENN-hard|lcl|subdataset_4|lcl|hemolytic
IPCGESCVWIPCISGMFGCSCKDKVCYS
>lcl|1605|lcl|HAPPENN|lcl|subdataset_5|lcl|hemolytic
GWLDVAKKIGKAAFNVAKNFL
>lcl|4446|lcl|HAPPENN|lcl|subdataset_11|lcl|non-hemolytic
IKKIWSKIKKWWK
>lcl|3940|lcl|HAPPENN|lcl|subdataset_9|lcl|non-hemolytic
FAHHIFRGIVHVGKTIHRLVTG
>lcl|2687|lcl|HAPPENN&HAPPENN-hard|lcl|subdataset_6|lcl|non-hemolytic|lcl|cTer
LKIAKGIIKSL
>lcl|3847|lcl|HAPPENN|lcl|subdataset_7|lcl|non-hemolytic
GLNALKKVFQGIHEAIKLFNNHVQ
>lcl|1432|lcl|HAPPENN&HAPPENN-RR90&HAPPENN-hard|lcl|subdataset_10|lcl|hemolytic|lcl|cTer
NIWKKIASIAKEVLKAL
>lcl|3836|lcl|HAPPENN|lcl|subdataset_7|lcl|hemolytic
GLNALKKVFQGIHKAIKLINNHVQ
>lcl|4202|lcl|HAPPENN&HAPPENN-RR90&HAPPENN-hard|lcl|subdataset_11|lcl|hemolytic|lcl|cTer
FLPLLFGALSTLLPKIF
>lcl|615|lcl|HAPPENN&HAPPENN-RR90|lcl|subdataset_5|lcl|non-hemolytic
DFKDWMKTAGEWLKKKGPGILKAAMAAAT
```

```
>lcl|2964|lcl|HAPPENN|lcl|subdataset_4|lcl|non-hemolytic|lcl|cTer
GLLKRIK
>lcl|1032|lcl|HAPPENN&HAPPENN-RR90|lcl|subdataset_6|lcl|non-hemolytic
ENAEEDIVLMENLFCSYIVGSADSFWT
>lcl|1163|lcl|HAPPENN&HAPPENN-hard|lcl|subdataset_8|lcl|non-hemolytic|lcl|cTer
LKLIPRKIVTAL
>lcl|2285|lcl|HAPPENN&HAPPENN-hard|lcl|subdataset_4|lcl|non-hemolytic
GFWSSVWDGAKNVGTAI
>lcl|387|lcl|HAPPENN|lcl|subdataset_12|lcl|hemolytic|lcl|cTer
KWKKLLKKLLKLLKKLLKKLKKLLK
>lcl|3355|lcl|HAPPENN|lcl|subdataset_4|lcl|non-hemolytic|lcl|cTer
KKFWEDDG
>lcl|3417|lcl|HAPPENN|lcl|subdataset_4|lcl|hemolytic
KKLFKKILKFL
>lcl|2651|lcl|HAPPENN&HAPPENN-RR90&HAPPENN-hard|lcl|subdataset_6|lcl|hemolytic|lcl|cTer
FLPFVGNLLNGLL
>lcl|4165|lcl|HAPPENN|lcl|subdataset_4|lcl|hemolytic
GKKLFKKILKYLAGPAKKLFKKILKYLKDEL
>lcl|3459|lcl|HAPPENN|lcl|subdataset_6|lcl|hemolytic|lcl|cTer
GLVGTLLGHIGKAILS
>lcl|2448|lcl|HAPPENN&HAPPENN-RR90|lcl|subdataset_9|lcl|non-hemolytic
HLNKRVQRLLIRWLRWLK
>lcl|2780|lcl|HAPPENN&HAPPENN-RR90&HAPPENN-hard|lcl|subdataset_8|lcl|non-hemolytic
FLGAVFKALSKLL
>lcl|1674|lcl|HAPPENN|lcl|subdataset_9|lcl|hemolytic|lcl|cTer
FRRPFKWFRRFFKFF
>lcl|3877|lcl|HAPPENN&HAPPENN-RR90|lcl|subdataset_12|lcl|non-hemolytic|lcl|cTer
LRKLKKILKKLF
>lcl|1527|lcl|HAPPENN&HAPPENN-RR90&HAPPENN-hard|lcl|subdataset_6|lcl|hemolytic|lcl|cTer
IPPFIKKVLTTVF
>lcl|1490|lcl|HAPPENN&HAPPENN-RR90&HAPPENN-hard|lcl|subdataset_6|lcl|hemolytic
VCGETCFGGTCNTPGCSCTWPICTRDGLP
>lcl|2497|lcl|HAPPENN&HAPPENN-RR90&HAPPENN-hard|lcl|subdataset_12|lcl|hemolytic|lcl|cTer
GIWKKWIKKWLKKLKNLF
>lcl|3179|lcl|HAPPENN&HAPPENN-RR90|lcl|subdataset_6|lcl|non-hemolytic
NALSMPRNKCNRALMCFG
>lcl|4041|lcl|HAPPENN&HAPPENN-RR90&HAPPENN-hard|lcl|subdataset_10|lcl|non-hemolytic
SIPCGESCVFIPCTVTALLGCSCKSKVCYKN
>lcl|2898|lcl|HAPPENN|lcl|subdataset_9|lcl|non-hemolytic
KKLKLFLKKQ
>lcl|1014|lcl|HAPPENN&HAPPENN-hard|lcl|subdataset_8|lcl|non-hemolytic
RLRKAVRLIK
>lcl|706|lcl|HAPPENN|lcl|subdataset_8|lcl|non-hemolytic|lcl|cTer
GLPALISWIKRKRQQ
>lcl|4274|lcl|HAPPENN&HAPPENN-RR90&HAPPENN-hard|lcl|subdataset_12|lcl|hemolytic
KWKLFKKIAKKFAKAFVAEIM
>lcl|2925|lcl|HAPPENN|lcl|subdataset_10|lcl|hemolytic
KLLKKFKKLQ
>lcl|1406|lcl|HAPPENN&HAPPENN-RR90&HAPPENN-hard|lcl|subdataset_6|lcl|hemolytic|lcl|cTer
INWKKIFESVKNLV
>lcl|423|lcl|HAPPENN&HAPPENN-RR90&HAPPENN-hard|lcl|subdataset_6|lcl|non-hemolytic|lcl|cTer
HFLLTLVNKAKKIL
>lcl|1845|lcl|HAPPENN&HAPPENN-RR90|lcl|subdataset_12|lcl|non-hemolytic
TSFLNELNKYNEKKFI
>lcl|253|lcl|HAPPENN&HAPPENN-RR90&HAPPENN-hard|lcl|subdataset_5|lcl|hemolytic|lcl|cTer
INWKKLLDAAKQIL
>lcl|4614|lcl|HAPPENN|lcl|subdataset_12|lcl|non-hemolytic|lcl|nTer|lcl|cTer
GFRKFHKFWA
>lcl|2053|lcl|HAPPENN|lcl|subdataset_5|lcl|non-hemolytic
FKARRWAWRMK
>lcl|1948|lcl|HAPPENN&HAPPENN-RR90&HAPPENN-hard|lcl|subdataset_5|lcl|non-hemolytic
FLPLLLAGLPSFLCLVFKKC
>lcl|4531|lcl|HAPPENN&HAPPENN-RR90&HAPPENN-hard|lcl|subdataset_7|lcl|hemolytic|lcl|cTer
FRRFRKWFRRFLKLF
>lcl|1316|lcl|HAPPENN&HAPPENN-RR90&HAPPENN-hard|lcl|subdataset_12|lcl|non-hemolytic
GIMSIVKDVAKTAAKEAAKGALSTLSCKLAKTC
>lcl|1842|lcl|HAPPENN&HAPPENN-hard|lcl|subdataset_4|lcl|non-hemolytic
```

```
NPEKALEKLIAIQKAIKGMLNGWFTGVGFRRKR
>lcl|4018|lcl|HAPPENN&HAPPENN-RR90|lcl|subdataset_11|lcl|non-hemolytic|lcl|nTer|lcl|cTer
VDKPDYRPRPWPRPN
>lcl|1895|lcl|HAPPENN&HAPPENN-RR90|lcl|subdataset_10|lcl|non-hemolytic
KQKLAKLKAKLQKLKQKLAKL
>lcl|1574|lcl|HAPPENN|lcl|subdataset_11|lcl|hemolytic|lcl|cTer
ALWKTMLKKLGTMAL
>lcl|2076|lcl|HAPPENN&HAPPENN-RR90&HAPPENN-hard|lcl|subdataset_10|lcl|non-hemolytic
KWKIFKKIEKVGRNIRNGIIKAVAVLGEAKAL
>lcl|4229|lcl|HAPPENN&HAPPENN-RR90|lcl|subdataset_11|lcl|non-hemolytic|lcl|cTer
IRIRCRRRFCRIRI
>lcl|4682|lcl|HAPPENN&HAPPENN-RR90&HAPPENN-hard|lcl|subdataset_12|lcl|hemolytic
IWSFLIKAATKLLGVGSLFGGGKKDS
>lcl|4354|lcl|HAPPENN&HAPPENN-hard|lcl|subdataset_9|lcl|non-hemolytic|lcl|cTer
GLLTRIKTLL
>lcl|233|lcl|HAPPENN&HAPPENN-hard|lcl|subdataset_12|lcl|non-hemolytic|lcl|cTer
LLKLLKWLLKLLK
>lcl|154|lcl|HAPPENN|lcl|subdataset_7|lcl|hemolytic
RGGRLCYCRRRFCVCW
>lcl|1441|lcl|HAPPENN|lcl|subdataset_7|lcl|hemolytic
VWPLVIRTVIAGYNLYRAIKKK
>lcl|4411|lcl|HAPPENN|lcl|subdataset_11|lcl|hemolytic|lcl|cTer
RWFKIQMQIRRWKNKK
>lcl|4335|lcl|HAPPENN&HAPPENN-RR90&HAPPENN-hard|lcl|subdataset_11|lcl|non-hemolytic|lcl|cTer
GIHDILKYAKPS
>lcl|4436|lcl|HAPPENN&HAPPENN-RR90|lcl|subdataset_11|lcl|non-hemolytic
IKKILSKIKKWLK
>lcl|2558|lcl|HAPPENN&HAPPENN-RR90&HAPPENN-hard|lcl|subdataset_12|lcl|hemolytic|lcl|cTer
FIGAIARLLSKIF
>lcl|3571|lcl|HAPPENN&HAPPENN-RR90&HAPPENN-hard|lcl|subdataset_4|lcl|hemolytic|lcl|cTer
GLLPLLSLLGKLL
>lcl|3497|lcl|HAPPENN|lcl|subdataset_11|lcl|hemolytic|lcl|cTer
ALWKNMLKGIGKLAGK
>lcl|2789|lcl|HAPPENN&HAPPENN-RR90|lcl|subdataset_10|lcl|non-hemolytic
FAGAAFKALSKLL
>lcl|3646|lcl|HAPPENN&HAPPENN-RR90&HAPPENN-hard|lcl|subdataset_7|lcl|hemolytic
FLPVIASVAAKVLPKVFCFITKKC
>lcl|3950|lcl|HAPPENN&HAPPENN-RR90&HAPPENN-hard|lcl|subdataset_9|lcl|non-hemolytic|lcl|nTer|lcl|cTer
GWQLLALHLQLLGLHLLQLALLQRRR
>lcl|4743|lcl|HAPPENN|lcl|subdataset_4|lcl|non-hemolytic|lcl|cTer
RKRWWWWFR
>lcl|1900|lcl|HAPPENN&HAPPENN-RR90&HAPPENN-hard|lcl|subdataset_5|lcl|hemolytic|lcl|cTer
AMWKDVLKKIGTVALHAGKAALGAVADTISQ
>lcl|2572|lcl|HAPPENN&HAPPENN-RR90|lcl|subdataset_11|lcl|non-hemolytic
FMPILSCSRFKRC
>lcl|725|lcl|HAPPENN&HAPPENN-RR90|lcl|subdataset_12|lcl|non-hemolytic|lcl|nTer|lcl|cTer
CVKVSVKVGSGVKVSVKVC
>lcl|2684|lcl|HAPPENN&HAPPENN-RR90|lcl|subdataset_4|lcl|non-hemolytic|lcl|cTer
KISKRILTGKK
>lcl|4785|lcl|HAPPENN|lcl|subdataset_8|lcl|hemolytic|lcl|cTer
GVVVRVGRVVVRWV
>lcl|626|lcl|HAPPENN&HAPPENN-RR90&HAPPENN-hard|lcl|subdataset_12|lcl|hemolytic|lcl|cTer
KWKLFKKISKFLHLAKKF
>lcl|2467|lcl|HAPPENN&HAPPENN-hard|lcl|subdataset_8|lcl|non-hemolytic|lcl|cTer
INWKKIKKKVKGML
>lcl|710|lcl|HAPPENN|lcl|subdataset_5|lcl|hemolytic|lcl|cTer
GLSALISWIKRKRQQ
>lcl|808|lcl|HAPPENN&HAPPENN-RR90|lcl|subdataset_11|lcl|non-hemolytic
KWCFCVCYRGICYCRCRG
>lcl|3527|lcl|HAPPENN&HAPPENN-RR90&HAPPENN-hard|lcl|subdataset_10|lcl|non-hemolytic|lcl|cTer
FIPLVSGLFSRLL
>lcl|3484|lcl|HAPPENN|lcl|subdataset_5|lcl|non-hemolytic
IASKVANTVQKLKRKAKNAV
>lcl|2979|lcl|HAPPENN|lcl|subdataset_12|lcl|non-hemolytic|lcl|cTer
```

```
GLLKRIKNLL
>lcl|1438|lcl|HAPPENN|lcl|subdataset_5|lcl|non-hemolytic
RVKRVWPLVIRTVIA
>lcl|4637|lcl|HAPPENN&HAPPENN-RR90&HAPPENN-hard|lcl|subdataset_6|lcl|hemolytic|lcl|cTer
GFLDVVKHIGKAALGAVTHLINQ
>lcl|4319|lcl|HAPPENN|lcl|subdataset_9|lcl|hemolytic|lcl|cTer
FFLLSLIPSAISAIKKI
>lcl|2090|lcl|HAPPENN|lcl|subdataset_11|lcl|hemolytic|lcl|cTer
ALWKKILKNAGKAALNKINQIVQ
>lcl|4275|lcl|HAPPENN|lcl|subdataset_12|lcl|hemolytic
FKRIVQRIKDFLRGIGKFLHSAKKF
>lcl|1270|lcl|HAPPENN|lcl|subdataset_10|lcl|hemolytic|lcl|cTer
GFLSILKKVLKKVMAHMK
>lcl|3688|lcl|HAPPENN|lcl|subdataset_8|lcl|hemolytic
WLGALFKVASKVL
>lcl|2146|lcl|HAPPENN&HAPPENN-RR90&HAPPENN-hard|lcl|subdataset_8|lcl|hemolytic
GAALAGLAKILCLWAKEFTGAFKKLNKKFAMKKK
>lcl|4687|lcl|HAPPENN|lcl|subdataset_6|lcl|non-hemolytic|lcl|cTer
VRVKVRVK
>lcl|2412|lcl|HAPPENN&HAPPENN-RR90&HAPPENN-hard|lcl|subdataset_4|lcl|hemolytic
FIVPSIFLLKKAFCIALKKC
>lcl|1788|lcl|HAPPENN&HAPPENN-RR90&HAPPENN-hard|lcl|subdataset_4|lcl|non-hemolytic
ASENGKCNLLCLVKKKLRAVGNVIKTVVGKIA
>lcl|3975|lcl|HAPPENN|lcl|subdataset_11|lcl|non-hemolytic|lcl|cTer
GVVDILKGAAKDIAGALASKVMNKL
>lcl|3448|lcl|HAPPENN&HAPPENN-RR90&HAPPENN-hard|lcl|subdataset_12|lcl|hemolytic
GIPCAESCVYIPCTVTALLGCSCSNRVCYN
>lcl|2531|lcl|HAPPENN&HAPPENN-RR90|lcl|subdataset_12|lcl|non-hemolytic|lcl|cTer
AARIILRRRFR
>lcl|703|lcl|HAPPENN|lcl|subdataset_8|lcl|hemolytic
ILGKIWEGIKSLF
>lcl|1330|lcl|HAPPENN&HAPPENN-RR90&HAPPENN-hard|lcl|subdataset_4|lcl|hemolytic|lcl|cTer
KNWGAILKHIIK
>lcl|3616|lcl|HAPPENN|lcl|subdataset_5|lcl|non-hemolytic
GVLDILKGAAKDLAGH
>lcl|4677|lcl|HAPPENN&HAPPENN-RR90|lcl|subdataset_4|lcl|non-hemolytic|lcl|cTer
KKWRKLLKWLAKK
>lcl|2622|lcl|HAPPENN|lcl|subdataset_4|lcl|hemolytic|lcl|nTer|lcl|cTer
GIGKFLHAAKKFAKAFVAEIMNS
>lcl|2348|lcl|HAPPENN&HAPPENN-RR90&HAPPENN-hard|lcl|subdataset_5|lcl|non-hemolytic
FVPAILCSILKTC
>lcl|1758|lcl|HAPPENN&HAPPENN-RR90&HAPPENN-hard|lcl|subdataset_4|lcl|hemolytic
NLLNKALGTVNGLLGRS
>lcl|4101|lcl|HAPPENN|lcl|subdataset_6|lcl|hemolytic|lcl|cTer
GWLDVAKKKGKAAFNVAKNFI
>lcl|1883|lcl|HAPPENN&HAPPENN-RR90|lcl|subdataset_12|lcl|non-hemolytic
NRFTARFRRTPWRLCLQFRQ
>lcl|4548|lcl|HAPPENN&HAPPENN-RR90|lcl|subdataset_4|lcl|non-hemolytic
SKVWRHWRRFWHRAHRLH
>lcl|1786|lcl|HAPPENN&HAPPENN-RR90|lcl|subdataset_11|lcl|non-hemolytic
RLSRIVVIRVSR
>lcl|3039|lcl|HAPPENN&HAPPENN-RR90&HAPPENN-hard|lcl|subdataset_10|lcl|hemolytic|lcl|cTer
GKWMSKLKHILK
>lcl|4495|lcl|HAPPENN&HAPPENN-RR90&HAPPENN-hard|lcl|subdataset_11|lcl|hemolytic
GMASLWAKVLPHVVKLIK
>lcl|4477|lcl|HAPPENN|lcl|subdataset_11|lcl|hemolytic|lcl|cTer
GILSSLWKKLKKIIAK
>lcl|2288|lcl|HAPPENN&HAPPENN-RR90|lcl|subdataset_9|lcl|non-hemolytic
TSVRQRWRWRQRVRTS
>lcl|3672|lcl|HAPPENN&HAPPENN-RR90|lcl|subdataset_10|lcl|non-hemolytic
RRRFVAQQNAIHSRVSRQVPTLSNSV
>lcl|2181|lcl|HAPPENN|lcl|subdataset_8|lcl|hemolytic|lcl|cTer
WLNALKKVWQGIHEAIKLIWNWVQ
>lcl|4671|lcl|HAPPENN&HAPPENN-RR90&HAPPENN-hard|lcl|subdataset_10|lcl|hemolytic|lcl|cTer
FFSMIPHIATGIASLVKNL
>lcl|3237|lcl|HAPPENN|lcl|subdataset_11|lcl|hemolytic|lcl|cTer
GLVTGLLNTAGGLLGDLFGSLSG
>lcl|491|lcl|HAPPENN|lcl|subdataset_7|lcl|non-hemolytic
AKKVFKRLEKLFSKIQN
```

```
>lcl|1185|lcl|HAPPENN&HAPPENN-RR90|lcl|subdataset_6|lcl|non-hemolytic
RRRRRRRRRRGIGKFLHSAKKFGKAFVGEIMNS
>lcl|1046|lcl|HAPPENN&HAPPENN-RR90|lcl|subdataset_8|lcl|non-hemolytic
RWKIFKKIEKVGQNIRDGIVKAGPAVAVVGQAATI
>lcl|4267|lcl|HAPPENN|lcl|subdataset_6|lcl|hemolytic
GIGAVLKVLTTGLCALISWIKRKRQQ
>lcl|3321|lcl|HAPPENN&HAPPENN-RR90&HAPPENN-hard|lcl|subdataset_10|lcl|hemolytic
GIRCPKSWKCKAFKQRVLKRLLAMLRQHAF
>lcl|1234|lcl|HAPPENN&HAPPENN-RR90&HAPPENN-hard|lcl|subdataset_8|lcl|hemolytic|lcl|cTer
IIGPVLGLVGSALGGLLKKI
>lcl|4810|lcl|HAPPENN|lcl|subdataset_12|lcl|hemolytic|lcl|cTer
KKYRYHLKPFSKK
>lcl|2454|lcl|HAPPENN&HAPPENN-RR90|lcl|subdataset_8|lcl|non-hemolytic|lcl|nTer|lcl|cTer
RKFLRRRGEVAHFSQKSLGLYCWWW
>lcl|1618|lcl|HAPPENN|lcl|subdataset_7|lcl|non-hemolytic|lcl|cTer
RRWWHWWRR
>lcl|186|lcl|HAPPENN&HAPPENN-RR90|lcl|subdataset_9|lcl|non-hemolytic|lcl|nTer|lcl|cTer
KWKLFKKIGKVLKKL
>lcl|1263|lcl|HAPPENN&HAPPENN-hard|lcl|subdataset_7|lcl|non-hemolytic
FLPVLAGVLSRA
>lcl|2158|lcl|HAPPENN&HAPPENN-RR90&HAPPENN-hard|lcl|subdataset_11|lcl|hemolytic|lcl|cTer
RIVQRILKWLKKWYKLGK
>lcl|2429|lcl|HAPPENN&HAPPENN-RR90&HAPPENN-hard|lcl|subdataset_7|lcl|hemolytic
GLLDLLKLLLKAAG
>lcl|4627|lcl|HAPPENN|lcl|subdataset_9|lcl|non-hemolytic|lcl|cTer
GRRPRPRPRPFFFF
>lcl|2777|lcl|HAPPENN|lcl|subdataset_6|lcl|hemolytic
GLPVCGETCVGGTCNTPGCSCSWPVCFRN
>lcl|1760|lcl|HAPPENN&HAPPENN-RR90&HAPPENN-hard|lcl|subdataset_8|lcl|hemolytic
GLPTCGETCTLGKCNTPKCTCNWPICYKD
>lcl|3864|lcl|HAPPENN&HAPPENN-RR90|lcl|subdataset_9|lcl|non-hemolytic|lcl|cTer
RKKRKKLIKRLI
>lcl|3092|lcl|HAPPENN&HAPPENN-RR90&HAPPENN-hard|lcl|subdataset_10|lcl|hemolytic|lcl|cTer
VNWRRILGRIIRVVR
>lcl|1118|lcl|HAPPENN|lcl|subdataset_5|lcl|non-hemolytic
RLMAAKAESRK
>lcl|1696|lcl|HAPPENN|lcl|subdataset_11|lcl|non-hemolytic|lcl|cTer
KKLFKKILKEL
>lcl|3951|lcl|HAPPENN&HAPPENN-RR90|lcl|subdataset_9|lcl|non-hemolytic|lcl|nTer|lcl|cTer
GWLQLAQHLLQLGQHLLQLAQQLRRR
>lcl|4241|lcl|HAPPENN&HAPPENN-RR90&HAPPENN-hard|lcl|subdataset_10|lcl|non-hemolytic
APVPFSCTRGCLTHLV
>lcl|1736|lcl|HAPPENN&HAPPENN-RR90&HAPPENN-hard|lcl|subdataset_4|lcl|hemolytic|lcl|cTer
IIGPVLGLIGKALGGLL
>lcl|2711|lcl|HAPPENN&HAPPENN-RR90&HAPPENN-hard|lcl|subdataset_10|lcl|hemolytic
VFIDILDKMENAIHKAAQAGIG
>lcl|334|lcl|HAPPENN&HAPPENN-RR90&HAPPENN-hard|lcl|subdataset_4|lcl|hemolytic|lcl|cTer
GLFGVLAKVAAKVVPAIAKHF
>lcl|2760|lcl|HAPPENN|lcl|subdataset_9|lcl|hemolytic
SMLSVLKNLGKVGLGFVKCKINKQC
>lcl|4475|lcl|HAPPENN|lcl|subdataset_8|lcl|hemolytic|lcl|cTer
GIWSSLLKKLKKIIAK
>lcl|3090|lcl|HAPPENN&HAPPENN-RR90&HAPPENN-hard|lcl|subdataset_5|lcl|hemolytic|lcl|cTer
VNWKKIILGKIIKVVK
>lcl|3283|lcl|HAPPENN&HAPPENN-RR90|lcl|subdataset_4|lcl|non-hemolytic|lcl|cTer
KFLHLAKKFPKWKLFKKI
>lcl|637|lcl|HAPPENN|lcl|subdataset_9|lcl|non-hemolytic|lcl|cTer
KWKLFKKIPLHLAKKF
>lcl|347|lcl|HAPPENN&HAPPENN-RR90&HAPPENN-hard|lcl|subdataset_12|lcl|hemolytic
GRSRWRDVCRNFMRRYQSRVIQGLV
>lcl|2442|lcl|HAPPENN|lcl|subdataset_11|lcl|non-hemolytic
HLNKRVQRELIRWLDWLK
>lcl|3181|lcl|HAPPENN|lcl|subdataset_9|lcl|hemolytic|lcl|cTer
FLSLIPHIVSGVASIAKHF
>lcl|2955|lcl|HAPPENN&HAPPENN-hard|lcl|subdataset_9|lcl|non-hemolytic|lcl|cTer
GLLARIKTLL
```

```
>lcl|644|lcl|HAPPENN|lcl|subdataset_6|lcl|non-hemolytic|lcl|cTer
KWKLFKKIPKFLH
>lcl|1396|lcl|HAPPENN|lcl|subdataset_5|lcl|hemolytic|lcl|cTer
INWKKIFEKVKNLV
>lcl|3404|lcl|HAPPENN|lcl|subdataset_11|lcl|hemolytic
KRLFKKLKFSLRKY
>lcl|1719|lcl|HAPPENN&HAPPENN-RR90|lcl|subdataset_9|lcl|non-hemolytic
RWCVYARRRGVRYRRCW
>lcl|1540|lcl|HAPPENN|lcl|subdataset_8|lcl|non-hemolytic|lcl|cTer
WMLKKFRGMF
>lcl|4541|lcl|HAPPENN|lcl|subdataset_12|lcl|hemolytic|lcl|cTer
FFHHIFRGIVHIGKTIHRLVTG
>lcl|125|lcl|HAPPENN&HAPPENN-hard|lcl|subdataset_8|lcl|non-hemolytic
CYCRRRFCVCVGR
>lcl|3413|lcl|HAPPENN&HAPPENN-hard|lcl|subdataset_6|lcl|non-hemolytic|lcl|cTer
HFLGTLVNLAKKKL
>lcl|4234|lcl|HAPPENN|lcl|subdataset_10|lcl|non-hemolytic|lcl|cTer
PRCRRRFCRP
>lcl|4657|lcl|HAPPENN&HAPPENN-RR90&HAPPENN-hard|lcl|subdataset_11|lcl|hemolytic|lcl|cTer
FLSMIPHIVSGVAALAKHL
>lcl|1641|lcl|HAPPENN&HAPPENN-RR90&HAPPENN-hard|lcl|subdataset_6|lcl|hemolytic|lcl|cTer
FLTGLIGGLMKALGK
>lcl|2692|lcl|HAPPENN&HAPPENN-RR90&HAPPENN-hard|lcl|subdataset_11|lcl|hemolytic|lcl|cTer
FDVMGIIKKIASAL
>lcl|3733|lcl|HAPPENN&HAPPENN-RR90&HAPPENN-hard|lcl|subdataset_5|lcl|non-hemolytic
FLGALWKVASK
>lcl|1437|lcl|HAPPENN&HAPPENN-RR90&HAPPENN-hard|lcl|subdataset_12|lcl|hemolytic
RVKRVWPLVIRTVIAGYNLYRAIKKK
>lcl|3122|lcl|HAPPENN&HAPPENN-RR90&HAPPENN-hard|lcl|subdataset_10|lcl|hemolytic|lcl|cTer
NILNTIINLAKKIL
>lcl|738|lcl|HAPPENN|lcl|subdataset_10|lcl|non-hemolytic|lcl|cTer
AKKVFKRLEKLFSKIQNDK
>lcl|3761|lcl|HAPPENN&HAPPENN-RR90&HAPPENN-hard|lcl|subdataset_7|lcl|hemolytic
FLPLLLSALPSFLCLVFKKC
>lcl|1095|lcl|HAPPENN|lcl|subdataset_5|lcl|hemolytic
FLPLLLASLPSFLCLVFKKC
>lcl|2840|lcl|HAPPENN|lcl|subdataset_5|lcl|hemolytic|lcl|cTer
INWKKIASIGKEV
>lcl|2632|lcl|HAPPENN&HAPPENN-RR90&HAPPENN-hard|lcl|subdataset_4|lcl|hemolytic
GILSSIKGVAKGVAKNVAAQLLDTLKCKITGC
>lcl|3582|lcl|HAPPENN|lcl|subdataset_8|lcl|hemolytic
FWGALAKGALKLIGSLFSSFSKKD
>lcl|2513|lcl|HAPPENN&HAPPENN-RR90|lcl|subdataset_10|lcl|non-hemolytic|lcl|cTer
KKLLKGLLKGW
>lcl|3660|lcl|HAPPENN|lcl|subdataset_11|lcl|non-hemolytic
SRSELIVHQR
>lcl|2297|lcl|HAPPENN&HAPPENN-RR90|lcl|subdataset_4|lcl|non-hemolytic|lcl|cTer
TKKTKLTEEEKNRL
>lcl|3306|lcl|HAPPENN&HAPPENN-hard|lcl|subdataset_10|lcl|non-hemolytic|lcl|cTer
GLKKIFKAGLGSLVKGIKAHVAS
>lcl|3123|lcl|HAPPENN&HAPPENN-RR90&HAPPENN-hard|lcl|subdataset_7|lcl|hemolytic
FLPLIASLAANFVPKIFCKITKKC
>lcl|1434|lcl|HAPPENN|lcl|subdataset_10|lcl|hemolytic|lcl|cTer
SNWKKIASIGKEVLKAL
>lcl|3583|lcl|HAPPENN|lcl|subdataset_12|lcl|hemolytic
FWGALAKGALKLIGPGSLFSSFSKKD
>lcl|1975|lcl|HAPPENN&HAPPENN-RR90|lcl|subdataset_5|lcl|non-hemolytic|lcl|cTer
GKEFKRIVGRIYRLCCR
>lcl|4454|lcl|HAPPENN&HAPPENN-hard|lcl|subdataset_11|lcl|non-hemolytic|lcl|cTer
KILGKLWKGVKSIF
>lcl|4709|lcl|HAPPENN&HAPPENN-hard|lcl|subdataset_5|lcl|non-hemolytic|lcl|nTer|lcl|cTer
KWKSFLKTFKSAEKTVLHTALKAISS
>lcl|2078|lcl|HAPPENN&HAPPENN-RR90|lcl|subdataset_10|lcl|non-hemolytic
KRCKNKMEGDDVAVSGRGARKAAKK
>lcl|424|lcl|HAPPENN|lcl|subdataset_10|lcl|non-hemolytic|lcl|cTer
HFLLTLVNLKKKIL
>lcl|1279|lcl|HAPPENN|lcl|subdataset_5|lcl|hemolytic|lcl|cTer
GFLSILKKVL
```

```
>lcl|2459|lcl|HAPPENN&HAPPENN-RR90&HAPPENN-hard|lcl|subdataset_6|lcl|hemolytic|lcl|cTer
INWLAIAKKVAGML
>lcl|1732|lcl|HAPPENN|lcl|subdataset_12|lcl|non-hemolytic|lcl|nTer|lcl|cTer
KWKSFLKTFKSAKKTALHTALKAISS
>lcl|2725|lcl|HAPPENN|lcl|subdataset_11|lcl|hemolytic
GFRDVLKGAAKAFVKTVAGHIAN
>lcl|1448|lcl|HAPPENN&HAPPENN-hard|lcl|subdataset_9|lcl|non-hemolytic
SSLLEKGLDGAKKAVGGLGKLGK
>lcl|3992|lcl|HAPPENN|lcl|subdataset_6|lcl|hemolytic|lcl|nTer|lcl|cTer
GFIFHIIKGLFHAGKMIHGLV
>lcl|3988|lcl|HAPPENN&HAPPENN-hard|lcl|subdataset_7|lcl|non-hemolytic|lcl|cTer
GVVAILKGAAKDIAGHLASKVMNKL
>lcl|2300|lcl|HAPPENN&HAPPENN-RR90|lcl|subdataset_4|lcl|non-hemolytic
RRLRPRRPRLPRPRPRPRPRPR
>lcl|4819|lcl|HAPPENN&HAPPENN-RR90|lcl|subdataset_11|lcl|non-hemolytic|lcl|cTer
GFWGKLWKPVKKAI
>lcl|3026|lcl|HAPPENN|lcl|subdataset_5|lcl|hemolytic|lcl|cTer
GKWSKILGHLIR
>lcl|3049|lcl|HAPPENN|lcl|subdataset_5|lcl|hemolytic|lcl|cTer
GKFMSLLKHILK
>lcl|3925|lcl|HAPPENN&HAPPENN-RR90|lcl|subdataset_9|lcl|non-hemolytic|lcl|cTer
RFRRLRWKTRWRLKKI
>lcl|2813|lcl|HAPPENN&HAPPENN-RR90|lcl|subdataset_8|lcl|non-hemolytic|lcl|cTer
LCAALCLAIGRR
>lcl|625|lcl|HAPPENN|lcl|subdataset_6|lcl|hemolytic|lcl|cTer
KWKLFKKILKFLHLAKKF
>lcl|1533|lcl|HAPPENN|lcl|subdataset_10|lcl|hemolytic|lcl|cTer
IWRIFRRIF
>lcl|4491|lcl|HAPPENN|lcl|subdataset_4|lcl|hemolytic
GMASLLAKVLPHVVKLIK
>lcl|2724|lcl|HAPPENN|lcl|subdataset_4|lcl|hemolytic
GFKDLLKGAAKALVKTVLF
>lcl|3380|lcl|HAPPENN&HAPPENN-RR90&HAPPENN-hard|lcl|subdataset_7|lcl|hemolytic|lcl|cTer
ILPWKWPWWPWAR
>lcl|389|lcl|HAPPENN|lcl|subdataset_10|lcl|hemolytic|lcl|cTer
KWKKLLKKLLKLLKKLLKKLK
>lcl|1703|lcl|HAPPENN&HAPPENN-RR90|lcl|subdataset_11|lcl|non-hemolytic
KRFKKFFKKLKKFVKKGFKKFAKV
>lcl|4263|lcl|HAPPENN&HAPPENN-RR90&HAPPENN-hard|lcl|subdataset_6|lcl|non-hemolytic|lcl|cTer
FFSLIPSLVGGLISAFK
>lcl|1857|lcl|HAPPENN|lcl|subdataset_8|lcl|hemolytic|lcl|cTer
FLSLIPKIAGGIAALAKHL
>lcl|1388|lcl|HAPPENN|lcl|subdataset_7|lcl|hemolytic|lcl|cTer
GLPALISWIKRKRQQG
>lcl|4317|lcl|HAPPENN&HAPPENN-RR90|lcl|subdataset_11|lcl|non-hemolytic|lcl|cTer
GKPRGNARLGLRPKRSRWL
>lcl|935|lcl|HAPPENN&HAPPENN-RR90&HAPPENN-hard|lcl|subdataset_9|lcl|hemolytic|lcl|cTer
FVQWFSKFLLRIL
>lcl|1761|lcl|HAPPENN|lcl|subdataset_7|lcl|hemolytic
GLPTCGETCTLGKCNTPKCTCNWPICYKN
>lcl|880|lcl|HAPPENN&HAPPENN-RR90&HAPPENN-hard|lcl|subdataset_7|lcl|hemolytic
MGIIAGIIKVIKSLIEQFTGK
>lcl|2258|lcl|HAPPENN&HAPPENN-RR90&HAPPENN-hard|lcl|subdataset_11|lcl|hemolytic
RKHGFLNLQGIFGDYYHFWHRGV
>lcl|3149|lcl|HAPPENN&HAPPENN-RR90|lcl|subdataset_4|lcl|non-hemolytic
APDRPRKFCGILG
>lcl|2666|lcl|HAPPENN|lcl|subdataset_7|lcl|non-hemolytic|lcl|cTer
AWLLAIRKR
>lcl|4580|lcl|HAPPENN&HAPPENN-hard|lcl|subdataset_4|lcl|non-hemolytic
VWRKWRRFW
>lcl|2874|lcl|HAPPENN|lcl|subdataset_6|lcl|non-hemolytic
KKKLLFKKLQ
>lcl|3248|lcl|HAPPENN&HAPPENN-RR90|lcl|subdataset_11|lcl|non-hemolytic
GRGREFMSNLKEKLSGVKEKMKNS
>lcl|1762|lcl|HAPPENN&HAPPENN-RR90&HAPPENN-hard|lcl|subdataset_5|lcl|hemolytic
GKYTCGETCFKGKCYTPGCTCSYPICKKD
>lcl|2479|lcl|HAPPENN&HAPPENN-RR90|lcl|subdataset_9|lcl|non-hemolytic
AKKKLSRWWLRWWVK
>lcl|3552|lcl|HAPPENN&HAPPENN-RR90&HAPPENN-hard|lcl|subdataset_6|lcl|hemolytic
GVWSTILGGLKKFAKGGLDAIVNPK
```

```
>lcl|4381|lcl|HAPPENN&HAPPENN-RR90&HAPPENN-hard|lcl|subdataset_5|lcl|non-hemolytic|lcl|cTer
ALLKLAPRLLAGIF
>lcl|4309|lcl|HAPPENN|lcl|subdataset_7|lcl|hemolytic
GIGKFLHSAKKAGKAFVGEIMNS
>lcl|3587|lcl|HAPPENN&HAPPENN-RR90|lcl|subdataset_6|lcl|non-hemolytic|lcl|cTer
RRRRRRRKDVY
>lcl|4478|lcl|HAPPENN&HAPPENN-RR90&HAPPENN-hard|lcl|subdataset_4|lcl|non-hemolytic|lcl|cTer
GKLSSLWKKLKKIIAK
>lcl|2916|lcl|HAPPENN|lcl|subdataset_9|lcl|non-hemolytic
LKLKLFKKKQ
>lcl|4004|lcl|HAPPENN&HAPPENN-hard|lcl|subdataset_12|lcl|non-hemolytic
KIGAKIKIGAKIKIGAKI
>lcl|2593|lcl|HAPPENN|lcl|subdataset_6|lcl|non-hemolytic|lcl|cTer
INLKAIAALAKKLL
>lcl|1126|lcl|HAPPENN&HAPPENN-RR90&HAPPENN-hard|lcl|subdataset_7|lcl|non-hemolytic
KKLLPIVANLLKSLL
>lcl|269|lcl|HAPPENN|lcl|subdataset_7|lcl|non-hemolytic|lcl|cTer
ILPKKWPKLPWRR
>lcl|1319|lcl|HAPPENN&HAPPENN-RR90&HAPPENN-hard|lcl|subdataset_7|lcl|non-hemolytic
FLPLLAGLAANFLPTIICKISYKC
>lcl|330|lcl|HAPPENN&HAPPENN-RR90&HAPPENN-hard|lcl|subdataset_8|lcl|hemolytic
FLPLLAGLAANFLPKIFCKITRKC
>lcl|301|lcl|HAPPENN|lcl|subdataset_5|lcl|non-hemolytic
MESVSVAVQLMLGFGTFVLMLLGLVVELIKNSNKK
>lcl|2008|lcl|HAPPENN&HAPPENN-RR90|lcl|subdataset_12|lcl|non-hemolytic|lcl|cTer
IVNHLVKLFDKGLNSIVNLR
>lcl|442|lcl|HAPPENN|lcl|subdataset_7|lcl|hemolytic|lcl|cTer
KKLFKKILKWL
>lcl|2810|lcl|HAPPENN|lcl|subdataset_11|lcl|non-hemolytic|lcl|cTer
LCAAHCLAIRRR
>lcl|4543|lcl|HAPPENN|lcl|subdataset_5|lcl|hemolytic|lcl|cTer
WKLFKKILKVL
>lcl|1645|lcl|HAPPENN&HAPPENN-RR90|lcl|subdataset_9|lcl|non-hemolytic
DNVYSRPPQRFGQNVIS
>lcl|4329|lcl|HAPPENN&HAPPENN-RR90|lcl|subdataset_11|lcl|non-hemolytic|lcl|cTer
GIHAILKYGKPS
>lcl|2135|lcl|HAPPENN&HAPPENN-RR90&HAPPENN-hard|lcl|subdataset_7|lcl|hemolytic|lcl|cTer
ALWKDLLKNVGKAAGKAVLNKVTDMVNQ
>lcl|2698|lcl|HAPPENN&HAPPENN-RR90&HAPPENN-hard|lcl|subdataset_4|lcl|non-hemolytic|lcl|cTer
RSNKGFNFMVDMIQALSK
>lcl|3208|lcl|HAPPENN&HAPPENN-hard|lcl|subdataset_7|lcl|non-hemolytic|lcl|cTer
GIGGALLSAGKSALKGLAKGLAEHF
>lcl|907|lcl|HAPPENN|lcl|subdataset_4|lcl|non-hemolytic|lcl|cTer
AKKKGKCKGPLKLVAKC
>lcl|1482|lcl|HAPPENN|lcl|subdataset_9|lcl|hemolytic
CGESCVWIPCISAVVGCSCKSKVCYKNGTLP
>lcl|3186|lcl|HAPPENN&HAPPENN-RR90&HAPPENN-hard|lcl|subdataset_6|lcl|non-hemolytic
ALYKKLLKKLLKSAKKLG
>lcl|1950|lcl|HAPPENN|lcl|subdataset_10|lcl|hemolytic
FLPLLLAGLPKFLCLVFKKC
>lcl|3054|lcl|HAPPENN&HAPPENN-RR90&HAPPENN-hard|lcl|subdataset_7|lcl|hemolytic|lcl|cTer
GKWMTLLKHILK
>lcl|4574|lcl|HAPPENN|lcl|subdataset_12|lcl|non-hemolytic
VWRHWRRFW
>lcl|3819|lcl|HAPPENN|lcl|subdataset_10|lcl|non-hemolytic|lcl|cTer
GRFRRLRKKTRKRLKKIGKV
>lcl|1724|lcl|HAPPENN&HAPPENN-RR90|lcl|subdataset_12|lcl|non-hemolytic
RLLFRKIRRLKR
>lcl|713|lcl|HAPPENN&HAPPENN-hard|lcl|subdataset_6|lcl|non-hemolytic|lcl|cTer
GLPALSSWIKRKRQQ
>lcl|4529|lcl|HAPPENN&HAPPENN-RR90&HAPPENN-hard|lcl|subdataset_4|lcl|hemolytic|lcl|cTer
LRRLRKWLRRLLKLL
>lcl|2411|lcl|HAPPENN&HAPPENN-RR90|lcl|subdataset_12|lcl|non-hemolytic
FFHHIFDPQIKSGLLVSMYD
>lcl|3229|lcl|HAPPENN|lcl|subdataset_4|lcl|hemolytic|lcl|cTer
ILGPVISTIGNALGGLLKNL
>lcl|3887|lcl|HAPPENN|lcl|subdataset_11|lcl|non-hemolytic|lcl|cTer
```

```
KRRKAIKILKLIAKAIRKKR
>lcl|4031|lcl|HAPPENN|lcl|subdataset_9|lcl|non-hemolytic
QPEWFKARRWQWRMKKLGA
>lcl|2707|lcl|HAPPENN|lcl|subdataset_4|lcl|non-hemolytic|lcl|cTer
KKKVVFKVKFK
>lcl|450|lcl|HAPPENN&HAPPENN-RR90&HAPPENN-hard|lcl|subdataset_10|lcl|hemolytic|lcl|cTer
SLLSLINKLIT
>lcl|714|lcl|HAPPENN&HAPPENN-hard|lcl|subdataset_12|lcl|non-hemolytic|lcl|cTer
GLPALILWIKRKRQQ
>lcl|3270|lcl|HAPPENN&HAPPENN-RR90&HAPPENN-hard|lcl|subdataset_10|lcl|hemolytic
GIPCAESCVWIPCTITALMGCSCKNNVCYNN
>lcl|3664|lcl|HAPPENN|lcl|subdataset_5|lcl|non-hemolytic|lcl|cTer
GVVVRVGRVVVRGVRR
>lcl|1135|lcl|HAPPENN|lcl|subdataset_7|lcl|hemolytic|lcl|cTer
FLSTIWNGIKSLL
>lcl|2441|lcl|HAPPENN&HAPPENN-RR90&HAPPENN-hard|lcl|subdataset_12|lcl|non-hemolytic|lcl|cTer
LKLMGIVKKVLGAL
>lcl|604|lcl|HAPPENN|lcl|subdataset_4|lcl|hemolytic
GWGSFFKKAAHVAKHVAKAALTHYL
>lcl|3171|lcl|HAPPENN&HAPPENN-RR90&HAPPENN-hard|lcl|subdataset_8|lcl|non-hemolytic
FIGPVLKMATSILPTAICKGFKKC
>lcl|954|lcl|HAPPENN&HAPPENN-hard|lcl|subdataset_6|lcl|non-hemolytic|lcl|cTer
FLPLIGRVLSGAL
>lcl|884|lcl|HAPPENN|lcl|subdataset_5|lcl|hemolytic|lcl|cTer
GWLDVAKKIGKAAFNVAKNFL
>lcl|4796|lcl|HAPPENN|lcl|subdataset_10|lcl|non-hemolytic|lcl|cTer
WRNGRWWRNGRW
>lcl|281|lcl|HAPPENN&HAPPENN-RR90|lcl|subdataset_11|lcl|non-hemolytic|lcl|cTer
ILPAKAPAAPARR
>lcl|3223|lcl|HAPPENN&HAPPENN-hard|lcl|subdataset_6|lcl|non-hemolytic|lcl|cTer
ILGPVLGLVSNTLDDVLGIL
>lcl|515|lcl|HAPPENN&HAPPENN-RR90&HAPPENN-hard|lcl|subdataset_9|lcl|hemolytic|lcl|cTer
GLLGKLLKIAAKVGSNLL
>lcl|1366|lcl|HAPPENN&HAPPENN-hard|lcl|subdataset_10|lcl|non-hemolytic|lcl|cTer
GFFALIPKIISSPLFKTL
>lcl|87|lcl|HAPPENN&HAPPENN-RR90|lcl|subdataset_12|lcl|non-hemolytic|lcl|nTer|lcl|cTer
KWKEFLKEFKEAKKEVLHEALKAISE
>lcl|2445|lcl|HAPPENN&HAPPENN-hard|lcl|subdataset_5|lcl|non-hemolytic
HLNKRVQRLLIGWLDWLK
>lcl|2628|lcl|HAPPENN|lcl|subdataset_9|lcl|hemolytic|lcl|cTer
GIGAVLLVLTTGLPALISWIKRKRQQ
>lcl|868|lcl|HAPPENN&HAPPENN-RR90&HAPPENN-hard|lcl|subdataset_6|lcl|hemolytic
GLWDTIKQAGKKFFLNVLDKIRCKVAGGCRT
>lcl|2714|lcl|HAPPENN|lcl|subdataset_12|lcl|hemolytic
SWLSKTAKKLENSAKKRISEGIAIAQGPR
>lcl|4791|lcl|HAPPENN&HAPPENN-RR90|lcl|subdataset_6|lcl|non-hemolytic|lcl|cTer
KKKAAFAAWAAFAAKKK
>lcl|3594|lcl|HAPPENN&HAPPENN-RR90&HAPPENN-hard|lcl|subdataset_8|lcl|hemolytic
LVQRGRFGRFLRKIRRFRPKVTITIQGSARF
>lcl|3473|lcl|HAPPENN|lcl|subdataset_6|lcl|non-hemolytic|lcl|cTer
LRPAILVRIK
>lcl|500|lcl|HAPPENN&HAPPENN-RR90&HAPPENN-hard|lcl|subdataset_6|lcl|hemolytic|lcl|cTer
GVIKSVLKGVAKTVALGML
>lcl|777|lcl|HAPPENN&HAPPENN-RR90&HAPPENN-hard|lcl|subdataset_8|lcl|hemolytic
GLMDTVKNAAKNLAGQLLDTIKCKMTGC
>lcl|1130|lcl|HAPPENN|lcl|subdataset_11|lcl|hemolytic|lcl|cTer
GFGALFKFLAKKVAKTVAKQAAKQGAKYVVNKQME
>lcl|2641|lcl|HAPPENN&HAPPENN-RR90&HAPPENN-hard|lcl|subdataset_5|lcl|non-hemolytic|lcl|cTer
FFPIVGKLLSGLSGLL
>lcl|4518|lcl|HAPPENN|lcl|subdataset_11|lcl|hemolytic
FWPVVIRTVVAGYNLYRAIKKK
>lcl|1485|lcl|HAPPENN|lcl|subdataset_9|lcl|hemolytic
CGESCVWIPCISAALGCSCKNKVCYRNGIP
>lcl|1821|lcl|HAPPENN&HAPPENN-RR90&HAPPENN-hard|lcl|subdataset_4|lcl|non-hemolytic|lcl|cTer
RRPWRWPWWPWRR
>lcl|1816|lcl|HAPPENN&HAPPENN-RR90&HAPPENN-hard|lcl|subdataset_11|lcl|non-hemolytic|lcl|cTer
LIGSLVRGILPLF
>lcl|1517|lcl|HAPPENN|lcl|subdataset_5|lcl|hemolytic|lcl|cTer
```

```
GFMKYIGPLIPHAVKAIKKLI
>lcl|134|lcl|HAPPENN|lcl|subdataset_8|lcl|non-hemolytic
CYCRRRFCVC
>lcl|1145|lcl|HAPPENN&HAPPENN-RR90&HAPPENN-hard|lcl|subdataset_7|lcl|hemolytic|lcl|cTer
GLAANFLPKIFCKITKRC
>lcl|3885|lcl|HAPPENN|lcl|subdataset_4|lcl|hemolytic|lcl|cTer
KRRKLIKILKLIAKLIRKKR
>lcl|4778|lcl|HAPPENN|lcl|subdataset_11|lcl|non-hemolytic|lcl|cTer
WGRAFSAGVHRLANGGNG
>lcl|4220|lcl|HAPPENN&HAPPENN-RR90&HAPPENN-hard|lcl|subdataset_10|lcl|non-hemolytic
GFMDTAKNVFKNVAVTLLDKLKCKIAGGC
>lcl|2746|lcl|HAPPENN|lcl|subdataset_9|lcl|hemolytic|lcl|cTer
GGLRSLGRKILRAWKKYGPIIVPIIRI
>lcl|1591|lcl|HAPPENN|lcl|subdataset_4|lcl|non-hemolytic|lcl|cTer
KIAGKIAKIAKKIAKIA
>lcl|555|lcl|HAPPENN|lcl|subdataset_8|lcl|hemolytic|lcl|cTer
KLLLKLLLKLLK
>lcl|2201|lcl|HAPPENN|lcl|subdataset_12|lcl|non-hemolytic|lcl|cTer
GLWSKIKEVGKEAAKAAAKAAGKAALGAVSEAV
>lcl|3781|lcl|HAPPENN|lcl|subdataset_7|lcl|non-hemolytic|lcl|cTer
GIRDILKYGKPS
>lcl|475|lcl|HAPPENN|lcl|subdataset_11|lcl|non-hemolytic|lcl|cTer
KWKLFKKIGIGKFLHSAKKF
>lcl|1246|lcl|HAPPENN&HAPPENN-RR90&HAPPENN-hard|lcl|subdataset_5|lcl|hemolytic|lcl|cTer
GIGKFLHTLKTFGKKWVGEIMNS
>lcl|152|lcl|HAPPENN|lcl|subdataset_11|lcl|hemolytic
RGGRLCYCRRRFCVCI
>lcl|185|lcl|HAPPENN|lcl|subdataset_6|lcl|non-hemolytic|lcl|nTer|lcl|cTer
KWKKFKKIGAVLKKL
>lcl|572|lcl|HAPPENN|lcl|subdataset_12|lcl|non-hemolytic|lcl|cTer
INPKSVQSLL
>lcl|477|lcl|HAPPENN&HAPPENN-RR90&HAPPENN-hard|lcl|subdataset_10|lcl|non-hemolytic
SADVAGAVIDGASLSFKILKTVLEALGNVKRK
>lcl|3995|lcl|HAPPENN|lcl|subdataset_7|lcl|hemolytic|lcl|cTer
LLKKVLKAAA
>lcl|2659|lcl|HAPPENN&HAPPENN-RR90&HAPPENN-hard|lcl|subdataset_10|lcl|hemolytic
GILDKLKEFGISAARGVAQSLLNTASCKLAKTC
>lcl|3705|lcl|HAPPENN|lcl|subdataset_6|lcl|non-hemolytic|lcl|nTer|lcl|cTer
KWKKLPKKLLKLL
>lcl|2871|lcl|HAPPENN|lcl|subdataset_10|lcl|non-hemolytic
KKLKKFLKLQ
>lcl|2602|lcl|HAPPENN&HAPPENN-hard|lcl|subdataset_8|lcl|non-hemolytic
SIRDKIKTIAIDLAKSAGMGILKTLICKLDKSC
>lcl|906|lcl|HAPPENN|lcl|subdataset_4|lcl|non-hemolytic|lcl|cTer
LCAIAKKKGKCKGPLKLVCKC
>lcl|1343|lcl|HAPPENN&HAPPENN-RR90&HAPPENN-hard|lcl|subdataset_9|lcl|hemolytic|lcl|cTer
LNWGAALKHAAK
>lcl|2299|lcl|HAPPENN&HAPPENN-RR90&HAPPENN-hard|lcl|subdataset_7|lcl|hemolytic
GRFKRFRKKLKRLWHKVGPFVGPILHY
>lcl|1931|lcl|HAPPENN&HAPPENN-hard|lcl|subdataset_10|lcl|non-hemolytic|lcl|cTer
RIGSILGALAKGLPTLKSWIKNR
>lcl|957|lcl|HAPPENN|lcl|subdataset_4|lcl|non-hemolytic
FKCRRWQWRMKKLGA
>lcl|4631|lcl|HAPPENN|lcl|subdataset_7|lcl|non-hemolytic|lcl|cTer
RRPRPRPRPFFF
>lcl|2615|lcl|HAPPENN|lcl|subdataset_9|lcl|non-hemolytic|lcl|cTer
KRWWKWIRW
>lcl|3368|lcl|HAPPENN|lcl|subdataset_4|lcl|hemolytic|lcl|cTer
WKWPWWPWRR
>lcl|1560|lcl|HAPPENN&HAPPENN-RR90|lcl|subdataset_10|lcl|non-hemolytic
KSRQWQWKIRRTWPIFSIRR
>lcl|2394|lcl|HAPPENN|lcl|subdataset_6|lcl|non-hemolytic|lcl|cTer
AKKVFKRLPKLPSKIWNWK
>lcl|4019|lcl|HAPPENN&HAPPENN-RR90|lcl|subdataset_11|lcl|non-hemolytic|lcl|nTer|lcl|cTer
PKRKSATKGDEPA
>lcl|4058|lcl|HAPPENN&HAPPENN-RR90|lcl|subdataset_9|lcl|non-hemolytic
ACYCRIPACIAGERRYGTCIYQGRLWAFCC
>lcl|1509|lcl|HAPPENN&HAPPENN-RR90|lcl|subdataset_6|lcl|non-hemolytic|lcl|cTer
SWKSMAKKLKEYMEKLKQRA
```

```
>lcl|4690|lcl|HAPPENN|lcl|subdataset_8|lcl|non-hemolytic|lcl|cTer
WRWKWRWK
>lcl|506|lcl|HAPPENN|lcl|subdataset_4|lcl|hemolytic|lcl|cTer
GFKDLLKGAAKALVKTVLF
>lcl|2197|lcl|HAPPENN&HAPPENN-RR90&HAPPENN-hard|lcl|subdataset_11|lcl|non-hemolytic
GVWGIAKIAGKVLGNILPHVFSSNQS
>lcl|57|lcl|HAPPENN|lcl|subdataset_7|lcl|non-hemolytic|lcl|nTer|lcl|cTer
KWKSFAKTFKSAKKTVLHTALKAISS
>lcl|1616|lcl|HAPPENN|lcl|subdataset_11|lcl|non-hemolytic|lcl|cTer
RRWWRWWRR
>lcl|3642|lcl|HAPPENN|lcl|subdataset_11|lcl|hemolytic
ALWMTLLKKVLKAAAKAALN
>lcl|2966|lcl|HAPPENN|lcl|subdataset_6|lcl|non-hemolytic|lcl|cTer
LKRIKTLL
>lcl|2770|lcl|HAPPENN&HAPPENN-RR90|lcl|subdataset_8|lcl|non-hemolytic
QWGRRCCGWGPGRRYCRRWC
>lcl|924|lcl|HAPPENN&HAPPENN-hard|lcl|subdataset_12|lcl|non-hemolytic|lcl|cTer
GWGSFFKKAAHVGKHVGKAALT
>lcl|3089|lcl|HAPPENN|lcl|subdataset_8|lcl|hemolytic|lcl|cTer
KNWKKILGKIIKVVK
>lcl|1918|lcl|HAPPENN&HAPPENN-RR90|lcl|subdataset_12|lcl|non-hemolytic
PNDPDSPCVYRMPNARGCSI
>lcl|1387|lcl|HAPPENN&HAPPENN-RR90&HAPPENN-hard|lcl|subdataset_9|lcl|hemolytic
PKLLKFLKWIG
>lcl|3565|lcl|HAPPENN|lcl|subdataset_8|lcl|hemolytic|lcl|cTer
FLPLLAGLAANFLPKIFCKITRKC
>lcl|3057|lcl|HAPPENN|lcl|subdataset_5|lcl|hemolytic|lcl|cTer
SKWMSLLKHILK
>lcl|3303|lcl|HAPPENN&HAPPENN-hard|lcl|subdataset_9|lcl|non-hemolytic|lcl|cTer
GLKKIFKAGLGSLVKGIAAHVAS
>lcl|4691|lcl|HAPPENN&HAPPENN-RR90|lcl|subdataset_10|lcl|non-hemolytic|lcl|cTer
VRVKVRVKVRVK
>lcl|4476|lcl|HAPPENN&HAPPENN-hard|lcl|subdataset_6|lcl|non-hemolytic|lcl|cTer
GILSSWLKKLKKIIAK
>lcl|673|lcl|HAPPENN|lcl|subdataset_8|lcl|non-hemolytic|lcl|cTer
RRRAAFAAWAAFAARRR
>lcl|1116|lcl|HAPPENN&HAPPENN-RR90&HAPPENN-hard|lcl|subdataset_11|lcl|non-hemolytic|lcl|cTer
DLLGTLGNLPLPFI
>lcl|2055|lcl|HAPPENN|lcl|subdataset_11|lcl|non-hemolytic
YKARRWAWRWK
>lcl|1498|lcl|HAPPENN&HAPPENN-hard|lcl|subdataset_11|lcl|non-hemolytic
CVHWQTNPARTSCIGP
>lcl|2209|lcl|HAPPENN|lcl|subdataset_4|lcl|non-hemolytic|lcl|cTer
TQQAFQKFLAAVTSALGKQYH
>lcl|3221|lcl|HAPPENN&HAPPENN-RR90&HAPPENN-hard|lcl|subdataset_8|lcl|non-hemolytic|lcl|cTer
ILGPVLGLVGDTLGDLL
>lcl|541|lcl|HAPPENN|lcl|subdataset_7|lcl|hemolytic
CGETCVGGTCNTPGCTCSWPVCTRNGLPV
>lcl|719|lcl|HAPPENN&HAPPENN-RR90&HAPPENN-hard|lcl|subdataset_12|lcl|non-hemolytic|lcl|cTer
FLSLIPHAINAVSTLVHHF
>lcl|3061|lcl|HAPPENN|lcl|subdataset_7|lcl|hemolytic|lcl|cTer
GLNALKKVFQGIHEAIKLINNHVQ
>lcl|4772|lcl|HAPPENN&HAPPENN-RR90|lcl|subdataset_11|lcl|non-hemolytic
KKCKFFCKVKKKIKSIGFQIPIVSIPFK
>lcl|4368|lcl|HAPPENN|lcl|subdataset_11|lcl|hemolytic
AIPWSIWWHLLFKG
>lcl|369|lcl|HAPPENN&HAPPENN-RR90&HAPPENN-hard|lcl|subdataset_10|lcl|hemolytic
FKIKPGKVLDKFGKIVGKVLKQLKKVSAVAKV
>lcl|538|lcl|HAPPENN&HAPPENN-hard|lcl|subdataset_4|lcl|non-hemolytic|lcl|cTer
LKWKSIVSWAKKVL
>lcl|2999|lcl|HAPPENN|lcl|subdataset_11|lcl|hemolytic
ALWKTMLKKLGTMALHAGKAAFGAAADTISQ
>lcl|1494|lcl|HAPPENN&HAPPENN-RR90&HAPPENN-hard|lcl|subdataset_9|lcl|hemolytic
GLWSKIKEAAKAAGKAALNAVTGLVNQGDQPS
>lcl|2373|lcl|HAPPENN|lcl|subdataset_9|lcl|non-hemolytic|lcl|cTer
IIRRIRRII
>lcl|136|lcl|HAPPENN&HAPPENN-RR90|lcl|subdataset_11|lcl|non-hemolytic
LCYCRRRRCVCV
>lcl|1265|lcl|HAPPENN&HAPPENN-RR90|lcl|subdataset_7|lcl|non-hemolytic
KIAEKFSGTRRG
```

```
>lcl|2647|lcl|HAPPENN|lcl|subdataset_11|lcl|hemolytic|lcl|cTer
FLPFVGNLLKGLL
>lcl|397|lcl|HAPPENN&HAPPENN-RR90&HAPPENN-hard|lcl|subdataset_12|lcl|hemolytic|lcl|cTer
VRRFAWWWAFLRR
>lcl|560|lcl|HAPPENN&HAPPENN-RR90|lcl|subdataset_11|lcl|non-hemolytic|lcl|cTer
AARRAARRAARR
>lcl|4198|lcl|HAPPENN&HAPPENN-RR90&HAPPENN-hard|lcl|subdataset_9|lcl|non-hemolytic|lcl|cTer
AGYLLGKINLKPLAALAKKIL
>lcl|2952|lcl|HAPPENN|lcl|subdataset_12|lcl|non-hemolytic|lcl|cTer
ALLKRIKTLL
>lcl|3094|lcl|HAPPENN|lcl|subdataset_11|lcl|hemolytic|lcl|cTer
VNWKKLLGKLLKVVK
>lcl|2776|lcl|HAPPENN&HAPPENN-RR90&HAPPENN-hard|lcl|subdataset_11|lcl|hemolytic
GLPVCGETCFTGSCYTPGCSCNWPVCNRN
>lcl|1026|lcl|HAPPENN|lcl|subdataset_6|lcl|hemolytic
GLPVCGETCVGGTCNTPGCNCSWPVCTRK
>lcl|2543|lcl|HAPPENN|lcl|subdataset_9|lcl|non-hemolytic|lcl|cTer
EPFKISIHL
>lcl|778|lcl|HAPPENN&HAPPENN-RR90&HAPPENN-hard|lcl|subdataset_11|lcl|hemolytic
GILDTIKNAAKTVAVGLLEKIKCKMTGC
>lcl|897|lcl|HAPPENN&HAPPENN-RR90|lcl|subdataset_4|lcl|non-hemolytic|lcl|cTer
KWKSFIKKLTSKFLHSKKKF
>lcl|1443|lcl|HAPPENN&HAPPENN-RR90&HAPPENN-hard|lcl|subdataset_6|lcl|hemolytic
RVKRVWKLVIRLVKALYKLYRAIKKK
>lcl|942|lcl|HAPPENN|lcl|subdataset_8|lcl|hemolytic|lcl|cTer
FVRWFSRFLGRIL
>lcl|3272|lcl|HAPPENN&HAPPENN-RR90&HAPPENN-hard|lcl|subdataset_9|lcl|hemolytic
IPCGESCVWIPCITAIAGCSCKNKVCYT
>lcl|3177|lcl|HAPPENN&HAPPENN-RR90&HAPPENN-hard|lcl|subdataset_5|lcl|hemolytic|lcl|cTer
FPFSLIPHAIGGLISAIK
>lcl|1172|lcl|HAPPENN&HAPPENN-RR90&HAPPENN-hard|lcl|subdataset_11|lcl|non-hemolytic
CSCKSKVCYKNSIPCGESCVFIPCTVTALLG
>lcl|2034|lcl|HAPPENN&HAPPENN-RR90&HAPPENN-hard|lcl|subdataset_8|lcl|non-hemolytic|lcl|cTer
FKRFKGSVKHGKGHLVHHIGVAL
>lcl|3052|lcl|HAPPENN&HAPPENN-RR90&HAPPENN-hard|lcl|subdataset_6|lcl|hemolytic|lcl|cTer
GKWMSALKHILK
>lcl|2483|lcl|HAPPENN|lcl|subdataset_7|lcl|non-hemolytic|lcl|cTer
AAKAAAKAAAKAA
>lcl|4380|lcl|HAPPENN|lcl|subdataset_7|lcl|non-hemolytic|lcl|nTer
RGRKGGRRKK
>lcl|2809|lcl|HAPPENN&HAPPENN-hard|lcl|subdataset_10|lcl|non-hemolytic|lcl|cTer
LCAAHCLAIGRR
>lcl|3543|lcl|HAPPENN&HAPPENN-hard|lcl|subdataset_7|lcl|non-hemolytic
GAFGDLLKGVAKEAGLKLLNMAQCKLSGNC
>lcl|3748|lcl|HAPPENN|lcl|subdataset_5|lcl|hemolytic
ISLNPPRSTIAMRAINNYRWRSKNQNTFLR
>lcl|33|lcl|HAPPENN&HAPPENN-RR90&HAPPENN-hard|lcl|subdataset_12|lcl|hemolytic|lcl|cTer
RRWCFRVCYKGFCRYKCR
>lcl|1621|lcl|HAPPENN&HAPPENN-RR90|lcl|subdataset_6|lcl|non-hemolytic|lcl|cTer
RERSKGSKYLYVG
>lcl|2696|lcl|HAPPENN&HAPPENN-RR90&HAPPENN-hard|lcl|subdataset_5|lcl|non-hemolytic|lcl|cTer
ILGKIWKPIKKLF
>lcl|89|lcl|HAPPENN&HAPPENN-RR90&HAPPENN-hard|lcl|subdataset_4|lcl|non-hemolytic|lcl|nTer|lcl|cTer
SWKSFLKTFSSAKSTVLHTALKAISS
>lcl|1209|lcl|HAPPENN&HAPPENN-RR90&HAPPENN-hard|lcl|subdataset_7|lcl|hemolytic
FFPLALLCKVFKKC
>lcl|1211|lcl|HAPPENN&HAPPENN-RR90&HAPPENN-hard|lcl|subdataset_4|lcl|hemolytic
IIPLPLGYFAKKT
>lcl|1470|lcl|HAPPENN|lcl|subdataset_6|lcl|hemolytic
CAESCVWIPCTVTALLGCSCSNNVCYNGIP
>lcl|436|lcl|HAPPENN&HAPPENN-hard|lcl|subdataset_5|lcl|non-hemolytic
FKRLEKLFKKIWNWK
>lcl|1979|lcl|HAPPENN&HAPPENN-RR90&HAPPENN-hard|lcl|subdataset_4|lcl|hemolytic
FFGHLFKLATKIIPSFFRRKNQ
>lcl|4576|lcl|HAPPENN|lcl|subdataset_4|lcl|non-hemolytic
RHWRRFWHR
```

```
>lcl|4793|lcl|HAPPENN|lcl|subdataset_10|lcl|non-hemolytic|lcl|cTer
WRPGRWWRPGRW
>lcl|1237|lcl|HAPPENN&HAPPENN-RR90&HAPPENN-hard|lcl|subdataset_7|lcl|hemolytic|lcl|cTer
AVAVVGQATQIAKKWKLFKKIEKVGQ
>lcl|4816|lcl|HAPPENN&HAPPENN-RR90&HAPPENN-hard|lcl|subdataset_11|lcl|non-hemolytic|lcl|cTer
FFSALLSGIKSLF
>lcl|3817|lcl|HAPPENN&HAPPENN-RR90&HAPPENN-hard|lcl|subdataset_4|lcl|non-hemolytic|lcl|cTer
LLIKFLKRFIKH
>lcl|2617|lcl|HAPPENN&HAPPENN-RR90&HAPPENN-hard|lcl|subdataset_8|lcl|hemolytic
GVPICGETCTLGTCYTAGCSCSWPVCTRN
>lcl|1225|lcl|HAPPENN&HAPPENN-RR90&HAPPENN-hard|lcl|subdataset_6|lcl|hemolytic|lcl|cTer
GIGKFIHAAKKFGKLFIGEIMNS
>lcl|2406|lcl|HAPPENN&HAPPENN-RR90&HAPPENN-hard|lcl|subdataset_6|lcl|non-hemolytic
KIRFTRTVSRLLKAALAST
>lcl|1415|lcl|HAPPENN|lcl|subdataset_6|lcl|hemolytic|lcl|cTer
INWLKLGKKLLSAL
>lcl|1809|lcl|HAPPENN|lcl|subdataset_6|lcl|non-hemolytic|lcl|cTer
GRFRRLRKKTRKRLKKIGKVLKAI
>lcl|302|lcl|HAPPENN&HAPPENN-hard|lcl|subdataset_10|lcl|non-hemolytic
MLSLSDAVQLMLGFGTFVLMLLGLVVELIKNSNKK
>lcl|2238|lcl|HAPPENN|lcl|subdataset_7|lcl|hemolytic|lcl|cTer
WKRWVQRWKRWLR
>lcl|2775|lcl|HAPPENN&HAPPENN-RR90&HAPPENN-hard|lcl|subdataset_4|lcl|hemolytic
GLPICGETCVGGTCNTPGCFCTWPVCTRN
>lcl|3691|lcl|HAPPENN&HAPPENN-RR90&HAPPENN-hard|lcl|subdataset_9|lcl|hemolytic
FLWALFKVASK
>lcl|3295|lcl|HAPPENN|lcl|subdataset_4|lcl|hemolytic
GFSSIFRGVAKFASKGLGKDLAKLGVDLVA
>lcl|38|lcl|HAPPENN|lcl|subdataset_10|lcl|non-hemolytic|lcl|cTer
RGLRRLGRKIAHGVKKY
>lcl|2731|lcl|HAPPENN&HAPPENN-RR90&HAPPENN-hard|lcl|subdataset_7|lcl|non-hemolytic
GANAAKKFATIAKKFINYLW
>lcl|1882|lcl|HAPPENN&HAPPENN-RR90|lcl|subdataset_4|lcl|non-hemolytic
RRLCRIVWVIRVCRR
>lcl|1040|lcl|HAPPENN|lcl|subdataset_6|lcl|hemolytic|lcl|cTer
KRAKKFFKKLK
>lcl|2612|lcl|HAPPENN|lcl|subdataset_12|lcl|non-hemolytic|lcl|cTer
KRWWKWWRR
>lcl|4168|lcl|HAPPENN&HAPPENN-RR90&HAPPENN-hard|lcl|subdataset_7|lcl|hemolytic
KKLFKKILKYLKKLFKKILKYLKKLFKKILKYL
>lcl|951|lcl|HAPPENN&HAPPENN-RR90&HAPPENN-hard|lcl|subdataset_10|lcl|non-hemolytic|lcl|cTer
FLPLIGRVASGIL
>lcl|2038|lcl|HAPPENN&HAPPENN-RR90&HAPPENN-hard|lcl|subdataset_11|lcl|non-hemolytic
DNTDSVVKIQSWFRMATARKSYL
>lcl|1566|lcl|HAPPENN&HAPPENN-RR90&HAPPENN-hard|lcl|subdataset_12|lcl|hemolytic|lcl|cTer
CLLKKLLKKLLKKC
>lcl|3566|lcl|HAPPENN|lcl|subdataset_9|lcl|hemolytic|lcl|cTer
LLAGLAANFLPKIFCKITRKC
>lcl|323|lcl|HAPPENN|lcl|subdataset_6|lcl|hemolytic
AKKVFKRLGIGAVLWVLTWG
>lcl|696|lcl|HAPPENN|lcl|subdataset_11|lcl|non-hemolytic|lcl|cTer
KLLKKPLKLKL
>lcl|4707|lcl|HAPPENN|lcl|subdataset_10|lcl|non-hemolytic|lcl|nTer|lcl|cTer
KARRAVRAI
>lcl|1687|lcl|HAPPENN|lcl|subdataset_12|lcl|hemolytic|lcl|cTer
KLALKLALKAWKAALKLA
>lcl|4555|lcl|HAPPENN&HAPPENN-hard|lcl|subdataset_5|lcl|non-hemolytic|lcl|cTer
FLKGIKGMLGKLL
>lcl|1000|lcl|HAPPENN&HAPPENN-hard|lcl|subdataset_4|lcl|non-hemolytic|lcl|cTer
GIIKKIIKKI
>lcl|4376|lcl|HAPPENN&HAPPENN-RR90&HAPPENN-hard|lcl|subdataset_5|lcl|hemolytic
KLRKLFRKLLKLIRKLLR
>lcl|1764|lcl|HAPPENN|lcl|subdataset_7|lcl|hemolytic
GLPTCGETCFKGKCYTPGCSCSYPICKKD
>lcl|1468|lcl|HAPPENN&HAPPENN-RR90&HAPPENN-hard|lcl|subdataset_9|lcl|non-hemolytic
GGLRSLGRKILRAWKKYGPIIVPIIRIG
>lcl|1201|lcl|HAPPENN|lcl|subdataset_8|lcl|hemolytic
```

```
GFLGPLLKLAAKGVAKVIPHLIPSRQQ
>lcl|1328|lcl|HAPPENN|lcl|subdataset_5|lcl|hemolytic|lcl|cTer
LNWGAILKHIIK
>lcl|3937|lcl|HAPPENN&HAPPENN-hard|lcl|subdataset_7|lcl|non-hemolytic
WLRRIKAWLRR
>lcl|3700|lcl|HAPPENN&HAPPENN-RR90&HAPPENN-hard|lcl|subdataset_10|lcl|hemolytic|lcl|nTer|lcl|cTer
WRSLGRTLLRLSHALKPLARRSGW
>lcl|4435|lcl|HAPPENN|lcl|subdataset_4|lcl|non-hemolytic
IKKIWSKIKKLLK
>lcl|2883|lcl|HAPPENN|lcl|subdataset_12|lcl|non-hemolytic
KKKLLFKLKQ
>lcl|2595|lcl|HAPPENN|lcl|subdataset_9|lcl|non-hemolytic
FKTWKRPPFQTSCWGIIKE
>lcl|3114|lcl|HAPPENN&HAPPENN-RR90&HAPPENN-hard|lcl|subdataset_4|lcl|non-hemolytic|lcl|cTer
FFPIVGKRLYGLL
>lcl|403|lcl|HAPPENN&HAPPENN-RR90&HAPPENN-hard|lcl|subdataset_10|lcl|hemolytic
FLPLAVSLAANFLPKLFCKITKKC
>lcl|1777|lcl|HAPPENN&HAPPENN-RR90&HAPPENN-hard|lcl|subdataset_10|lcl|hemolytic
TRWLWLLRGGLKAAGWGIRAHLNRNQ
>lcl|370|lcl|HAPPENN|lcl|subdataset_8|lcl|hemolytic
FKIKPGKVLDKFGKIVGKVLKQLKKVS
>lcl|3058|lcl|HAPPENN|lcl|subdataset_11|lcl|hemolytic|lcl|cTer
HKWMSLLKHILK
>lcl|1177|lcl|HAPPENN|lcl|subdataset_11|lcl|hemolytic
GFGALFKFLAKKVAKTVAKQAAKQGAKYVVNKQME
>lcl|2803|lcl|HAPPENN&HAPPENN-hard|lcl|subdataset_12|lcl|non-hemolytic|lcl|cTer
GICKFLHSAKKFGKAFVGEIMNS
>lcl|961|lcl|HAPPENN|lcl|subdataset_7|lcl|hemolytic|lcl|cTer
WLRRIGKGVKIIGGAALDHL
>lcl|2529|lcl|HAPPENN&HAPPENN-RR90|lcl|subdataset_9|lcl|non-hemolytic|lcl|cTer
AARRILRWIFR
>lcl|147|lcl|HAPPENN&HAPPENN-hard|lcl|subdataset_10|lcl|non-hemolytic
ECYCRRRFCVCVGR
>lcl|2216|lcl|HAPPENN|lcl|subdataset_12|lcl|non-hemolytic|lcl|cTer
LLRIALRKK
>lcl|3573|lcl|HAPPENN&HAPPENN-RR90&HAPPENN-hard|lcl|subdataset_6|lcl|hemolytic|lcl|cTer
FFGSLLKLLPKLL
>lcl|4369|lcl|HAPPENN|lcl|subdataset_5|lcl|non-hemolytic|lcl|cTer
GVVKRIKTVV
>lcl|3110|lcl|HAPPENN|lcl|subdataset_10|lcl|hemolytic|lcl|cTer
GFWGKLWEGVKNAI
>lcl|3634|lcl|HAPPENN&HAPPENN-hard|lcl|subdataset_5|lcl|non-hemolytic
WKLFKKILKVL
>lcl|2943|lcl|HAPPENN&HAPPENN-hard|lcl|subdataset_4|lcl|non-hemolytic
GILDTLKQFAKGVGKDLVKGAAQGVLSTVS
>lcl|1297|lcl|HAPPENN&HAPPENN-RR90&HAPPENN-hard|lcl|subdataset_7|lcl|hemolytic
GLLSGILGAGKHIICGLSGLC
>lcl|4470|lcl|HAPPENN|lcl|subdataset_8|lcl|non-hemolytic|lcl|cTer
GIMSSLMKKLAKIIKK
>lcl|3519|lcl|HAPPENN|lcl|subdataset_8|lcl|non-hemolytic|lcl|cTer
SADLVKKIWDNPAL
>lcl|2762|lcl|HAPPENN|lcl|subdataset_10|lcl|hemolytic
SMLSVLKNLGKVGLGFVACKINKKC
>lcl|1345|lcl|HAPPENN&HAPPENN-RR90&HAPPENN-hard|lcl|subdataset_6|lcl|hemolytic|lcl|cTer
LNWGAGLKHGGK
>lcl|3488|lcl|HAPPENN&HAPPENN-hard|lcl|subdataset_10|lcl|non-hemolytic|lcl|cTer
KGIVGMLGKLF
>lcl|4824|lcl|HAPPENN&HAPPENN-hard|lcl|subdataset_11|lcl|non-hemolytic|lcl|cTer
FRIRVRV
>lcl|1386|lcl|HAPPENN&HAPPENN-RR90&HAPPENN-hard|lcl|subdataset_8|lcl|non-hemolytic
PKLKTFLSKWIG
>lcl|4540|lcl|HAPPENN&HAPPENN-RR90&HAPPENN-hard|lcl|subdataset_8|lcl|hemolytic|lcl|cTer
FFHHFARGIVHVGKTIHRLVTG
>lcl|1923|lcl|HAPPENN&HAPPENN-RR90|lcl|subdataset_6|lcl|non-hemolytic
EPCSPKNNYHDLFYRT
>lcl|4388|lcl|HAPPENN&HAPPENN-hard|lcl|subdataset_8|lcl|non-hemolytic|lcl|cTer
RAWVAWRNR
>lcl|590|lcl|HAPPENN&HAPPENN-RR90|lcl|subdataset_4|lcl|non-hemolytic|lcl|cTer
```

```
KWFRVYRGIYRRR
>lcl|2959|lcl|HAPPENN|lcl|subdataset_7|lcl|hemolytic|lcl|cTer
GLLKRIKALL
>lcl|1871|lcl|HAPPENN&HAPPENN-RR90|lcl|subdataset_6|lcl|non-hemolytic|lcl|cTer
WKKWWKKWWKKW
>lcl|3729|lcl|HAPPENN|lcl|subdataset_12|lcl|hemolytic|lcl|nTer|lcl|cTer
RGLRRLGKKIAHGVKKYGPTVLRIIRIAG
>lcl|2154|lcl|HAPPENN&HAPPENN-hard|lcl|subdataset_12|lcl|non-hemolytic|lcl|cTer
FLKLLKALL
>lcl|3580|lcl|HAPPENN&HAPPENN-RR90|lcl|subdataset_8|lcl|non-hemolytic|lcl|cTer
LLRHVVKILEKYL
>lcl|4285|lcl|HAPPENN&HAPPENN-RR90|lcl|subdataset_7|lcl|non-hemolytic|lcl|cTer
GVIDIIKGAGKDLIAHAIGKLAEKV
>lcl|4513|lcl|HAPPENN&HAPPENN-RR90&HAPPENN-hard|lcl|subdataset_4|lcl|non-hemolytic|lcl|cTer
AKKVFKRLEKLFSKIYNYK
>lcl|517|lcl|HAPPENN&HAPPENN-RR90&HAPPENN-hard|lcl|subdataset_5|lcl|hemolytic|lcl|cTer
GLLGPLLKIAAKVGKNLL
>lcl|151|lcl|HAPPENN|lcl|subdataset_8|lcl|hemolytic
RGGRLCYCRRRFCVCL
>lcl|3361|lcl|HAPPENN|lcl|subdataset_8|lcl|hemolytic|lcl|cTer
ILPWKWPWWPWR
>lcl|3245|lcl|HAPPENN|lcl|subdataset_6|lcl|hemolytic|lcl|cTer
FIHHIIGGLFSVGKHIHSLIHGH
>lcl|2718|lcl|HAPPENN|lcl|subdataset_10|lcl|hemolytic
VRRRRRPR
>lcl|3323|lcl|HAPPENN&HAPPENN-RR90&HAPPENN-hard|lcl|subdataset_6|lcl|hemolytic
KRFWPLVPVAINTVAAGINLYKAIRRK
>lcl|3741|lcl|HAPPENN|lcl|subdataset_5|lcl|hemolytic|lcl|nTer|lcl|cTer
KNLRRIIRKGIHIIKKYG
>lcl|3050|lcl|HAPPENN&HAPPENN-RR90&HAPPENN-hard|lcl|subdataset_7|lcl|hemolytic|lcl|cTer
GKWMSLLKQILK
>lcl|1682|lcl|HAPPENN|lcl|subdataset_10|lcl|non-hemolytic
GLNALKKVFQGIHEAIKKINNHVQ
>lcl|3506|lcl|HAPPENN&HAPPENN-hard|lcl|subdataset_4|lcl|non-hemolytic
NLKAIAALAKKLL
>lcl|1568|lcl|HAPPENN|lcl|subdataset_5|lcl|hemolytic|lcl|cTer
ALWTTMLKKLGKMALHAGKAALGAAADTI
>lcl|978|lcl|HAPPENN&HAPPENN-RR90&HAPPENN-hard|lcl|subdataset_11|lcl|hemolytic
GFLGILFHGVHHGRKKALHMNSERRS
>lcl|2856|lcl|HAPPENN|lcl|subdataset_9|lcl|non-hemolytic|lcl|cTer
KLWKKWKKWKK
>lcl|4638|lcl|HAPPENN&HAPPENN-RR90&HAPPENN-hard|lcl|subdataset_8|lcl|non-hemolytic
AAKIILNPKFRCKAAFC
>lcl|4717|lcl|HAPPENN&HAPPENN-RR90&HAPPENN-hard|lcl|subdataset_7|lcl|hemolytic|lcl|cTer
FPVTFRFFKFFKG
>lcl|1318|lcl|HAPPENN&HAPPENN-hard|lcl|subdataset_4|lcl|non-hemolytic
FLGALFKVASKVLPSVKCAITKKC
>lcl|1545|lcl|HAPPENN&HAPPENN-RR90|lcl|subdataset_5|lcl|non-hemolytic
RYRRKKKMKKALQYIKLLKE
>lcl|3124|lcl|HAPPENN|lcl|subdataset_4|lcl|hemolytic
FLPLIASVAANLVPKIFCKITKKC
>lcl|2260|lcl|HAPPENN&HAPPENN-RR90&HAPPENN-hard|lcl|subdataset_10|lcl|non-hemolytic|lcl|cTer
GIGKFIHSAKRFGRAWVGEIMNS
>lcl|1361|lcl|HAPPENN&HAPPENN-hard|lcl|subdataset_8|lcl|non-hemolytic
GLGSLLGKAFKIGLKTVGKMMGGAPREQ
>lcl|1413|lcl|HAPPENN|lcl|subdataset_12|lcl|hemolytic|lcl|cTer
INSLKLGKKILGAL
>lcl|1829|lcl|HAPPENN&HAPPENN-RR90&HAPPENN-hard|lcl|subdataset_11|lcl|non-hemolytic|lcl|cTer
FLGGLIKPWWPWRR
>lcl|3544|lcl|HAPPENN&HAPPENN-RR90&HAPPENN-hard|lcl|subdataset_6|lcl|hemolytic
SLFGTFAKMALKGASKLIPHLLPSRQQ
>lcl|4378|lcl|HAPPENN|lcl|subdataset_4|lcl|non-hemolytic|lcl|nTer
RGRKVVRRKK
>lcl|2703|lcl|HAPPENN&HAPPENN-RR90&HAPPENN-hard|lcl|subdataset_8|lcl|hemolytic|lcl|cTer
FLPIVTNLLSGLL
>lcl|2375|lcl|HAPPENN&HAPPENN-RR90&HAPPENN-hard|lcl|subdataset_9|lcl|hemolytic|lcl|cTer
```

```
GIGHFLHKVKSFGKSWIGEIMNS
>lcl|3430|lcl|HAPPENN|lcl|subdataset_4|lcl|hemolytic
FSISPGKVLDKFGKIVGKVLKQLKKVSAVAKV
>lcl|4124|lcl|HAPPENN&HAPPENN-RR90&HAPPENN-hard|lcl|subdataset_5|lcl|hemolytic|lcl|cTer
KWKVFKKIEKMGRNIRNGIVKAGPKWKVFKKIEK
>lcl|3360|lcl|HAPPENN|lcl|subdataset_11|lcl|hemolytic|lcl|nTer|lcl|cTer
ILPWKWPWWPWRR
>lcl|1094|lcl|HAPPENN&HAPPENN-RR90&HAPPENN-hard|lcl|subdataset_11|lcl|hemolytic
FLPFLLSALPKVFCFFSKKC
>lcl|2079|lcl|HAPPENN&HAPPENN-RR90&HAPPENN-hard|lcl|subdataset_10|lcl|non-hemolytic
GLVGTLLGHIGKAILGG
>lcl|4082|lcl|HAPPENN&HAPPENN-RR90&HAPPENN-hard|lcl|subdataset_11|lcl|non-hemolytic
GLARGLREASKALDR
>lcl|3826|lcl|HAPPENN&HAPPENN-RR90&HAPPENN-hard|lcl|subdataset_8|lcl|non-hemolytic|lcl|cTer
KRKLTLVFGVMAGVIGTKKR
>lcl|1392|lcl|HAPPENN&HAPPENN-hard|lcl|subdataset_9|lcl|non-hemolytic|lcl|cTer
KWKLFKKIGIGAVLKVLTTG
>lcl|3235|lcl|HAPPENN|lcl|subdataset_5|lcl|hemolytic|lcl|cTer
GLVTSLIKGAGKLLGGLFGSVTG
>lcl|3970|lcl|HAPPENN|lcl|subdataset_4|lcl|non-hemolytic|lcl|cTer
GVVDILKGAAKDIAGHLASKAMNKL
>lcl|3056|lcl|HAPPENN&HAPPENN-RR90&HAPPENN-hard|lcl|subdataset_7|lcl|hemolytic|lcl|cTer
GKWLSLLKHILK
>lcl|2455|lcl|HAPPENN&HAPPENN-RR90|lcl|subdataset_7|lcl|non-hemolytic|lcl|nTer|lcl|cTer
PIHRRIPPRWPRLKRRW
>lcl|3775|lcl|HAPPENN&HAPPENN-RR90|lcl|subdataset_10|lcl|non-hemolytic
KKPWWKPWWPKWKK
>lcl|780|lcl|HAPPENN&HAPPENN-RR90&HAPPENN-hard|lcl|subdataset_7|lcl|hemolytic|lcl|cTer
FLPIVGRLISGLL
>lcl|4409|lcl|HAPPENN|lcl|subdataset_9|lcl|hemolytic|lcl|cTer
RIWFQNRRMRWRR
>lcl|592|lcl|HAPPENN|lcl|subdataset_5|lcl|hemolytic
FPFSLIPHAIGGLISAIK
>lcl|2257|lcl|HAPPENN&HAPPENN-hard|lcl|subdataset_9|lcl|non-hemolytic
GIMDTVKNAAKNLAGQLLDKLKCSITAC
>lcl|1870|lcl|HAPPENN|lcl|subdataset_7|lcl|non-hemolytic|lcl|cTer
WKKWWKKWWK
>lcl|998|lcl|HAPPENN|lcl|subdataset_6|lcl|non-hemolytic
SWLRDIWDWECEVLSDFK
>lcl|1976|lcl|HAPPENN&HAPPENN-RR90&HAPPENN-hard|lcl|subdataset_8|lcl|non-hemolytic|lcl|cTer
VTCYCRRTRCGFRERLSGACGYRGRIYRLCCR
>lcl|3419|lcl|HAPPENN&HAPPENN-RR90&HAPPENN-hard|lcl|subdataset_10|lcl|hemolytic
KKLIKKILKIL
>lcl|1119|lcl|HAPPENN|lcl|subdataset_6|lcl|non-hemolytic
LMAAKAESRK
>lcl|2889|lcl|HAPPENN&HAPPENN-RR90|lcl|subdataset_4|lcl|non-hemolytic
KRFKKFFKKLKNSVKKRAKKFFKKPRVIGVSIPF
>lcl|1195|lcl|HAPPENN&HAPPENN-RR90|lcl|subdataset_4|lcl|non-hemolytic
KKLKKLLKKWKKLLKKLK
>lcl|630|lcl|HAPPENN|lcl|subdataset_11|lcl|non-hemolytic|lcl|cTer
WFKKIPKFLHLAKKF
>lcl|3408|lcl|HAPPENN&HAPPENN-hard|lcl|subdataset_10|lcl|non-hemolytic|lcl|cTer
HFLGTLKNLAKKIL
>lcl|2653|lcl|HAPPENN|lcl|subdataset_11|lcl|hemolytic
GLWNTIKEAGKKFAINVLDKIRCGIAGGCKT
>lcl|4258|lcl|HAPPENN|lcl|subdataset_12|lcl|non-hemolytic
KTRNWFSEHFKKVKEKLKDTFA
>lcl|1890|lcl|HAPPENN|lcl|subdataset_5|lcl|hemolytic|lcl|cTer
FLPIVAKLLSGLL
>lcl|4357|lcl|HAPPENN&HAPPENN-RR90&HAPPENN-hard|lcl|subdataset_5|lcl|non-hemolytic|lcl|cTer
GKLTKDKLKRGAKKALNVASKVAPIVAAGASIAR
>lcl|3446|lcl|HAPPENN&HAPPENN-RR90&HAPPENN-hard|lcl|subdataset_9|lcl|hemolytic
GLPTCGETCFGGTCNTPGCSCSSWPICTRN
>lcl|4071|lcl|HAPPENN&HAPPENN-RR90&HAPPENN-hard|lcl|subdataset_4|lcl|hemolytic|lcl|cTer
KKYLLPIVGNLLKSLL
>lcl|381|lcl|HAPPENN|lcl|subdataset_8|lcl|non-hemolytic
```

```
KLKLKLKQ
>lcl|1671|lcl|HAPPENN&HAPPENN-RR90|lcl|subdataset_10|lcl|non-hemolytic|lcl|cTer
KLPLLPWLPKK
>lcl|56|lcl|HAPPENN|lcl|subdataset_11|lcl|non-hemolytic|lcl|nTer|lcl|cTer
KWKSFAKTFKSAKKTVLHTAAKAISS
>lcl|2256|lcl|HAPPENN&HAPPENN-hard|lcl|subdataset_5|lcl|non-hemolytic
GIMDTVKNAAKNLAGQLLDKLK
>lcl|2907|lcl|HAPPENN|lcl|subdataset_6|lcl|non-hemolytic
PRPPHPPRPPHPRL
>lcl|4370|lcl|HAPPENN|lcl|subdataset_6|lcl|non-hemolytic|lcl|cTer
GMMKRIKTMM
>lcl|497|lcl|HAPPENN&HAPPENN-hard|lcl|subdataset_8|lcl|non-hemolytic
AKKVFKRLEKLFSKIWNDK
>lcl|1985|lcl|HAPPENN|lcl|subdataset_10|lcl|non-hemolytic
KLNRLKEKNKAKNSKENN
>lcl|1794|lcl|HAPPENN|lcl|subdataset_6|lcl|hemolytic|lcl|cTer
WRWLRRIW
>lcl|1179|lcl|HAPPENN|lcl|subdataset_12|lcl|hemolytic|lcl|cTer
ILGPVISTIGGVLGGLLKNL
>lcl|536|lcl|HAPPENN&HAPPENN-RR90&HAPPENN-hard|lcl|subdataset_6|lcl|hemolytic|lcl|cTer
ILPILGNLLNSLL
>lcl|4339|lcl|HAPPENN|lcl|subdataset_5|lcl|non-hemolytic
WGIRRILKYGKRS
>lcl|1093|lcl|HAPPENN&HAPPENN-RR90&HAPPENN-hard|lcl|subdataset_8|lcl|hemolytic|lcl|cTer
FLGALLGPLMNLLQ
>lcl|833|lcl|HAPPENN|lcl|subdataset_5|lcl|hemolytic
GLFDIIKKIAESF
>lcl|4808|lcl|HAPPENN&HAPPENN-RR90&HAPPENN-hard|lcl|subdataset_12|lcl|non-hemolytic|lcl|cTer
INWKKIKSIIKAAMN
>lcl|1810|lcl|HAPPENN|lcl|subdataset_6|lcl|non-hemolytic|lcl|cTer
GRFRRLRKKTRKRLKKIGKVLKKI
>lcl|2561|lcl|HAPPENN&HAPPENN-RR90&HAPPENN-hard|lcl|subdataset_10|lcl|non-hemolytic
FLIGMTHGLICLISRKC
>lcl|4359|lcl|HAPPENN&HAPPENN-RR90&HAPPENN-hard|lcl|subdataset_12|lcl|hemolytic
KRFWQLVPLAIKIYRAWKRR
>lcl|4291|lcl|HAPPENN&HAPPENN-RR90&HAPPENN-hard|lcl|subdataset_5|lcl|non-hemolytic|lcl|cTer
FKRLKKLISWIKRKRQQ
>lcl|1692|lcl|HAPPENN|lcl|subdataset_7|lcl|non-hemolytic|lcl|cTer
KKLFKKILKIL
>lcl|3164|lcl|HAPPENN&HAPPENN-RR90|lcl|subdataset_12|lcl|non-hemolytic|lcl|cTer
GMASKAGSVLGKVAKVALKAAL
>lcl|1033|lcl|HAPPENN&HAPPENN-RR90|lcl|subdataset_5|lcl|non-hemolytic
DANVENGEDAEDLTDKFIGLMG
>lcl|150|lcl|HAPPENN|lcl|subdataset_5|lcl|hemolytic
RGGRLCYCRRRFCVCA
>lcl|3115|lcl|HAPPENN&HAPPENN-RR90&HAPPENN-hard|lcl|subdataset_4|lcl|hemolytic|lcl|cTer
FLSTLLKVAFKVVPTLFCPITKKC
>lcl|1640|lcl|HAPPENN&HAPPENN-RR90&HAPPENN-hard|lcl|subdataset_8|lcl|hemolytic
GLWTTIKEGLKKFSLGVLDKIRCKIAGGC
>lcl|2713|lcl|HAPPENN&HAPPENN-RR90&HAPPENN-hard|lcl|subdataset_12|lcl|hemolytic
KWKVFKKIEKNGRNIRNGIVKAGPAIAVLGEAKAL
>lcl|4093|lcl|HAPPENN|lcl|subdataset_9|lcl|non-hemolytic
SAVGRHLRRFLLRKHRKH
>lcl|2029|lcl|HAPPENN|lcl|subdataset_11|lcl|hemolytic
FKFGSFIKRMWRSKLAKKLRAKGKELLRDYA
>lcl|2841|lcl|HAPPENN|lcl|subdataset_10|lcl|non-hemolytic|lcl|cTer
KLWKKWKKWLK
>lcl|1686|lcl|HAPPENN&HAPPENN-hard|lcl|subdataset_10|lcl|non-hemolytic
GLNALKKVFQPIHKAIKKINNHVQ
>lcl|1350|lcl|HAPPENN&HAPPENN-RR90|lcl|subdataset_11|lcl|non-hemolytic|lcl|cTer
KAYSMPRCKGGFRAVMCWL
>lcl|658|lcl|HAPPENN|lcl|subdataset_12|lcl|hemolytic
VRRFAWWWAFLRR
>lcl|3513|lcl|HAPPENN&HAPPENN-hard|lcl|subdataset_9|lcl|non-hemolytic
FLPIIGKLLSG
>lcl|2744|lcl|HAPPENN|lcl|subdataset_4|lcl|hemolytic
IWLTALKFLGKHAAKHLAKQQLSKL
>lcl|1315|lcl|HAPPENN&HAPPENN-hard|lcl|subdataset_10|lcl|non-hemolytic
GIMSIVKDVAKNAAKEAAKGALSTLSCKLAKTC
```

```
>lcl|1365|lcl|HAPPENN&HAPPENN-RR90&HAPPENN-hard|lcl|subdataset_9|lcl|hemolytic
GFFALIPKIISSPLFKTLLSAVGSALSSSGEQE
>lcl|1672|lcl|HAPPENN|lcl|subdataset_8|lcl|non-hemolytic|lcl|cTer
KLPLLPWLPLK
>lcl|2014|lcl|HAPPENN&HAPPENN-RR90&HAPPENN-hard|lcl|subdataset_8|lcl|non-hemolytic|lcl|cTer
GAKLAKKQVRALGKFFSF
>lcl|3335|lcl|HAPPENN|lcl|subdataset_11|lcl|non-hemolytic|lcl|cTer
QLKVDLWGTR
>lcl|2397|lcl|HAPPENN|lcl|subdataset_10|lcl|hemolytic|lcl|cTer
FLSLIPKAISAISALANHF
>lcl|743|lcl|HAPPENN&HAPPENN-RR90&HAPPENN-hard|lcl|subdataset_12|lcl|non-hemolytic|lcl|cTer
ILGKIWKIKKLF
>lcl|1139|lcl|HAPPENN|lcl|subdataset_10|lcl|non-hemolytic
RKKRRQRRR
>lcl|2668|lcl|HAPPENN|lcl|subdataset_9|lcl|non-hemolytic|lcl|cTer
ALWLAIRKR
>lcl|3097|lcl|HAPPENN&HAPPENN-RR90&HAPPENN-hard|lcl|subdataset_9|lcl|hemolytic|lcl|cTer
NKWKKILGKIIKVVK
>lcl|3125|lcl|HAPPENN&HAPPENN-RR90&HAPPENN-hard|lcl|subdataset_10|lcl|hemolytic
SLLGTVKDLLIGAGKSAAQSVLKGLSCKLSKDC
>lcl|164|lcl|HAPPENN|lcl|subdataset_10|lcl|non-hemolytic
LCYCRRRFCGCV
>lcl|2360|lcl|HAPPENN|lcl|subdataset_6|lcl|non-hemolytic|lcl|cTer
KRIVQRIKDFLA
>lcl|1103|lcl|HAPPENN&HAPPENN-RR90&HAPPENN-hard|lcl|subdataset_11|lcl|hemolytic|lcl|cTer
ILGKLLKTAAGLLSNL
>lcl|3364|lcl|HAPPENN|lcl|subdataset_9|lcl|hemolytic|lcl|cTer
ILPWKWPWW
>lcl|3132|lcl|HAPPENN&HAPPENN-RR90&HAPPENN-hard|lcl|subdataset_4|lcl|hemolytic|lcl|cTer
FLPIITNLLGKLL
>lcl|84|lcl|HAPPENN|lcl|subdataset_7|lcl|hemolytic|lcl|cTer
NFLGTLVNLAKKIL
>lcl|4718|lcl|HAPPENN&HAPPENN-RR90|lcl|subdataset_4|lcl|non-hemolytic|lcl|cTer
FPVTWPTKWWKS
>lcl|2761|lcl|HAPPENN|lcl|subdataset_8|lcl|hemolytic
SMLSVLKNLGKVGLGFVACKIKKQC
>lcl|4257|lcl|HAPPENN&HAPPENN-RR90|lcl|subdataset_6|lcl|non-hemolytic
FSTKTRNWFSEHFKKVKEKLKDTFA
>lcl|2472|lcl|HAPPENN&HAPPENN-RR90|lcl|subdataset_11|lcl|non-hemolytic|lcl|nTer|lcl|cTer
FKLKLKFSNKLKWKW
>lcl|3008|lcl|HAPPENN&HAPPENN-RR90|lcl|subdataset_8|lcl|non-hemolytic|lcl|cTer
KLAGKLAKWAGKLAKLAGKLA
>lcl|1769|lcl|HAPPENN|lcl|subdataset_11|lcl|non-hemolytic|lcl|cTer
RLLKFIKKLL
>lcl|3643|lcl|HAPPENN&HAPPENN-RR90&HAPPENN-hard|lcl|subdataset_8|lcl|hemolytic
GIMDTIKGAAKDLAGQLLDKLKCKITKC
>lcl|163|lcl|HAPPENN|lcl|subdataset_11|lcl|non-hemolytic
LCYCRRRFCTCV
>lcl|4266|lcl|HAPPENN|lcl|subdataset_6|lcl|hemolytic|lcl|cTer
FLKGIVGMLGKLF
>lcl|1793|lcl|HAPPENN|lcl|subdataset_7|lcl|hemolytic|lcl|cTer
RWWLRRIW
>lcl|4769|lcl|HAPPENN|lcl|subdataset_12|lcl|hemolytic
WWWLRKIW
>lcl|3861|lcl|HAPPENN|lcl|subdataset_6|lcl|non-hemolytic|lcl|cTer
KRKKLLKRLI
>lcl|1648|lcl|HAPPENN&HAPPENN-RR90&HAPPENN-hard|lcl|subdataset_7|lcl|non-hemolytic
FKTWKNRPILSSCSGIIKG
>lcl|4269|lcl|HAPPENN&HAPPENN-RR90|lcl|subdataset_5|lcl|non-hemolytic|lcl|cTer
RWKIFKKIPKFLHSAKKF
>lcl|1245|lcl|HAPPENN&HAPPENN-hard|lcl|subdataset_12|lcl|non-hemolytic|lcl|cTer
GIGKFLHSAKKFGKAWVGEIMNS
>lcl|2820|lcl|HAPPENN|lcl|subdataset_5|lcl|non-hemolytic|lcl|cTer
ALRLAIRRR
>lcl|1919|lcl|HAPPENN&HAPPENN-RR90|lcl|subdataset_10|lcl|non-hemolytic
YDLSDSNCLPANRDKRYYVI
>lcl|509|lcl|HAPPENN&HAPPENN-RR90&HAPPENN-hard|lcl|subdataset_7|lcl|non-hemolytic|lcl|
```

cTer
GFKDLLKGAKKALVKTVLF
>lcl|1182|lcl|HAPPENN&HAPPENN-RR90&HAPPENN-hard|lcl|subdataset_11|lcl|hemolytic|lcl|cTer
ILGPVISKIGGVLGGLLKNL
>lcl|3773|lcl|HAPPENN|lcl|subdataset_8|lcl|non-hemolytic
KKTWWKTWWTKW
>lcl|2437|lcl|HAPPENN|lcl|subdataset_12|lcl|non-hemolytic|lcl|cTer
RWKIFKKVVKKW
>lcl|1259|lcl|HAPPENN|lcl|subdataset_5|lcl|hemolytic
IISTIGDLVKWIIKTV
>lcl|3246|lcl|HAPPENN|lcl|subdataset_5|lcl|hemolytic|lcl|cTer
FIHHIIGGLFSAGKAIHRLIRRRRR
>lcl|4831|lcl|HAPPENN&HAPPENN-RR90&HAPPENN-hard|lcl|subdataset_8|lcl|hemolytic|lcl|cTer
FFGHLFRGIINVGKHIHGLLSG
>lcl|292|lcl|HAPPENN|lcl|subdataset_12|lcl|hemolytic
PAICQRATATLGTVGSNTSGTTAIEACILL
>lcl|582|lcl|HAPPENN&HAPPENN-RR90|lcl|subdataset_7|lcl|non-hemolytic
KRFKQDGGWSHWSPWSS
>lcl|1088|lcl|HAPPENN&HAPPENN-RR90&HAPPENN-hard|lcl|subdataset_7|lcl|hemolytic
GFWTTAAEGLKKFAKAGLASILNPK
>lcl|3720|lcl|HAPPENN|lcl|subdataset_10|lcl|non-hemolytic|lcl|nTer|lcl|cTer
KKTWWKTWWTKWSQPKK
>lcl|610|lcl|HAPPENN&HAPPENN-RR90&HAPPENN-hard|lcl|subdataset_5|lcl|non-hemolytic
GIWGTALKWGVKLLPKLVGMAQTKKQ
>lcl|920|lcl|HAPPENN&HAPPENN-RR90|lcl|subdataset_6|lcl|non-hemolytic|lcl|cTer
KRLRRVWRRWR
>lcl|4755|lcl|HAPPENN&HAPPENN-RR90&HAPPENN-hard|lcl|subdataset_5|lcl|non-hemolytic|lcl|cTer
FLSLIPKAIKAVGVKAKKF
>lcl|810|lcl|HAPPENN&HAPPENN-RR90&HAPPENN-hard|lcl|subdataset_8|lcl|hemolytic
LFGFLIKLIPSLFGALSNIGRNRNQ
>lcl|3824|lcl|HAPPENN&HAPPENN-RR90&HAPPENN-hard|lcl|subdataset_5|lcl|non-hemolytic|lcl|cTer
LKALRKALKHLA
>lcl|2708|lcl|HAPPENN&HAPPENN-RR90|lcl|subdataset_7|lcl|non-hemolytic|lcl|cTer
KKKVVFKVKFKKK
>lcl|4644|lcl|HAPPENN&HAPPENN-RR90|lcl|subdataset_5|lcl|non-hemolytic
CKKLLKLCKKLLKLAG
>lcl|956|lcl|HAPPENN&HAPPENN-RR90&HAPPENN-hard|lcl|subdataset_4|lcl|non-hemolytic
FKCRRWQWRMKKLGAPSITCVRRAF
>lcl|2815|lcl|HAPPENN&HAPPENN-RR90|lcl|subdataset_11|lcl|non-hemolytic|lcl|cTer
LLAAHLLAIGRR
>lcl|291|lcl|HAPPENN|lcl|subdataset_7|lcl|hemolytic
PAICQRATATLGTVGSNTSGTTEIEAAILL
>lcl|4492|lcl|HAPPENN&HAPPENN-RR90&HAPPENN-hard|lcl|subdataset_6|lcl|hemolytic
GMAKLLAKVLPHVVKLIK
>lcl|2879|lcl|HAPPENN|lcl|subdataset_7|lcl|non-hemolytic
KKKLKFLLKQ
>lcl|2895|lcl|HAPPENN|lcl|subdataset_9|lcl|non-hemolytic
QPRPPHPRL
>lcl|1180|lcl|HAPPENN&HAPPENN-RR90&HAPPENN-hard|lcl|subdataset_11|lcl|hemolytic|lcl|cTer
ILGPVLSMVGSALGGLIKKI
>lcl|948|lcl|HAPPENN&HAPPENN-RR90&HAPPENN-hard|lcl|subdataset_5|lcl|hemolytic|lcl|cTer
FLPLIARVLSGIL
>lcl|2385|lcl|HAPPENN|lcl|subdataset_11|lcl|non-hemolytic
KKWRWWLKALAKK
>lcl|3849|lcl|HAPPENN&HAPPENN-RR90|lcl|subdataset_12|lcl|non-hemolytic|lcl|cTer
RKKRLRVVRRLV
>lcl|4116|lcl|HAPPENN&HAPPENN-RR90|lcl|subdataset_4|lcl|non-hemolytic|lcl|cTer
RFRRLRWRTRWRLRRI
>lcl|3346|lcl|HAPPENN|lcl|subdataset_4|lcl|non-hemolytic
GIIKKIIKKI
>lcl|1282|lcl|HAPPENN&HAPPENN-RR90&HAPPENN-hard|lcl|subdataset_11|lcl|non-hemolytic
GLLNGLALRLGKRALKKIIKRLCR
>lcl|614|lcl|HAPPENN&HAPPENN-RR90&HAPPENN-hard|lcl|subdataset_4|lcl|non-hemolytic
GWKDWLNKGKEWLKKKGPGIMKAALKAATQ
>lcl|1423|lcl|HAPPENN|lcl|subdataset_6|lcl|hemolytic|lcl|cTer
WKKIASIGKEVLKAL
>lcl|2167|lcl|HAPPENN&HAPPENN-RR90&HAPPENN-hard|lcl|subdataset_5|lcl|hemolytic|lcl|cTer

```
KWKVFKKAEKMIRNIRNKIVK
>lcl|812|lcl|HAPPENN&HAPPENN-RR90&HAPPENN-hard|lcl|subdataset_4|lcl|non-hemolytic|lcl|cTer
GVIIDTLKGAAKTVAAELLRKAHCKLTNSC
>lcl|493|lcl|HAPPENN|lcl|subdataset_5|lcl|non-hemolytic
AKKVFKRLEKLFS
>lcl|2501|lcl|HAPPENN&HAPPENN-hard|lcl|subdataset_8|lcl|non-hemolytic
KLLSHSLLVTLA
>lcl|3512|lcl|HAPPENN&HAPPENN-hard|lcl|subdataset_10|lcl|non-hemolytic
FLPIIGKLLSGLL
>lcl|2435|lcl|HAPPENN|lcl|subdataset_10|lcl|non-hemolytic|lcl|cTer
RWKIFKKAAKKA
>lcl|3690|lcl|HAPPENN&HAPPENN-hard|lcl|subdataset_8|lcl|non-hemolytic
FWGALFKVASK
>lcl|4313|lcl|HAPPENN|lcl|subdataset_5|lcl|hemolytic
GIGKFLHSAKKFGKAFVAEIMNS
>lcl|1848|lcl|HAPPENN&HAPPENN-RR90|lcl|subdataset_8|lcl|non-hemolytic
RRRCPIVVIRVCRR
>lcl|3409|lcl|HAPPENN&HAPPENN-RR90&HAPPENN-hard|lcl|subdataset_5|lcl|non-hemolytic|lcl|cTer
HFLGTKVNLAKKIL
>lcl|970|lcl|HAPPENN&HAPPENN-RR90&HAPPENN-hard|lcl|subdataset_6|lcl|hemolytic|lcl|cTer
GWKKWFNRAKKVGKTVGGLAVDHYL
>lcl|1222|lcl|HAPPENN|lcl|subdataset_8|lcl|hemolytic
CIGAVLKVLTTGLPALISWIKRKRQQ
>lcl|439|lcl|HAPPENN&HAPPENN-RR90&HAPPENN-hard|lcl|subdataset_9|lcl|hemolytic
WKKVFKRLEKLFSKIWNWK
>lcl|606|lcl|HAPPENN&HAPPENN-RR90&HAPPENN-hard|lcl|subdataset_7|lcl|non-hemolytic
GWGSFFKKAAAVGKAVGKAALTAYL
>lcl|934|lcl|HAPPENN|lcl|subdataset_9|lcl|hemolytic|lcl|cTer
LLQWLSKLLGRWL
>lcl|2068|lcl|HAPPENN&HAPPENN-RR90&HAPPENN-hard|lcl|subdataset_8|lcl|hemolytic|lcl|cTer
CLNLKALLAVAKKILC
>lcl|3921|lcl|HAPPENN&HAPPENN-RR90|lcl|subdataset_7|lcl|non-hemolytic
RIIRPIIQIIKQKIR
>lcl|3845|lcl|HAPPENN|lcl|subdataset_4|lcl|non-hemolytic
GLNALKKVFQGFHEAIKLINNHVQ
>lcl|100|lcl|HAPPENN|lcl|subdataset_5|lcl|non-hemolytic|lcl|cTer
KKKKKKAAFALWLAFLA
>lcl|372|lcl|HAPPENN|lcl|subdataset_10|lcl|hemolytic
GLFGKILGVGKKVLCGLSGMC
>lcl|2247|lcl|HAPPENN&HAPPENN-RR90&HAPPENN-hard|lcl|subdataset_11|lcl|hemolytic
GLPTCGETCFGGTCNTPGCTCDPWPVCTHN
>lcl|1036|lcl|HAPPENN|lcl|subdataset_4|lcl|hemolytic
SRWRDVARNFMRRYQSRVIQGLVA
>lcl|2476|lcl|HAPPENN&HAPPENN-RR90&HAPPENN-hard|lcl|subdataset_5|lcl|hemolytic
GIWKKWIKKWLKKLLKKLWKKG
>lcl|1590|lcl|HAPPENN|lcl|subdataset_5|lcl|non-hemolytic|lcl|cTer
KIAGKIAKIAKKIAK
>lcl|2318|lcl|HAPPENN&HAPPENN-RR90&HAPPENN-hard|lcl|subdataset_10|lcl|hemolytic|lcl|cTer
FLSLIPHIVSGVAALANHL
>lcl|2265|lcl|HAPPENN&HAPPENN-hard|lcl|subdataset_5|lcl|non-hemolytic
GLPVCGETCVGGTCNYPGCTCSWPVCTRN
>lcl|3966|lcl|HAPPENN|lcl|subdataset_5|lcl|non-hemolytic|lcl|cTer
GVVDILKGAAKDIAGHLASKVMNAL
>lcl|1772|lcl|HAPPENN&HAPPENN-RR90&HAPPENN-hard|lcl|subdataset_5|lcl|hemolytic
IVPFLLGMVPKLVCLITKKC
>lcl|4624|lcl|HAPPENN|lcl|subdataset_9|lcl|non-hemolytic
GRRPRPRPRPFFFF
>lcl|979|lcl|HAPPENN&HAPPENN-RR90&HAPPENN-hard|lcl|subdataset_8|lcl|hemolytic|lcl|cTer
GWKDWFRKAKKVGKTVGGLALNHYL
>lcl|4762|lcl|HAPPENN|lcl|subdataset_9|lcl|hemolytic|lcl|cTer
FLGELWNVWKSVF
>lcl|1577|lcl|HAPPENN|lcl|subdataset_9|lcl|hemolytic|lcl|cTer
ALYKTMLKKLGTMAL
>lcl|2511|lcl|HAPPENN&HAPPENN-RR90|lcl|subdataset_7|lcl|non-hemolytic|lcl|cTer
LKGKLWLKGKL
>lcl|3717|lcl|HAPPENN|lcl|subdataset_11|lcl|non-hemolytic|lcl|nTer|lcl|cTer
RGRGKQGGKVRAKAKTRSS
>lcl|3485|lcl|HAPPENN|lcl|subdataset_12|lcl|non-hemolytic
IASKVANTVQKLKRKAKNAVA
```

```
>lcl|2816|lcl|HAPPENN|lcl|subdataset_9|lcl|non-hemolytic|lcl|cTer
AAHCLAIGRR
>lcl|4447|lcl|HAPPENN&HAPPENN-hard|lcl|subdataset_10|lcl|non-hemolytic|lcl|cTer
IDGLKAIWKKVADLLKNT
>lcl|2508|lcl|HAPPENN|lcl|subdataset_5|lcl|non-hemolytic|lcl|cTer
KKLGWGLKK
>lcl|762|lcl|HAPPENN|lcl|subdataset_6|lcl|non-hemolytic|lcl|cTer
RGGRLAYCRRRFCVAVGR
>lcl|3671|lcl|HAPPENN&HAPPENN-RR90|lcl|subdataset_6|lcl|non-hemolytic
RRRFVAEQDAIHSRVSREVPTLSDSV
>lcl|3900|lcl|HAPPENN|lcl|subdataset_6|lcl|hemolytic|lcl|cTer
GIWDTIKSMGKVFAGKIKQNL
>lcl|4514|lcl|HAPPENN|lcl|subdataset_11|lcl|non-hemolytic
RIKRFWPVVIR
>lcl|1977|lcl|HAPPENN&HAPPENN-RR90&HAPPENN-hard|lcl|subdataset_6|lcl|hemolytic|lcl|cTer
KALAALLKKWAKLLAALK
>lcl|4688|lcl|HAPPENN|lcl|subdataset_12|lcl|non-hemolytic|lcl|cTer
IRVKIRVK
>lcl|1565|lcl|HAPPENN|lcl|subdataset_12|lcl|hemolytic|lcl|cTer
LLKKLLKKLLKKC
>lcl|2263|lcl|HAPPENN|lcl|subdataset_9|lcl|non-hemolytic
YYHFWHRGVTKRSLSPHRPRHSR
>lcl|2906|lcl|HAPPENN&HAPPENN-RR90|lcl|subdataset_10|lcl|non-hemolytic
RREAEPEAEPGNNRPVYIPQPRPPHPRL
>lcl|1737|lcl|HAPPENN&HAPPENN-hard|lcl|subdataset_10|lcl|non-hemolytic|lcl|cTer
FWRIRKWR
>lcl|3994|lcl|HAPPENN|lcl|subdataset_4|lcl|hemolytic|lcl|nTer|lcl|cTer
FIFHIIKGLFHAGKMI
>lcl|4371|lcl|HAPPENN&HAPPENN-hard|lcl|subdataset_7|lcl|non-hemolytic|lcl|cTer
GVLKRIKTLV
>lcl|4355|lcl|HAPPENN&HAPPENN-hard|lcl|subdataset_7|lcl|non-hemolytic|lcl|cTer
GLLSRIKTLL
>lcl|1997|lcl|HAPPENN|lcl|subdataset_11|lcl|hemolytic|lcl|cTer
KIAKGALKALKIAKGALKAL
>lcl|3692|lcl|HAPPENN|lcl|subdataset_8|lcl|non-hemolytic|lcl|nTer|lcl|cTer
FKRLEKLFSKIWNWK
>lcl|709|lcl|HAPPENN|lcl|subdataset_4|lcl|hemolytic|lcl|cTer
GLLALISWIKRKRQQ
>lcl|2011|lcl|HAPPENN&HAPPENN-RR90&HAPPENN-hard|lcl|subdataset_12|lcl|non-hemolytic|lcl|cTer
GLLFKELQKLIRYQIFIGK
>lcl|2198|lcl|HAPPENN|lcl|subdataset_6|lcl|non-hemolytic
GGLRSLGRKILRAWKKYG
>lcl|3568|lcl|HAPPENN&HAPPENN-hard|lcl|subdataset_9|lcl|non-hemolytic
IISTIGDLVKWIIDTV
>lcl|3808|lcl|HAPPENN|lcl|subdataset_6|lcl|hemolytic|lcl|cTer
RRLFRRILRYL
>lcl|3960|lcl|HAPPENN&HAPPENN-RR90&HAPPENN-hard|lcl|subdataset_7|lcl|hemolytic|lcl|cTer
LVKKLLKLAMGFG
>lcl|2212|lcl|HAPPENN&HAPPENN-hard|lcl|subdataset_11|lcl|non-hemolytic|lcl|cTer
GVWGIAKIAGKVLGNILPHVFSSNQS
>lcl|1442|lcl|HAPPENN|lcl|subdataset_10|lcl|hemolytic
RVKRVWPLVIRTVIALYNLYRAIKKK
>lcl|3567|lcl|HAPPENN|lcl|subdataset_5|lcl|hemolytic|lcl|cTer
GLAANFLPKIFCKITRKC
>lcl|3766|lcl|HAPPENN|lcl|subdataset_4|lcl|non-hemolytic|lcl|cTer
YRWARWWRR
>lcl|2967|lcl|HAPPENN|lcl|subdataset_12|lcl|non-hemolytic|lcl|cTer
KRIKTLL
>lcl|3551|lcl|HAPPENN&HAPPENN-RR90&HAPPENN-hard|lcl|subdataset_12|lcl|hemolytic
GWLPTFGKILRKAMQLGPKLIQPI
>lcl|4316|lcl|HAPPENN&HAPPENN-RR90&HAPPENN-hard|lcl|subdataset_4|lcl|non-hemolytic|lcl|cTer
GKPIGNAILGLRPKISRWL
>lcl|2795|lcl|HAPPENN|lcl|subdataset_12|lcl|hemolytic|lcl|cTer
KWKLFKKIGIGAVLKVLTTGLPALIS
>lcl|4542|lcl|HAPPENN&HAPPENN-RR90&HAPPENN-hard|lcl|subdataset_12|lcl|non-hemolytic
LKAAAAAAKLAAKAAKAALKAAAAAAKL
>lcl|564|lcl|HAPPENN&HAPPENN-RR90&HAPPENN-hard|lcl|subdataset_12|lcl|hemolytic|lcl|cTer
LLRRLLRRLLRRLLRR
```

```
>lcl|3035|lcl|HAPPENN&HAPPENN-RR90&HAPPENN-hard|lcl|subdataset_7|lcl|hemolytic|lcl|cTer
GMWPKILGHLIR
>lcl|4744|lcl|HAPPENN|lcl|subdataset_6|lcl|non-hemolytic|lcl|cTer
IWKRWWWKR
>lcl|336|lcl|HAPPENN&HAPPENN-hard|lcl|subdataset_10|lcl|non-hemolytic|lcl|cTer
LFGVLAKVAAHVVPAIAEHF
>lcl|834|lcl|HAPPENN&HAPPENN-RR90&HAPPENN-hard|lcl|subdataset_6|lcl|non-hemolytic
FSEAIKKIIDFLGEGLFDIIKKIAESF
>lcl|622|lcl|HAPPENN|lcl|subdataset_6|lcl|hemolytic|lcl|cTer
FLPLIGRVLSGIL
>lcl|1624|lcl|HAPPENN&HAPPENN-RR90&HAPPENN-hard|lcl|subdataset_11|lcl|non-hemolytic|lcl|cTer
KFWKLLKKALRLWAKVL
>lcl|3249|lcl|HAPPENN&HAPPENN-RR90|lcl|subdataset_10|lcl|non-hemolytic
VKLIQIRIWIQYVTVLQMFSMKTKQ
>lcl|4468|lcl|HAPPENN|lcl|subdataset_12|lcl|hemolytic|lcl|cTer
GIMSSLMKKLAKIIAK
>lcl|2548|lcl|HAPPENN&HAPPENN-RR90|lcl|subdataset_12|lcl|non-hemolytic|lcl|cTer
GGLRSLGRKILRAWKKYGPQATPATRQ
>lcl|1341|lcl|HAPPENN|lcl|subdataset_5|lcl|hemolytic|lcl|cTer
KNWKAILKHIIK
>lcl|4723|lcl|HAPPENN&HAPPENN-RR90&HAPPENN-hard|lcl|subdataset_9|lcl|hemolytic|lcl|cTer
FPVTWRWWTWWKG
>lcl|3021|lcl|HAPPENN|lcl|subdataset_9|lcl|hemolytic|lcl|cTer
GMWSKILGHLIK
>lcl|937|lcl|HAPPENN&HAPPENN-RR90&HAPPENN-hard|lcl|subdataset_10|lcl|hemolytic|lcl|cTer
FLPWFSKFLGRIL
>lcl|1034|lcl|HAPPENN|lcl|subdataset_9|lcl|hemolytic
SVSNAATRVCRTGRSRWRDVCRNFMR
>lcl|1954|lcl|HAPPENN&HAPPENN-RR90|lcl|subdataset_11|lcl|non-hemolytic
KRFKKFFRKLKKSVKKRKKEFKKKPRVIGVSIPF
>lcl|1633|lcl|HAPPENN&HAPPENN-RR90|lcl|subdataset_9|lcl|non-hemolytic
FFGTKGIFSKVEPIFCKISHSC
>lcl|1836|lcl|HAPPENN&HAPPENN-RR90&HAPPENN-hard|lcl|subdataset_9|lcl|hemolytic
GIGSMLLGLAKNVGMSLLNKAQCKISGKC
>lcl|2866|lcl|HAPPENN|lcl|subdataset_6|lcl|non-hemolytic
KKLKKFKLLQ
>lcl|426|lcl|HAPPENN|lcl|subdataset_7|lcl|non-hemolytic|lcl|cTer
HFLLTLVNLAKKIK
>lcl|3852|lcl|HAPPENN&HAPPENN-RR90|lcl|subdataset_5|lcl|non-hemolytic|lcl|cTer
RKKKIKIIKRLI
>lcl|2677|lcl|HAPPENN&HAPPENN-RR90&HAPPENN-hard|lcl|subdataset_10|lcl|hemolytic
FLPLLAGVVANFLPQIICKIARKC
>lcl|3724|lcl|HAPPENN|lcl|subdataset_12|lcl|non-hemolytic|lcl|nTer|lcl|cTer
RPAFRKAAFRVMRACV
>lcl|4407|lcl|HAPPENN&HAPPENN-RR90&HAPPENN-hard|lcl|subdataset_10|lcl|hemolytic|lcl|cTer
KQIKIWFQNKKMKWKK
>lcl|4360|lcl|HAPPENN&HAPPENN-RR90&HAPPENN-hard|lcl|subdataset_4|lcl|non-hemolytic
FLGMLLHGVGHAIHGLIHGKQNVE
>lcl|1488|lcl|HAPPENN|lcl|subdataset_6|lcl|hemolytic
VCGETCVGGTCNTPGCSCSRPVCTRNGLP
>lcl|3782|lcl|HAPPENN&HAPPENN-RR90|lcl|subdataset_4|lcl|non-hemolytic|lcl|cTer
GIHKILKYGKPS
>lcl|1111|lcl|HAPPENN|lcl|subdataset_5|lcl|hemolytic|lcl|cTer
ILGKLLSTAWGLLSKL
>lcl|3511|lcl|HAPPENN&HAPPENN-RR90|lcl|subdataset_10|lcl|non-hemolytic
INLKAIAALARNY
>lcl|687|lcl|HAPPENN&HAPPENN-RR90&HAPPENN-hard|lcl|subdataset_12|lcl|hemolytic|lcl|cTer
FIGLLISAGKAIHDLIRRRH
>lcl|222|lcl|HAPPENN|lcl|subdataset_10|lcl|non-hemolytic|lcl|cTer
FRPKVTITIQGSARF
>lcl|3828|lcl|HAPPENN&HAPPENN-RR90|lcl|subdataset_11|lcl|non-hemolytic|lcl|cTer
KRRLAAFRAFRGALKSVLKK
>lcl|4680|lcl|HAPPENN|lcl|subdataset_5|lcl|non-hemolytic|lcl|cTer
IRIRIRIR
>lcl|237|lcl|HAPPENN|lcl|subdataset_10|lcl|hemolytic
GLFGKLIKKFLRKAISYAVKKARGKH
>lcl|4603|lcl|HAPPENN|lcl|subdataset_9|lcl|non-hemolytic|lcl|cTer
```

SGKLYYRRKK
>lcl|3570|lcl|HAPPENN|lcl|subdataset_8|lcl|hemolytic|lcl|cTer
GLLSLLSLLGKLL
>lcl|1797|lcl|HAPPENN|lcl|subdataset_7|lcl|hemolytic|lcl|cTer
WWWLRRRW
>lcl|3999|lcl|HAPPENN|lcl|subdataset_10|lcl|hemolytic|lcl|nTer|lcl|cTer
GFFALIPKIISSPLFKTLLSAVGSALSSSGGQE
>lcl|3194|lcl|HAPPENN&HAPPENN-RR90&HAPPENN-hard|lcl|subdataset_10|lcl|non-hemolytic|lcl|cTer
FIKELLPHLSGIIDSVANAIK
>lcl|2881|lcl|HAPPENN|lcl|subdataset_4|lcl|non-hemolytic
KLKKKFLLKQ
>lcl|48|lcl|HAPPENN&HAPPENN-RR90&HAPPENN-hard|lcl|subdataset_5|lcl|non-hemolytic
FLLFPLMCKIQGKC
>lcl|3116|lcl|HAPPENN&HAPPENN-hard|lcl|subdataset_8|lcl|non-hemolytic
FLPLFLPKIICVITKKC
>lcl|3190|lcl|HAPPENN|lcl|subdataset_4|lcl|hemolytic
FLGAIAAALPHVINAVTNAL
>lcl|3441|lcl|HAPPENN&HAPPENN-RR90|lcl|subdataset_7|lcl|non-hemolytic
YGAKAKAAKAAKAKAAKA
>lcl|1694|lcl|HAPPENN|lcl|subdataset_6|lcl|non-hemolytic|lcl|cTer
KKLFKKILKTL
>lcl|510|lcl|HAPPENN&HAPPENN-RR90&HAPPENN-hard|lcl|subdataset_4|lcl|non-hemolytic|lcl|cTer
GFKDLLKGAAKALKKTVLF
>lcl|3406|lcl|HAPPENN&HAPPENN-RR90&HAPPENN-hard|lcl|subdataset_9|lcl|non-hemolytic|lcl|cTer
HKLGTLVNLAKKIL
>lcl|3816|lcl|HAPPENN&HAPPENN-RR90|lcl|subdataset_9|lcl|non-hemolytic|lcl|cTer
ALAHFLKKAIKK
>lcl|3339|lcl|HAPPENN|lcl|subdataset_5|lcl|non-hemolytic|lcl|cTer
GKSDVRRWRSRY
>lcl|3398|lcl|HAPPENN&HAPPENN-RR90|lcl|subdataset_4|lcl|non-hemolytic
KWCRCVCRRGICRCRCRK
>lcl|1251|lcl|HAPPENN&HAPPENN-RR90&HAPPENN-hard|lcl|subdataset_9|lcl|hemolytic
KWKSFLRTLKSPAKTVFHTALKAISS
>lcl|4740|lcl|HAPPENN|lcl|subdataset_7|lcl|non-hemolytic|lcl|cTer
KWWRWRRWW
>lcl|2249|lcl|HAPPENN|lcl|subdataset_8|lcl|non-hemolytic|lcl|cTer
HKKHKKHKK
>lcl|4270|lcl|HAPPENN&HAPPENN-RR90|lcl|subdataset_11|lcl|non-hemolytic
GFCRCLCRRGVCRCICTR
>lcl|2025|lcl|HAPPENN&HAPPENN-RR90|lcl|subdataset_10|lcl|non-hemolytic|lcl|cTer
IARRALKKAKRAAHKIPAAKKFARR
>lcl|1303|lcl|HAPPENN|lcl|subdataset_10|lcl|hemolytic
GFFALIPKIISSPLFKTLLSAVGSALSSSGG
>lcl|3652|lcl|HAPPENN&HAPPENN-RR90&HAPPENN-hard|lcl|subdataset_7|lcl|non-hemolytic|lcl|cTer
ILPWKWPKWPWRR
>lcl|3495|lcl|HAPPENN|lcl|subdataset_12|lcl|hemolytic
ALWFTMLKKLGTMALHAGKAALGAAANTISQGTQ
>lcl|4135|lcl|HAPPENN|lcl|subdataset_7|lcl|non-hemolytic
RWCVYRYVRVRGVLVRYRRCW
>lcl|3625|lcl|HAPPENN|lcl|subdataset_4|lcl|non-hemolytic|lcl|cTer
GRAKRFRKKAKKLFKKLSPVIPLLHL
>lcl|4351|lcl|HAPPENN|lcl|subdataset_6|lcl|hemolytic|lcl|cTer
GLFDVIKKLLKKIGGL
>lcl|1092|lcl|HAPPENN&HAPPENN-RR90&HAPPENN-hard|lcl|subdataset_4|lcl|hemolytic|lcl|cTer
GFLGSLLKTGLKVGSNLL
>lcl|3739|lcl|HAPPENN|lcl|subdataset_5|lcl|hemolytic|lcl|nTer|lcl|cTer
RGLRRLGRKIAHGVKKYGPTVKRIKRKA
>lcl|3805|lcl|HAPPENN&HAPPENN-RR90&HAPPENN-hard|lcl|subdataset_11|lcl|hemolytic|lcl|cTer
FLPFIAGMAAKFLPKIFCAISKK
>lcl|1440|lcl|HAPPENN|lcl|subdataset_7|lcl|non-hemolytic
LVIRTVIAGYNLYRAIKKK
>lcl|3482|lcl|HAPPENN|lcl|subdataset_9|lcl|non-hemolytic
IKSIASKVANTVQKLKRKAKNAV
>lcl|1208|lcl|HAPPENN|lcl|subdataset_8|lcl|hemolytic|lcl|cTer
GFMKYIGPLIPHAVKAISDLI
>lcl|3273|lcl|HAPPENN&HAPPENN-RR90&HAPPENN-hard|lcl|subdataset_7|lcl|hemolytic
AIPCGESCVWIPCISTVIGCSCSNKVCYR

```
>lcl|3145|lcl|HAPPENN&HAPPENN-RR90|lcl|subdataset_8|lcl|non-hemolytic
VTPPWARIYYGCAKA
>lcl|3730|lcl|HAPPENN&HAPPENN-RR90&HAPPENN-hard|lcl|subdataset_9|lcl|hemolytic|lcl|nTer|lcl|cTer
GALRRLGRKITHAVKKYGPTVLRIIRIAG
>lcl|596|lcl|HAPPENN|lcl|subdataset_9|lcl|hemolytic
IKIPAVVKDTLKKVAKGVLSAVAGALTQ
>lcl|3892|lcl|HAPPENN&HAPPENN-RR90|lcl|subdataset_10|lcl|non-hemolytic|lcl|cTer
KRRKLAFRAFRFALKAVLKK
>lcl|200|lcl|HAPPENN|lcl|subdataset_11|lcl|hemolytic
FWGALAKGALKLIVGSLFSSFSKKD
>lcl|1207|lcl|HAPPENN&HAPPENN-RR90&HAPPENN-hard|lcl|subdataset_4|lcl|hemolytic|lcl|cTer
FIQYLAPLIPHAVKAISDLI
>lcl|1631|lcl|HAPPENN&HAPPENN-RR90&HAPPENN-hard|lcl|subdataset_4|lcl|non-hemolytic
GIMDTIKNAAKDVVQSLLNKASCKLAKTC
>lcl|4665|lcl|HAPPENN&HAPPENN-RR90|lcl|subdataset_11|lcl|non-hemolytic|lcl|cTer
IRVKIRVKIRVK
>lcl|2542|lcl|HAPPENN|lcl|subdataset_8|lcl|non-hemolytic|lcl|cTer
TPFKISIH
>lcl|1924|lcl|HAPPENN&HAPPENN-RR90|lcl|subdataset_6|lcl|non-hemolytic
CNPLNGADRRTDSFPRFTVI
>lcl|2745|lcl|HAPPENN|lcl|subdataset_6|lcl|non-hemolytic
INLKAIAALAKKLL
>lcl|4222|lcl|HAPPENN&HAPPENN-RR90&HAPPENN-hard|lcl|subdataset_6|lcl|non-hemolytic|lcl|cTer
KWKVFKKIEKKWKVFKKIEKAGPKWKVFKKIEK
>lcl|2324|lcl|HAPPENN&HAPPENN-RR90|lcl|subdataset_4|lcl|non-hemolytic
GFAWNVCVYRNGVRVCHRRAN
>lcl|694|lcl|HAPPENN|lcl|subdataset_8|lcl|non-hemolytic|lcl|cTer
KWKKLLKKPLKLKL
>lcl|1390|lcl|HAPPENN&HAPPENN-hard|lcl|subdataset_5|lcl|non-hemolytic
WNPFKELERAGQRIRDSIISAAP
>lcl|1750|lcl|HAPPENN&HAPPENN-RR90&HAPPENN-hard|lcl|subdataset_7|lcl|non-hemolytic
FFGWLIKGAIHAPKAIHPLIHRRRH
>lcl|4502|lcl|HAPPENN|lcl|subdataset_5|lcl|non-hemolytic
RIKRFWPVVIRTVVAGYNLYR
>lcl|3521|lcl|HAPPENN&HAPPENN-hard|lcl|subdataset_10|lcl|non-hemolytic
PKLKFLSKWIG
>lcl|1721|lcl|HAPPENN&HAPPENN-RR90|lcl|subdataset_5|lcl|non-hemolytic
RWCVYARVRGVRYRRCR
>lcl|2489|lcl|HAPPENN&HAPPENN-RR90|lcl|subdataset_8|lcl|non-hemolytic|lcl|cTer
WFKAAAKAAAKFW
>lcl|3|lcl|HAPPENN|lcl|subdataset_8|lcl|hemolytic
ALWDTLLKKVLKAAAKAALDAVLVGANA
>lcl|3326|lcl|HAPPENN|lcl|subdataset_10|lcl|non-hemolytic|lcl|cTer
FKDLKKIANIINSIFKK
>lcl|3105|lcl|HAPPENN|lcl|subdataset_8|lcl|non-hemolytic|lcl|cTer
RWKIFKKIEKVGQNIRDGIVKAGPAVAVVGQAATI
>lcl|3444|lcl|HAPPENN|lcl|subdataset_5|lcl|hemolytic|lcl|cTer
RRWCFRVCYKGRCRYKCR
>lcl|1571|lcl|HAPPENN|lcl|subdataset_10|lcl|hemolytic|lcl|cTer
ALWKTMLKKLGTMA
>lcl|1042|lcl|HAPPENN&HAPPENN-RR90&HAPPENN-hard|lcl|subdataset_8|lcl|non-hemolytic
DVNDLKNLCAKTHNLLPMCAMFGKK
>lcl|2422|lcl|HAPPENN&HAPPENN-RR90|lcl|subdataset_9|lcl|non-hemolytic
RTCIPIPPVCF
>lcl|2672|lcl|HAPPENN&HAPPENN-hard|lcl|subdataset_4|lcl|non-hemolytic
GVLDTFKDVAIGVAKGAGTGVLKALLCKLDKSC
>lcl|2533|lcl|HAPPENN&HAPPENN-RR90|lcl|subdataset_10|lcl|non-hemolytic
KLLRHRLLVTLA
>lcl|4154|lcl|HAPPENN&HAPPENN-hard|lcl|subdataset_8|lcl|non-hemolytic
KKLFKKILKYL
>lcl|2460|lcl|HAPPENN|lcl|subdataset_9|lcl|hemolytic|lcl|cTer
INWLAIAAKVAGML
>lcl|2704|lcl|HAPPENN&HAPPENN-RR90&HAPPENN-hard|lcl|subdataset_4|lcl|hemolytic|lcl|cTer
FLSHIAGFLSNLF
>lcl|573|lcl|HAPPENN|lcl|subdataset_12|lcl|non-hemolytic|lcl|cTer
LSPAVMASLA
>lcl|2983|lcl|HAPPENN|lcl|subdataset_10|lcl|hemolytic|lcl|cTer
GLLKVIKVLL
>lcl|1614|lcl|HAPPENN|lcl|subdataset_11|lcl|non-hemolytic|lcl|cTer
```

```
5018 RRHWRWWRR
5019 >lcl|4259|lcl|HAPPENN&HAPPENN-RR90|lcl|subdataset_9|lcl|non-hemolytic
5020 PPPVIKFNRPFLMWIVERDTRSILFMGKIVNPKAP
5021 >lcl|13|lcl|HAPPENN|lcl|subdataset_5|lcl|hemolytic
5022 AAAKAALNAVLVGANA
5023 >lcl|3572|lcl|HAPPENN&HAPPENN-RR90&HAPPENN-hard|lcl|subdataset_9|lcl|hemolytic|lcl|cTer
5024 FFGSVLKLIPKIL
5025 >lcl|1557|lcl|HAPPENN&HAPPENN-RR90|lcl|subdataset_8|lcl|non-hemolytic
5026 KCRQWQSKIRRTNPIFCIRR
5027 >lcl|3155|lcl|HAPPENN&HAPPENN-RR90|lcl|subdataset_10|lcl|non-hemolytic|lcl|cTer
5028 GMASKAGSIVGKIAKIALGAL
5029 >lcl|947|lcl|HAPPENN&HAPPENN-RR90&HAPPENN-hard|lcl|subdataset_9|lcl|non-hemolytic|lcl|cTer
5030 FLPLAGRVLSGIL
5031 >lcl|3574|lcl|HAPPENN&HAPPENN-RR90&HAPPENN-hard|lcl|subdataset_8|lcl|hemolytic|lcl|cTer
5032 LLGSLLKLLPKLL
5033 >lcl|4581|lcl|HAPPENN|lcl|subdataset_12|lcl|non-hemolytic
5034 VWRRWRRFW
5035 >lcl|2831|lcl|HAPPENN|lcl|subdataset_4|lcl|hemolytic|lcl|cTer
5036 YKLLKLLLPKLKPLLPKL
5037 >lcl|888|lcl|HAPPENN&HAPPENN-RR90|lcl|subdataset_9|lcl|non-hemolytic|lcl|cTer
5038 KFKSFIKKLTSKFLHSAKKF
5039 >lcl|313|lcl|HAPPENN&HAPPENN-RR90&HAPPENN-hard|lcl|subdataset_5|lcl|non-hemolytic|lcl|cTer
5040 FAKWAFKWLKK
5041 >lcl|2174|lcl|HAPPENN&HAPPENN-RR90&HAPPENN-hard|lcl|subdataset_4|lcl|non-hemolytic
5042 FLGAVLKVAGKLVPAAI
5043 >lcl|1462|lcl|HAPPENN&HAPPENN-RR90&HAPPENN-hard|lcl|subdataset_7|lcl|non-hemolytic
5044 FMPIIGRLMSGSL
5045 >lcl|226|lcl|HAPPENN&HAPPENN-hard|lcl|subdataset_9|lcl|non-hemolytic|lcl|cTer
5046 LLKWLKKL
5047 >lcl|4801|lcl|HAPPENN|lcl|subdataset_4|lcl|non-hemolytic
5048 VFRLKKWMQKVIDRFGG
5049 >lcl|3274|lcl|HAPPENN&HAPPENN-RR90&HAPPENN-hard|lcl|subdataset_12|lcl|hemolytic
5050 GEYCGESCYLIPCFTPGCYCVSRQCVNKN
5051 >lcl|4173|lcl|HAPPENN|lcl|subdataset_4|lcl|hemolytic
5052 KKLFKKILKYLTTGLPALISW
5053 >lcl|2286|lcl|HAPPENN|lcl|subdataset_10|lcl|hemolytic
5054 IKNAKVCVYAVCVSHK
5055 >lcl|568|lcl|HAPPENN&HAPPENN-RR90&HAPPENN-hard|lcl|subdataset_4|lcl|non-hemolytic
5056 INLKGLIKKVASLLT
5057 >lcl|1582|lcl|HAPPENN|lcl|subdataset_9|lcl|non-hemolytic|lcl|cTer
5058 KIAGKIAKKAGKIAK
5059 >lcl|1826|lcl|HAPPENN&HAPPENN-RR90&HAPPENN-hard|lcl|subdataset_10|lcl|hemolytic|lcl|cTer
5060 FLGGLIKWKWPWWPWRR
5061 >lcl|1864|lcl|HAPPENN&HAPPENN-hard|lcl|subdataset_9|lcl|non-hemolytic|lcl|cTer
5062 LKKLLKKLLK
5063 >lcl|2500|lcl|HAPPENN|lcl|subdataset_9|lcl|non-hemolytic
5064 VNFKLLSHSLLVTLASHL
5065 >lcl|3770|lcl|HAPPENN|lcl|subdataset_5|lcl|non-hemolytic|lcl|cTer
5066 RWWRWWR
5067 >lcl|1822|lcl|HAPPENN&HAPPENN-RR90|lcl|subdataset_8|lcl|non-hemolytic|lcl|cTer
5068 RRPWRWPRWPWRR
5069 >lcl|317|lcl|HAPPENN&HAPPENN-RR90|lcl|subdataset_6|lcl|non-hemolytic|lcl|cTer
5070 KWKKLLKKPLKLLKKLLK
5071 >lcl|3133|lcl|HAPPENN&HAPPENN-RR90&HAPPENN-hard|lcl|subdataset_9|lcl|hemolytic|lcl|cTer
5072 NFLDTLINLAKKFI
5073 >lcl|1304|lcl|HAPPENN|lcl|subdataset_7|lcl|hemolytic
5074 GFFALIPKIISSPLFKTLLSAV
5075 >lcl|4721|lcl|HAPPENN|lcl|subdataset_5|lcl|non-hemolytic|lcl|cTer
5076 FPVTWPTKWLKG
5077 >lcl|3372|lcl|HAPPENN&HAPPENN-RR90&HAPPENN-hard|lcl|subdataset_11|lcl|hemolytic|lcl|cTer
5078 ILAWKWPWWPWRR
5079 >lcl|2721|lcl|HAPPENN&HAPPENN-hard|lcl|subdataset_4|lcl|non-hemolytic
5080 GVIIDTLKGAAKTVAAELLRKAHCKLTNSC
5081 >lcl|1469|lcl|HAPPENN&HAPPENN-RR90&HAPPENN-hard|lcl|subdataset_6|lcl|hemolytic|lcl|cTer
5082 ILGTILGLLKSL
```

```
>lcl|203|lcl|HAPPENN&HAPPENN-RR90&HAPPENN-hard|lcl|subdataset_6|lcl|hemolytic|lcl|cTer
ILGIITSLLKSL
>lcl|3377|lcl|HAPPENN&HAPPENN-RR90&HAPPENN-hard|lcl|subdataset_5|lcl|hemolytic|lcl|cTer
ILPWKWPAWPWRR
>lcl|1331|lcl|HAPPENN&HAPPENN-RR90&HAPPENN-hard|lcl|subdataset_4|lcl|hemolytic|lcl|cTer
LKWGAILKHIIK
>lcl|1266|lcl|HAPPENN&HAPPENN-RR90&HAPPENN-hard|lcl|subdataset_6|lcl|hemolytic|lcl|cTer
IFGAIAGLLKNIF
>lcl|2897|lcl|HAPPENN|lcl|subdataset_7|lcl|non-hemolytic
KKKLLFLKKQ
>lcl|842|lcl|HAPPENN|lcl|subdataset_9|lcl|non-hemolytic|lcl|cTer
LLKKLLKWLKK
>lcl|2044|lcl|HAPPENN&HAPPENN-hard|lcl|subdataset_12|lcl|non-hemolytic|lcl|cTer
FLRFIGSVKHGKGHLVHHIGVAL
>lcl|2931|lcl|HAPPENN|lcl|subdataset_6|lcl|non-hemolytic
LKKKKFKKLQ
>lcl|326|lcl|HAPPENN|lcl|subdataset_9|lcl|hemolytic|lcl|cTer
ILGTILGLLKGL
>lcl|2987|lcl|HAPPENN|lcl|subdataset_4|lcl|hemolytic
GVWSTVLGGLKKFAKGGLEAIVNPK
>lcl|3024|lcl|HAPPENN&HAPPENN-RR90&HAPPENN-hard|lcl|subdataset_8|lcl|hemolytic|lcl|cTer
GMWSKILKHLIR
>lcl|4469|lcl|HAPPENN|lcl|subdataset_7|lcl|non-hemolytic|lcl|cTer
GIMSSLMKKLKKIIAK
>lcl|1221|lcl|HAPPENN|lcl|subdataset_8|lcl|hemolytic|lcl|cTer
LKLKSIVSWALLVL
>lcl|616|lcl|HAPPENN&HAPPENN-RR90&HAPPENN-hard|lcl|subdataset_12|lcl|non-hemolytic|lcl|cTer
GLVDVLGKVGGLIKKLLP
>lcl|3503|lcl|HAPPENN|lcl|subdataset_12|lcl|non-hemolytic|lcl|cTer
RLYRRLYR
>lcl|3619|lcl|HAPPENN|lcl|subdataset_9|lcl|hemolytic|lcl|cTer
RLWLAWKRR
>lcl|2145|lcl|HAPPENN&HAPPENN-RR90|lcl|subdataset_9|lcl|non-hemolytic
GFALAGLARILCLWFREFSGFFRRLNRRFAMRRR
>lcl|1071|lcl|HAPPENN&HAPPENN-RR90|lcl|subdataset_10|lcl|non-hemolytic|lcl|cTer
GRLKRLRKKLKKLLKKLS
>lcl|2266|lcl|HAPPENN|lcl|subdataset_10|lcl|non-hemolytic
YYHFWHRGVTKR
>lcl|756|lcl|HAPPENN&HAPPENN-hard|lcl|subdataset_5|lcl|non-hemolytic
RLYRKVYGRLYRKVYGRLYRKVYGRLYRKVYGKKK
>lcl|416|lcl|HAPPENN&HAPPENN-hard|lcl|subdataset_5|lcl|non-hemolytic|lcl|cTer
LKLGTLVNLAKKIL
>lcl|2057|lcl|HAPPENN|lcl|subdataset_5|lcl|non-hemolytic|lcl|cTer
YKAWRWAWRWK
>lcl|4698|lcl|HAPPENN&HAPPENN-RR90&HAPPENN-hard|lcl|subdataset_4|lcl|non-hemolytic
KRMGIFHLFWAGLRKLGNLIKNKIQQGIENFLG
>lcl|2162|lcl|HAPPENN&HAPPENN-RR90&HAPPENN-hard|lcl|subdataset_9|lcl|hemolytic|lcl|cTer
GILSSFKGVAKGVAKDLAGKLLETLK
>lcl|1667|lcl|HAPPENN|lcl|subdataset_12|lcl|non-hemolytic|lcl|cTer
KKPKLPWKPKK
>lcl|2308|lcl|HAPPENN&HAPPENN-RR90|lcl|subdataset_7|lcl|non-hemolytic
ACFLTRLGTYVC
>lcl|2231|lcl|HAPPENN&HAPPENN-RR90|lcl|subdataset_4|lcl|non-hemolytic
HGVSGHGQHGTHG
>lcl|4813|lcl|HAPPENN|lcl|subdataset_9|lcl|hemolytic|lcl|cTer
FLSTIWNGIKGLL
>lcl|4804|lcl|HAPPENN&HAPPENN-hard|lcl|subdataset_6|lcl|non-hemolytic
YGFYTHVFRLKKWMQKVIDRFGG
>lcl|1100|lcl|HAPPENN&HAPPENN-RR90|lcl|subdataset_9|lcl|non-hemolytic|lcl|cTer
LFKEILEKIKAKL
>lcl|4333|lcl|HAPPENN&HAPPENN-RR90|lcl|subdataset_4|lcl|non-hemolytic|lcl|cTer
GIHDILAYGKPS
>lcl|2334|lcl|HAPPENN&HAPPENN-RR90|lcl|subdataset_6|lcl|non-hemolytic|lcl|nTer|lcl|cTer
KYTGKCKTKKNKCK
>lcl|2001|lcl|HAPPENN|lcl|subdataset_12|lcl|hemolytic|lcl|cTer
GIKDLLKGAAKALVKTVLF
```

```
>lcl|1085|lcl|HAPPENN&HAPPENN-RR90&HAPPENN-hard|lcl|subdataset_6|lcl|hemolytic|lcl|cTer
GMATKAGTALGKVAKAVIGAAL
>lcl|1746|lcl|HAPPENN|lcl|subdataset_7|lcl|non-hemolytic|lcl|cTer
FWRIRIRR
>lcl|4404|lcl|HAPPENN&HAPPENN-RR90|lcl|subdataset_6|lcl|non-hemolytic
HTASDAAAAAALTAANAAAAAAASMA
>lcl|600|lcl|HAPPENN|lcl|subdataset_4|lcl|hemolytic
GIWKSLFTKLLKG
>lcl|819|lcl|HAPPENN|lcl|subdataset_10|lcl|non-hemolytic
FKDLKKIANIINSIFKK
>lcl|76|lcl|HAPPENN&HAPPENN-RR90|lcl|subdataset_5|lcl|non-hemolytic|lcl|cTer
KISKKIMRTFLRRILTGKK
>lcl|2590|lcl|HAPPENN&HAPPENN-hard|lcl|subdataset_4|lcl|non-hemolytic|lcl|cTer
RIWVIWRR
>lcl|2457|lcl|HAPPENN&HAPPENN-RR90|lcl|subdataset_12|lcl|non-hemolytic|lcl|nTer|lcl|cTer
RRRWRKRRWWW
>lcl|1072|lcl|HAPPENN&HAPPENN-RR90|lcl|subdataset_5|lcl|non-hemolytic|lcl|cTer
GRIKRIRKKIKKLIKKLS
>lcl|3242|lcl|HAPPENN&HAPPENN-RR90&HAPPENN-hard|lcl|subdataset_6|lcl|hemolytic
GFMDTAKNVAKNVAVTLIDKLRCKVTGGC
>lcl|2175|lcl|HAPPENN&HAPPENN-RR90&HAPPENN-hard|lcl|subdataset_8|lcl|non-hemolytic
FLGAVLKVCKISKKCAGKLVPAAI
>lcl|1955|lcl|HAPPENN|lcl|subdataset_6|lcl|non-hemolytic
KRWRWRW
>lcl|1673|lcl|HAPPENN&HAPPENN-RR90&HAPPENN-hard|lcl|subdataset_5|lcl|hemolytic|lcl|cTer
FRRFFKWFRRFFKFF
>lcl|2136|lcl|HAPPENN|lcl|subdataset_11|lcl|hemolytic|lcl|cTer
ALWKSLLKNVGKAAGKAALNAVTDMVNQ
>lcl|2591|lcl|HAPPENN&HAPPENN-hard|lcl|subdataset_9|lcl|non-hemolytic|lcl|cTer
INWKGIAAMAKKLL
>lcl|671|lcl|HAPPENN|lcl|subdataset_4|lcl|non-hemolytic|lcl|cTer
KKAFAAAAAFAAWAAFAKKKK
>lcl|325|lcl|HAPPENN&HAPPENN-RR90&HAPPENN-hard|lcl|subdataset_12|lcl|hemolytic|lcl|cTer
ILNWLKLGKAIIDAIL
>lcl|2016|lcl|HAPPENN&HAPPENN-RR90|lcl|subdataset_12|lcl|non-hemolytic|lcl|cTer
GSLHGFMYRYLKNMVLNLF
>lcl|982|lcl|HAPPENN&HAPPENN-RR90&HAPPENN-hard|lcl|subdataset_9|lcl|hemolytic|lcl|cTer
GWRKWIKKATHVGKHIGKAALDAYI
>lcl|3307|lcl|HAPPENN&HAPPENN-RR90&HAPPENN-hard|lcl|subdataset_10|lcl|hemolytic
FLPILASLAAKFGPKLFSLVTKKS
>lcl|556|lcl|HAPPENN&HAPPENN-hard|lcl|subdataset_8|lcl|non-hemolytic|lcl|cTer
FFRRFFRR
>lcl|656|lcl|HAPPENN&HAPPENN-RR90&HAPPENN-hard|lcl|subdataset_8|lcl|hemolytic|lcl|cTer
KKKLLLLLLLLLKKK
>lcl|4160|lcl|HAPPENN|lcl|subdataset_10|lcl|hemolytic
SKKLFKKILKYLAGPAKKLFKKILKYL
>lcl|1289|lcl|HAPPENN|lcl|subdataset_8|lcl|non-hemolytic
GLLRASSVWGRKYYVDLAGCAKA
>lcl|4350|lcl|HAPPENN&HAPPENN-RR90&HAPPENN-hard|lcl|subdataset_8|lcl|hemolytic|lcl|cTer
GLFDVIKKVLKKIGGL
>lcl|4119|lcl|HAPPENN&HAPPENN-hard|lcl|subdataset_10|lcl|non-hemolytic
SVKKFWGGVKAIFKGARKGLK
>lcl|468|lcl|HAPPENN&HAPPENN-RR90|lcl|subdataset_12|lcl|non-hemolytic|lcl|nTer|lcl|cTer
NRLARHFRDIAGRVNQRL
>lcl|4441|lcl|HAPPENN&HAPPENN-RR90|lcl|subdataset_5|lcl|non-hemolytic|lcl|cTer
AKRHHGLNCAKFH
>lcl|479|lcl|HAPPENN&HAPPENN-RR90|lcl|subdataset_6|lcl|non-hemolytic|lcl|cTer
FQWQRNIRKVR
>lcl|2430|lcl|HAPPENN|lcl|subdataset_9|lcl|non-hemolytic
HLGHHALDHLLK
>lcl|3986|lcl|HAPPENN|lcl|subdataset_11|lcl|non-hemolytic|lcl|cTer
GVVDIAKGAAKDIAGHLASKVMNKL
>lcl|4552|lcl|HAPPENN&HAPPENN-RR90&HAPPENN-hard|lcl|subdataset_6|lcl|hemolytic|lcl|cTer
WKLFKKILKWL
>lcl|3465|lcl|HAPPENN&HAPPENN-RR90&HAPPENN-hard|lcl|subdataset_6|lcl|hemolytic|lcl|cTer
```

```
KRFKKFFMKLKKSVKKRVMKFFKKPMVIGVTFPF
>lcl|3727|lcl|HAPPENN&HAPPENN-RR90&HAPPENN-hard|lcl|subdataset_8|lcl|non-hemolytic|lcl|nTer|lcl|cTer
WHWTWLRIRKKLR
>lcl|2172|lcl|HAPPENN&HAPPENN-RR90&HAPPENN-hard|lcl|subdataset_5|lcl|non-hemolytic|lcl|cTer
GFKRLVQRLKDFLRNLV
>lcl|3198|lcl|HAPPENN|lcl|subdataset_11|lcl|hemolytic
GIFGKILGVGKKVLCGLSGMC
>lcl|17|lcl|HAPPENN|lcl|subdataset_10|lcl|hemolytic|lcl|cTer
ALWKTLLKKVLKA
>lcl|1348|lcl|HAPPENN|lcl|subdataset_5|lcl|hemolytic
LDVKKIICVACKIKPNPACKKICPK
>lcl|3285|lcl|HAPPENN&HAPPENN-RR90&HAPPENN-hard|lcl|subdataset_7|lcl|hemolytic|lcl|cTer
ILGKIWEGIKSIF
>lcl|3943|lcl|HAPPENN&HAPPENN-RR90|lcl|subdataset_7|lcl|non-hemolytic
HPLKQYWWRPSI
>lcl|764|lcl|HAPPENN&HAPPENN-hard|lcl|subdataset_6|lcl|non-hemolytic
GIGKFLHSAGKFGKAFVGEIMKS
>lcl|120|lcl|HAPPENN|lcl|subdataset_6|lcl|hemolytic
LCYCRRRFCVCV
>lcl|414|lcl|HAPPENN|lcl|subdataset_12|lcl|hemolytic|lcl|cTer
HFLGTLVNLAKKIL
>lcl|4508|lcl|HAPPENN&HAPPENN-RR90&HAPPENN-hard|lcl|subdataset_10|lcl|non-hemolytic|lcl|cTer
AKKVFKRLEKLFSKIFNFK
>lcl|617|lcl|HAPPENN&HAPPENN-RR90&HAPPENN-hard|lcl|subdataset_11|lcl|non-hemolytic|lcl|cTer
LLKELWTKIKGAGKAVLGKIKGLL
>lcl|2046|lcl|HAPPENN&HAPPENN-RR90&HAPPENN-hard|lcl|subdataset_5|lcl|non-hemolytic|lcl|cTer
RLKLLLLLRLK
>lcl|3789|lcl|HAPPENN|lcl|subdataset_5|lcl|non-hemolytic|lcl|nTer|lcl|cTer
WGIRRILKYGKRS
>lcl|4648|lcl|HAPPENN|lcl|subdataset_5|lcl|hemolytic|lcl|cTer
IFGAIWPLALGALKNLIK
>lcl|4833|lcl|HAPPENN|lcl|subdataset_8|lcl|non-hemolytic
KIKKGFKKIFKRLPPIGVGVSIPLAGKR
>lcl|1148|lcl|HAPPENN&HAPPENN-RR90|lcl|subdataset_9|lcl|non-hemolytic
PKDLKETLQEKKP
>lcl|3675|lcl|HAPPENN&HAPPENN-RR90|lcl|subdataset_7|lcl|non-hemolytic
RRRSVGEEDAIPSHIEVNKFFLRKPAKEHI
>lcl|4393|lcl|HAPPENN&HAPPENN-hard|lcl|subdataset_11|lcl|non-hemolytic
LLSNVAGLLKQFAK
>lcl|1558|lcl|HAPPENN&HAPPENN-RR90&HAPPENN-hard|lcl|subdataset_8|lcl|non-hemolytic
KARQWQSKIRRTNPIFAIRR
>lcl|1569|lcl|HAPPENN|lcl|subdataset_10|lcl|hemolytic|lcl|cTer
ALWKTMLKKLGTMALHAGK
>lcl|2036|lcl|HAPPENN&HAPPENN-RR90|lcl|subdataset_5|lcl|non-hemolytic
ASEELLLRFQATSSGPILREEFE
>lcl|895|lcl|HAPPENN|lcl|subdataset_12|lcl|non-hemolytic|lcl|cTer
KWKSFIKKLTKKFLHSAKKF
>lcl|2779|lcl|HAPPENN&HAPPENN-RR90&HAPPENN-hard|lcl|subdataset_6|lcl|non-hemolytic
FVGALFKALSKLL
>lcl|3298|lcl|HAPPENN&HAPPENN-hard|lcl|subdataset_11|lcl|non-hemolytic
KGLGKDLAKLGVDLVACKISKQC
>lcl|496|lcl|HAPPENN|lcl|subdataset_7|lcl|non-hemolytic
AKKVFKRLEKLFSKIQNWK
>lcl|443|lcl|HAPPENN|lcl|subdataset_5|lcl|hemolytic|lcl|cTer
FKLFKKILKFL
>lcl|3825|lcl|HAPPENN&HAPPENN-RR90&HAPPENN-hard|lcl|subdataset_5|lcl|hemolytic|lcl|cTer
KRRLIARILRLAARALVKKR
>lcl|3367|lcl|HAPPENN|lcl|subdataset_8|lcl|hemolytic|lcl|cTer
PWKWPWWPWRR
>lcl|3469|lcl|HAPPENN&HAPPENN-RR90&HAPPENN-hard|lcl|subdataset_11|lcl|hemolytic|lcl|cTer
GVLSVIKNALPGIMRFIA
>lcl|3531|lcl|HAPPENN&HAPPENN-RR90&HAPPENN-hard|lcl|subdataset_4|lcl|hemolytic|lcl|cTer
FFPFLLGALGSLLPKIF
>lcl|794|lcl|HAPPENN|lcl|subdataset_8|lcl|non-hemolytic
```

```
GEKLKKIGQKIKKFFQKL
>lcl|3334|lcl|HAPPENN|lcl|subdataset_7|lcl|hemolytic|lcl|cTer
LRRLWLRANRLLRRLWLRANRL
>lcl|3874|lcl|HAPPENN&HAPPENN-RR90&HAPPENN-hard|lcl|subdataset_9|lcl|hemolytic|lcl|cTer
LRILRRLLRRLF
>lcl|1990|lcl|HAPPENN&HAPPENN-RR90&HAPPENN-hard|lcl|subdataset_8|lcl|hemolytic|lcl|cTer
GFLLKLKLGAKLFASAFVNSIK
>lcl|1083|lcl|HAPPENN&HAPPENN-RR90|lcl|subdataset_7|lcl|non-hemolytic
CVDIHVWDGVC
>lcl|1755|lcl|HAPPENN|lcl|subdataset_7|lcl|non-hemolytic|lcl|cTer
PFWRIRIRR
>lcl|2281|lcl|HAPPENN&HAPPENN-RR90&HAPPENN-hard|lcl|subdataset_7|lcl|hemolytic
RRGCFRVCYRGFCFQRCR
>lcl|1949|lcl|HAPPENN|lcl|subdataset_7|lcl|hemolytic
FLPKLLAGLPSFLCLVFKKC
>lcl|2706|lcl|HAPPENN|lcl|subdataset_9|lcl|non-hemolytic|lcl|cTer
KVVFKVKFKKK
>lcl|257|lcl|HAPPENN&HAPPENN-hard|lcl|subdataset_7|lcl|non-hemolytic|lcl|cTer
ILPWKWPWLPWRR
>lcl|552|lcl|HAPPENN|lcl|subdataset_7|lcl|non-hemolytic|lcl|cTer
DSHAKRHHGYKIKFHENHHSHRGY
>lcl|482|lcl|HAPPENN|lcl|subdataset_5|lcl|non-hemolytic
STVLTSKYR
>lcl|581|lcl|HAPPENN&HAPPENN-RR90|lcl|subdataset_5|lcl|non-hemolytic
QPTRRPRPGTGPGRRPRPRPRP
>lcl|1885|lcl|HAPPENN&HAPPENN-hard|lcl|subdataset_6|lcl|non-hemolytic|lcl|cTer
RLSRIVVIRVCR
>lcl|4537|lcl|HAPPENN|lcl|subdataset_5|lcl|hemolytic|lcl|cTer
FFHHVFRGIVHVGKTIHRLVTG
>lcl|172|lcl|HAPPENN|lcl|subdataset_8|lcl|non-hemolytic|lcl|cTer
AAGMGFFGAR
>lcl|2769|lcl|HAPPENN&HAPPENN-hard|lcl|subdataset_9|lcl|non-hemolytic|lcl|cTer
GLPALISWIKRKR
>lcl|2030|lcl|HAPPENN|lcl|subdataset_5|lcl|non-hemolytic|lcl|nTer|lcl|cTer
AAAAAAAAAK
>lcl|991|lcl|HAPPENN|lcl|subdataset_11|lcl|non-hemolytic
SWLRDLWDWICELLSDFK
>lcl|4239|lcl|HAPPENN|lcl|subdataset_11|lcl|hemolytic
SKHWLWLW
>lcl|3588|lcl|HAPPENN&HAPPENN-RR90&HAPPENN-hard|lcl|subdataset_11|lcl|non-hemolytic|lcl|cTer
WPKWWKWKRRWGRKKAKKRRG
>lcl|3059|lcl|HAPPENN&HAPPENN-RR90&HAPPENN-hard|lcl|subdataset_8|lcl|hemolytic|lcl|cTer
ILPILSLIGGLL
>lcl|3268|lcl|HAPPENN|lcl|subdataset_12|lcl|hemolytic
GIPCGESCVFIPCITAAIGCSCKSKVCYRN
>lcl|1647|lcl|HAPPENN&HAPPENN-RR90&HAPPENN-hard|lcl|subdataset_8|lcl|non-hemolytic
CVVSSGWKWNYKIRCKLTGNC
>lcl|4417|lcl|HAPPENN|lcl|subdataset_10|lcl|hemolytic|lcl|cTer
WRRRRRRRR
>lcl|1735|lcl|HAPPENN|lcl|subdataset_5|lcl|hemolytic|lcl|cTer
GIGGALLSAGKSALKGLAKGLAEHFAN
>lcl|3466|lcl|HAPPENN|lcl|subdataset_4|lcl|non-hemolytic|lcl|cTer
KRFKKFFKKLKNSVKKRAKKFFKKPRVIGVSIPF
>lcl|1352|lcl|HAPPENN|lcl|subdataset_5|lcl|non-hemolytic|lcl|cTer
KAYSTPRCKYLFRAVLCWL
>lcl|4503|lcl|HAPPENN|lcl|subdataset_4|lcl|non-hemolytic
RIKRFWPVVIRTVVAGYNLY
>lcl|1051|lcl|HAPPENN&HAPPENN-RR90|lcl|subdataset_10|lcl|non-hemolytic
FSPQMLQDIIEKKTKIL
>lcl|1030|lcl|HAPPENN&HAPPENN-RR90&HAPPENN-hard|lcl|subdataset_4|lcl|non-hemolytic
ENMFNIKSSVESDSFWG
>lcl|4363|lcl|HAPPENN&HAPPENN-RR90&HAPPENN-hard|lcl|subdataset_8|lcl|hemolytic
AIPWIWIWRLLRKG
>lcl|566|lcl|HAPPENN&HAPPENN-RR90&HAPPENN-hard|lcl|subdataset_6|lcl|non-hemolytic|lcl|cTer
KDLHTVVSAILQAL
>lcl|3981|lcl|HAPPENN|lcl|subdataset_7|lcl|non-hemolytic|lcl|cTer
GVVDILKGAAADIAGHLASKVMNKL
>lcl|4798|lcl|HAPPENN|lcl|subdataset_6|lcl|non-hemolytic
```

```
WMQKVIDRFGG
>lcl|1973|lcl|HAPPENN&HAPPENN-RR90&HAPPENN-hard|lcl|subdataset_8|lcl|non-hemolytic|lcl|cTer
GKEFKRIVWLSKTAKKL
>lcl|474|lcl|HAPPENN|lcl|subdataset_6|lcl|non-hemolytic
QKKIRVRLSA
>lcl|2973|lcl|HAPPENN|lcl|subdataset_4|lcl|hemolytic|lcl|cTer
GLLKWIKTLL
>lcl|4002|lcl|HAPPENN|lcl|subdataset_12|lcl|non-hemolytic
ILGPVLGLVSDTLDDVLGIL
>lcl|4|lcl|HAPPENN|lcl|subdataset_5|lcl|hemolytic
ALWMTLLKKVLKAAAKAALKAVLVGANA
>lcl|4344|lcl|HAPPENN|lcl|subdataset_4|lcl|non-hemolytic|lcl|cTer
GSKKPVPIIYCNRRTGKCQRM
>lcl|4418|lcl|HAPPENN&HAPPENN-RR90|lcl|subdataset_7|lcl|non-hemolytic
LAAKLTKAATKLTAALTKLAAALT
>lcl|4734|lcl|HAPPENN&HAPPENN-RR90&HAPPENN-hard|lcl|subdataset_11|lcl|non-hemolytic|lcl|cTer
KIAKVALKALKIAKGALKAL
>lcl|2444|lcl|HAPPENN|lcl|subdataset_10|lcl|non-hemolytic
HLNKRVQRELIGWLRWLK
>lcl|871|lcl|HAPPENN|lcl|subdataset_12|lcl|hemolytic
FIGAIARLLSKIF
>lcl|1463|lcl|HAPPENN&HAPPENN-RR90&HAPPENN-hard|lcl|subdataset_4|lcl|non-hemolytic
INMKASAAVAKKLL
>lcl|4100|lcl|HAPPENN&HAPPENN-RR90|lcl|subdataset_9|lcl|non-hemolytic
GLARALTRLLRQLTRQLTRA
>lcl|3716|lcl|HAPPENN|lcl|subdataset_12|lcl|non-hemolytic|lcl|nTer|lcl|cTer
KWKKLLKKPLLKKLLKKL
>lcl|2404|lcl|HAPPENN&HAPPENN-RR90&HAPPENN-hard|lcl|subdataset_8|lcl|non-hemolytic
KIFDAEILLNGKRKGLG
>lcl|913|lcl|HAPPENN|lcl|subdataset_12|lcl|hemolytic|lcl|cTer
VNSKKISGKSIKVSK
>lcl|2786|lcl|HAPPENN&HAPPENN-RR90&HAPPENN-hard|lcl|subdataset_12|lcl|non-hemolytic
FLGALFKVVSKLL
>lcl|2185|lcl|HAPPENN&HAPPENN-RR90&HAPPENN-hard|lcl|subdataset_4|lcl|non-hemolytic|lcl|cTer
ALNALKKVSQGIHEAIKLIANHVQ
>lcl|4398|lcl|HAPPENN&HAPPENN-RR90&HAPPENN-hard|lcl|subdataset_8|lcl|hemolytic|lcl|cTer
VKRWKKFWRKWKKWW
>lcl|94|lcl|HAPPENN|lcl|subdataset_8|lcl|non-hemolytic|lcl|cTer
KKKKKKAAFALWAAFAA
>lcl|4532|lcl|HAPPENN&HAPPENN-RR90|lcl|subdataset_4|lcl|non-hemolytic|lcl|cTer
ARRARKWARRALKLA
>lcl|2059|lcl|HAPPENN&HAPPENN-RR90&HAPPENN-hard|lcl|subdataset_5|lcl|hemolytic|lcl|cTer
YRMWRWAWRWR
>lcl|3834|lcl|HAPPENN&HAPPENN-hard|lcl|subdataset_8|lcl|non-hemolytic
GLKALKKVFQGIHEAIKLINNHVQ
>lcl|3152|lcl|HAPPENN&HAPPENN-RR90|lcl|subdataset_8|lcl|non-hemolytic
CVDIGFSPTGKRPPFCPYPG
>lcl|520|lcl|HAPPENN|lcl|subdataset_10|lcl|hemolytic|lcl|cTer
GLLGKLLKIAAKVGKKLL
>lcl|1252|lcl|HAPPENN&HAPPENN-RR90&HAPPENN-hard|lcl|subdataset_8|lcl|hemolytic
KWKSFLKKLTSAAKKVLTTALKPISS
>lcl|898|lcl|HAPPENN|lcl|subdataset_7|lcl|non-hemolytic|lcl|cTer
KWKSFIKKLTSKFLHSADKF
>lcl|385|lcl|HAPPENN|lcl|subdataset_8|lcl|hemolytic
CIGAVLKVLTTGLPALISWIKRKRQQC
>lcl|4042|lcl|HAPPENN|lcl|subdataset_8|lcl|non-hemolytic|lcl|cTer
KLKKKLKKLLKK
>lcl|4174|lcl|HAPPENN&HAPPENN-hard|lcl|subdataset_7|lcl|non-hemolytic
KKLFKKILKYLAGPATTGLPALISW
>lcl|1133|lcl|HAPPENN&HAPPENN-RR90&HAPPENN-hard|lcl|subdataset_12|lcl|hemolytic|lcl|cTer
GFGTILKALAKIAGKVVKKLATKPGATYMLKENLK
>lcl|1796|lcl|HAPPENN|lcl|subdataset_5|lcl|hemolytic|lcl|cTer
WWWRRRIW
>lcl|2965|lcl|HAPPENN|lcl|subdataset_11|lcl|non-hemolytic|lcl|cTer
LLKRIKTLL
>lcl|3271|lcl|HAPPENN|lcl|subdataset_9|lcl|hemolytic
GASCGETCFTGICFTAGCSCNPWPTCTRN
```

```
>lcl|3509|lcl|HAPPENN&HAPPENN-RR90&HAPPENN-hard|lcl|subdataset_10|lcl|non-hemolytic
VIVKAIATLASKLL
>lcl|2152|lcl|HAPPENN|lcl|subdataset_6|lcl|non-hemolytic|lcl|cTer
FLKLAKKLL
>lcl|4415|lcl|HAPPENN&HAPPENN-RR90&HAPPENN-hard|lcl|subdataset_4|lcl|non-hemolytic
AGLQFPVGRIGRLLRK
>lcl|2020|lcl|HAPPENN&HAPPENN-RR90&HAPPENN-hard|lcl|subdataset_10|lcl|hemolytic|lcl|cTer
GFKLGRKLVKVFKWII
>lcl|4837|lcl|HAPPENN|lcl|subdataset_6|lcl|hemolytic|lcl|cTer
FFHHIFRGIVHVGKKIHRLVTG
>lcl|2169|lcl|HAPPENN|lcl|subdataset_5|lcl|hemolytic|lcl|cTer
KWKVFKKIEKMIRNIRNKIAK
>lcl|1170|lcl|HAPPENN&HAPPENN-RR90&HAPPENN-hard|lcl|subdataset_4|lcl|non-hemolytic
CSCKNKVCYRNGIPCGESCVWIPCISAALG
>lcl|3241|lcl|HAPPENN&HAPPENN-RR90&HAPPENN-hard|lcl|subdataset_11|lcl|hemolytic
DSMGAVKLAKLLIDKMKCEVTKAC
>lcl|4089|lcl|HAPPENN&HAPPENN-RR90|lcl|subdataset_7|lcl|non-hemolytic
GLRRALLRLLRSLRRLLLRA
>lcl|4646|lcl|HAPPENN&HAPPENN-RR90|lcl|subdataset_10|lcl|non-hemolytic
LKKLLKLCKKLCKLAG
>lcl|1728|lcl|HAPPENN|lcl|subdataset_12|lcl|non-hemolytic|lcl|nTer|lcl|cTer
KWKSFLKTFKSAKKTKLHTALKAISS
>lcl|2193|lcl|HAPPENN|lcl|subdataset_9|lcl|non-hemolytic
KWKLFKKIGKVLKKL
>lcl|3286|lcl|HAPPENN&HAPPENN-RR90&HAPPENN-hard|lcl|subdataset_9|lcl|hemolytic|lcl|cTer
ILSYLWNGIKSIF
>lcl|2584|lcl|HAPPENN&HAPPENN-RR90&HAPPENN-hard|lcl|subdataset_10|lcl|non-hemolytic
GFSPNLPGKGLRIS
>lcl|458|lcl|HAPPENN&HAPPENN-RR90|lcl|subdataset_10|lcl|non-hemolytic
KRLFRRWQWRMKKY
>lcl|1799|lcl|HAPPENN|lcl|subdataset_4|lcl|non-hemolytic|lcl|cTer
RRLRKKTRKRLKKIGKVLKWI
>lcl|4562|lcl|HAPPENN&HAPPENN-RR90&HAPPENN-hard|lcl|subdataset_6|lcl|non-hemolytic
KKVWFIFHVCPKLKQRIL
>lcl|3786|lcl|HAPPENN&HAPPENN-RR90|lcl|subdataset_8|lcl|non-hemolytic|lcl|cTer
GIKKILKYGKKS
>lcl|2005|lcl|HAPPENN&HAPPENN-hard|lcl|subdataset_11|lcl|non-hemolytic|lcl|cTer
GKLGPLLKIAAKVGSKLL
>lcl|2187|lcl|HAPPENN&HAPPENN-hard|lcl|subdataset_10|lcl|non-hemolytic|lcl|cTer
LNALKKVFQKIHEAIKLI
>lcl|1716|lcl|HAPPENN|lcl|subdataset_6|lcl|non-hemolytic
RWCVYARVRGVRYRRCW
>lcl|2910|lcl|HAPPENN|lcl|subdataset_9|lcl|non-hemolytic
LKLKKFLKKQ
>lcl|3032|lcl|HAPPENN&HAPPENN-RR90&HAPPENN-hard|lcl|subdataset_8|lcl|hemolytic|lcl|cTer
GMWSKILGPLIR
>lcl|1332|lcl|HAPPENN&HAPPENN-RR90&HAPPENN-hard|lcl|subdataset_11|lcl|hemolytic|lcl|cTer
LNKGAILKHIIK
>lcl|827|lcl|HAPPENN|lcl|subdataset_6|lcl|non-hemolytic
WWSYVRRWRSR
>lcl|1907|lcl|HAPPENN&HAPPENN-RR90|lcl|subdataset_4|lcl|non-hemolytic
RLVRILVSKRPVAIKPYFRL
>lcl|653|lcl|HAPPENN&HAPPENN-hard|lcl|subdataset_10|lcl|non-hemolytic
IFGAIWNGIKS
>lcl|2370|lcl|HAPPENN|lcl|subdataset_6|lcl|non-hemolytic|lcl|cTer
RFRFFFRFR
>lcl|1866|lcl|HAPPENN|lcl|subdataset_4|lcl|non-hemolytic|lcl|cTer
IKKIIKKI
>lcl|569|lcl|HAPPENN&HAPPENN-RR90&HAPPENN-hard|lcl|subdataset_7|lcl|non-hemolytic|lcl|cTer
FDLLGLVKKVASAL
>lcl|2962|lcl|HAPPENN|lcl|subdataset_10|lcl|non-hemolytic|lcl|cTer
GLLKRIKTL
>lcl|2401|lcl|HAPPENN&HAPPENN-RR90&HAPPENN-hard|lcl|subdataset_9|lcl|non-hemolytic|lcl|cTer
RRLIRLWLRLLR
>lcl|4522|lcl|HAPPENN&HAPPENN-hard|lcl|subdataset_9|lcl|non-hemolytic
VIRTVVAGYNLYRAIKKK
>lcl|1022|lcl|HAPPENN|lcl|subdataset_5|lcl|hemolytic
```

```
GLPVCGETCVGGTCNTPGCKCSWPVCTRN
>lcl|4392|lcl|HAPPENN&HAPPENN-RR90&HAPPENN-hard|lcl|subdataset_9|lcl|non-hemolytic
AVAGEKLWLLPHLLKMLLTPTP
>lcl|2166|lcl|HAPPENN&HAPPENN-hard|lcl|subdataset_8|lcl|non-hemolytic|lcl|cTer
KWKAFKKIEKMIRNIRNKIVK
>lcl|2817|lcl|HAPPENN|lcl|subdataset_4|lcl|non-hemolytic|lcl|cTer
AHCLAIGRR
>lcl|2940|lcl|HAPPENN&HAPPENN-hard|lcl|subdataset_4|lcl|non-hemolytic|lcl|cTer
ILPWKWKWWKWRR
>lcl|792|lcl|HAPPENN|lcl|subdataset_11|lcl|non-hemolytic
GKKLKKIGQKIKNFFQKL
>lcl|3112|lcl|HAPPENN|lcl|subdataset_7|lcl|hemolytic|lcl|cTer
ILSAIWSGIKSLF
>lcl|1763|lcl|HAPPENN|lcl|subdataset_8|lcl|hemolytic
GKPTCGETCFKGKCYTPGCTCSYPLCKKD
>lcl|2276|lcl|HAPPENN&HAPPENN-RR90&HAPPENN-hard|lcl|subdataset_9|lcl|non-hemolytic|lcl|cTer
GLLSGILGAGKKIVF
>lcl|2787|lcl|HAPPENN&HAPPENN-RR90&HAPPENN-hard|lcl|subdataset_10|lcl|non-hemolytic
FLGVLFKVLSKLL
>lcl|1307|lcl|HAPPENN&HAPPENN-RR90&HAPPENN-hard|lcl|subdataset_6|lcl|hemolytic|lcl|cTer
FLPAIAGILSQLF
>lcl|53|lcl|HAPPENN|lcl|subdataset_5|lcl|hemolytic|lcl|cTer
RWRWRWRW
>lcl|3679|lcl|HAPPENN&HAPPENN-RR90&HAPPENN-hard|lcl|subdataset_6|lcl|non-hemolytic|lcl|cTer
YSLQMGATAIKQVKKLFKKKGG
>lcl|4464|lcl|HAPPENN&HAPPENN-hard|lcl|subdataset_6|lcl|non-hemolytic|lcl|cTer
GIMSSLMKKLKKHIAK
>lcl|4597|lcl|HAPPENN|lcl|subdataset_9|lcl|non-hemolytic
SGKLYYRRKK
>lcl|217|lcl|HAPPENN&HAPPENN-RR90&HAPPENN-hard|lcl|subdataset_4|lcl|non-hemolytic
VGKTWIKVIRGIGKSKIKWQ
>lcl|1905|lcl|HAPPENN|lcl|subdataset_8|lcl|non-hemolytic
GKPRPYSPKPTSHPRPIRV
>lcl|1791|lcl|HAPPENN&HAPPENN-RR90&HAPPENN-hard|lcl|subdataset_8|lcl|hemolytic
WLRRIKAWLRRKRK
>lcl|244|lcl|HAPPENN&HAPPENN-hard|lcl|subdataset_6|lcl|non-hemolytic|lcl|cTer
ILPWKWKFFPWRR
>lcl|260|lcl|HAPPENN&HAPPENN-RR90|lcl|subdataset_7|lcl|non-hemolytic|lcl|cTer
ILPYKYPYYPYRR
>lcl|712|lcl|HAPPENN|lcl|subdataset_6|lcl|non-hemolytic|lcl|cTer
GLPASISWIKRKRQQ
>lcl|4467|lcl|HAPPENN|lcl|subdataset_5|lcl|non-hemolytic
TRSSRAGLQWAVGRVHRLLRK
>lcl|839|lcl|HAPPENN|lcl|subdataset_6|lcl|non-hemolytic|lcl|cTer
LLKWLKKLLKK
>lcl|1143|lcl|HAPPENN|lcl|subdataset_8|lcl|hemolytic|lcl|cTer
FLPLLAGLAANFLPKIFCKITKRC
>lcl|1828|lcl|HAPPENN|lcl|subdataset_10|lcl|hemolytic|lcl|cTer
FLGGLIKWPWWPWRR
>lcl|2006|lcl|HAPPENN&HAPPENN-RR90&HAPPENN-hard|lcl|subdataset_7|lcl|hemolytic|lcl|cTer
GKILKYLLYLLRKYANLIIR
>lcl|2253|lcl|HAPPENN|lcl|subdataset_10|lcl|non-hemolytic|lcl|cTer
KKFFRAWWAPRFLK
>lcl|912|lcl|HAPPENN&HAPPENN-RR90&HAPPENN-hard|lcl|subdataset_8|lcl|hemolytic|lcl|cTer
VSWKKSLGKIIKVVK
>lcl|571|lcl|HAPPENN&HAPPENN-RR90|lcl|subdataset_3|lcl|non-hemolytic|lcl|cTer
AINPKSVQSLL
>lcl|2436|lcl|HAPPENN&HAPPENN-RR90|lcl|subdataset_3|lcl|non-hemolytic|lcl|cTer
RWKIFKKVVKKA
>lcl|2870|lcl|HAPPENN|lcl|subdataset_3|lcl|non-hemolytic
KKKLKFLKLQ
>lcl|3452|lcl|HAPPENN&HAPPENN-RR90|lcl|subdataset_3|lcl|non-hemolytic
RIIDLLARVRRPQKPKFVTVWVR
>lcl|8|lcl|HAPPENN|lcl|subdataset_3|lcl|hemolytic
ALWMTLLKKVLKAAAK
>lcl|1770|lcl|HAPPENN&HAPPENN-hard|lcl|subdataset_3|lcl|non-hemolytic|lcl|cTer
PFFWRIRIRR
>lcl|3556|lcl|HAPPENN&HAPPENN-RR90&HAPPENN-hard|lcl|subdataset_3|lcl|hemolytic
GMATKAGTAFGKAAKAIIGAAL
```

```
>lcl|1802|lcl|HAPPENN&HAPPENN-hard|lcl|subdataset_3|lcl|non-hemolytic|lcl|cTer
RLKKIGKVLKWI
>lcl|4802|lcl|HAPPENN|lcl|subdataset_3|lcl|non-hemolytic
THVFRLKKWMQKVIDRFGG
>lcl|1384|lcl|HAPPENN&HAPPENN-RR90&HAPPENN-hard|lcl|subdataset_3|lcl|hemolytic
PKLLKFLSKWIG
>lcl|3009|lcl|HAPPENN|lcl|subdataset_3|lcl|non-hemolytic
IIIQYEGHKH
>lcl|3557|lcl|HAPPENN&HAPPENN-RR90&HAPPENN-hard|lcl|subdataset_3|lcl|hemolytic|lcl|cTer
ILPIRSLIKKLL
>lcl|3857|lcl|HAPPENN&HAPPENN-RR90|lcl|subdataset_3|lcl|non-hemolytic|lcl|cTer
RKKKFKFFKRLF
>lcl|418|lcl|HAPPENN&HAPPENN-RR90&HAPPENN-hard|lcl|subdataset_3|lcl|hemolytic|lcl|cTer
HFLKTLVNLAKKIL
>lcl|3344|lcl|HAPPENN|lcl|subdataset_3|lcl|non-hemolytic|lcl|cTer
WKSYVRRW
>lcl|1713|lcl|HAPPENN&HAPPENN-RR90&HAPPENN-hard|lcl|subdataset_3|lcl|hemolytic|lcl|cTer
FAKGIAGMAGKLF
>lcl|4183|lcl|HAPPENN&HAPPENN-hard|lcl|subdataset_3|lcl|non-hemolytic
GKKLFKKILKYLAGPAKFLHSAK
>lcl|4466|lcl|HAPPENN&HAPPENN-RR90&HAPPENN-hard|lcl|subdataset_3|lcl|hemolytic|lcl|cTer
GIMSSLMKKLAAIIAK
>lcl|2604|lcl|HAPPENN|lcl|subdataset_3|lcl|hemolytic|lcl|cTer
FLPLVLGALSGILPKIL
>lcl|422|lcl|HAPPENN&HAPPENN-RR90&HAPPENN-hard|lcl|subdataset_3|lcl|hemolytic|lcl|cTer
HFLLTLVKLAKKIL
>lcl|2891|lcl|HAPPENN|lcl|subdataset_3|lcl|non-hemolytic
LKLKKFKLKQ
>lcl|52|lcl|HAPPENN|lcl|subdataset_3|lcl|hemolytic|lcl|cTer
GIGAVLKVLTTGLPALISWIKRKRQQ
>lcl|639|lcl|HAPPENN&HAPPENN-RR90|lcl|subdataset_3|lcl|non-hemolytic|lcl|cTer
KWKLFKKIPHLAKKF
>lcl|2255|lcl|HAPPENN&HAPPENN-RR90&HAPPENN-hard|lcl|subdataset_3|lcl|non-hemolytic
GIMDTVKNAAKNLAGQLLDKLKCKITAC
>lcl|1608|lcl|HAPPENN&HAPPENN-RR90|lcl|subdataset_3|lcl|non-hemolytic
FKAWRWAWRMKKLAAPS
>lcl|1727|lcl|HAPPENN&HAPPENN-hard|lcl|subdataset_3|lcl|non-hemolytic
KEFKRIVQRIKDFLRNLV
>lcl|4507|lcl|HAPPENN|lcl|subdataset_3|lcl|non-hemolytic
RIKRFWPVVIRTVVAG
>lcl|2194|lcl|HAPPENN&HAPPENN-RR90|lcl|subdataset_3|lcl|non-hemolytic|lcl|cTer
CWWKKKKKWWC
>lcl|4794|lcl|HAPPENN&HAPPENN-RR90|lcl|subdataset_3|lcl|non-hemolytic|lcl|cTer
WRPGRWWRPGRWWRPGRW
>lcl|4373|lcl|HAPPENN|lcl|subdataset_3|lcl|non-hemolytic|lcl|cTer
GMLKRIKTLM
>lcl|3862|lcl|HAPPENN|lcl|subdataset_3|lcl|non-hemolytic|lcl|cTer
KKKKIIKRLI
>lcl|1742|lcl|HAPPENN&HAPPENN-RR90&HAPPENN-hard|lcl|subdataset_3|lcl|hemolytic|lcl|cTer
ILGKLWEGLKSLF
>lcl|108|lcl|HAPPENN&HAPPENN-RR90&HAPPENN-hard|lcl|subdataset_3|lcl|hemolytic
RGGRLCYCRRRFCVCVGR
>lcl|4413|lcl|HAPPENN&HAPPENN-RR90&HAPPENN-hard|lcl|subdataset_3|lcl|hemolytic|lcl|cTer
KWFKIQLQIKKWKNKK
>lcl|640|lcl|HAPPENN|lcl|subdataset_3|lcl|non-hemolytic|lcl|cTer
KWKLFKKIPKFLHLA
>lcl|98|lcl|HAPPENN&HAPPENN-RR90|lcl|subdataset_3|lcl|non-hemolytic|lcl|cTer
KKKKKKLLFAAWAAFAA
>lcl|1994|lcl|HAPPENN&HAPPENN-RR90&HAPPENN-hard|lcl|subdataset_3|lcl|non-hemolytic|lcl|cTer
ILGEIWKGIKDIL
>lcl|288|lcl|HAPPENN&HAPPENN-RR90&HAPPENN-hard|lcl|subdataset_3|lcl|hemolytic
NPVLVKDATGSTQFGPVQALGAQYSMWKLK
>lcl|621|lcl|HAPPENN&HAPPENN-RR90&HAPPENN-hard|lcl|subdataset_3|lcl|hemolytic
FLPLLAGLCKITRKCAANFLPKIF
>lcl|384|lcl|HAPPENN|lcl|subdataset_3|lcl|hemolytic
GIGAVLKVLTTGLPALISWIKRKRQQ
>lcl|3366|lcl|HAPPENN|lcl|subdataset_3|lcl|hemolytic|lcl|cTer
```

```
LPWKWPWWPWRR
>lcl|4722|lcl|HAPPENN&HAPPENN-RR90&HAPPENN-hard|lcl|subdataset_3|lcl|hemolytic|lcl|cTer
MPLSWFFPRTWGKR
>lcl|3696|lcl|HAPPENN|lcl|subdataset_3|lcl|non-hemolytic|lcl|nTer|lcl|cTer
KFFRKLKKSVKKRAKEFFKKPRVIGVSIPF
>lcl|2541|lcl|HAPPENN|lcl|subdataset_3|lcl|non-hemolytic|lcl|cTer
TPFKISIHL
>lcl|1947|lcl|HAPPENN&HAPPENN-RR90&HAPPENN-hard|lcl|subdataset_3|lcl|non-hemolytic|lcl|nTer|lcl|cTer
WRKLWRGGLKRWLK
>lcl|734|lcl|HAPPENN&HAPPENN-RR90|lcl|subdataset_3|lcl|non-hemolytic
GCRFCCNCCPNMSGCGVCCRF
>lcl|1846|lcl|HAPPENN&HAPPENN-RR90&HAPPENN-hard|lcl|subdataset_3|lcl|non-hemolytic
VIDKAKMESLGITSRDTT
>lcl|896|lcl|HAPPENN&HAPPENN-RR90|lcl|subdataset_3|lcl|non-hemolytic|lcl|cTer
KWKSFIKKLTSKALHSAKKF
>lcl|3389|lcl|HAPPENN&HAPPENN-RR90&HAPPENN-hard|lcl|subdataset_3|lcl|hemolytic|lcl|cTer
ILPWKWPWWPWKK
>lcl|3231|lcl|HAPPENN&HAPPENN-RR90&HAPPENN-hard|lcl|subdataset_3|lcl|non-hemolytic
FFGHLYRGITSVVKHVHGLLSG
>lcl|1409|lcl|HAPPENN&HAPPENN-RR90&HAPPENN-hard|lcl|subdataset_3|lcl|hemolytic|lcl|cTer
INWLKLGKKILGAL
>lcl|2186|lcl|HAPPENN&HAPPENN-hard|lcl|subdataset_3|lcl|non-hemolytic|lcl|cTer
LNALKKVFQGIHEAIKLI
>lcl|1149|lcl|HAPPENN|lcl|subdataset_3|lcl|hemolytic|lcl|nTer|lcl|cTer
GIGAVLKVLTTGLPALISWIKRKRQQ
>lcl|4668|lcl|HAPPENN|lcl|subdataset_3|lcl|non-hemolytic|lcl|cTer
KRFKKFFKKVKKSV
>lcl|3930|lcl|HAPPENN|lcl|subdataset_3|lcl|hemolytic|lcl|cTer
LLPIVGNLLKSLLGAKRKRAG
>lcl|699|lcl|HAPPENN&HAPPENN-RR90&HAPPENN-hard|lcl|subdataset_3|lcl|hemolytic
GILSTFKGLAKGVAKDLAGNLLDKFKCKITGC
>lcl|1596|lcl|HAPPENN&HAPPENN-hard|lcl|subdataset_3|lcl|non-hemolytic|lcl|cTer
KIAGKIAKIAGKIA
>lcl|1397|lcl|HAPPENN&HAPPENN-RR90&HAPPENN-hard|lcl|subdataset_3|lcl|hemolytic|lcl|cTer
INWKKIFQKVKNLV
>lcl|3148|lcl|HAPPENN&HAPPENN-RR90|lcl|subdataset_3|lcl|non-hemolytic
LLPPWLRPRNG
>lcl|3487|lcl|HAPPENN|lcl|subdataset_3|lcl|non-hemolytic
KVANTVQKLKRKAKNAVA
>lcl|1743|lcl|HAPPENN&HAPPENN-RR90&HAPPENN-hard|lcl|subdataset_3|lcl|non-hemolytic|lcl|cTer
ILGKIWEGIESLF
>lcl|478|lcl|HAPPENN&HAPPENN-hard|lcl|subdataset_3|lcl|non-hemolytic
GASLSFKILKTVLEALGNVKRK
>lcl|3007|lcl|HAPPENN|lcl|subdataset_3|lcl|non-hemolytic
AWKLFDDGV
>lcl|3142|lcl|HAPPENN&HAPPENN-RR90&HAPPENN-hard|lcl|subdataset_3|lcl|non-hemolytic
IPWKLPATFRPVERPFSKPFCRKD
>lcl|2695|lcl|HAPPENN&HAPPENN-RR90|lcl|subdataset_3|lcl|non-hemolytic|lcl|cTer
ALRSAVRTVARVGRAVLPHVAI
>lcl|2781|lcl|HAPPENN&HAPPENN-RR90&HAPPENN-hard|lcl|subdataset_3|lcl|non-hemolytic
FLGALFKAVSKLL
>lcl|636|lcl|HAPPENN|lcl|subdataset_3|lcl|non-hemolytic|lcl|cTer
KWKLFKKIPKFLHLAKK
>lcl|4155|lcl|HAPPENN&HAPPENN-hard|lcl|subdataset_3|lcl|non-hemolytic
KKLFKKILKYLAGPA
>lcl|3065|lcl|HAPPENN&HAPPENN-RR90&HAPPENN-hard|lcl|subdataset_3|lcl|non-hemolytic|lcl|cTer
FLKPLFNAALKLLP
>lcl|3020|lcl|HAPPENN|lcl|subdataset_3|lcl|hemolytic|lcl|cTer
GMWSKILGHL
>lcl|2223|lcl|HAPPENN&HAPPENN-RR90&HAPPENN-hard|lcl|subdataset_3|lcl|hemolytic|lcl|cTer
GLLDFLKAAGKGLVSNLLEK
>lcl|1707|lcl|HAPPENN|lcl|subdataset_3|lcl|hemolytic|lcl|cTer
YGRKKRRQRRR
>lcl|2294|lcl|HAPPENN&HAPPENN-RR90|lcl|subdataset_3|lcl|non-hemolytic|lcl|cTer
TESYFVFSVGM
```

```
>lcl|4512|lcl|HAPPENN|lcl|subdataset_3|lcl|non-hemolytic
RIKRFWPVVIRT
>lcl|1098|lcl|HAPPENN&HAPPENN-RR90&HAPPENN-hard|lcl|subdataset_3|lcl|hemolytic
LALKSGGWLRLFGLKDKKH
>lcl|2629|lcl|HAPPENN|lcl|subdataset_3|lcl|hemolytic|lcl|cTer
GIGAVLVVLTTGLPALISWIKRKRQQ
>lcl|3216|lcl|HAPPENN&HAPPENN-RR90&HAPPENN-hard|lcl|subdataset_3|lcl|non-hemolytic|lcl|cTer
SIGAKILGGVKTFFKGALKELAFTYLQ
>lcl|1164|lcl|HAPPENN&HAPPENN-hard|lcl|subdataset_3|lcl|non-hemolytic|lcl|cTer
LPLILRKIVTAL
>lcl|4611|lcl|HAPPENN|lcl|subdataset_3|lcl|non-hemolytic|lcl|cTer
PLLKKLLKKP
>lcl|386|lcl|HAPPENN&HAPPENN-RR90&HAPPENN-hard|lcl|subdataset_3|lcl|hemolytic
CKKIGAVLKVLTTGLPALISWIKRKRQQKKC
>lcl|3762|lcl|HAPPENN|lcl|subdataset_3|lcl|hemolytic|lcl|cTer
LALKSGGWLRLFGLKDKKH
>lcl|911|lcl|HAPPENN&HAPPENN-RR90&HAPPENN-hard|lcl|subdataset_3|lcl|hemolytic|lcl|cTer
VNWKKILGKSIKVSK
>lcl|2624|lcl|HAPPENN|lcl|subdataset_3|lcl|hemolytic|lcl|cTer
GIGAVLDVLTTGLPALISWIKRKRQQ
>lcl|4820|lcl|HAPPENN&HAPPENN-RR90&HAPPENN-hard|lcl|subdataset_3|lcl|non-hemolytic|lcl|cTer
GFWGKLLEGVKKAI
>lcl|3129|lcl|HAPPENN&HAPPENN-RR90&HAPPENN-hard|lcl|subdataset_3|lcl|hemolytic
FLPVLTGLTPSIVPKLVCLLTKKC
>lcl|3191|lcl|HAPPENN&HAPPENN-RR90&HAPPENN-hard|lcl|subdataset_3|lcl|hemolytic
FFGAIAAALPHVISAIKNAL
>lcl|814|lcl|HAPPENN&HAPPENN-RR90|lcl|subdataset_3|lcl|non-hemolytic|lcl|cTer
SWSSFFKKAAHSGKHVGKSASTHYL
>lcl|1191|lcl|HAPPENN&HAPPENN-RR90&HAPPENN-hard|lcl|subdataset_3|lcl|hemolytic
CKLLKTFLSKWIC
>lcl|567|lcl|HAPPENN|lcl|subdataset_3|lcl|non-hemolytic|lcl|cTer
LDPKVVQSLL
>lcl|1204|lcl|HAPPENN|lcl|subdataset_3|lcl|hemolytic|lcl|cTer
FFGAIAAALPHVISAIKNAL
>lcl|3528|lcl|HAPPENN&HAPPENN-RR90&HAPPENN-hard|lcl|subdataset_3|lcl|non-hemolytic|lcl|cTer
VIPIVSGLLSSLL
>lcl|1951|lcl|HAPPENN&HAPPENN-RR90&HAPPENN-hard|lcl|subdataset_3|lcl|non-hemolytic|lcl|cTer
ILGKLWEGVKSI
>lcl|1946|lcl|HAPPENN&HAPPENN-RR90&HAPPENN-hard|lcl|subdataset_3|lcl|hemolytic|lcl|cTer
GEILCNLCTGLINTLENLLTTKRKRQQ
>lcl|3127|lcl|HAPPENN&HAPPENN-RR90&HAPPENN-hard|lcl|subdataset_3|lcl|hemolytic
GILDTFKGVAKGVAKDLAVHMLENLKCKMTGC
>lcl|2207|lcl|HAPPENN&HAPPENN-hard|lcl|subdataset_3|lcl|non-hemolytic|lcl|cTer
RPAWRKAAFRVMRACV
>lcl|4302|lcl|HAPPENN&HAPPENN-RR90&HAPPENN-hard|lcl|subdataset_3|lcl|hemolytic
FLPGLIKAAVGIGSTIFCKISKKC
>lcl|795|lcl|HAPPENN|lcl|subdataset_3|lcl|non-hemolytic
GEKLKKIGQKIKNFFKKL
>lcl|570|lcl|HAPPENN&HAPPENN-RR90&HAPPENN-hard|lcl|subdataset_3|lcl|hemolytic|lcl|cTer
FDLLGLVKSVVSAL
>lcl|4092|lcl|HAPPENN&HAPPENN-hard|lcl|subdataset_3|lcl|non-hemolytic
RKSKEKIGKEFKRIVQRIKDFLRNLVPRTES
>lcl|3062|lcl|HAPPENN&HAPPENN-hard|lcl|subdataset_3|lcl|non-hemolytic|lcl|cTer
GLNALKKVAQGIHEAIKLINNHVQ
>lcl|3304|lcl|HAPPENN&HAPPENN-RR90&HAPPENN-hard|lcl|subdataset_3|lcl|non-hemolytic|lcl|cTer
GLKKIFKKGLGSLVKGIAAHVAS
>lcl|1254|lcl|HAPPENN&HAPPENN-RR90&HAPPENN-hard|lcl|subdataset_3|lcl|hemolytic
KWKSFLRTFKSPVRTVFHTALKPISS
>lcl|1192|lcl|HAPPENN|lcl|subdataset_3|lcl|hemolytic|lcl|cTer
CKLLKTFLSKWIC
>lcl|2095|lcl|HAPPENN&HAPPENN-RR90|lcl|subdataset_3|lcl|non-hemolytic|lcl|cTer
GILDAIKAIAKAAG
>lcl|4524|lcl|HAPPENN|lcl|subdataset_3|lcl|non-hemolytic
RTVVAGYNLYRAIKKK
>lcl|4788|lcl|HAPPENN|lcl|subdataset_3|lcl|hemolytic|lcl|cTer
GVVVRVPRVVVRWVRR
>lcl|643|lcl|HAPPENN|lcl|subdataset_3|lcl|non-hemolytic|lcl|cTer
```

```
KWKLFKKIPLKKF
>lcl|2357|lcl|HAPPENN&HAPPENN-hard|lcl|subdataset_3|lcl|non-hemolytic|lcl|cTer
KAIVQRIKDFLR
>lcl|3987|lcl|HAPPENN|lcl|subdataset_3|lcl|non-hemolytic|lcl|cTer
GVVDALKGAAKDIAGHLASKVMNKL
>lcl|1216|lcl|HAPPENN|lcl|subdataset_3|lcl|hemolytic|lcl|cTer
LKLLSIVSWAKKVL
>lcl|2968|lcl|HAPPENN|lcl|subdataset_3|lcl|hemolytic|lcl|cTer
GLLKVIKTLL
>lcl|788|lcl|HAPPENN&HAPPENN-hard|lcl|subdataset_3|lcl|non-hemolytic
GWGSFFKKAAHVAKHVGKAALTHYL
>lcl|1224|lcl|HAPPENN|lcl|subdataset_3|lcl|hemolytic|lcl|cTer
GIGKFIHSAKKFGKLFVGEIMNS
>lcl|190|lcl|HAPPENN&HAPPENN-RR90&HAPPENN-hard|lcl|subdataset_3|lcl|hemolytic|lcl|cTer
KLALKLALKALKAALKLA
>lcl|1049|lcl|HAPPENN&HAPPENN-hard|lcl|subdataset_3|lcl|non-hemolytic
GLKKIFKKGLGSLVKGIAAHVAS
>lcl|315|lcl|HAPPENN|lcl|subdataset_3|lcl|hemolytic
NAGSLLSGWG
>lcl|2738|lcl|HAPPENN&HAPPENN-RR90|lcl|subdataset_3|lcl|non-hemolytic
GYNYAKKLANLAKKFANALW
>lcl|2213|lcl|HAPPENN&HAPPENN-RR90&HAPPENN-hard|lcl|subdataset_3|lcl|hemolytic|lcl|cTer
RRWRIVVIRVRR
>lcl|2371|lcl|HAPPENN|lcl|subdataset_3|lcl|non-hemolytic|lcl|cTer
FFRRFRRFF
>lcl|574|lcl|HAPPENN|lcl|subdataset_3|lcl|non-hemolytic|lcl|cTer
LSPAAMASLA
>lcl|2892|lcl|HAPPENN|lcl|subdataset_3|lcl|non-hemolytic
RPVYIPQPRPPHPRL
>lcl|191|lcl|HAPPENN|lcl|subdataset_3|lcl|hemolytic|lcl|nTer|lcl|cTer
KLALKLALKALKAALKLA
>lcl|1157|lcl|HAPPENN|lcl|subdataset_3|lcl|hemolytic
MAQDIISTIGDLVKWIIDTVNKFPKK
>lcl|2648|lcl|HAPPENN|lcl|subdataset_3|lcl|hemolytic|lcl|cTer
FFPIVGKLLSGLF
>lcl|831|lcl|HAPPENN|lcl|subdataset_3|lcl|non-hemolytic
WKSYVRRW
>lcl|2517|lcl|HAPPENN&HAPPENN-RR90|lcl|subdataset_3|lcl|non-hemolytic|lcl|cTer
KKLGLCLGLKK
>lcl|34|lcl|HAPPENN&HAPPENN-RR90&HAPPENN-hard|lcl|subdataset_3|lcl|hemolytic|lcl|cTer
FRWCFRVCYKGRCRYKCR
>lcl|1054|lcl|HAPPENN|lcl|subdataset_3|lcl|non-hemolytic|lcl|cTer
KWLKKWL
>lcl|4650|lcl|HAPPENN|lcl|subdataset_3|lcl|hemolytic|lcl|cTer
DIFGAIWPLALGALKNLIK
>lcl|2292|lcl|HAPPENN&HAPPENN-RR90|lcl|subdataset_3|lcl|non-hemolytic
VWNQPVRGFKVYE
>lcl|3917|lcl|HAPPENN&HAPPENN-RR90&HAPPENN-hard|lcl|subdataset_3|lcl|hemolytic|lcl|cTer
FAVTWATKWWKG
>lcl|2368|lcl|HAPPENN|lcl|subdataset_3|lcl|non-hemolytic|lcl|cTer
RLRLLLRLR
>lcl|2273|lcl|HAPPENN&HAPPENN-RR90|lcl|subdataset_3|lcl|non-hemolytic
VGTDFSGNDDISDVQK
>lcl|1544|lcl|HAPPENN&HAPPENN-RR90&HAPPENN-hard|lcl|subdataset_3|lcl|non-hemolytic|lcl|cTer
GRRRRSVQWCA
>lcl|2359|lcl|HAPPENN&HAPPENN-RR90|lcl|subdataset_3|lcl|non-hemolytic|lcl|cTer
KRIVQRIADFLR
>lcl|2919|lcl|HAPPENN|lcl|subdataset_3|lcl|non-hemolytic
LKLLKFKKKQ
>lcl|1749|lcl|HAPPENN&HAPPENN-hard|lcl|subdataset_3|lcl|non-hemolytic
FFGWLIKGAIHAGKAIHPLIHRRRH
>lcl|413|lcl|HAPPENN|lcl|subdataset_3|lcl|hemolytic|lcl|cTer
RGGRLCYCRRRFCVCVGR
>lcl|4113|lcl|HAPPENN&HAPPENN-RR90|lcl|subdataset_3|lcl|non-hemolytic|lcl|cTer
RFRRLRDKTRDRLKKI
>lcl|282|lcl|HAPPENN|lcl|subdataset_3|lcl|hemolytic|lcl|cTer
FFFLSRIF
>lcl|246|lcl|HAPPENN|lcl|subdataset_3|lcl|hemolytic|lcl|nTer|lcl|cTer
KLLLKLWLKLLK
>lcl|1262|lcl|HAPPENN&HAPPENN-hard|lcl|subdataset_3|lcl|non-hemolytic
```

```
FLKPLFNAALKLLP
>lcl|3169|lcl|HAPPENN&HAPPENN-RR90&HAPPENN-hard|lcl|subdataset_3|lcl|hemolytic
FMGSALRIAAKVLPAALCQIFKKC
>lcl|841|lcl|HAPPENN&HAPPENN-RR90|lcl|subdataset_3|lcl|non-hemolytic|lcl|cTer
LKKLLKWLKKL
>lcl|459|lcl|HAPPENN&HAPPENN-RR90&HAPPENN-hard|lcl|subdataset_3|lcl|non-hemolytic
KRLFRRLLFSMKKY
>lcl|3030|lcl|HAPPENN|lcl|subdataset_3|lcl|hemolytic|lcl|cTer
GKWKKILGHLIR
>lcl|1530|lcl|HAPPENN&HAPPENN-RR90&HAPPENN-hard|lcl|subdataset_3|lcl|non-hemolytic
AKKVFKRLGIGAVLKVLTTG
>lcl|627|lcl|HAPPENN&HAPPENN-hard|lcl|subdataset_3|lcl|non-hemolytic|lcl|cTer
WKLFKKIPKFLHLAKKF
>lcl|3621|lcl|HAPPENN|lcl|subdataset_3|lcl|hemolytic|lcl|cTer
RLWLAIGRR
>lcl|1917|lcl|HAPPENN&HAPPENN-RR90|lcl|subdataset_3|lcl|non-hemolytic
LPLPSCSSHGGDADNTSQRN
>lcl|2326|lcl|HAPPENN&HAPPENN-RR90&HAPPENN-hard|lcl|subdataset_3|lcl|hemolytic|lcl|cTer
FFSLIPSLVKKLIKAFK
>lcl|3732|lcl|HAPPENN|lcl|subdataset_3|lcl|hemolytic|lcl|nTer|lcl|cTer
RGLRRLGRKIAHGVKKYGPTVLRIIRIAGGGGGSC
>lcl|953|lcl|HAPPENN|lcl|subdataset_3|lcl|hemolytic|lcl|cTer
FLPLIGRVLSAIL
>lcl|3055|lcl|HAPPENN&HAPPENN-RR90&HAPPENN-hard|lcl|subdataset_3|lcl|hemolytic|lcl|cTer
GKWQSLLKHILK
>lcl|2237|lcl|HAPPENN&HAPPENN-RR90&HAPPENN-hard|lcl|subdataset_3|lcl|hemolytic|lcl|cTer
WKRWVQRWKRFLR
>lcl|4340|lcl|HAPPENN&HAPPENN-RR90|lcl|subdataset_3|lcl|non-hemolytic|lcl|cTer
WGIRDILKYGKPS
>lcl|3220|lcl|HAPPENN&HAPPENN-RR90&HAPPENN-hard|lcl|subdataset_3|lcl|non-hemolytic|lcl|cTer
ILGPVISTIGNVLGGLLKNL
>lcl|698|lcl|HAPPENN&HAPPENN-RR90&HAPPENN-hard|lcl|subdataset_3|lcl|hemolytic
FLPILASLAAKLGPKLFCLVTKKC
>lcl|2077|lcl|HAPPENN&HAPPENN-RR90|lcl|subdataset_3|lcl|non-hemolytic
CKILSKTIKCRIPCGRRKEY
>lcl|3712|lcl|HAPPENN&HAPPENN-RR90&HAPPENN-hard|lcl|subdataset_3|lcl|non-hemolytic|lcl|nTer|lcl|cTer
KNLRRITRKIIHIIKKYG
>lcl|1484|lcl|HAPPENN&HAPPENN-RR90&HAPPENN-hard|lcl|subdataset_3|lcl|hemolytic
CGESCVYIPCISGVIGCSCTDKVCYLNGTP
>lcl|1912|lcl|HAPPENN&HAPPENN-RR90|lcl|subdataset_3|lcl|non-hemolytic
LSVDKRPVLHPEHIYGHNHY
>lcl|654|lcl|HAPPENN&HAPPENN-RR90&HAPPENN-hard|lcl|subdataset_3|lcl|hemolytic|lcl|cTer
LKLLKKLLKKLLKLL
>lcl|4636|lcl|HAPPENN&HAPPENN-RR90&HAPPENN-hard|lcl|subdataset_3|lcl|hemolytic|lcl|cTer
GFLDVIKHVGKAALGVVTHLINQ
>lcl|4066|lcl|HAPPENN&HAPPENN-RR90|lcl|subdataset_3|lcl|non-hemolytic|lcl|cTer
KLAKLAKKLAKLAK
>lcl|3954|lcl|HAPPENN&HAPPENN-hard|lcl|subdataset_3|lcl|non-hemolytic
FFHHIFRGIKHVGKTIHRLVTG
>lcl|4520|lcl|HAPPENN&HAPPENN-hard|lcl|subdataset_3|lcl|non-hemolytic
PVVIRTVVAGYNLYRAIKKK
>lcl|3794|lcl|HAPPENN|lcl|subdataset_3|lcl|non-hemolytic|lcl|cTer
KRIVQRIKDWLR
>lcl|512|lcl|HAPPENN|lcl|subdataset_3|lcl|hemolytic|lcl|cTer
GLLGPLLKIAAKVGSNLL
>lcl|4205|lcl|HAPPENN|lcl|subdataset_3|lcl|hemolytic
FLPGLIKVAVGVGSTILCKITKKC
>lcl|1843|lcl|HAPPENN&HAPPENN-hard|lcl|subdataset_3|lcl|non-hemolytic
ACPIFTKIQGTCYRG
>lcl|1677|lcl|HAPPENN&HAPPENN-RR90|lcl|subdataset_3|lcl|non-hemolytic|lcl|cTer
FRRPFKWPRRFFKFF
>lcl|4214|lcl|HAPPENN&HAPPENN-RR90&HAPPENN-hard|lcl|subdataset_3|lcl|non-hemolytic
GFMDTAKNVAKNVAKNVAVTLLDKLRCKVTGGC
>lcl|1066|lcl|HAPPENN&HAPPENN-hard|lcl|subdataset_3|lcl|non-hemolytic
CVYAYVRVRGVLVRYRRCW
>lcl|3356|lcl|HAPPENN|lcl|subdataset_3|lcl|non-hemolytic|lcl|cTer
IYYPPNHNFP
```

>lcl|3394|lcl|HAPPENN&HAPPENN-RR90|lcl|subdataset_3|lcl|non-hemolytic
KPWRFRRAIRRVRWRKVAPYIPFVVKTVGKK
>lcl|132|lcl|HAPPENN|lcl|subdataset_3|lcl|non-hemolytic
CYCRRRFCVCV
>lcl|2323|lcl|HAPPENN&HAPPENN-RR90|lcl|subdataset_3|lcl|non-hemolytic
LESLASSAVRTANKARAKL
>lcl|1500|lcl|HAPPENN&HAPPENN-RR90&HAPPENN-hard|lcl|subdataset_3|lcl|hemolytic
SLWETIKNAGKGFILNILDKIRCKVAGGCKT
>lcl|4776|lcl|HAPPENN|lcl|subdataset_3|lcl|hemolytic|lcl|cTer
FIGMIPGLIGGLISAFK
>lcl|655|lcl|HAPPENN&HAPPENN-RR90&HAPPENN-hard|lcl|subdataset_3|lcl|hemolytic|lcl|cTer
KLKLLKLLKLLKLLK
>lcl|2611|lcl|HAPPENN|lcl|subdataset_3|lcl|hemolytic|lcl|cTer
ILKWKWPWWPWRR
>lcl|3848|lcl|HAPPENN&HAPPENN-RR90|lcl|subdataset_3|lcl|non-hemolytic|lcl|cTer
RKKRLKVVKRLV
>lcl|739|lcl|HAPPENN&HAPPENN-RR90&HAPPENN-hard|lcl|subdataset_3|lcl|non-hemolytic|lcl|cTer
ILGKIAEGIKSLF
>lcl|546|lcl|HAPPENN&HAPPENN-RR90|lcl|subdataset_3|lcl|non-hemolytic|lcl|cTer
LKLKSIVSAAKKVL
>lcl|4195|lcl|HAPPENN&HAPPENN-RR90&HAPPENN-hard|lcl|subdataset_3|lcl|non-hemolytic
KKLFKKILKYLAGPAVAVVGQATQIAKKDEL
>lcl|3001|lcl|HAPPENN&HAPPENN-RR90&HAPPENN-hard|lcl|subdataset_3|lcl|hemolytic
GLWSKIKEAAKTAGLMAMGFVNDMV
>lcl|3118|lcl|HAPPENN&HAPPENN-RR90&HAPPENN-hard|lcl|subdataset_3|lcl|hemolytic
IFGAIWKGISSLL
>lcl|2144|lcl|HAPPENN&HAPPENN-RR90&HAPPENN-hard|lcl|subdataset_3|lcl|non-hemolytic
GFGLGGLARILCLGNRQWSNFFKKLNRKCAMVKK
>lcl|1193|lcl|HAPPENN|lcl|subdataset_3|lcl|hemolytic
KLALKLALKALKAALKLA
>lcl|67|lcl|HAPPENN|lcl|subdataset_3|lcl|hemolytic
FFHHIFRAIVHVAKTIHRLVTG
>lcl|1233|lcl|HAPPENN&HAPPENN-RR90|lcl|subdataset_3|lcl|non-hemolytic
ALLHHGLNCAKGVLAALLHHGLNCAKGVLA
>lcl|3891|lcl|HAPPENN&HAPPENN-RR90&HAPPENN-hard|lcl|subdataset_3|lcl|hemolytic|lcl|cTer
KRRLFLFRLFRLFLRLFLKK
>lcl|4547|lcl|HAPPENN|lcl|subdataset_3|lcl|non-hemolytic|lcl|cTer
KKLFKKILKVL
>lcl|3163|lcl|HAPPENN&HAPPENN-RR90&HAPPENN-hard|lcl|subdataset_3|lcl|hemolytic
GVSKILHSAGKFGKAFLGEIMKS
>lcl|1154|lcl|HAPPENN&HAPPENN-RR90&HAPPENN-hard|lcl|subdataset_3|lcl|hemolytic
GLMDTLKNLAKTAGKGALQSLLNKASCKLSGQC
>lcl|3138|lcl|HAPPENN&HAPPENN-RR90|lcl|subdataset_3|lcl|non-hemolytic|lcl|cTer
KLKKLWKKLLK
>lcl|4460|lcl|HAPPENN|lcl|subdataset_3|lcl|non-hemolytic|lcl|cTer
GIMSSLMKKLAAHIAK
>lcl|972|lcl|HAPPENN&HAPPENN-RR90&HAPPENN-hard|lcl|subdataset_3|lcl|hemolytic|lcl|cTer
AGWGSIFKHIFKAGKFIHGAIQAHND
>lcl|4835|lcl|HAPPENN|lcl|subdataset_3|lcl|non-hemolytic
QPFSLERW
>lcl|631|lcl|HAPPENN|lcl|subdataset_3|lcl|non-hemolytic|lcl|cTer
WKKIPKFLHLAKKF
>lcl|3682|lcl|HAPPENN|lcl|subdataset_3|lcl|non-hemolytic|lcl|cTer
GATAIKQVKKLWKKKGG
>lcl|2082|lcl|HAPPENN|lcl|subdataset_3|lcl|non-hemolytic|lcl|cTer
LKWLKKL
>lcl|2903|lcl|HAPPENN|lcl|subdataset_3|lcl|non-hemolytic
LKKLKFLKKQ
>lcl|2341|lcl|HAPPENN&HAPPENN-RR90|lcl|subdataset_3|lcl|non-hemolytic
SISLLYGRKKRRQRRR
>lcl|3492|lcl|HAPPENN&HAPPENN-RR90|lcl|subdataset_3|lcl|non-hemolytic|lcl|cTer
KAYSMPRCKYLFRAVLCWL
>lcl|2377|lcl|HAPPENN&HAPPENN-RR90|lcl|subdataset_3|lcl|non-hemolytic|lcl|cTer
RRLRLLLRLRR
>lcl|1175|lcl|HAPPENN&HAPPENN-RR90&HAPPENN-hard|lcl|subdataset_3|lcl|non-hemolytic
FIHHIFRGIVHAGRSIGRFLTG
>lcl|303|lcl|HAPPENN&HAPPENN-hard|lcl|subdataset_3|lcl|non-hemolytic
MESVSDAVQLMLGFGTFVLMLLGLVVELIKNSNKK
>lcl|4008|lcl|HAPPENN&HAPPENN-RR90&HAPPENN-hard|lcl|subdataset_3|lcl|non-hemolytic
LGGAATGVIGYISNQTCPTTACTRAC
>lcl|279|lcl|HAPPENN&HAPPENN-RR90&HAPPENN-hard|lcl|subdataset_3|lcl|non-hemolytic|lcl|

cTer
ILPWKWPAAPARR
>lcl|1838|lcl|HAPPENN&HAPPENN-hard|lcl|subdataset_3|lcl|non-hemolytic|lcl|cTer
IKKILSKIKKLL
>lcl|3109|lcl|HAPPENN&HAPPENN-RR90&HAPPENN-hard|lcl|subdataset_3|lcl|non-hemolytic|lcl|cTer
FLKGIIDTVSKLF
>lcl|599|lcl|HAPPENN|lcl|subdataset_3|lcl|hemolytic
PKLLKTFLSKWIG
>lcl|421|lcl|HAPPENN&HAPPENN-RR90&HAPPENN-hard|lcl|subdataset_3|lcl|non-hemolytic|lcl|cTer
HFLLTLKNLAKKIL
>lcl|2824|lcl|HAPPENN&HAPPENN-RR90|lcl|subdataset_3|lcl|non-hemolytic|lcl|cTer
RSGGYCNGKRVCVCR
>lcl|2358|lcl|HAPPENN&HAPPENN-RR90|lcl|subdataset_3|lcl|non-hemolytic|lcl|cTer
KRIVQAIKDFLR
>lcl|318|lcl|HAPPENN&HAPPENN-RR90&HAPPENN-hard|lcl|subdataset_3|lcl|hemolytic|lcl|cTer
KWKKLLKKLLPLLKKLLK
>lcl|4812|lcl|HAPPENN&HAPPENN-RR90&HAPPENN-hard|lcl|subdataset_3|lcl|hemolytic|lcl|cTer
ILSAIWSGIKGLL
>lcl|2037|lcl|HAPPENN&HAPPENN-RR90|lcl|subdataset_3|lcl|non-hemolytic
EQEENVVKIQAFWKGYKQRKEYM
>lcl|903|lcl|HAPPENN|lcl|subdataset_3|lcl|non-hemolytic
GLPRKILCAIAKKKGKAKGPLKLVCKA
>lcl|454|lcl|HAPPENN|lcl|subdataset_3|lcl|non-hemolytic|lcl|cTer
KWKLFKKIPKFLHLAKKF
>lcl|2098|lcl|HAPPENN&HAPPENN-RR90&HAPPENN-hard|lcl|subdataset_3|lcl|hemolytic|lcl|cTer
KALAKALAKLWKALAKAA
>lcl|1391|lcl|HAPPENN&HAPPENN-RR90&HAPPENN-hard|lcl|subdataset_3|lcl|non-hemolytic
WNPFKELERAGQRVRDAIISAAP
>lcl|2878|lcl|HAPPENN|lcl|subdataset_3|lcl|non-hemolytic
KKKKLFLLKQ
>lcl|2367|lcl|HAPPENN&HAPPENN-RR90|lcl|subdataset_3|lcl|non-hemolytic|lcl|nTer|lcl|cTer
FKKLKKLFSKLWNWKRKKRRQRRR
>lcl|2953|lcl|HAPPENN|lcl|subdataset_3|lcl|non-hemolytic|lcl|cTer
GALKRIKTLL
>lcl|1768|lcl|HAPPENN&HAPPENN-hard|lcl|subdataset_3|lcl|non-hemolytic|lcl|cTer
KLLKFIKKLL
>lcl|349|lcl|HAPPENN|lcl|subdataset_3|lcl|hemolytic
AQDIISTIGDLVKWIIDTVNKFTKK
>lcl|3998|lcl|HAPPENN|lcl|subdataset_3|lcl|hemolytic|lcl|cTer
KVLKAAA
>lcl|3811|lcl|HAPPENN|lcl|subdataset_3|lcl|non-hemolytic|lcl|cTer
RKKRLKLLKRLV
>lcl|607|lcl|HAPPENN&HAPPENN-RR90|lcl|subdataset_3|lcl|non-hemolytic
VLSAADKGNVKAAWGKVGGHAAE
>lcl|127|lcl|HAPPENN&HAPPENN-RR90|lcl|subdataset_3|lcl|non-hemolytic
LCYTRRRFTVCV
>lcl|177|lcl|HAPPENN|lcl|subdataset_3|lcl|non-hemolytic
KFFRKLKKSVKKRAKEFFKKPRVIGVSIPF
>lcl|2431|lcl|HAPPENN&HAPPENN-RR90|lcl|subdataset_3|lcl|non-hemolytic
ASHLGHHALDHLLK
>lcl|3798|lcl|HAPPENN&HAPPENN-RR90&HAPPENN-hard|lcl|subdataset_3|lcl|hemolytic|lcl|cTer
KRIVKLILKWLR
>lcl|4527|lcl|HAPPENN|lcl|subdataset_3|lcl|non-hemolytic
VAGYNLYRAIKKK
>lcl|2270|lcl|HAPPENN|lcl|subdataset_3|lcl|non-hemolytic
CPAIQRCC
>lcl|4501|lcl|HAPPENN&HAPPENN-RR90|lcl|subdataset_3|lcl|non-hemolytic|lcl|cTer
AKKVSKRLEKLFSKIQNDK
>lcl|967|lcl|HAPPENN&HAPPENN-RR90&HAPPENN-hard|lcl|subdataset_3|lcl|hemolytic|lcl|cTer
FFRLLFHGVHHGGGYLNAA
>lcl|889|lcl|HAPPENN&HAPPENN-RR90|lcl|subdataset_3|lcl|non-hemolytic|lcl|cTer
KWKSFINKLTSKFLHSAKKF
>lcl|3926|lcl|HAPPENN&HAPPENN-RR90|lcl|subdataset_3|lcl|non-hemolytic|lcl|cTer
RFRRLRKWTRWRLKKI
>lcl|2976|lcl|HAPPENN|lcl|subdataset_3|lcl|hemolytic|lcl|cTer
GLLKRIKLLL
>lcl|3043|lcl|HAPPENN&HAPPENN-RR90&HAPPENN-hard|lcl|subdataset_3|lcl|hemolytic|lcl|cTe

```
r
GKWMSLLKHIWK
>lcl|398|lcl|HAPPENN&HAPPENN-RR90|lcl|subdataset_3|lcl|non-hemolytic|lcl|cTer
VRRFPYYYPFLRR
>lcl|3374|lcl|HAPPENN&HAPPENN-RR90&HAPPENN-hard|lcl|subdataset_3|lcl|hemolytic|lcl|cTer
ILPWAWPWWPWRR
>lcl|4070|lcl|HAPPENN&HAPPENN-RR90&HAPPENN-hard|lcl|subdataset_3|lcl|hemolytic|lcl|cTer
PFKIDIHLGGY
>lcl|1073|lcl|HAPPENN&HAPPENN-RR90&HAPPENN-hard|lcl|subdataset_3|lcl|hemolytic
GIGKFLHSAGKFGKAFIGEIMKS
>lcl|3796|lcl|HAPPENN&HAPPENN-RR90&HAPPENN-hard|lcl|subdataset_3|lcl|non-hemolytic|lcl|cTer
KRIVKRIKKWLR
>lcl|4583|lcl|HAPPENN|lcl|subdataset_3|lcl|non-hemolytic
VWRRWRRFWRR
>lcl|4533|lcl|HAPPENN&HAPPENN-RR90|lcl|subdataset_3|lcl|non-hemolytic|lcl|cTer
GIGAVVKVLTTGVPAVISWIKRKRQQ
>lcl|822|lcl|HAPPENN|lcl|subdataset_3|lcl|hemolytic
FVKLKKILNIINSIFKK
>lcl|4821|lcl|HAPPENN&HAPPENN-RR90|lcl|subdataset_3|lcl|non-hemolytic|lcl|cTer
GFWGKLWEGVKNAIKKK
>lcl|4461|lcl|HAPPENN&HAPPENN-RR90&HAPPENN-hard|lcl|subdataset_3|lcl|non-hemolytic|lcl|cTer
GIMSSLMKKLKAHIAK
>lcl|815|lcl|HAPPENN|lcl|subdataset_3|lcl|hemolytic|lcl|cTer
GFKKLLKGAAKALVKTVLF
>lcl|1024|lcl|HAPPENN|lcl|subdataset_3|lcl|hemolytic
GLPVCGETCVGGTCNTPGCTCSWPVCTRK
>lcl|2827|lcl|HAPPENN|lcl|subdataset_3|lcl|hemolytic|lcl|cTer
YKLLKLLLPKLKPLLFKL
>lcl|647|lcl|HAPPENN|lcl|subdataset_3|lcl|non-hemolytic
KNLRRITRKIIHIIKKYG
>lcl|2798|lcl|HAPPENN|lcl|subdataset_3|lcl|non-hemolytic|lcl|cTer
ALYLAIRRR
>lcl|2792|lcl|HAPPENN&HAPPENN-RR90&HAPPENN-hard|lcl|subdataset_3|lcl|non-hemolytic
GSVLNCGETCLLGTCYTTGCTCNKYRVCTKD
>lcl|2842|lcl|HAPPENN|lcl|subdataset_3|lcl|non-hemolytic|lcl|cTer
RLWRRWRRWLR
>lcl|280|lcl|HAPPENN|lcl|subdataset_3|lcl|non-hemolytic|lcl|cTer
ILPWKAPAAPARR
>lcl|1556|lcl|HAPPENN&HAPPENN-RR90&HAPPENN-hard|lcl|subdataset_3|lcl|non-hemolytic
SRSEKAGLQFPVGRIGRMLKK
>lcl|1679|lcl|HAPPENN&HAPPENN-hard|lcl|subdataset_3|lcl|non-hemolytic|lcl|cTer
LLGMIPVAITAISALSKL
>lcl|1865|lcl|HAPPENN&HAPPENN-hard|lcl|subdataset_3|lcl|non-hemolytic|lcl|cTer
LKKLLKKLLKKL
>lcl|1699|lcl|HAPPENN|lcl|subdataset_3|lcl|non-hemolytic|lcl|cTer
KKLFKKILKHL
>lcl|1701|lcl|HAPPENN|lcl|subdataset_3|lcl|non-hemolytic
FKKFFKKLKNSVKKRAKKFFKKPRVIGVSIPF
>lcl|3953|lcl|HAPPENN|lcl|subdataset_3|lcl|non-hemolytic
VIPFVASVAAEMMQHIFCAASRKC
>lcl|2262|lcl|HAPPENN&HAPPENN-hard|lcl|subdataset_3|lcl|non-hemolytic
GLPVCGETCVGGTCDTPGCTCSWPVCTRN
>lcl|118|lcl|HAPPENN&HAPPENN-hard|lcl|subdataset_3|lcl|non-hemolytic
RGGRLCYCRRRFCVCR
>lcl|3799|lcl|HAPPENN|lcl|subdataset_3|lcl|hemolytic|lcl|cTer
LRIVKLILKWLR
>lcl|2550|lcl|HAPPENN&HAPPENN-hard|lcl|subdataset_3|lcl|non-hemolytic|lcl|nTer|lcl|cTer
KWKSFLKTFKSAGKTVLHTALKAISS
>lcl|4345|lcl|HAPPENN|lcl|subdataset_3|lcl|non-hemolytic
RRLCRIVVIRVCRR
>lcl|3378|lcl|HAPPENN|lcl|subdataset_3|lcl|hemolytic|lcl|cTer
ILPWKWPWAPWRR
>lcl|3542|lcl|HAPPENN&HAPPENN-RR90&HAPPENN-hard|lcl|subdataset_3|lcl|hemolytic
GAFGNLLKGVAKKAGLKILSIAQCKLSGTC
>lcl|4840|lcl|HAPPENN&HAPPENN-RR90|lcl|subdataset_3|lcl|non-hemolytic
SAVWRRWRRFWLRKRRKR
>lcl|3871|lcl|HAPPENN&HAPPENN-RR90&HAPPENN-hard|lcl|subdataset_3|lcl|hemolytic|lcl|cTer
```

```
LRFIKKILKKLI
>lcl|4341|lcl|HAPPENN|lcl|subdataset_3|lcl|non-hemolytic
WGIRDILKYGKPS
>lcl|3894|lcl|HAPPENN|lcl|subdataset_3|lcl|hemolytic|lcl|cTer
GIWDTIKSMGKVFAGAILQNL
>lcl|1798|lcl|HAPPENN|lcl|subdataset_3|lcl|hemolytic|lcl|cTer
WWWLRRIR
>lcl|3376|lcl|HAPPENN&HAPPENN-RR90&HAPPENN-hard|lcl|subdataset_3|lcl|hemolytic|lcl|cTer
ILPWKWAWWPWRR
>lcl|2230|lcl|HAPPENN|lcl|subdataset_3|lcl|hemolytic|lcl|cTer
GIGGALLSFGKSALKGLAKGLAEHF
>lcl|1859|lcl|HAPPENN|lcl|subdataset_3|lcl|hemolytic|lcl|cTer
IIGPVLGLVGKALGGLL
>lcl|697|lcl|HAPPENN|lcl|subdataset_3|lcl|hemolytic
FLPILASLAAKFGPKLFCLVTKKC
>lcl|535|lcl|HAPPENN|lcl|subdataset_3|lcl|hemolytic|lcl|cTer
ILPILGNLLNGLL
>lcl|4474|lcl|HAPPENN&HAPPENN-hard|lcl|subdataset_3|lcl|non-hemolytic|lcl|cTer
GILSKLLKKLKKIIAK
>lcl|1993|lcl|HAPPENN|lcl|subdataset_3|lcl|hemolytic|lcl|cTer
FWGALAKGALKLIPSLVSSFT
>lcl|1815|lcl|HAPPENN&HAPPENN-RR90&HAPPENN-hard|lcl|subdataset_3|lcl|non-hemolytic|lcl|cTer
KKLIKVFAKGFKKAKKLFKGIG
>lcl|4481|lcl|HAPPENN&HAPPENN-RR90&HAPPENN-hard|lcl|subdataset_3|lcl|hemolytic|lcl|cTer
GILSSLWKKLKKWIAK
>lcl|2788|lcl|HAPPENN&HAPPENN-RR90|lcl|subdataset_3|lcl|non-hemolytic
FLGALAKALSKLL
>lcl|166|lcl|HAPPENN&HAPPENN-hard|lcl|subdataset_3|lcl|non-hemolytic
GRRRRSVQWCA
>lcl|254|lcl|HAPPENN|lcl|subdataset_3|lcl|hemolytic|lcl|cTer
ILPLKWPWWPWRR
>lcl|329|lcl|HAPPENN&HAPPENN-RR90|lcl|subdataset_3|lcl|non-hemolytic
VIPFVASVAAEMMHHVYCAASKRC
>lcl|562|lcl|HAPPENN&HAPPENN-hard|lcl|subdataset_3|lcl|non-hemolytic|lcl|cTer
LLKKLLKKLLKK
>lcl|2764|lcl|HAPPENN&HAPPENN-RR90&HAPPENN-hard|lcl|subdataset_3|lcl|hemolytic|lcl|cTer
GLKEIFKAGLGSLVKGIAAHVAN
>lcl|2235|lcl|HAPPENN&HAPPENN-RR90&HAPPENN-hard|lcl|subdataset_3|lcl|hemolytic|lcl|cTer
WKRIVRRIWRWLR
>lcl|2446|lcl|HAPPENN&HAPPENN-RR90|lcl|subdataset_3|lcl|non-hemolytic
AFCWNVCVYRNAVRVCHRRCN
>lcl|160|lcl|HAPPENN&HAPPENN-RR90|lcl|subdataset_3|lcl|non-hemolytic
LCYTHHHFTVCV
>lcl|2340|lcl|HAPPENN|lcl|subdataset_3|lcl|non-hemolytic
YGRKKRRQRRR
>lcl|634|lcl|HAPPENN&HAPPENN-RR90|lcl|subdataset_3|lcl|non-hemolytic|lcl|cTer
WKKIPKFLHLLKKF
>lcl|45|lcl|HAPPENN&HAPPENN-RR90&HAPPENN-hard|lcl|subdataset_3|lcl|hemolytic
GLMDTVKNAAKNLAGQMLDKLKCKITGSC
>lcl|3395|lcl|HAPPENN&HAPPENN-hard|lcl|subdataset_3|lcl|non-hemolytic
KWCFCVCYRGICRCRCRG
>lcl|4773|lcl|HAPPENN|lcl|subdataset_3|lcl|non-hemolytic
ATYRTGRATRESLSGVEISGRLYRLR
>lcl|3809|lcl|HAPPENN|lcl|subdataset_3|lcl|hemolytic|lcl|cTer
KLKLLLKLK
>lcl|1425|lcl|HAPPENN|lcl|subdataset_3|lcl|hemolytic|lcl|cTer
KIASIGKEVLKAL
>lcl|3296|lcl|HAPPENN&HAPPENN-hard|lcl|subdataset_3|lcl|non-hemolytic
RGVAKFASKGLGKDLAKLGVDLVACKISKQC
>lcl|4410|lcl|HAPPENN|lcl|subdataset_3|lcl|hemolytic|lcl|cTer
KIWFQNKKMKWKK
>lcl|2864|lcl|HAPPENN|lcl|subdataset_3|lcl|hemolytic
KKKKLFKLLQ
>lcl|1044|lcl|HAPPENN&HAPPENN-RR90|lcl|subdataset_3|lcl|non-hemolytic
YVPPVQKPHPNGPKFPTFP
>lcl|1725|lcl|HAPPENN&HAPPENN-RR90&HAPPENN-hard|lcl|subdataset_3|lcl|hemolytic|lcl|cTer
GLLWKWGWKWKEFLRIVGY
```

```
>lcl|876|lcl|HAPPENN&HAPPENN-RR90&HAPPENN-hard|lcl|subdataset_3|lcl|non-hemolytic
NWCKRGRKQCKTHPHFVIPY
>lcl|2452|lcl|HAPPENN|lcl|subdataset_3|lcl|non-hemolytic
HWRRFWHR
>lcl|561|lcl|HAPPENN&HAPPENN-hard|lcl|subdataset_3|lcl|non-hemolytic|lcl|cTer
LLRRLLRRLLRR
>lcl|499|lcl|HAPPENN|lcl|subdataset_3|lcl|non-hemolytic
AKKVSKRLEKLFSKIQNDK
>lcl|480|lcl|HAPPENN|lcl|subdataset_3|lcl|non-hemolytic
KLKLKLKE
>lcl|3329|lcl|HAPPENN|lcl|subdataset_3|lcl|hemolytic|lcl|cTer
FVKLKKILNIINSIFKK
>lcl|3893|lcl|HAPPENN&HAPPENN-RR90&HAPPENN-hard|lcl|subdataset_3|lcl|hemolytic|lcl|cTer
KRRKLAFRLFRLFLKLVLKK
>lcl|4158|lcl|HAPPENN|lcl|subdataset_3|lcl|hemolytic
KKLFKKILKYLAGPAKKLFKKILKYL
>lcl|1741|lcl|HAPPENN&HAPPENN-RR90&HAPPENN-hard|lcl|subdataset_3|lcl|non-hemolytic|lcl|cTer
ILGKVWEGVKSLF
>lcl|2656|lcl|HAPPENN|lcl|subdataset_3|lcl|hemolytic
FLSTLLNVASNVVPTLICKITKKC
>lcl|3108|lcl|HAPPENN&HAPPENN-RR90&HAPPENN-hard|lcl|subdataset_3|lcl|non-hemolytic|lcl|cTer
FLKGIIDTVSNWL
>lcl|1338|lcl|HAPPENN&HAPPENN-RR90&HAPPENN-hard|lcl|subdataset_3|lcl|hemolytic|lcl|cTer
LNWGAILKHKIK
>lcl|2988|lcl|HAPPENN&HAPPENN-hard|lcl|subdataset_3|lcl|non-hemolytic
GLLGPLLKIAAKVGSNLL
>lcl|1575|lcl|HAPPENN|lcl|subdataset_3|lcl|hemolytic|lcl|cTer
ALFKTMLKKLGTMAL
>lcl|3763|lcl|HAPPENN|lcl|subdataset_3|lcl|hemolytic
FLGWLFKWASK
>lcl|2528|lcl|HAPPENN&HAPPENN-RR90|lcl|subdataset_1|lcl|non-hemolytic|lcl|cTer
AARIILRWRFR
>lcl|4521|lcl|HAPPENN|lcl|subdataset_1|lcl|non-hemolytic
VVIRTVVAGYNLYRAIKKK
>lcl|2963|lcl|HAPPENN|lcl|subdataset_1|lcl|non-hemolytic|lcl|cTer
GLLKRIKT
>lcl|1970|lcl|HAPPENN&HAPPENN-RR90&HAPPENN-hard|lcl|subdataset_1|lcl|hemolytic
TRSRWRRFIRGAGRFARRYGWRIALGLVG
>lcl|4240|lcl|HAPPENN&HAPPENN-RR90|lcl|subdataset_1|lcl|non-hemolytic
SKVWRHWRRFWHRAHRKK
>lcl|388|lcl|HAPPENN&HAPPENN-RR90|lcl|subdataset_1|lcl|non-hemolytic|lcl|cTer
KWKKLLKKLLKLPKKLLKKLKKLLK
>lcl|2932|lcl|HAPPENN|lcl|subdataset_1|lcl|non-hemolytic
LKLLKFKKLQ
>lcl|3549|lcl|HAPPENN&HAPPENN-RR90&HAPPENN-hard|lcl|subdataset_1|lcl|hemolytic
GLKEVAHSAKKFAKGFISGLTGS
>lcl|543|lcl|HAPPENN&HAPPENN-RR90&HAPPENN-hard|lcl|subdataset_1|lcl|hemolytic
GFGSLLGKALKIGTNLL
>lcl|1563|lcl|HAPPENN|lcl|subdataset_1|lcl|non-hemolytic|lcl|cTer
CLLKKLLKKC
>lcl|893|lcl|HAPPENN&HAPPENN-RR90|lcl|subdataset_1|lcl|non-hemolytic|lcl|cTer
KWKSFIKKLASKFLHSAKKF
>lcl|2149|lcl|HAPPENN&HAPPENN-hard|lcl|subdataset_1|lcl|non-hemolytic|lcl|cTer
FAKLLKKLL
>lcl|4225|lcl|HAPPENN&HAPPENN-RR90&HAPPENN-hard|lcl|subdataset_1|lcl|hemolytic
ILPIIGKILSTIFGK
>lcl|4539|lcl|HAPPENN&HAPPENN-RR90&HAPPENN-hard|lcl|subdataset_1|lcl|hemolytic|lcl|cTer
FFHHIFRGIVHVGKTVHRLVTG
>lcl|4566|lcl|HAPPENN&HAPPENN-RR90|lcl|subdataset_1|lcl|non-hemolytic
LRHKVYGYCVLGP
>lcl|1137|lcl|HAPPENN&HAPPENN-hard|lcl|subdataset_1|lcl|non-hemolytic
RAGLQFPVGRLLRRLLR
>lcl|2594|lcl|HAPPENN|lcl|subdataset_1|lcl|non-hemolytic
YQCGQGG
>lcl|4613|lcl|HAPPENN&HAPPENN-RR90&HAPPENN-hard|lcl|subdataset_1|lcl|non-hemolytic
FLGWLFKVASK
>lcl|2767|lcl|HAPPENN|lcl|subdataset_1|lcl|hemolytic|lcl|cTer
GLPALISWIKRKRQ
```

>lcl|3793|lcl|HAPPENN|lcl|subdataset_1|lcl|non-hemolytic|lcl|cTer
KRIVQRIKDFLR
>lcl|1465|lcl|HAPPENN&HAPPENN-RR90|lcl|subdataset_1|lcl|non-hemolytic|lcl|cTer
KIKLFKKWPKFLHLAKKF
>lcl|394|lcl|HAPPENN&HAPPENN-RR90&HAPPENN-hard|lcl|subdataset_1|lcl|hemolytic
VRRFPWWWPFLRR
>lcl|4305|lcl|HAPPENN&HAPPENN-RR90|lcl|subdataset_1|lcl|non-hemolytic|lcl|nTer|lcl|cTer
KWKSFLKTFKSAKKTVAHTAAKAISS
>lcl|4349|lcl|HAPPENN&HAPPENN-RR90|lcl|subdataset_1|lcl|non-hemolytic|lcl|cTer
KWKKKKKKPKFL
>lcl|70|lcl|HAPPENN&HAPPENN-RR90&HAPPENN-hard|lcl|subdataset_1|lcl|hemolytic
FFHHIFRPIVHVPKTIHRLVTG
>lcl|3934|lcl|HAPPENN&HAPPENN-RR90&HAPPENN-hard|lcl|subdataset_1|lcl|non-hemolytic|lcl|cTer
WKKIWSKIKKLLK
>lcl|3963|lcl|HAPPENN&HAPPENN-RR90&HAPPENN-hard|lcl|subdataset_1|lcl|non-hemolytic|lcl|cTer
IKKIVSKIKKVLK
>lcl|3301|lcl|HAPPENN&HAPPENN-RR90&HAPPENN-hard|lcl|subdataset_1|lcl|hemolytic
IKIPSFFRNILKKVGKEAVSLIAGALKQS
>lcl|1407|lcl|HAPPENN&HAPPENN-RR90&HAPPENN-hard|lcl|subdataset_1|lcl|hemolytic|lcl|cTer
INWKSIFEKVKNLV
>lcl|4290|lcl|HAPPENN&HAPPENN-hard|lcl|subdataset_1|lcl|non-hemolytic
GVFDIIKGAGKQLIARAMGKIAEKVGLNKDGN
>lcl|2797|lcl|HAPPENN&HAPPENN-RR90&HAPPENN-hard|lcl|subdataset_1|lcl|hemolytic|lcl|cTer
KLKLLLLLKLK
>lcl|2291|lcl|HAPPENN&HAPPENN-hard|lcl|subdataset_1|lcl|non-hemolytic
INLKAIAALVKKV
>lcl|2927|lcl|HAPPENN|lcl|subdataset_1|lcl|non-hemolytic
LLKKKFKKLQ
>lcl|4301|lcl|HAPPENN&HAPPENN-RR90&HAPPENN-hard|lcl|subdataset_1|lcl|hemolytic
FLPGLIKAAVGVGSTILCKITKKC
>lcl|2264|lcl|HAPPENN|lcl|subdataset_1|lcl|non-hemolytic
YYHFWHRGVTKRSLSPHRPR
>lcl|300|lcl|HAPPENN|lcl|subdataset_1|lcl|non-hemolytic
MVSVSVAVQLMLGFGTFVLMLLGLVVELIKNSNKK
>lcl|4683|lcl|HAPPENN|lcl|subdataset_1|lcl|hemolytic
IWSFLIKAATKLLPSLFGG
>lcl|250|lcl|HAPPENN&HAPPENN-RR90&HAPPENN-hard|lcl|subdataset_1|lcl|hemolytic|lcl|cTer
IDWLKLGKMVMDVL
>lcl|268|lcl|HAPPENN&HAPPENN-RR90&HAPPENN-hard|lcl|subdataset_1|lcl|non-hemolytic|lcl|cTer
ILPWKWPWLPKRR
>lcl|162|lcl|HAPPENN|lcl|subdataset_1|lcl|hemolytic
LCYCRRRFCFCV
>lcl|2613|lcl|HAPPENN|lcl|subdataset_1|lcl|hemolytic|lcl|cTer
KWPWWPWRR
>lcl|1995|lcl|HAPPENN&HAPPENN-RR90&HAPPENN-hard|lcl|subdataset_1|lcl|hemolytic|lcl|cTer
ILGKIWKGIKNIL
>lcl|2347|lcl|HAPPENN&HAPPENN-RR90&HAPPENN-hard|lcl|subdataset_1|lcl|non-hemolytic|lcl|cTer
AAKKGCWTVSIPPKPCF
>lcl|750|lcl|HAPPENN|lcl|subdataset_1|lcl|hemolytic
RLYRRLYRRLYRRLYRRLYRRLYRRLYRRLYR
>lcl|4728|lcl|HAPPENN&HAPPENN-RR90&HAPPENN-hard|lcl|subdataset_1|lcl|non-hemolytic|lcl|cTer
ICLKKWPWWPWRRCK
>lcl|4606|lcl|HAPPENN|lcl|subdataset_1|lcl|non-hemolytic|lcl|cTer
RGKRWWRRKK
>lcl|264|lcl|HAPPENN&HAPPENN-RR90&HAPPENN-hard|lcl|subdataset_1|lcl|hemolytic|lcl|cTer
ILPWKWPYLPWRR
>lcl|1996|lcl|HAPPENN&HAPPENN-RR90&HAPPENN-hard|lcl|subdataset_1|lcl|hemolytic|lcl|cTer
KIAKGALKALKIAKVALKAL
>lcl|1617|lcl|HAPPENN|lcl|subdataset_1|lcl|non-hemolytic|lcl|cTer
HRWWRWWRR
>lcl|3868|lcl|HAPPENN|lcl|subdataset_1|lcl|hemolytic|lcl|cTer
LRFLKKILKKLF
>lcl|666|lcl|HAPPENN|lcl|subdataset_1|lcl|non-hemolytic

```
GSKKPVPIIYCNRRAATGKCQRM
>lcl|69|lcl|HAPPENN|lcl|subdataset_1|lcl|hemolytic
FFHHIFRGIVHVPKTIHRLVTG
>lcl|3535|lcl|HAPPENN&HAPPENN-RR90&HAPPENN-hard|lcl|subdataset_1|lcl|non-hemolytic
AMPWRPATGLLPIKPTHIKPLCGDD
>lcl|1987|lcl|HAPPENN&HAPPENN-hard|lcl|subdataset_1|lcl|non-hemolytic
GFLEKLKTGAKDFASAFVNSIKGT
>lcl|3585|lcl|HAPPENN&HAPPENN-RR90|lcl|subdataset_1|lcl|non-hemolytic
FWGLKGLKGPGKFSKKL
>lcl|943|lcl|HAPPENN|lcl|subdataset_1|lcl|hemolytic|lcl|cTer
ALPLIGRVLSGIL
>lcl|1335|lcl|HAPPENN&HAPPENN-RR90&HAPPENN-hard|lcl|subdataset_1|lcl|hemolytic|lcl|cTer
LNWGAKLKHIIK
>lcl|3505|lcl|HAPPENN|lcl|subdataset_1|lcl|hemolytic|lcl|cTer
RLYRRLYRRLYRRLYRRLYRRLYRRLYRRLYR
>lcl|3765|lcl|HAPPENN&HAPPENN-hard|lcl|subdataset_1|lcl|non-hemolytic|lcl|cTer
YRWWRWARRW
>lcl|4517|lcl|HAPPENN&HAPPENN-RR90&HAPPENN-hard|lcl|subdataset_1|lcl|hemolytic
RFWPVVIRTVVAGYNLYRAIKKK
>lcl|4230|lcl|HAPPENN|lcl|subdataset_1|lcl|non-hemolytic|lcl|cTer
FRCRRRFCRF
>lcl|489|lcl|HAPPENN|lcl|subdataset_1|lcl|non-hemolytic
FKRLEKLFSKIQNDK
>lcl|4559|lcl|HAPPENN&HAPPENN-RR90|lcl|subdataset_1|lcl|non-hemolytic
GTFIKQQRKQKQQRHHTSGTRKRMAK
>lcl|1104|lcl|HAPPENN&HAPPENN-RR90&HAPPENN-hard|lcl|subdataset_1|lcl|hemolytic|lcl|cTer
ILGKLLSTAAKLLSNL
>lcl|2239|lcl|HAPPENN&HAPPENN-RR90&HAPPENN-hard|lcl|subdataset_1|lcl|hemolytic|lcl|cTer
WKRWVRRWKRWLR
>lcl|3077|lcl|HAPPENN&HAPPENN-RR90&HAPPENN-hard|lcl|subdataset_1|lcl|hemolytic|lcl|cTer
VNWKKILKKIIKVAK
>lcl|4331|lcl|HAPPENN&HAPPENN-RR90|lcl|subdataset_1|lcl|non-hemolytic
HRHQGPIFDTRPSPFNPNQPRPGPIY
>lcl|3842|lcl|HAPPENN&HAPPENN-RR90&HAPPENN-hard|lcl|subdataset_1|lcl|hemolytic
GLKALKKVFKGIHKAIKLINKHVK
>lcl|96|lcl|HAPPENN&HAPPENN-RR90|lcl|subdataset_1|lcl|non-hemolytic|lcl|cTer
KKKKKKAAFLAWALFAA
>lcl|2425|lcl|HAPPENN&HAPPENN-RR90&HAPPENN-hard|lcl|subdataset_1|lcl|non-hemolytic|lcl|cTer
LKPLLKPLLKPLLKPLLKPLLKPLLKPL
>lcl|1368|lcl|HAPPENN&HAPPENN-RR90&HAPPENN-hard|lcl|subdataset_1|lcl|hemolytic|lcl|cTer
GFFALIAQIISSPLFQTL
>lcl|1759|lcl|HAPPENN&HAPPENN-RR90&HAPPENN-hard|lcl|subdataset_1|lcl|hemolytic|lcl|cTer
NLLKSALKTVNKLLAAS
>lcl|4171|lcl|HAPPENN|lcl|subdataset_1|lcl|non-hemolytic
KDWEHLKDWEHLKDWEHL
>lcl|4311|lcl|HAPPENN|lcl|subdataset_1|lcl|hemolytic
GIGKFLHSAKKFGKAAVGEIMNS
>lcl|2361|lcl|HAPPENN&HAPPENN-RR90|lcl|subdataset_1|lcl|non-hemolytic|lcl|cTer
AQIVQRIKDFLR
>lcl|1299|lcl|HAPPENN&HAPPENN-RR90&HAPPENN-hard|lcl|subdataset_1|lcl|hemolytic
GLLSGVLGVGKKVLCGLSGLC
>lcl|852|lcl|HAPPENN&HAPPENN-RR90|lcl|subdataset_1|lcl|non-hemolytic
QLQGKQVSGEVVQKVLQELIQSVAKP
>lcl|4720|lcl|HAPPENN&HAPPENN-RR90&HAPPENN-hard|lcl|subdataset_1|lcl|non-hemolytic|lcl|cTer
FPVTWPTKWWEG
>lcl|2346|lcl|HAPPENN&HAPPENN-RR90|lcl|subdataset_1|lcl|non-hemolytic
GLLRASSKWGRKYYVDLAGCAKA
>lcl|1059|lcl|HAPPENN|lcl|subdataset_1|lcl|hemolytic|lcl|cTer
LKWLLKWLL
>lcl|23|lcl|HAPPENN&HAPPENN-RR90&HAPPENN-hard|lcl|subdataset_1|lcl|hemolytic|lcl|cTer
DAACAAHCLWR
>lcl|1047|lcl|HAPPENN|lcl|subdataset_1|lcl|non-hemolytic
GSKKPVPIIYCNRRSGKCQRM
>lcl|4330|lcl|HAPPENN&HAPPENN-RR90|lcl|subdataset_1|lcl|non-hemolytic|lcl|cTer
GIHDALKYGKPS
```

```
>lcl|2250|lcl|HAPPENN|lcl|subdataset_1|lcl|non-hemolytic|lcl|cTer
HKKHKKHKKHKK
>lcl|3460|lcl|HAPPENN&HAPPENN-hard|lcl|subdataset_1|lcl|non-hemolytic
GLVGTLLGHIGKAILG
>lcl|752|lcl|HAPPENN|lcl|subdataset_1|lcl|non-hemolytic
RLYRRLYRRLYRRLYRKKK
>lcl|1587|lcl|HAPPENN|lcl|subdataset_1|lcl|hemolytic|lcl|cTer
KIAGKIAAIAGKIAKIAGAIAKIAGK
>lcl|4617|lcl|HAPPENN|lcl|subdataset_1|lcl|non-hemolytic
GRRPRPRPRPWWW
>lcl|3184|lcl|HAPPENN|lcl|subdataset_1|lcl|non-hemolytic
PLYKKIIKKLLES
>lcl|3776|lcl|HAPPENN&HAPPENN-RR90|lcl|subdataset_1|lcl|non-hemolytic|lcl|cTer
AKKVFKRLPKLFSKIWNWK
>lcl|925|lcl|HAPPENN|lcl|subdataset_1|lcl|non-hemolytic|lcl|cTer
GWGSFFKKAAHVGKHVGKA
>lcl|4600|lcl|HAPPENN|lcl|subdataset_1|lcl|non-hemolytic
RGKRWWRRKK
>lcl|2697|lcl|HAPPENN&HAPPENN-RR90&HAPPENN-hard|lcl|subdataset_1|lcl|hemolytic|lcl|cTer
LVRGCWTKSYPPKPCFVR
>lcl|3895|lcl|HAPPENN&HAPPENN-RR90|lcl|subdataset_1|lcl|non-hemolytic
RMKKLGNHKVSCERNTKRCRKAI
>lcl|4621|lcl|HAPPENN|lcl|subdataset_1|lcl|non-hemolytic|lcl|cTer
GRRPRPRPRPWWW
>lcl|2592|lcl|HAPPENN&HAPPENN-RR90|lcl|subdataset_1|lcl|non-hemolytic
AGETHTVMINHAGRGAPKLVVGGKKLS
>lcl|399|lcl|HAPPENN&HAPPENN-RR90&HAPPENN-hard|lcl|subdataset_1|lcl|hemolytic|lcl|cTer
VRRYPWWWPYLRR
>lcl|1956|lcl|HAPPENN&HAPPENN-RR90&HAPPENN-hard|lcl|subdataset_1|lcl|hemolytic|lcl|cTer
KAIAKSILKWIKSIAKAI
>lcl|735|lcl|HAPPENN|lcl|subdataset_1|lcl|non-hemolytic
TFRRLKWK
>lcl|3423|lcl|HAPPENN|lcl|subdataset_1|lcl|non-hemolytic|lcl|cTer
KKIMRTFLRRISKDILTGKK
>lcl|239|lcl|HAPPENN&HAPPENN-RR90&HAPPENN-hard|lcl|subdataset_1|lcl|hemolytic|lcl|cTer
GFFGKMKEYFKKFGASFKRRFANLKKRL
>lcl|1258|lcl|HAPPENN|lcl|subdataset_1|lcl|hemolytic
IISTIGKLVKWIIDTV
>lcl|1308|lcl|HAPPENN&HAPPENN-RR90&HAPPENN-hard|lcl|subdataset_1|lcl|hemolytic
ALWKTMLKKLGTMALHAGKAALGAAADTISQGTQ
>lcl|3017|lcl|HAPPENN|lcl|subdataset_1|lcl|hemolytic|lcl|cTer
MWSKILGHLIR
>lcl|2958|lcl|HAPPENN|lcl|subdataset_1|lcl|hemolytic|lcl|cTer
GLLKRIATLL
>lcl|2774|lcl|HAPPENN&HAPPENN-RR90&HAPPENN-hard|lcl|subdataset_1|lcl|hemolytic
GYPICGESCVGGICNIPGCSCSWPVCTTN
>lcl|2676|lcl|HAPPENN|lcl|subdataset_1|lcl|hemolytic|lcl|cTer
FLAGLIGGLAKMLGK
>lcl|2936|lcl|HAPPENN|lcl|subdataset_1|lcl|hemolytic
LLLLKFKKLQ
>lcl|4365|lcl|HAPPENN&HAPPENN-RR90&HAPPENN-hard|lcl|subdataset_1|lcl|hemolytic
RIRFPWPWRWPWWPRFRG
>lcl|2985|lcl|HAPPENN|lcl|subdataset_1|lcl|hemolytic|lcl|cTer
KLLKRIKKLL
>lcl|3379|lcl|HAPPENN&HAPPENN-RR90&HAPPENN-hard|lcl|subdataset_1|lcl|hemolytic|lcl|cTer
ILPWKWPWWAWRR
>lcl|4670|lcl|HAPPENN|lcl|subdataset_1|lcl|hemolytic|lcl|cTer
FLSMIPKIAGGIASLVKNL
>lcl|174|lcl|HAPPENN&HAPPENN-RR90&HAPPENN-hard|lcl|subdataset_1|lcl|hemolytic
MDFIIDIIKKIVGLFTGK
>lcl|4568|lcl|HAPPENN&HAPPENN-RR90&HAPPENN-hard|lcl|subdataset_1|lcl|non-hemolytic
VGRKHSILNCIPYLKKKKIMRL
>lcl|4412|lcl|HAPPENN&HAPPENN-RR90&HAPPENN-hard|lcl|subdataset_1|lcl|hemolytic|lcl|cTer
RWFKIQLQIRRWKNKK
>lcl|3801|lcl|HAPPENN&HAPPENN-RR90&HAPPENN-hard|lcl|subdataset_1|lcl|non-hemolytic|lcl|cTer
KRIRKRIKKWKR
>lcl|55|lcl|HAPPENN&HAPPENN-hard|lcl|subdataset_1|lcl|non-hemolytic|lcl|nTer|lcl|cTer
KWKSFLKTFKSAKKTVLHTALKAISS
```

```
>lcl|667|lcl|HAPPENN|lcl|subdataset_1|lcl|non-hemolytic
GSKKPVPIIYANRRTGKAQRM
>lcl|4787|lcl|HAPPENN&HAPPENN-RR90&HAPPENN-hard|lcl|subdataset_1|lcl|hemolytic|lcl|cTer
GVVVRVGRVVVRWVRRRR
>lcl|4429|lcl|HAPPENN&HAPPENN-RR90&HAPPENN-hard|lcl|subdataset_1|lcl|non-hemolytic|lcl|cTer
AKLWLKAAKLWLKA
>lcl|2812|lcl|HAPPENN&HAPPENN-RR90|lcl|subdataset_1|lcl|non-hemolytic|lcl|cTer
LCAARCLAIGRR
>lcl|890|lcl|HAPPENN&HAPPENN-RR90|lcl|subdataset_1|lcl|non-hemolytic|lcl|cTer
KWKSFIKKSTSKFLHSAKKF
>lcl|2545|lcl|HAPPENN&HAPPENN-hard|lcl|subdataset_1|lcl|non-hemolytic
KRIVQRIKDFLRNLVPRTES
>lcl|3247|lcl|HAPPENN|lcl|subdataset_1|lcl|non-hemolytic|lcl|cTer
QLQGKQVSGEVVQKVLQELIQSVAKP
>lcl|3633|lcl|HAPPENN&HAPPENN-RR90|lcl|subdataset_1|lcl|non-hemolytic|lcl|cTer
FKKLKKLFKKILKLK
>lcl|2510|lcl|HAPPENN&HAPPENN-RR90|lcl|subdataset_1|lcl|non-hemolytic|lcl|cTer
GKKLLWLLKKG
>lcl|4510|lcl|HAPPENN|lcl|subdataset_1|lcl|non-hemolytic
RIKRFWPVVIRTVV
>lcl|1984|lcl|HAPPENN&HAPPENN-hard|lcl|subdataset_1|lcl|non-hemolytic
NLVSALIEGRKYLKNVLK
>lcl|2176|lcl|HAPPENN|lcl|subdataset_1|lcl|hemolytic|lcl|cTer
GLNALKKVFQKIHEAIKLINNHVQ
>lcl|1107|lcl|HAPPENN|lcl|subdataset_1|lcl|hemolytic|lcl|cTer
ILGKLLSTAAKLLSKL
>lcl|2830|lcl|HAPPENN|lcl|subdataset_1|lcl|hemolytic|lcl|cTer
YKLLKLLLPKLKGLLPKL
>lcl|492|lcl|HAPPENN|lcl|subdataset_1|lcl|non-hemolytic
AKKVFKRLEKLFSKI
>lcl|2606|lcl|HAPPENN&HAPPENN-RR90&HAPPENN-hard|lcl|subdataset_1|lcl|hemolytic|lcl|cTer
ILGPILGLVSNALGGLL
>lcl|3096|lcl|HAPPENN&HAPPENN-RR90&HAPPENN-hard|lcl|subdataset_1|lcl|hemolytic|lcl|cTer
VNWKKVLGKVVKVVK
>lcl|3640|lcl|HAPPENN&HAPPENN-RR90&HAPPENN-hard|lcl|subdataset_1|lcl|hemolytic
KWKLFKKIFKCWRWQWRWKKLGA
>lcl|2268|lcl|HAPPENN&HAPPENN-hard|lcl|subdataset_1|lcl|non-hemolytic
GLPVCGETCVGGTCNTPGCTCSYPVCTRN
>lcl|1429|lcl|HAPPENN|lcl|subdataset_1|lcl|hemolytic|lcl|cTer
INQKKIASIGKEV
>lcl|2226|lcl|HAPPENN|lcl|subdataset_1|lcl|non-hemolytic|lcl|cTer
IHKFWRPGRWFKHI
>lcl|18|lcl|HAPPENN|lcl|subdataset_1|lcl|hemolytic|lcl|cTer
ALWKTLLKKV
>lcl|2133|lcl|HAPPENN&HAPPENN-RR90&HAPPENN-hard|lcl|subdataset_1|lcl|non-hemolytic
FFHHIFNGLVGVGKTIHRLI
>lcl|2610|lcl|HAPPENN|lcl|subdataset_1|lcl|hemolytic
GIPCGESCVWIPCISSAIGCSCKSKVCYRN
>lcl|187|lcl|HAPPENN&HAPPENN-RR90&HAPPENN-hard|lcl|subdataset_1|lcl|hemolytic
PICTRNGLPVCGETCFGGTCNTPGCTCTW
>lcl|4771|lcl|HAPPENN|lcl|subdataset_1|lcl|hemolytic
WWWLRRIW
>lcl|3666|lcl|HAPPENN&HAPPENN-hard|lcl|subdataset_1|lcl|non-hemolytic|lcl|cTer
GVVVRWGRVVVRGVRR
>lcl|4439|lcl|HAPPENN|lcl|subdataset_1|lcl|non-hemolytic|lcl|cTer
ALLHHGYKRKFH
>lcl|4336|lcl|HAPPENN&HAPPENN-RR90&HAPPENN-hard|lcl|subdataset_1|lcl|non-hemolytic|lcl|cTer
GIHDILKYGAPS
>lcl|4822|lcl|HAPPENN&HAPPENN-RR90&HAPPENN-hard|lcl|subdataset_1|lcl|non-hemolytic|lcl|cTer
FPFLKLSLKIPKSAIKSAIKRL
>lcl|2805|lcl|HAPPENN|lcl|subdataset_1|lcl|non-hemolytic|lcl|cTer
RVIEVVQGACRAIRHIPRRIR
>lcl|940|lcl|HAPPENN&HAPPENN-RR90&HAPPENN-hard|lcl|subdataset_1|lcl|hemolytic|lcl|cTer
FVQWFSRFLGRIL
>lcl|540|lcl|HAPPENN&HAPPENN-hard|lcl|subdataset_1|lcl|non-hemolytic|lcl|cTer
LKLKSIVSYAKKVL
>lcl|613|lcl|HAPPENN&HAPPENN-RR90|lcl|subdataset_1|lcl|non-hemolytic
```

```
GWKDWAKKAGGWLKKKGPGMAKAALKAAMQ
>lcl|3944|lcl|HAPPENN&HAPPENN-hard|lcl|subdataset_1|lcl|non-hemolytic
FKHHIFRGIVHVGKTIHRLVTG
>lcl|4673|lcl|HAPPENN|lcl|subdataset_1|lcl|non-hemolytic|lcl|cTer
KKRLKKIFKKPMVIGVTIPF
>lcl|3598|lcl|HAPPENN&HAPPENN-hard|lcl|subdataset_1|lcl|non-hemolytic
RRFRPKVTITIQGSARF
>lcl|1372|lcl|HAPPENN&HAPPENN-RR90&HAPPENN-hard|lcl|subdataset_1|lcl|hemolytic
HALWKNMLKGIGKLAGKAALGAVKKLVGAES
>lcl|153|lcl|HAPPENN|lcl|subdataset_1|lcl|hemolytic
RGGRLCYCRRRFCVCF
>lcl|3211|lcl|HAPPENN&HAPPENN-RR90&HAPPENN-hard|lcl|subdataset_1|lcl|non-hemolytic|lcl|cTer
GIGGALLSVGKSALKGLTKGLAEHF
>lcl|4556|lcl|HAPPENN&HAPPENN-RR90&HAPPENN-hard|lcl|subdataset_1|lcl|hemolytic|lcl|cTer
FLKGIVGMLGKLW
>lcl|2254|lcl|HAPPENN&HAPPENN-RR90|lcl|subdataset_1|lcl|non-hemolytic|lcl|cTer
KKFFRAWWARRFLK
>lcl|2690|lcl|HAPPENN&HAPPENN-hard|lcl|subdataset_1|lcl|non-hemolytic
LNLKGLFKKVASLLT
>lcl|4414|lcl|HAPPENN&HAPPENN-RR90&HAPPENN-hard|lcl|subdataset_1|lcl|hemolytic|lcl|cTer
RWFRIQLQIRRWRNRR
>lcl|1428|lcl|HAPPENN|lcl|subdataset_1|lcl|hemolytic|lcl|cTer
INWKKIASIGKEVL
>lcl|1284|lcl|HAPPENN&HAPPENN-RR90&HAPPENN-hard|lcl|subdataset_1|lcl|hemolytic|lcl|cTer
GLLDFVTGVGKDIFAQLIKQI
>lcl|2369|lcl|HAPPENN|lcl|subdataset_1|lcl|non-hemolytic|lcl|cTer
LLRRLRRLL
>lcl|3563|lcl|HAPPENN&HAPPENN-RR90&HAPPENN-hard|lcl|subdataset_1|lcl|non-hemolytic
GKCNVLCQLKQKLRSIGSGSHIGSVVLPRG
>lcl|4228|lcl|HAPPENN|lcl|subdataset_1|lcl|non-hemolytic|lcl|cTer
IRCRRRFCRI
>lcl|4011|lcl|HAPPENN&HAPPENN-RR90|lcl|subdataset_1|lcl|non-hemolytic|lcl|nTer|lcl|cTer
AERVGAGAPVYL
>lcl|3311|lcl|HAPPENN&HAPPENN-RR90&HAPPENN-hard|lcl|subdataset_1|lcl|hemolytic|lcl|cTer
IKLSKKTKDNLKKVLKGAIKGAIAVAKMV
>lcl|3037|lcl|HAPPENN|lcl|subdataset_1|lcl|hemolytic|lcl|cTer
GKWMSLLKHILK
>lcl|2039|lcl|HAPPENN&HAPPENN-RR90|lcl|subdataset_1|lcl|non-hemolytic
DHNNEIVKIQSLLRANKARDDYK
>lcl|4193|lcl|HAPPENN&HAPPENN-RR90&HAPPENN-hard|lcl|subdataset_1|lcl|non-hemolytic
KKLFKKILKYLAGPAGIGKFLHSAKKDEL
>lcl|674|lcl|HAPPENN|lcl|subdataset_1|lcl|non-hemolytic|lcl|cTer
KKKKKKAAFAAWAAFAA
>lcl|1298|lcl|HAPPENN&HAPPENN-RR90&HAPPENN-hard|lcl|subdataset_1|lcl|hemolytic
GLLSGILGAGKQKVCGLSGLC
>lcl|4715|lcl|HAPPENN|lcl|subdataset_1|lcl|hemolytic|lcl|cTer
FSVTWRWWKWWKG
>lcl|2982|lcl|HAPPENN|lcl|subdataset_1|lcl|hemolytic|lcl|cTer
KLLKRIKTLL
>lcl|1823|lcl|HAPPENN&HAPPENN-RR90|lcl|subdataset_1|lcl|non-hemolytic
GIHHILKYGKPS
>lcl|4484|lcl|HAPPENN&HAPPENN-RR90&HAPPENN-hard|lcl|subdataset_1|lcl|hemolytic|lcl|cTer
INWKALLDAAKKVL
>lcl|1523|lcl|HAPPENN|lcl|subdataset_1|lcl|hemolytic
GFMKYIKPLIPHAVKAIKKLI
>lcl|3942|lcl|HAPPENN|lcl|subdataset_1|lcl|hemolytic
KFHHIFRGIVHVGKTIHRLVTG
>lcl|307|lcl|HAPPENN&HAPPENN-hard|lcl|subdataset_1|lcl|non-hemolytic|lcl|cTer
FLGWLFKWAWK
>lcl|3209|lcl|HAPPENN&HAPPENN-RR90&HAPPENN-hard|lcl|subdataset_1|lcl|non-hemolytic|lcl|cTer
GIGGKILGGLKTALKGAAKELASTYLH
>lcl|682|lcl|HAPPENN&HAPPENN-RR90|lcl|subdataset_1|lcl|non-hemolytic
LRLKKRRWKYRV
>lcl|2234|lcl|HAPPENN&HAPPENN-RR90&HAPPENN-hard|lcl|subdataset_1|lcl|hemolytic|lcl|cTer
```

```
WKRIVRWIKRWLR
>lcl|4382|lcl|HAPPENN&HAPPENN-RR90&HAPPENN-hard|lcl|subdataset_1|lcl|non-hemolytic|lcl|cTer
KLLNLLPGLLAGIF
>lcl|4534|lcl|HAPPENN&HAPPENN-hard|lcl|subdataset_1|lcl|non-hemolytic|lcl|cTer
FFHHAFRGIVHVGKTIHRLVTG
>lcl|3743|lcl|HAPPENN|lcl|subdataset_1|lcl|hemolytic
TIAMRAINNYRWRSKNQNTFLR
>lcl|66|lcl|HAPPENN|lcl|subdataset_1|lcl|hemolytic
FFHHIFRGIVHVAKTIHRLVTG
>lcl|4653|lcl|HAPPENN|lcl|subdataset_1|lcl|non-hemolytic|lcl|cTer
CRRWQWRMKKLGC
>lcl|965|lcl|HAPPENN&HAPPENN-RR90&HAPPENN-hard|lcl|subdataset_1|lcl|hemolytic|lcl|cTer
RWGKWFKKATHVGKHVGKAALTAYL
>lcl|603|lcl|HAPPENN&HAPPENN-hard|lcl|subdataset_1|lcl|non-hemolytic
AWASFFKKAAHVGKHVGKAALTHYL
>lcl|4383|lcl|HAPPENN&HAPPENN-RR90&HAPPENN-hard|lcl|subdataset_1|lcl|non-hemolytic|lcl|cTer
LLNSGVKLGTKLLSGLLN
>lcl|1117|lcl|HAPPENN&HAPPENN-RR90|lcl|subdataset_1|lcl|non-hemolytic
RRLMAAKAESRK
>lcl|4659|lcl|HAPPENN|lcl|subdataset_1|lcl|hemolytic|lcl|cTer
FSFLSRIF
>lcl|1506|lcl|HAPPENN&HAPPENN-RR90&HAPPENN-hard|lcl|subdataset_1|lcl|hemolytic|lcl|cTer
AVDLAKIANKVLSSLF
>lcl|382|lcl|HAPPENN|lcl|subdataset_1|lcl|non-hemolytic
LKLKLKLQ
>lcl|2156|lcl|HAPPENN|lcl|subdataset_1|lcl|non-hemolytic|lcl|cTer
FLKLLKKLA
>lcl|3354|lcl|HAPPENN&HAPPENN-RR90|lcl|subdataset_1|lcl|non-hemolytic|lcl|cTer
IKFEPPLPPKKAH
>lcl|1542|lcl|HAPPENN|lcl|subdataset_1|lcl|hemolytic|lcl|cTer
AKRHHGYKRKFHAKRHHGYKRKFH
>lcl|333|lcl|HAPPENN|lcl|subdataset_1|lcl|hemolytic|lcl|cTer
GLFGVLAKVAAHVVPAIAKHF
>lcl|2468|lcl|HAPPENN&HAPPENN-RR90&HAPPENN-hard|lcl|subdataset_1|lcl|non-hemolytic|lcl|cTer
LKLLGIVKKVLGAI
>lcl|3336|lcl|HAPPENN|lcl|subdataset_1|lcl|non-hemolytic|lcl|cTer
LWGTRSGIQP
>lcl|2778|lcl|HAPPENN&HAPPENN-RR90&HAPPENN-hard|lcl|subdataset_1|lcl|hemolytic
GTLPCGESCVWIPCISSVVGCACKSKVCYKD
>lcl|4215|lcl|HAPPENN&HAPPENN-RR90&HAPPENN-hard|lcl|subdataset_1|lcl|non-hemolytic
SIMSTLKQFGISAIKGAAQNVLGVLSCKIAKTC
>lcl|4408|lcl|HAPPENN&HAPPENN-RR90&HAPPENN-hard|lcl|subdataset_1|lcl|hemolytic|lcl|cTer
KIWFQNRRMKWKK
>lcl|1891|lcl|HAPPENN&HAPPENN-hard|lcl|subdataset_1|lcl|non-hemolytic|lcl|cTer
FLYIVAKLLSGLL
>lcl|4678|lcl|HAPPENN&HAPPENN-RR90|lcl|subdataset_1|lcl|non-hemolytic|lcl|cTer
KKWRKLLKKLKKLL
>lcl|1801|lcl|HAPPENN&HAPPENN-hard|lcl|subdataset_1|lcl|non-hemolytic|lcl|cTer
TRKRLKKIGKVLKWI
>lcl|4681|lcl|HAPPENN|lcl|subdataset_1|lcl|hemolytic
IWSFLIKAATKLLPSLFGGGKKDS
>lcl|2309|lcl|HAPPENN&HAPPENN-RR90|lcl|subdataset_1|lcl|non-hemolytic
DKERPICSNTFRGRKC
>lcl|1431|lcl|HAPPENN&HAPPENN-RR90&HAPPENN-hard|lcl|subdataset_1|lcl|hemolytic|lcl|cTer
INWKKGKEVLKAL
>lcl|312|lcl|HAPPENN&HAPPENN-RR90&HAPPENN-hard|lcl|subdataset_1|lcl|non-hemolytic|lcl|cTer
FAKWAFKWAKK
>lcl|4391|lcl|HAPPENN&HAPPENN-RR90&HAPPENN-hard|lcl|subdataset_1|lcl|non-hemolytic|lcl|cTer
PPHKKKLAVYPVFLFYLFLSWFSLIV
>lcl|4200|lcl|HAPPENN&HAPPENN-RR90&HAPPENN-hard|lcl|subdataset_1|lcl|non-hemolytic|lcl|cTer
AGYLLPKINLKPLAKLPKKIL
>lcl|31|lcl|HAPPENN|lcl|subdataset_1|lcl|hemolytic|lcl|cTer
RRWCFRVCYRGFCYRKCR
>lcl|4506|lcl|HAPPENN|lcl|subdataset_1|lcl|non-hemolytic
```

```
RIKRFWPVVIRTVVAGY
>lcl|4186|lcl|HAPPENN&HAPPENN-hard|lcl|subdataset_1|lcl|non-hemolytic
KKLFKKILKYLAGPAKFLHSAKKDEL
>lcl|2530|lcl|HAPPENN&HAPPENN-RR90|lcl|subdataset_1|lcl|non-hemolytic|lcl|cTer
AARIILRWFRR
>lcl|3759|lcl|HAPPENN&HAPPENN-RR90&HAPPENN-hard|lcl|subdataset_1|lcl|non-hemolytic
FLGALFKKASK
>lcl|4022|lcl|HAPPENN&HAPPENN-RR90|lcl|subdataset_1|lcl|non-hemolytic|lcl|nTer|lcl|cTer
FAEPLPSEEEGESYSKEPPEMEKRYGGFM
>lcl|2694|lcl|HAPPENN|lcl|subdataset_1|lcl|hemolytic|lcl|cTer
AIGSILGALAKGLPTLISWIKNR
>lcl|2546|lcl|HAPPENN&HAPPENN-RR90&HAPPENN-hard|lcl|subdataset_1|lcl|non-hemolytic|lcl|cTer
KWKLKKHIGIGKHFLSAKKF
>lcl|918|lcl|HAPPENN&HAPPENN-RR90|lcl|subdataset_1|lcl|non-hemolytic|lcl|cTer
KSLRRVWRSWR
>lcl|4247|lcl|HAPPENN|lcl|subdataset_1|lcl|non-hemolytic|lcl|cTer
KLLKWWKKLL
>lcl|3357|lcl|HAPPENN&HAPPENN-RR90|lcl|subdataset_1|lcl|non-hemolytic|lcl|cTer
KKAAAAAAAAAAAAWAAAAAAKKKK
>lcl|3478|lcl|HAPPENN|lcl|subdataset_1|lcl|non-hemolytic
VDIHVWAGV
>lcl|1526|lcl|HAPPENN&HAPPENN-RR90&HAPPENN-hard|lcl|subdataset_1|lcl|non-hemolytic|lcl|cTer
GLWSTIKQKGKEAAIAAAKAAGQAALGAL
>lcl|395|lcl|HAPPENN|lcl|subdataset_1|lcl|hemolytic|lcl|cTer
VRRFPWWWPFLRR
>lcl|2070|lcl|HAPPENN&HAPPENN-RR90|lcl|subdataset_1|lcl|non-hemolytic
FFQELKEGWKNIKKA
>lcl|2557|lcl|HAPPENN|lcl|subdataset_1|lcl|non-hemolytic
KRIVQRIKDFLR
>lcl|2917|lcl|HAPPENN|lcl|subdataset_1|lcl|non-hemolytic
LLKKLFKKKQ
>lcl|2978|lcl|HAPPENN|lcl|subdataset_1|lcl|hemolytic|lcl|cTer
GLLKRIKFLL
>lcl|3038|lcl|HAPPENN&HAPPENN-RR90&HAPPENN-hard|lcl|subdataset_1|lcl|hemolytic|lcl|cTer
GKWMSLLKKILK
>lcl|1086|lcl|HAPPENN&HAPPENN-RR90&HAPPENN-hard|lcl|subdataset_1|lcl|hemolytic|lcl|cTer
GMATAAGTTLGKLAKFVIGAV
>lcl|60|lcl|HAPPENN&HAPPENN-hard|lcl|subdataset_1|lcl|non-hemolytic|lcl|nTer|lcl|cTer
KWKSFLKTFKSAKKTVLHTLLKAISS
>lcl|3547|lcl|HAPPENN&HAPPENN-RR90&HAPPENN-hard|lcl|subdataset_1|lcl|hemolytic|lcl|cTer
KLGFENFLVKALKTVMHVPTSPLL
>lcl|11|lcl|HAPPENN|lcl|subdataset_1|lcl|hemolytic
TLLKKVLKAAAKAALNAVLVGANA
>lcl|3330|lcl|HAPPENN&HAPPENN-RR90&HAPPENN-hard|lcl|subdataset_1|lcl|hemolytic|lcl|cTer
FVKLKKILNIILSIFKK
>lcl|3134|lcl|HAPPENN&HAPPENN-RR90|lcl|subdataset_1|lcl|non-hemolytic
KKKKPLFGLFFGLF
>lcl|3375|lcl|HAPPENN&HAPPENN-RR90&HAPPENN-hard|lcl|subdataset_1|lcl|hemolytic|lcl|cTer
ILPWKAPWWPWRR
>lcl|1630|lcl|HAPPENN|lcl|subdataset_1|lcl|hemolytic
FLPLLAGLAANFLPTIICKIARKC
>lcl|2682|lcl|HAPPENN&HAPPENN-RR90|lcl|subdataset_1|lcl|non-hemolytic|lcl|cTer
KISKKIMRTFLRRISKDILTGKK
>lcl|3810|lcl|HAPPENN|lcl|subdataset_1|lcl|hemolytic|lcl|cTer
KLKLLLLKLK
>lcl|1519|lcl|HAPPENN|lcl|subdataset_1|lcl|hemolytic|lcl|cTer
GFMKYIKPLIPHAVKAIKKLI
>lcl|2017|lcl|HAPPENN&HAPPENN-RR90|lcl|subdataset_1|lcl|non-hemolytic|lcl|cTer
GSLHGFMYKYLKTLVLRLY
>lcl|4278|lcl|HAPPENN&HAPPENN-RR90|lcl|subdataset_1|lcl|non-hemolytic
DNRMVNHFVQEFKRKHKK
>lcl|1521|lcl|HAPPENN|lcl|subdataset_1|lcl|hemolytic
GFMKYIGPLIPHAVKAISKLI
>lcl|576|lcl|HAPPENN&HAPPENN-RR90&HAPPENN-hard|lcl|subdataset_1|lcl|non-hemolytic
FGPVIGLLSGILKSLL
```

```
>lcl|3143|lcl|HAPPENN&HAPPENN-RR90&HAPPENN-hard|lcl|subdataset_1|lcl|non-hemolytic
KNNFCQVLYVWLLRLGKQCFVKFSKDVET
>lcl|4201|lcl|HAPPENN&HAPPENN-RR90&HAPPENN-hard|lcl|subdataset_1|lcl|non-hemolytic|lcl|cTer
FLPVLGKVIKLVGGLL
>lcl|1178|lcl|HAPPENN&HAPPENN-RR90&HAPPENN-hard|lcl|subdataset_1|lcl|hemolytic
GFGSLFKFLAKKVAKTVAKQAAKQGAKYVANKHME
>lcl|4061|lcl|HAPPENN&HAPPENN-RR90&HAPPENN-hard|lcl|subdataset_1|lcl|hemolytic|lcl|cTer
IAAERRYATIYQARLWAF
>lcl|1817|lcl|HAPPENN&HAPPENN-RR90&HAPPENN-hard|lcl|subdataset_1|lcl|non-hemolytic|lcl|cTer
SLLSLIRLLIT
>lcl|2837|lcl|HAPPENN&HAPPENN-RR90&HAPPENN-hard|lcl|subdataset_1|lcl|hemolytic
FFGTALKIAANVLPTAICKILKKC
>lcl|1359|lcl|HAPPENN|lcl|subdataset_1|lcl|hemolytic
LVRGCWTKSYPPKPCFVR
>lcl|2670|lcl|HAPPENN&HAPPENN-hard|lcl|subdataset_1|lcl|non-hemolytic
GAFGDLLKGVAKEAGMKLLNMAQCKLSGKC
>lcl|1581|lcl|HAPPENN&HAPPENN-RR90|lcl|subdataset_1|lcl|non-hemolytic|lcl|cTer
KKAGKIAKKAGKIA
>lcl|4348|lcl|HAPPENN&HAPPENN-RR90|lcl|subdataset_1|lcl|non-hemolytic|lcl|cTer
KWKLFKKIGIGKFLKKK
>lcl|4091|lcl|HAPPENN&HAPPENN-RR90|lcl|subdataset_1|lcl|non-hemolytic
SAVGRHLRRFGLRKHRKH
>lcl|10|lcl|HAPPENN|lcl|subdataset_1|lcl|hemolytic
TLLKKVLKAAAK
>lcl|1934|lcl|HAPPENN&HAPPENN-hard|lcl|subdataset_1|lcl|non-hemolytic|lcl|cTer
RIGSILGRLAKGLPTLRSWIKNR
>lcl|2935|lcl|HAPPENN|lcl|subdataset_1|lcl|non-hemolytic
KLKKKFKKLQ
>lcl|3098|lcl|HAPPENN|lcl|subdataset_1|lcl|hemolytic|lcl|cTer
VNWKKVLAKIIKVVK
>lcl|1481|lcl|HAPPENN|lcl|subdataset_1|lcl|hemolytic
CGESCVYIPCLTSAVGCSCKSKVCYRNGIP
>lcl|1892|lcl|HAPPENN&HAPPENN-RR90&HAPPENN-hard|lcl|subdataset_1|lcl|hemolytic|lcl|cTer
FLPIVAKLLSGLLGRKKRRQRRR
>lcl|4692|lcl|HAPPENN&HAPPENN-RR90|lcl|subdataset_1|lcl|non-hemolytic|lcl|cTer
IRIKIRIKIRIK
>lcl|717|lcl|HAPPENN|lcl|subdataset_1|lcl|non-hemolytic|lcl|cTer
GLPALISWSKRKRQQ
>lcl|3424|lcl|HAPPENN|lcl|subdataset_1|lcl|non-hemolytic|lcl|cTer
KKIMRTFLRRISKKILTGKK
>lcl|3939|lcl|HAPPENN|lcl|subdataset_1|lcl|hemolytic
AFHHIFRGIVHVGKTIHRLVTG
>lcl|470|lcl|HAPPENN&HAPPENN-RR90&HAPPENN-hard|lcl|subdataset_1|lcl|non-hemolytic
EWGRRCCGWGPGRRYCVRWC
>lcl|3240|lcl|HAPPENN|lcl|subdataset_1|lcl|non-hemolytic|lcl|cTer
PEEMNKYLTALRHYINLVTRQRY
>lcl|1702|lcl|HAPPENN|lcl|subdataset_1|lcl|non-hemolytic
KRFKKFFKKLKNSVKKRAKKFFKK
>lcl|2033|lcl|HAPPENN&HAPPENN-hard|lcl|subdataset_1|lcl|non-hemolytic|lcl|cTer
FLRFAGSVIHGAGHLVHHIGVAL
>lcl|4426|lcl|HAPPENN&HAPPENN-RR90|lcl|subdataset_1|lcl|non-hemolytic|lcl|cTer
GKLWGLKGKLWGLKGKLWGLK
>lcl|1863|lcl|HAPPENN&HAPPENN-hard|lcl|subdataset_1|lcl|non-hemolytic|lcl|cTer
LKKLLKKL
>lcl|823|lcl|HAPPENN|lcl|subdataset_1|lcl|hemolytic
FVKLKKILNIILSIFKK
>lcl|1516|lcl|HAPPENN|lcl|subdataset_1|lcl|hemolytic|lcl|cTer
GFMKYIGPLIPHAVKAISKLI
>lcl|2709|lcl|HAPPENN&HAPPENN-RR90&HAPPENN-hard|lcl|subdataset_1|lcl|hemolytic|lcl|cTer
IKWKKLLRAAKRIL
>lcl|2410|lcl|HAPPENN&HAPPENN-RR90|lcl|subdataset_1|lcl|non-hemolytic
GSTSFHLIYNKWFAVKRRRKR
>lcl|235|lcl|HAPPENN|lcl|subdataset_1|lcl|hemolytic
GLFGKLQKKFGRKAISYAVKKARGKH
>lcl|4825|lcl|HAPPENN&HAPPENN-RR90&HAPPENN-hard|lcl|subdataset_1|lcl|non-hemolytic|lcl|cTer
FRIRVRVFKRIVQRIKDFLR
>lcl|4667|lcl|HAPPENN&HAPPENN-RR90&HAPPENN-hard|lcl|subdataset_1|lcl|hemolytic
```

```
KWKLFKKIFKRIVQRIKDFLRN
>lcl|3074|lcl|HAPPENN|lcl|subdataset_1|lcl|hemolytic|lcl|cTer
VNWKKILGKIIKVAK
>lcl|3879|lcl|HAPPENN&HAPPENN-hard|lcl|subdataset_1|lcl|non-hemolytic|lcl|cTer
KRRLIARILRLAIRALVKKR
>lcl|3453|lcl|HAPPENN|lcl|subdataset_1|lcl|non-hemolytic
RIIDLLWRVRRPQKPKFVTVAVR
>lcl|4283|lcl|HAPPENN&HAPPENN-hard|lcl|subdataset_1|lcl|non-hemolytic|lcl|cTer
GVFDIIKDAGKQLVAHAMGKIAEKV
>lcl|3185|lcl|HAPPENN&HAPPENN-RR90&HAPPENN-hard|lcl|subdataset_1|lcl|non-hemolytic
ALYKKIIKKLLESAKKLG
>lcl|3137|lcl|HAPPENN&HAPPENN-RR90|lcl|subdataset_1|lcl|non-hemolytic
EMLKKKKEVKMERKT
>lcl|2271|lcl|HAPPENN|lcl|subdataset_1|lcl|non-hemolytic
NIQPPCRCC
>lcl|1792|lcl|HAPPENN|lcl|subdataset_1|lcl|hemolytic|lcl|cTer
WWWLRRIW
>lcl|1998|lcl|HAPPENN&HAPPENN-RR90&HAPPENN-hard|lcl|subdataset_1|lcl|hemolytic|lcl|cTer
KIGKALGKALKALGKALGKA
>lcl|3876|lcl|HAPPENN&HAPPENN-RR90|lcl|subdataset_1|lcl|non-hemolytic|lcl|cTer
LRFARRALRRLF
>lcl|3559|lcl|HAPPENN&HAPPENN-RR90|lcl|subdataset_1|lcl|non-hemolytic
GFREKHFQRFVKYAVPESTLR
>lcl|2876|lcl|HAPPENN|lcl|subdataset_1|lcl|non-hemolytic
KLKKLFKKLQ
>lcl|2689|lcl|HAPPENN&HAPPENN-RR90&HAPPENN-hard|lcl|subdataset_1|lcl|non-hemolytic
LNLKGLIKKVASLLN
>lcl|193|lcl|HAPPENN&HAPPENN-RR90&HAPPENN-hard|lcl|subdataset_1|lcl|non-hemolytic|lcl|nTer|lcl|cTer
GIGKFIHAVKKWGKTFIGEIAKS
>lcl|4604|lcl|HAPPENN|lcl|subdataset_1|lcl|non-hemolytic|lcl|cTer
SGKLWWRRKK
>lcl|4703|lcl|HAPPENN&HAPPENN-RR90&HAPPENN-hard|lcl|subdataset_1|lcl|non-hemolytic
GIKHILFMAKTKLPRATCTAEIKENCDRKK
>lcl|945|lcl|HAPPENN|lcl|subdataset_1|lcl|hemolytic|lcl|cTer
FLALIGRVLSGIL
>lcl|3027|lcl|HAPPENN|lcl|subdataset_1|lcl|hemolytic|lcl|cTer
KMWSKILGHLIR
>lcl|563|lcl|HAPPENN&HAPPENN-RR90&HAPPENN-hard|lcl|subdataset_1|lcl|hemolytic|lcl|cTer
FFRRFFRRFFRRFFRR
>lcl|594|lcl|HAPPENN|lcl|subdataset_1|lcl|hemolytic|lcl|cTer
IKLSPETKDNLKKVLKGAIKGAIAVAKMV
>lcl|3978|lcl|HAPPENN&HAPPENN-RR90&HAPPENN-hard|lcl|subdataset_1|lcl|hemolytic
WSPQEEDRIIEGGI
>lcl|4428|lcl|HAPPENN&HAPPENN-hard|lcl|subdataset_1|lcl|non-hemolytic|lcl|cTer
AKLWLKA
>lcl|1559|lcl|HAPPENN&HAPPENN-RR90|lcl|subdataset_1|lcl|non-hemolytic
KARQWQWKIRRTWPIFAIRR
>lcl|2655|lcl|HAPPENN|lcl|subdataset_1|lcl|hemolytic
FLPIVAGLAANFLPKIVCKITKKC
>lcl|4334|lcl|HAPPENN&HAPPENN-RR90|lcl|subdataset_1|lcl|non-hemolytic|lcl|cTer
GIHDILKAGKPS
>lcl|3458|lcl|HAPPENN|lcl|subdataset_1|lcl|hemolytic|lcl|cTer
GLVGTLLGHIGKAILG
>lcl|4598|lcl|HAPPENN|lcl|subdataset_1|lcl|non-hemolytic
SGKLWWRRKK
>lcl|1401|lcl|HAPPENN|lcl|subdataset_1|lcl|hemolytic|lcl|cTer
IDWSKIFEKVKNLV
>lcl|3100|lcl|HAPPENN&HAPPENN-RR90&HAPPENN-hard|lcl|subdataset_1|lcl|hemolytic|lcl|cTer
GTGLPMSERRKIMLMMR
>lcl|1273|lcl|HAPPENN|lcl|subdataset_1|lcl|hemolytic|lcl|cTer
GFLSILKKVLGKVMAHMK
>lcl|3262|lcl|HAPPENN&HAPPENN-RR90&HAPPENN-hard|lcl|subdataset_2|lcl|hemolytic
GLMDTVKNAAKNLAGQLLDRLKCKITGC
>lcl|356|lcl|HAPPENN&HAPPENN-RR90&HAPPENN-hard|lcl|subdataset_2|lcl|hemolytic|lcl|nTer|lcl|cTer
FRKLFRVYSNFLRGKLKL
>lcl|2164|lcl|HAPPENN&HAPPENN-hard|lcl|subdataset_2|lcl|non-hemolytic|lcl|cTer
KWKVFKKIEKMGRNIRNGIVK
>lcl|4457|lcl|HAPPENN&HAPPENN-RR90|lcl|subdataset_2|lcl|non-hemolytic|lcl|cTer
DIGKYCGYAHALN
```

```
>lcl|4612|lcl|HAPPENN|lcl|subdataset_2|lcl|hemolytic|lcl|cTer
WLLKKLLKKW
>lcl|1549|lcl|HAPPENN|lcl|subdataset_2|lcl|non-hemolytic|lcl|cTer
RKKTRKR
>lcl|3084|lcl|HAPPENN|lcl|subdataset_2|lcl|hemolytic
VNWKKILGKIIKVVK
>lcl|4664|lcl|HAPPENN&HAPPENN-RR90&HAPPENN-hard|lcl|subdataset_2|lcl|hemolytic
INLKILARLAKKIL
>lcl|711|lcl|HAPPENN&HAPPENN-hard|lcl|subdataset_2|lcl|non-hemolytic|lcl|cTer
GLPLLISWIKRKRQQ
>lcl|3915|lcl|HAPPENN&HAPPENN-RR90&HAPPENN-hard|lcl|subdataset_2|lcl|hemolytic|lcl|cTer
FPVTWRTKWWKG
>lcl|47|lcl|HAPPENN&HAPPENN-RR90&HAPPENN-hard|lcl|subdataset_2|lcl|hemolytic
FLPIIAGAAAKVVQKIFCAISKKC
>lcl|3928|lcl|HAPPENN&HAPPENN-RR90&HAPPENN-hard|lcl|subdataset_2|lcl|non-hemolytic|lcl|cTer
LLPIVGNLLKSLLGWKRKRFG
>lcl|1600|lcl|HAPPENN&HAPPENN-RR90|lcl|subdataset_2|lcl|non-hemolytic|lcl|cTer
KIAGKIAKIAGKIAKIAGK
>lcl|2981|lcl|HAPPENN|lcl|subdataset_2|lcl|non-hemolytic|lcl|cTer
GLLKRIKKLL
>lcl|2242|lcl|HAPPENN&HAPPENN-RR90&HAPPENN-hard|lcl|subdataset_2|lcl|non-hemolytic|lcl|cTer
KRFIKWYKAWNKKWRKY
>lcl|305|lcl|HAPPENN&HAPPENN-RR90&HAPPENN-hard|lcl|subdataset_2|lcl|non-hemolytic
MESQSNAVQLMLGFGTFVLMLLGLVVELIKNSNKK
>lcl|1394|lcl|HAPPENN&HAPPENN-RR90&HAPPENN-hard|lcl|subdataset_2|lcl|hemolytic|lcl|cTer
FLSIIAKVLGSLF
>lcl|4815|lcl|HAPPENN&HAPPENN-RR90&HAPPENN-hard|lcl|subdataset_2|lcl|hemolytic|lcl|cTer
ISQSDAILSAIWSGIKSLF
>lcl|2229|lcl|HAPPENN&HAPPENN-RR90|lcl|subdataset_2|lcl|non-hemolytic|lcl|cTer
IHFKWRRWKFHI
>lcl|41|lcl|HAPPENN&HAPPENN-RR90&HAPPENN-hard|lcl|subdataset_2|lcl|non-hemolytic|lcl|cTer
GLLDTLKGAAKNVVGSLASKVMEKL
>lcl|376|lcl|HAPPENN&HAPPENN-RR90&HAPPENN-hard|lcl|subdataset_2|lcl|hemolytic
GLLDTFKNLALALNAAKSAGVLNSLSCKLSKTC
>lcl|1069|lcl|HAPPENN|lcl|subdataset_2|lcl|non-hemolytic|lcl|cTer
GRFKRFRKKFKKLFKKLS
>lcl|2199|lcl|HAPPENN&HAPPENN-RR90&HAPPENN-hard|lcl|subdataset_2|lcl|non-hemolytic
IGLRGLGRKIALIHKKYG
>lcl|4179|lcl|HAPPENN&HAPPENN-RR90&HAPPENN-hard|lcl|subdataset_2|lcl|hemolytic
KKLFKKILKYLGIGAVLKVLTTGLKDEL
>lcl|2379|lcl|HAPPENN&HAPPENN-RR90|lcl|subdataset_2|lcl|non-hemolytic|lcl|cTer
RRFRFFFRFRR
>lcl|97|lcl|HAPPENN&HAPPENN-RR90&HAPPENN-hard|lcl|subdataset_2|lcl|non-hemolytic|lcl|cTer
KKKKKKALFAAWAAFLA
>lcl|3955|lcl|HAPPENN&HAPPENN-RR90&HAPPENN-hard|lcl|subdataset_2|lcl|non-hemolytic
KFHHIFRGIKHVGKTIHRLVTG
>lcl|3968|lcl|HAPPENN&HAPPENN-RR90&HAPPENN-hard|lcl|subdataset_2|lcl|non-hemolytic|lcl|cTer
GVVDILKGAAKDIAGHLASKVMAKL
>lcl|3856|lcl|HAPPENN&HAPPENN-RR90|lcl|subdataset_2|lcl|non-hemolytic|lcl|cTer
RKKKLKFFKRLF
>lcl|1729|lcl|HAPPENN|lcl|subdataset_2|lcl|non-hemolytic|lcl|nTer|lcl|cTer
KWKSFLKTFKSAKKTGLHTALKAISS
>lcl|1678|lcl|HAPPENN&HAPPENN-RR90&HAPPENN-hard|lcl|subdataset_2|lcl|non-hemolytic|lcl|cTer
FRRFFKWPRRPFKFF
>lcl|3254|lcl|HAPPENN&HAPPENN-RR90&HAPPENN-hard|lcl|subdataset_2|lcl|hemolytic
FLPAIVGAAAKFLPKIFCAISKKC
>lcl|902|lcl|HAPPENN&HAPPENN-RR90&HAPPENN-hard|lcl|subdataset_2|lcl|non-hemolytic|lcl|cTer
GLPRKILCAIAKKKGKCKGPLKLVCKC
>lcl|1074|lcl|HAPPENN&HAPPENN-RR90|lcl|subdataset_2|lcl|non-hemolytic|lcl|cTer
WCPPMIPLCSRF
>lcl|4117|lcl|HAPPENN&HAPPENN-RR90&HAPPENN-hard|lcl|subdataset_2|lcl|hemolytic
SFKKFWGGVKAIFKGARKGWK
>lcl|4118|lcl|HAPPENN|lcl|subdataset_2|lcl|hemolytic
```

```
FFKKFWGGVKAIFKGARKGWK
>lcl|2503|lcl|HAPPENN&HAPPENN-RR90&HAPPENN-hard|lcl|subdataset_2|lcl|non-hemolytic|lcl|cTer
INWKGIAAMAKKLA
>lcl|1593|lcl|HAPPENN|lcl|subdataset_2|lcl|hemolytic|lcl|cTer
KIAGKIASIAGKIAKIAGKIAKIA
>lcl|3069|lcl|HAPPENN|lcl|subdataset_2|lcl|hemolytic|lcl|cTer
VNWKKVLGKIIKVAK
>lcl|2970|lcl|HAPPENN|lcl|subdataset_2|lcl|hemolytic|lcl|cTer
GLLKIIKTLL
>lcl|4578|lcl|HAPPENN|lcl|subdataset_2|lcl|non-hemolytic
SKVWRHW
>lcl|704|lcl|HAPPENN&HAPPENN-RR90&HAPPENN-hard|lcl|subdataset_2|lcl|hemolytic
IFGAIWNGIKSLF
>lcl|1493|lcl|HAPPENN&HAPPENN-RR90&HAPPENN-hard|lcl|subdataset_2|lcl|hemolytic
VCGETCEGGTCNTPGCSCSWPVCTRNGLP
>lcl|4459|lcl|HAPPENN&HAPPENN-RR90|lcl|subdataset_2|lcl|non-hemolytic
TRSSRAGLQWPVGRVHRLLRK
>lcl|4000|lcl|HAPPENN|lcl|subdataset_2|lcl|hemolytic|lcl|nTer|lcl|cTer
GFFALIPKIISSPLFKTLLSAVGSALS
>lcl|3095|lcl|HAPPENN|lcl|subdataset_2|lcl|hemolytic|lcl|cTer
VYWKKILGKIIKVVK
>lcl|365|lcl|HAPPENN&HAPPENN-RR90&HAPPENN-hard|lcl|subdataset_2|lcl|non-hemolytic
KQLKKVSAVAKVAMKKGAALLKKMGVK
>lcl|3859|lcl|HAPPENN&HAPPENN-RR90|lcl|subdataset_2|lcl|non-hemolytic|lcl|cTer
RKKKFKIFKRLF
>lcl|3015|lcl|HAPPENN|lcl|subdataset_2|lcl|non-hemolytic|lcl|cTer
KLAGLAKKWAGLAKKLAGLAK
>lcl|206|lcl|HAPPENN|lcl|subdataset_2|lcl|non-hemolytic
ELLKAVRLIK
>lcl|20|lcl|HAPPENN&HAPPENN-RR90&HAPPENN-hard|lcl|subdataset_2|lcl|non-hemolytic
GGTIFDCGETCFLGTCYTPGCSCGNYGFCYGTN
>lcl|887|lcl|HAPPENN|lcl|subdataset_2|lcl|non-hemolytic|lcl|cTer
KWKSFIKKLTSKFLHSAKKF
>lcl|1029|lcl|HAPPENN&HAPPENN-RR90|lcl|subdataset_2|lcl|non-hemolytic
KEKLKLKCKAPKCYNDKLACT
>lcl|2961|lcl|HAPPENN|lcl|subdataset_2|lcl|non-hemolytic|lcl|cTer
GLLKRIKTLA
>lcl|1474|lcl|HAPPENN&HAPPENN-RR90&HAPPENN-hard|lcl|subdataset_2|lcl|hemolytic
CGESCVWIPCLTSAIGCSCKSKVCYRNGIP
>lcl|391|lcl|HAPPENN|lcl|subdataset_2|lcl|hemolytic|lcl|cTer
KWKKLLKKLLKLLKKLL
>lcl|30|lcl|HAPPENN&HAPPENN-RR90|lcl|subdataset_2|lcl|non-hemolytic|lcl|cTer
ILPLKLPLLPWRR
>lcl|22|lcl|HAPPENN&HAPPENN-RR90&HAPPENN-hard|lcl|subdataset_2|lcl|hemolytic
GIPCAESCVWIPCTVTALVGCSCSDKVCYN
>lcl|960|lcl|HAPPENN&HAPPENN-RR90&HAPPENN-hard|lcl|subdataset_2|lcl|hemolytic|lcl|cTer
GKGRWLERIGKAGGIIIGGALDHL
>lcl|4759|lcl|HAPPENN&HAPPENN-RR90&HAPPENN-hard|lcl|subdataset_2|lcl|hemolytic|lcl|cTer
FLGELWNVAKSVF
>lcl|4252|lcl|HAPPENN&HAPPENN-RR90&HAPPENN-hard|lcl|subdataset_2|lcl|hemolytic|lcl|cTer
FLFSLIPSAIAGLVSAIRN
>lcl|425|lcl|HAPPENN&HAPPENN-RR90&HAPPENN-hard|lcl|subdataset_2|lcl|non-hemolytic|lcl|cTer
HFLLTLVNLAKKKL
>lcl|2854|lcl|HAPPENN&HAPPENN-RR90&HAPPENN-hard|lcl|subdataset_2|lcl|non-hemolytic|lcl|cTer
ILRWPWWPWRRK
>lcl|1657|lcl|HAPPENN|lcl|subdataset_2|lcl|hemolytic
IFGLLLHGAIHVGKLIHGLVRRH
>lcl|4079|lcl|HAPPENN&HAPPENN-RR90&HAPPENN-hard|lcl|subdataset_2|lcl|hemolytic|lcl|cTer
KWKLWKKIEKWGQGIGAVLKWLTTWL
>lcl|1620|lcl|HAPPENN|lcl|subdataset_2|lcl|non-hemolytic|lcl|cTer
VGVGGGFGR
>lcl|2482|lcl|HAPPENN|lcl|subdataset_2|lcl|hemolytic|lcl|cTer
GIWKKWIKKWLKVLKNLF
>lcl|2930|lcl|HAPPENN|lcl|subdataset_2|lcl|hemolytic
LLLKKFKKLQ
>lcl|1625|lcl|HAPPENN&HAPPENN-RR90&HAPPENN-hard|lcl|subdataset_2|lcl|hemolytic|lcl|cTer
```

```
WFKKLLKKALRLWKKVL
>lcl|72|lcl|HAPPENN|lcl|subdataset_2|lcl|hemolytic
FFHHIFRPIVHVAKTIHRLVTG
>lcl|1867|lcl|HAPPENN|lcl|subdataset_2|lcl|non-hemolytic|lcl|cTer
IKKIIKKIIK
>lcl|2793|lcl|HAPPENN&HAPPENN-RR90&HAPPENN-hard|lcl|subdataset_2|lcl|hemolytic
SISCGESCAMISFCFTEVIGCSCKNKVCYLN
>lcl|3005|lcl|HAPPENN&HAPPENN-RR90|lcl|subdataset_2|lcl|non-hemolytic|lcl|cTer
KLAKGLAKWAKGLAKLAKGLA
>lcl|2031|lcl|HAPPENN|lcl|subdataset_2|lcl|non-hemolytic
RGRKYYRRKK
>lcl|490|lcl|HAPPENN|lcl|subdataset_2|lcl|non-hemolytic
RLEKLFSKIQNDK
>lcl|2013|lcl|HAPPENN&HAPPENN-RR90&HAPPENN-hard|lcl|subdataset_2|lcl|non-hemolytic|lcl|cTer
KVLSKVHTLLKAVLAL
>lcl|2|lcl|HAPPENN|lcl|subdataset_2|lcl|hemolytic
ALWDTLLKKVLKAAAKAALNAVLVGANA
>lcl|3445|lcl|HAPPENN&HAPPENN-RR90&HAPPENN-hard|lcl|subdataset_2|lcl|hemolytic
GLPVCGETCFGGTCNTPGCSCTWPICTRD
>lcl|733|lcl|HAPPENN&HAPPENN-RR90&HAPPENN-hard|lcl|subdataset_2|lcl|non-hemolytic|lcl|nTer|lcl|cTer
SVDMVMKGLKIWPL
>lcl|121|lcl|HAPPENN|lcl|subdataset_2|lcl|non-hemolytic
LCYARRRFAVCV
>lcl|2376|lcl|HAPPENN|lcl|subdataset_2|lcl|non-hemolytic|lcl|cTer
WWRRWRRWW
>lcl|2306|lcl|HAPPENN|lcl|subdataset_2|lcl|non-hemolytic
ALQGAKERAHQQ
>lcl|1773|lcl|HAPPENN&HAPPENN-RR90&HAPPENN-hard|lcl|subdataset_2|lcl|hemolytic|lcl|cTer
FLGAIAQALTSLLGKL
>lcl|2949|lcl|HAPPENN|lcl|subdataset_2|lcl|non-hemolytic
GILDTLKQFAKGVGKDLVKGAAQG
>lcl|870|lcl|HAPPENN&HAPPENN-RR90&HAPPENN-hard|lcl|subdataset_2|lcl|hemolytic
FIKRIARLLRKIF
>lcl|2012|lcl|HAPPENN&HAPPENN-RR90|lcl|subdataset_2|lcl|non-hemolytic|lcl|cTer
GSLHGFMYKYLKNMVLTLF
>lcl|2934|lcl|HAPPENN|lcl|subdataset_2|lcl|non-hemolytic
LLKLKFKKLQ
>lcl|4424|lcl|HAPPENN&HAPPENN-RR90|lcl|subdataset_2|lcl|non-hemolytic|lcl|cTer
GKLWLKGGKLWLKGGKLWLKG
>lcl|1820|lcl|HAPPENN&HAPPENN-RR90&HAPPENN-hard|lcl|subdataset_2|lcl|non-hemolytic|lcl|cTer
LLPWKWPWWKWRR
>lcl|287|lcl|HAPPENN&HAPPENN-RR90&HAPPENN-hard|lcl|subdataset_2|lcl|hemolytic
KNLRRIIRKGIHIIKKYF
>lcl|289|lcl|HAPPENN|lcl|subdataset_2|lcl|hemolytic
PAICQRATATLGTVGSNTSGTTEIEACILL
>lcl|4030|lcl|HAPPENN&HAPPENN-RR90|lcl|subdataset_2|lcl|non-hemolytic
GFCWYVCVYRNGVRVCYRRCN
>lcl|488|lcl|HAPPENN|lcl|subdataset_2|lcl|non-hemolytic
KVFKRLEKLFSKIQNDK
>lcl|1534|lcl|HAPPENN|lcl|subdataset_2|lcl|non-hemolytic|lcl|cTer
FGVLAKVAAHVVPAIAEHF
>lcl|3371|lcl|HAPPENN&HAPPENN-RR90&HAPPENN-hard|lcl|subdataset_2|lcl|hemolytic|lcl|cTer
IAPWKWPWWPWRR
>lcl|2532|lcl|HAPPENN&HAPPENN-RR90|lcl|subdataset_2|lcl|non-hemolytic|lcl|cTer
AARIILRDRFR
>lcl|2067|lcl|HAPPENN|lcl|subdataset_2|lcl|hemolytic|lcl|cTer
LNLKALLAVAKKIL
>lcl|1146|lcl|HAPPENN&HAPPENN-RR90&HAPPENN-hard|lcl|subdataset_2|lcl|hemolytic
KFTIVFPHNQKGNWKNVPSNYHYCP
>lcl|1056|lcl|HAPPENN|lcl|subdataset_2|lcl|non-hemolytic|lcl|cTer
KKWLKKWLK
>lcl|3873|lcl|HAPPENN&HAPPENN-RR90&HAPPENN-hard|lcl|subdataset_2|lcl|hemolytic|lcl|cTer
LRIIRRILRRLI
>lcl|3086|lcl|HAPPENN|lcl|subdataset_2|lcl|hemolytic|lcl|cTer
ILGKIIKVVK
>lcl|4704|lcl|HAPPENN|lcl|subdataset_2|lcl|non-hemolytic|lcl|nTer|lcl|cTer
KARRAVRWI
```

```
>lcl|1495|lcl|HAPPENN|lcl|subdataset_2|lcl|hemolytic|lcl|cTer
LLGMIPLAISAISALSKL
>lcl|2382|lcl|HAPPENN&HAPPENN-RR90|lcl|subdataset_2|lcl|non-hemolytic|lcl|cTer
RIIRRIRRIIR
>lcl|818|lcl|HAPPENN&HAPPENN-hard|lcl|subdataset_2|lcl|non-hemolytic
FVDLKKIANIINSIFKK
>lcl|727|lcl|HAPPENN&HAPPENN-RR90|lcl|subdataset_2|lcl|non-hemolytic|lcl|nTer|lcl|cTer
CVKVQVKVGSGVKVQVKVC
>lcl|3880|lcl|HAPPENN&HAPPENN-RR90&HAPPENN-hard|lcl|subdataset_2|lcl|hemolytic|lcl|cTer
KRRLILRILRLAIRALVKKR
>lcl|820|lcl|HAPPENN&HAPPENN-hard|lcl|subdataset_2|lcl|non-hemolytic
FVKLKKIANIINSIFKK
>lcl|3348|lcl|HAPPENN&HAPPENN-RR90&HAPPENN-hard|lcl|subdataset_2|lcl|hemolytic
GIIKKIIKKIIKKIIKKI
>lcl|1572|lcl|HAPPENN|lcl|subdataset_2|lcl|hemolytic|lcl|cTer
ALWKTMLKKLGTM
>lcl|2948|lcl|HAPPENN|lcl|subdataset_2|lcl|non-hemolytic
GILDTLKQFAKGVGKDLVKGAAQGV
>lcl|3047|lcl|HAPPENN&HAPPENN-RR90&HAPPENN-hard|lcl|subdataset_2|lcl|hemolytic|lcl|cTer
GKWWSLLKHILK
>lcl|1333|lcl|HAPPENN&HAPPENN-RR90&HAPPENN-hard|lcl|subdataset_2|lcl|hemolytic|lcl|cTer
LNWKAILKHIIK
>lcl|901|lcl|HAPPENN|lcl|subdataset_2|lcl|non-hemolytic
GLPRKILCAIAKKKGKCKGPLKLVCKC
>lcl|724|lcl|HAPPENN&HAPPENN-RR90|lcl|subdataset_2|lcl|non-hemolytic|lcl|nTer|lcl|cTer
CVKVKVKVGSGVKVKVKVC
>lcl|3884|lcl|HAPPENN&HAPPENN-RR90&HAPPENN-hard|lcl|subdataset_2|lcl|hemolytic|lcl|cTer
KRRKLIKILKLIIKLIRKKR
>lcl|1239|lcl|HAPPENN&HAPPENN-RR90&HAPPENN-hard|lcl|subdataset_2|lcl|hemolytic|lcl|cTer
LISWIKRKRQQGIGAVLKVLTTGL
>lcl|708|lcl|HAPPENN|lcl|subdataset_2|lcl|non-hemolytic|lcl|cTer
GSPALISWIKRKRQQ
>lcl|1459|lcl|HAPPENN|lcl|subdataset_2|lcl|non-hemolytic|lcl|cTer
KKFPWWWPFKK
>lcl|1202|lcl|HAPPENN&HAPPENN-RR90&HAPPENN-hard|lcl|subdataset_2|lcl|hemolytic
GVFLDALKKFAKGGMNAVLNPK
>lcl|4102|lcl|HAPPENN&HAPPENN-RR90|lcl|subdataset_2|lcl|non-hemolytic
VQWRIRVAVIRK
>lcl|1078|lcl|HAPPENN&HAPPENN-RR90|lcl|subdataset_2|lcl|non-hemolytic
LGRVDIHVWDGVYIRGR
>lcl|367|lcl|HAPPENN&HAPPENN-RR90&HAPPENN-hard|lcl|subdataset_2|lcl|hemolytic
KFGKIVGKVLKQLKKVSAVAKVAMKKG
>lcl|2544|lcl|HAPPENN&HAPPENN-RR90&HAPPENN-hard|lcl|subdataset_2|lcl|hemolytic|lcl|cTer
GIWDTIKSMGKVFAGKILQNL
>lcl|4194|lcl|HAPPENN&HAPPENN-hard|lcl|subdataset_2|lcl|non-hemolytic
KKLFKKILKYLAVAVVGQATQIAKKDEL
>lcl|2009|lcl|HAPPENN|lcl|subdataset_2|lcl|non-hemolytic|lcl|cTer
GSLHGFMYKYLKNMVLNLF
>lcl|3768|lcl|HAPPENN|lcl|subdataset_2|lcl|non-hemolytic|lcl|cTer
RRWWRWWR
>lcl|1518|lcl|HAPPENN&HAPPENN-RR90&HAPPENN-hard|lcl|subdataset_2|lcl|hemolytic|lcl|cTer
GFMKYIKPLIPHAVKAISKLI
>lcl|1241|lcl|HAPPENN&HAPPENN-RR90&HAPPENN-hard|lcl|subdataset_2|lcl|non-hemolytic
RCLPAGKTCVRGPMRVPCCGSCSQNKCT
>lcl|591|lcl|HAPPENN|lcl|subdataset_2|lcl|hemolytic
FLFSLIPHAIGGLISAFK
>lcl|1002|lcl|HAPPENN|lcl|subdataset_2|lcl|hemolytic|lcl|cTer
GIIKKIIKKIIKKIIKKI
>lcl|131|lcl|HAPPENN&HAPPENN-RR90|lcl|subdataset_2|lcl|non-hemolytic
LCYTFRGRFVCV
>lcl|544|lcl|HAPPENN&HAPPENN-RR90&HAPPENN-hard|lcl|subdataset_2|lcl|non-hemolytic|lcl|cTer
LKAKSIVSWAKKVL
>lcl|2938|lcl|HAPPENN|lcl|subdataset_2|lcl|hemolytic|lcl|cTer
ILPWKWPWWKWRR
>lcl|3668|lcl|HAPPENN&HAPPENN-hard|lcl|subdataset_2|lcl|non-hemolytic
```

```
GALFKVASKVL
>lcl|904|lcl|HAPPENN&HAPPENN-hard|lcl|subdataset_2|lcl|non-hemolytic
GLPRKILAAIAKKKGKCKGPLKLVAKC
>lcl|964|lcl|HAPPENN&HAPPENN-RR90&HAPPENN-hard|lcl|subdataset_2|lcl|hemolytic|lcl|cTer
GWGSIFKHGRHAAKHIGHAAVNHYL
>lcl|3899|lcl|HAPPENN|lcl|subdataset_2|lcl|non-hemolytic
SRWPSPGRPRPFPGRPKPIFRPR
>lcl|2475|lcl|HAPPENN&HAPPENN-RR90|lcl|subdataset_2|lcl|non-hemolytic
LPKPPKPVSKMRMATPLLMQALPM
>lcl|4725|lcl|HAPPENN&HAPPENN-RR90|lcl|subdataset_2|lcl|non-hemolytic|lcl|cTer
FPLTCPTKWWKG
>lcl|2678|lcl|HAPPENN&HAPPENN-RR90&HAPPENN-hard|lcl|subdataset_2|lcl|hemolytic
FLGSLLGLVGKVVPTLFCKISKKC
>lcl|3663|lcl|HAPPENN&HAPPENN-hard|lcl|subdataset_2|lcl|non-hemolytic
FLGALFKVASKVL
>lcl|993|lcl|HAPPENN&HAPPENN-RR90|lcl|subdataset_2|lcl|non-hemolytic
SWLRDLWDWLCELLSDFK
>lcl|3156|lcl|HAPPENN|lcl|subdataset_2|lcl|hemolytic
GVGKFLHSAKKFGQALASEIMKS
>lcl|3807|lcl|HAPPENN|lcl|subdataset_2|lcl|hemolytic|lcl|cTer
RRLFRRILRWL
>lcl|1402|lcl|HAPPENN|lcl|subdataset_2|lcl|hemolytic|lcl|cTer
INWSSIFEKVKNLV
>lcl|4577|lcl|HAPPENN|lcl|subdataset_2|lcl|non-hemolytic
RHWRRFWH
>lcl|3823|lcl|HAPPENN&HAPPENN-hard|lcl|subdataset_2|lcl|non-hemolytic|lcl|cTer
GIGKFLHSAKKPGKAFVGEIMNS
>lcl|3936|lcl|HAPPENN&HAPPENN-RR90&HAPPENN-hard|lcl|subdataset_2|lcl|hemolytic|lcl|cTer
FPVTWRWWKWWKG
>lcl|4635|lcl|HAPPENN&HAPPENN-RR90&HAPPENN-hard|lcl|subdataset_2|lcl|hemolytic|lcl|cTer
GFLDIVKGVGKVALGAVSKLF
>lcl|3990|lcl|HAPPENN&HAPPENN-hard|lcl|subdataset_2|lcl|non-hemolytic|lcl|cTer
GVADILKGAAKDIAGHLASKVMNKL
>lcl|1087|lcl|HAPPENN&HAPPENN-RR90&HAPPENN-hard|lcl|subdataset_2|lcl|hemolytic
GLASTIGSLLGKFAKGGAQAFLQPK
>lcl|2180|lcl|HAPPENN|lcl|subdataset_2|lcl|hemolytic|lcl|cTer
WLNALKKVFQGIHEAIKLIWNWVQ
>lcl|2773|lcl|HAPPENN&HAPPENN-RR90&HAPPENN-hard|lcl|subdataset_2|lcl|hemolytic
GLPVCGETCFGGTCNTPGCSCETWPVCSRN
>lcl|527|lcl|HAPPENN&HAPPENN-RR90&HAPPENN-hard|lcl|subdataset_2|lcl|hemolytic
WEAKLAKALAKALAKHLAKALAKALKACEA
>lcl|4509|lcl|HAPPENN|lcl|subdataset_2|lcl|non-hemolytic
RIKRFWPVVIRTVVA
>lcl|4346|lcl|HAPPENN|lcl|subdataset_2|lcl|non-hemolytic|lcl|cTer
KWKLFKKIGIGKFL
>lcl|3736|lcl|HAPPENN|lcl|subdataset_2|lcl|hemolytic|lcl|nTer|lcl|cTer
RGLRRLGRKIAHGVKKYGPTVLRIIRIAG
>lcl|3243|lcl|HAPPENN&HAPPENN-RR90|lcl|subdataset_2|lcl|non-hemolytic|lcl|cTer
FDWDSVLKGVEGFVRGYF
>lcl|1910|lcl|HAPPENN&HAPPENN-RR90|lcl|subdataset_2|lcl|non-hemolytic
SNGDGTLDAGSTCAPFYARA
>lcl|3812|lcl|HAPPENN&HAPPENN-RR90|lcl|subdataset_2|lcl|non-hemolytic|lcl|cTer
RKRAARLLKRLV
>lcl|2343|lcl|HAPPENN&HAPPENN-RR90&HAPPENN-hard|lcl|subdataset_2|lcl|hemolytic
FIHHIIGGLFHVGKSIHDLIR
>lcl|116|lcl|HAPPENN|lcl|subdataset_2|lcl|hemolytic
RGGRLCYCRRRFCVCV
>lcl|2424|lcl|HAPPENN&HAPPENN-RR90|lcl|subdataset_2|lcl|non-hemolytic|lcl|cTer
IKPIIKPIIKPIIKPIIKPIIKPIIKPI
>lcl|690|lcl|HAPPENN|lcl|subdataset_2|lcl|hemolytic
RRFPWWWPFRR
>lcl|3328|lcl|HAPPENN&HAPPENN-hard|lcl|subdataset_2|lcl|non-hemolytic|lcl|cTer
FVKLKKIANIINSIFKK
>lcl|1321|lcl|HAPPENN|lcl|subdataset_2|lcl|hemolytic|lcl|cTer
IFGAILPLALGALKNLIK
>lcl|2960|lcl|HAPPENN|lcl|subdataset_2|lcl|non-hemolytic|lcl|cTer
GLLKRIKTAL
>lcl|2631|lcl|HAPPENN|lcl|subdataset_2|lcl|hemolytic
GILDSFKGVAKGVAKDLAGKLLDKLKCKITGC
>lcl|2344|lcl|HAPPENN|lcl|subdataset_2|lcl|hemolytic|lcl|cTer
FIHHIIGGLFHVGKSIHDLIR
```

```
>lcl|3822|lcl|HAPPENN&HAPPENN-RR90&HAPPENN-hard|lcl|subdataset_2|lcl|non-hemolytic|lcl|cTer
LRALAKALKHKL
>lcl|4103|lcl|HAPPENN|lcl|subdataset_2|lcl|non-hemolytic
KFFKKLKNSVKKRAKKFFKKPRVIGVSIPF
>lcl|3117|lcl|HAPPENN&HAPPENN-RR90|lcl|subdataset_2|lcl|non-hemolytic
FLPIVENCSLVCWENNQKC
>lcl|4553|lcl|HAPPENN&HAPPENN-RR90&HAPPENN-hard|lcl|subdataset_2|lcl|non-hemolytic|lcl|cTer
FLKGIVGKLGKLF
>lcl|4401|lcl|HAPPENN&HAPPENN-RR90&HAPPENN-hard|lcl|subdataset_2|lcl|hemolytic|lcl|cTer
VKWRWKWKWRWKWKWKV
>lcl|2765|lcl|HAPPENN|lcl|subdataset_2|lcl|hemolytic|lcl|cTer
GLKEIFKAGLGSLVKGIAAHVAS
>lcl|1745|lcl|HAPPENN&HAPPENN-RR90&HAPPENN-hard|lcl|subdataset_2|lcl|non-hemolytic|lcl|cTer
ILGAVWNGVKSLF
>lcl|1255|lcl|HAPPENN&HAPPENN-RR90&HAPPENN-hard|lcl|subdataset_2|lcl|hemolytic
KWKSFLKTFKSPVKTVFYTALKPISS
>lcl|4730|lcl|HAPPENN&HAPPENN-RR90&HAPPENN-hard|lcl|subdataset_2|lcl|non-hemolytic
KWLRWVRRRWW
>lcl|1789|lcl|HAPPENN&HAPPENN-RR90&HAPPENN-hard|lcl|subdataset_2|lcl|hemolytic
WKRRIKIWKKIR
>lcl|2908|lcl|HAPPENN|lcl|subdataset_2|lcl|non-hemolytic
PRPPHPPRPPHPPRPPHPRL
>lcl|3317|lcl|HAPPENN&HAPPENN-RR90|lcl|subdataset_2|lcl|non-hemolytic
AWLDKLKSLGKVVG
>lcl|4361|lcl|HAPPENN&HAPPENN-hard|lcl|subdataset_2|lcl|non-hemolytic
NLCASLRARHTIPQCKKFGRR
>lcl|2691|lcl|HAPPENN&HAPPENN-RR90&HAPPENN-hard|lcl|subdataset_2|lcl|hemolytic|lcl|cTer
FDIMGLIKKVAGAL
>lcl|1584|lcl|HAPPENN|lcl|subdataset_2|lcl|hemolytic|lcl|cTer
KIAGKIAAIAGKIAKIAGKIAK
>lcl|3455|lcl|HAPPENN&HAPPENN-hard|lcl|subdataset_2|lcl|non-hemolytic|lcl|cTer
RWKIFKKIEKMGRNIRDGIVKAGPAIEVLGSAKAI
>lcl|350|lcl|HAPPENN|lcl|subdataset_2|lcl|hemolytic
IISTIGDLVKWIIDTVNKFTKK
>lcl|4708|lcl|HAPPENN&HAPPENN-RR90&HAPPENN-hard|lcl|subdataset_2|lcl|hemolytic|lcl|cTer
GIGRFLHSARRFGRAFVGEIMNS
>lcl|976|lcl|HAPPENN&HAPPENN-RR90&HAPPENN-hard|lcl|subdataset_2|lcl|hemolytic
GWKKWFTKGERLSQRHFA
>lcl|2161|lcl|HAPPENN&HAPPENN-RR90&HAPPENN-hard|lcl|subdataset_2|lcl|hemolytic|lcl|cTer
GFRKIVQKIRDFLRNLV
>lcl|77|lcl|HAPPENN&HAPPENN-RR90|lcl|subdataset_2|lcl|non-hemolytic|lcl|cTer
RILRGVSRRIMRRILTGRR
>lcl|324|lcl|HAPPENN&HAPPENN-RR90&HAPPENN-hard|lcl|subdataset_2|lcl|hemolytic
AWKVFKRLGIGAVLWVLTWG
>lcl|909|lcl|HAPPENN|lcl|subdataset_2|lcl|hemolytic|lcl|cTer
VNWKKILGKIIKVVK
>lcl|4572|lcl|HAPPENN|lcl|subdataset_2|lcl|non-hemolytic
VWRHWRRFWHR
>lcl|4592|lcl|HAPPENN|lcl|subdataset_2|lcl|non-hemolytic|lcl|cTer
MLLKKLLKKM
>lcl|4827|lcl|HAPPENN&HAPPENN-RR90|lcl|subdataset_2|lcl|non-hemolytic|lcl|cTer
FRIRVRVKWKLFKKI
>lcl|678|lcl|HAPPENN&HAPPENN-RR90|lcl|subdataset_2|lcl|non-hemolytic|lcl|cTer
KKKKKKAAWAAWAAWAA
>lcl|2210|lcl|HAPPENN&HAPPENN-RR90&HAPPENN-hard|lcl|subdataset_2|lcl|hemolytic|lcl|cTer
RPAWLKAAFRVMRACV
>lcl|3881|lcl|HAPPENN&HAPPENN-hard|lcl|subdataset_2|lcl|non-hemolytic|lcl|cTer
KRRLILRILRLAIRILVKKR
>lcl|4610|lcl|HAPPENN|lcl|subdataset_2|lcl|non-hemolytic|lcl|cTer
GRKKRRQRRRPWQ
>lcl|4588|lcl|HAPPENN|lcl|subdataset_2|lcl|non-hemolytic|lcl|cTer
ILLKKLLKKI
>lcl|2386|lcl|HAPPENN&HAPPENN-RR90|lcl|subdataset_2|lcl|non-hemolytic
KKWRKWLKALAKK
>lcl|1583|lcl|HAPPENN|lcl|subdataset_2|lcl|non-hemolytic|lcl|cTer
```

```
KIAGKIAKKAGKIAKIA
>lcl|1385|lcl|HAPPENN&HAPPENN-RR90&HAPPENN-hard|lcl|subdataset_2|lcl|hemolytic
PKLLKTFLKWIG
>lcl|2433|lcl|HAPPENN&HAPPENN-RR90|lcl|subdataset_2|lcl|non-hemolytic|lcl|cTer
RWKIFKKAAKKG
>lcl|1271|lcl|HAPPENN|lcl|subdataset_2|lcl|hemolytic|lcl|cTer
GFLSILKKVLKVMAHMK
>lcl|1240|lcl|HAPPENN&HAPPENN-RR90&HAPPENN-hard|lcl|subdataset_2|lcl|non-hemolytic|lcl|cTer
ACGILHDNCVYVPAQNPCCRGLQCRYGKCLVQV
>lcl|1048|lcl|HAPPENN|lcl|subdataset_2|lcl|hemolytic
GLKEIFKAGLGSLVKGIAAHVAS
>lcl|688|lcl|HAPPENN|lcl|subdataset_2|lcl|non-hemolytic
KKFPWWWPFKK
>lcl|1364|lcl|HAPPENN&HAPPENN-RR90&HAPPENN-hard|lcl|subdataset_2|lcl|hemolytic
SLSRYAKLANRLANPKLLETFLSKWIG
>lcl|4044|lcl|HAPPENN&HAPPENN-hard|lcl|subdataset_2|lcl|non-hemolytic|lcl|cTer
KLKKLLKKLLKL
>lcl|3130|lcl|HAPPENN|lcl|subdataset_2|lcl|hemolytic
FLPVLAGLTPSIVPKLVCLLTKKC
>lcl|224|lcl|HAPPENN|lcl|subdataset_2|lcl|non-hemolytic|lcl|cTer
LLKWLKK
>lcl|1814|lcl|HAPPENN&HAPPENN-RR90&HAPPENN-hard|lcl|subdataset_2|lcl|non-hemolytic|lcl|cTer
KRRWPWWPWRLI
>lcl|4389|lcl|HAPPENN|lcl|subdataset_2|lcl|non-hemolytic|lcl|cTer
RKWVWWRNR
>lcl|1276|lcl|HAPPENN|lcl|subdataset_2|lcl|hemolytic|lcl|cTer
LPKVMAHMK
>lcl|1515|lcl|HAPPENN&HAPPENN-RR90&HAPPENN-hard|lcl|subdataset_2|lcl|non-hemolytic|lcl|cTer
GFMKYIGPLIPHKVKAISDLI
>lcl|3510|lcl|HAPPENN&HAPPENN-RR90&HAPPENN-hard|lcl|subdataset_2|lcl|non-hemolytic
INLKAIAALVKKLL
>lcl|3890|lcl|HAPPENN|lcl|subdataset_2|lcl|hemolytic|lcl|cTer
GIWKTIKSMGKVFAGKILQNL
>lcl|3908|lcl|HAPPENN|lcl|subdataset_2|lcl|non-hemolytic
RVVRQWPIGRVVRRVVRRVVRL
>lcl|1757|lcl|HAPPENN|lcl|subdataset_2|lcl|hemolytic
NLLNDALGTVNGLLGRS
>lcl|1141|lcl|HAPPENN|lcl|subdataset_2|lcl|non-hemolytic
MASRAARLAARLARLALRAL
>lcl|3073|lcl|HAPPENN|lcl|subdataset_2|lcl|hemolytic|lcl|cTer
VNWKKVLPKIIKVAK
>lcl|3846|lcl|HAPPENN|lcl|subdataset_2|lcl|hemolytic
GLNALKKVFQGIHEAFKLINNHVQ
>lcl|3325|lcl|HAPPENN&HAPPENN-hard|lcl|subdataset_2|lcl|non-hemolytic|lcl|cTer
FVDLKKIANIINSIFKK
>lcl|516|lcl|HAPPENN&HAPPENN-RR90&HAPPENN-hard|lcl|subdataset_2|lcl|non-hemolytic|lcl|cTer
GLLGPLLKIAKKVGSNLL
>lcl|3529|lcl|HAPPENN&HAPPENN-RR90&HAPPENN-hard|lcl|subdataset_2|lcl|non-hemolytic
GLLLDTVKGAAKNVAGILLNKLKCKMTGDC
>lcl|519|lcl|HAPPENN|lcl|subdataset_2|lcl|hemolytic|lcl|cTer
GLLGPLLKIAAKVGKKLL
>lcl|539|lcl|HAPPENN&HAPPENN-RR90&HAPPENN-hard|lcl|subdataset_2|lcl|non-hemolytic|lcl|cTer
LKLKNIVSWAKKVL
>lcl|4323|lcl|HAPPENN&HAPPENN-RR90&HAPPENN-hard|lcl|subdataset_2|lcl|hemolytic|lcl|cTer
GIGGALLNVGKVALKGLAKGLAEHFAN
>lcl|3222|lcl|HAPPENN&HAPPENN-hard|lcl|subdataset_2|lcl|non-hemolytic|lcl|cTer
ILGPVLGLVSNALGGLL
>lcl|248|lcl|HAPPENN|lcl|subdataset_2|lcl|non-hemolytic
GMWSKIKNAGKAAKAAAKAAGKAALGAVSEAM
>lcl|2004|lcl|HAPPENN&HAPPENN-RR90&HAPPENN-hard|lcl|subdataset_2|lcl|non-hemolytic|lcl|cTer
GKLGPLLKIAAKVGSNLL
>lcl|3414|lcl|HAPPENN&HAPPENN-RR90&HAPPENN-hard|lcl|subdataset_2|lcl|non-hemolytic|lcl|cTer
HFLGTLVNLAKKIK
>lcl|3040|lcl|HAPPENN&HAPPENN-RR90&HAPPENN-hard|lcl|subdataset_2|lcl|hemolytic|lcl|cTer
```

```
GKWMKLLKHILK
>lcl|2405|lcl|HAPPENN&HAPPENN-RR90&HAPPENN-hard|lcl|subdataset_2|lcl|hemolytic
FIKIFHHIFKGNPKFSIIK
>lcl|1901|lcl|HAPPENN|lcl|subdataset_2|lcl|non-hemolytic
GRPNPVPNPKPPHPRL
>lcl|4083|lcl|HAPPENN&HAPPENN-RR90|lcl|subdataset_2|lcl|non-hemolytic
GLAKGIKEAAKALDKAL
>lcl|3238|lcl|HAPPENN&HAPPENN-RR90&HAPPENN-hard|lcl|subdataset_2|lcl|hemolytic
RIGVLLARLPKLFSLFKLMGKKV
>lcl|4453|lcl|HAPPENN|lcl|subdataset_2|lcl|hemolytic|lcl|cTer
FILGKLWKGVKSIF
>lcl|471|lcl|HAPPENN|lcl|subdataset_2|lcl|non-hemolytic
EWGRRCCGWGPGRRYCRRWC
>lcl|3401|lcl|HAPPENN&HAPPENN-RR90&HAPPENN-hard|lcl|subdataset_2|lcl|non-hemolytic|lcl|cTer
RLARIVVIRVAR
>lcl|3411|lcl|HAPPENN&HAPPENN-hard|lcl|subdataset_2|lcl|non-hemolytic|lcl|cTer
HFLGTLVNKAKKIL
>lcl|3843|lcl|HAPPENN&HAPPENN-hard|lcl|subdataset_2|lcl|non-hemolytic
GLNAFKKVFQGIHEAIKLINNHVQ
>lcl|3399|lcl|HAPPENN&HAPPENN-RR90&HAPPENN-hard|lcl|subdataset_2|lcl|non-hemolytic|lcl|cTer
GFKDLLKGAAKALVKTVKF
>lcl|3851|lcl|HAPPENN&HAPPENN-RR90&HAPPENN-hard|lcl|subdataset_2|lcl|non-hemolytic|lcl|cTer
RKKKLKIIKRLI
>lcl|1562|lcl|HAPPENN|lcl|subdataset_2|lcl|non-hemolytic|lcl|cTer
LLKKLLKKC
>lcl|971|lcl|HAPPENN&HAPPENN-RR90&HAPPENN-hard|lcl|subdataset_2|lcl|hemolytic|lcl|cTer
GWRTLLKKAEVKTVGKLALKHYL
>lcl|4294|lcl|HAPPENN&HAPPENN-hard|lcl|subdataset_2|lcl|non-hemolytic|lcl|cTer
FIGALLRPALKLLA
>lcl|4012|lcl|HAPPENN&HAPPENN-hard|lcl|subdataset_2|lcl|non-hemolytic|lcl|cTer
GLLKKIKKLL
>lcl|1535|lcl|HAPPENN|lcl|subdataset_2|lcl|hemolytic
FKIKASKVLDKFGKIVGKVLKQLKKVSAVAKV
>lcl|229|lcl|HAPPENN|lcl|subdataset_2|lcl|non-hemolytic|lcl|cTer
KKLLKWLKKLL
>lcl|188|lcl|HAPPENN&HAPPENN-RR90&HAPPENN-hard|lcl|subdataset_2|lcl|hemolytic
PVCTRNGLPVCGETCVGGTCNTPGCTCSW
>lcl|3772|lcl|HAPPENN|lcl|subdataset_2|lcl|non-hemolytic
KKTWWKTWWTKWSQP
>lcl|4561|lcl|HAPPENN&HAPPENN-RR90|lcl|subdataset_2|lcl|non-hemolytic
ASITHVKNRGKYIYMHLKFRKTNVLI
>lcl|2888|lcl|HAPPENN|lcl|subdataset_2|lcl|non-hemolytic
LKKLKFKLKQ
>lcl|1937|lcl|HAPPENN|lcl|subdataset_2|lcl|non-hemolytic|lcl|cTer
WKKIRVRLSA
>lcl|178|lcl|HAPPENN|lcl|subdataset_2|lcl|hemolytic|lcl|cTer
FFHHIFRGIVHVGKTIHRLVTG
>lcl|3176|lcl|HAPPENN|lcl|subdataset_2|lcl|hemolytic|lcl|cTer
FLFSLIPHAIGGLISAFK
>lcl|2342|lcl|HAPPENN&HAPPENN-RR90|lcl|subdataset_2|lcl|non-hemolytic
YGRKKRRQRRRSISLL
>lcl|1131|lcl|HAPPENN|lcl|subdataset_2|lcl|hemolytic|lcl|cTer
GAGALAKFLAKKVAKTVAKQAAKQGAKYVVNKQME
>lcl|285|lcl|HAPPENN|lcl|subdataset_2|lcl|hemolytic|lcl|cTer
KWKLFKKIGAVLKVL
>lcl|3539|lcl|HAPPENN&HAPPENN-RR90&HAPPENN-hard|lcl|subdataset_2|lcl|hemolytic
FMGTALKIAANVLPAAFCKIFKKC
>lcl|3820|lcl|HAPPENN&HAPPENN-RR90&HAPPENN-hard|lcl|subdataset_2|lcl|hemolytic|lcl|cTer
LLKALKKLLKKLL
>lcl|481|lcl|HAPPENN&HAPPENN-RR90|lcl|subdataset_2|lcl|non-hemolytic
VTLASHLPSDFTPAVHASLDKFLANVSTVLTSKYR
>lcl|1272|lcl|HAPPENN|lcl|subdataset_2|lcl|hemolytic|lcl|cTer
GFLSILKKVLAKVMAHMK
>lcl|980|lcl|HAPPENN|lcl|subdataset_2|lcl|hemolytic
GIRKWFKKAAHVGKEVGKVALNACL
>lcl|1862|lcl|HAPPENN&HAPPENN-RR90|lcl|subdataset_2|lcl|non-hemolytic|lcl|cTer
RICRIVVIRCIR
>lcl|4406|lcl|HAPPENN&HAPPENN-RR90&HAPPENN-hard|lcl|subdataset_2|lcl|hemolytic|lcl|cTer
```

```
RQIRIWFQNRRMRWRR
>lcl|1925|lcl|HAPPENN&HAPPENN-RR90&HAPPENN-hard|lcl|subdataset_2|lcl|hemolytic|lcl|cTer
KWLKKWLKWLKK
>lcl|373|lcl|HAPPENN|lcl|subdataset_2|lcl|hemolytic
GLLSGILGAGKHIVCGLSGLC
>lcl|2356|lcl|HAPPENN|lcl|subdataset_2|lcl|non-hemolytic|lcl|cTer
ARIVQRIKDFLR
>lcl|1320|lcl|HAPPENN&HAPPENN-RR90|lcl|subdataset_2|lcl|non-hemolytic
QLKVDLWGTRSGIQPEQHSSGKSDVRRWRSRY
>lcl|3292|lcl|HAPPENN|lcl|subdataset_2|lcl|hemolytic|lcl|cTer
ILGKLLKTAAKLLSNL
>lcl|3784|lcl|HAPPENN|lcl|subdataset_2|lcl|non-hemolytic|lcl|cTer
GIHDILKYGKKS
>lcl|21|lcl|HAPPENN&HAPPENN-RR90&HAPPENN-hard|lcl|subdataset_2|lcl|hemolytic
GVPCGESCVFIPCITGVIGCSCSSNVCYLN
>lcl|665|lcl|HAPPENN|lcl|subdataset_2|lcl|non-hemolytic
GSKKPVPIIYCNRRATGKCQRM
>lcl|4758|lcl|HAPPENN&HAPPENN-RR90&HAPPENN-hard|lcl|subdataset_2|lcl|hemolytic|lcl|cTer
FLGALWNVAKKVF
>lcl|3626|lcl|HAPPENN&HAPPENN-RR90&HAPPENN-hard|lcl|subdataset_2|lcl|non-hemolytic|lcl|cTer
GRFKRFRKKFKKAFKKASPVIPLLHL
>lcl|3282|lcl|HAPPENN&HAPPENN-RR90|lcl|subdataset_2|lcl|non-hemolytic|lcl|cTer
FKKALHLFKPIKKFLKWK
>lcl|3913|lcl|HAPPENN&HAPPENN-RR90&HAPPENN-hard|lcl|subdataset_2|lcl|non-hemolytic|lcl|cTer
FPVTWKWWKWWKG
>lcl|420|lcl|HAPPENN|lcl|subdataset_2|lcl|non-hemolytic|lcl|cTer
HFLLTKVNLAKKIL
>lcl|840|lcl|HAPPENN&HAPPENN-RR90|lcl|subdataset_2|lcl|non-hemolytic|lcl|cTer
KLLKWLKKLLK
>lcl|2956|lcl|HAPPENN|lcl|subdataset_2|lcl|hemolytic|lcl|cTer
GLLKAIKTLL
>lcl|2426|lcl|HAPPENN&HAPPENN-RR90&HAPPENN-hard|lcl|subdataset_2|lcl|non-hemolytic|lcl|cTer
IRPIIRPIIRPIIRPIIRPIIRPIIRPI
>lcl|2219|lcl|HAPPENN|lcl|subdataset_2|lcl|non-hemolytic|lcl|cTer
LLLIVLRKK
>lcl|2882|lcl|HAPPENN|lcl|subdataset_2|lcl|non-hemolytic
LKKKKFLLKQ
>lcl|3023|lcl|HAPPENN|lcl|subdataset_2|lcl|hemolytic|lcl|cTer
GMWSKILGKLIR
>lcl|83|lcl|HAPPENN&HAPPENN-RR90&HAPPENN-hard|lcl|subdataset_2|lcl|hemolytic|lcl|cTer
FLPLVTGLLSGLL
>lcl|1489|lcl|HAPPENN|lcl|subdataset_2|lcl|hemolytic
ICGETCVGGTCNTPGCSCSWPVCTRNGLP
>lcl|2284|lcl|HAPPENN&HAPPENN-RR90&HAPPENN-hard|lcl|subdataset_2|lcl|hemolytic
GFWSSVWDGAKNVGTAIIKNAKVCVYAVCVSHK
>lcl|1253|lcl|HAPPENN|lcl|subdataset_2|lcl|hemolytic
KWKSFLKTLKSPVKTVFYTALKPISS
>lcl|1989|lcl|HAPPENN&HAPPENN-hard|lcl|subdataset_2|lcl|non-hemolytic|lcl|cTer
GFLEKLKTGAKDFASAFVNSIK
>lcl|1374|lcl|HAPPENN&HAPPENN-RR90|lcl|subdataset_2|lcl|non-hemolytic
GVVKVSRLKGESLRARL
>lcl|1733|lcl|HAPPENN&HAPPENN-hard|lcl|subdataset_2|lcl|non-hemolytic|lcl|cTer
FWQRNIRKWR
>lcl|3312|lcl|HAPPENN|lcl|subdataset_2|lcl|hemolytic|lcl|cTer
IKLSKETKKNLKKVLKGAIKGAIAVAKMV
>lcl|1806|lcl|HAPPENN|lcl|subdataset_2|lcl|non-hemolytic|lcl|cTer
PPIVGSIPLGCG
>lcl|2710|lcl|HAPPENN&HAPPENN-RR90&HAPPENN-hard|lcl|subdataset_2|lcl|hemolytic|lcl|cTer
FFGKVLKLIRKIF
>lcl|660|lcl|HAPPENN&HAPPENN-RR90|lcl|subdataset_2|lcl|non-hemolytic
VRRFPAAAPFLRR
>lcl|2225|lcl|HAPPENN&HAPPENN-RR90|lcl|subdataset_2|lcl|non-hemolytic|lcl|cTer
IHKFWRCRRRFCRWFKHI
>lcl|1920|lcl|HAPPENN&HAPPENN-RR90|lcl|subdataset_2|lcl|non-hemolytic
SMLAYVDKNDHINPPHSPRS
>lcl|3603|lcl|HAPPENN&HAPPENN-RR90|lcl|subdataset_2|lcl|non-hemolytic
RSMRLSFRARGYGFR
```

```
>lcl|3969|lcl|HAPPENN&HAPPENN-hard|lcl|subdataset_2|lcl|non-hemolytic|lcl|cTer
GVVDILKGAAKDIAGHLASKVANKL
>lcl|2041|lcl|HAPPENN&HAPPENN-RR90&HAPPENN-hard|lcl|subdataset_2|lcl|non-hemolytic
TWLPAVIKIQAHWRGYRQRKIYL
>lcl|3622|lcl|HAPPENN|lcl|subdataset_2|lcl|hemolytic|lcl|cTer
RLWLAIWRR
>lcl|2639|lcl|HAPPENN&HAPPENN-RR90&HAPPENN-hard|lcl|subdataset_2|lcl|non-hemolytic|lcl|cTer
FLPIAGKLLSGLSGLL
>lcl|3345|lcl|HAPPENN|lcl|subdataset_2|lcl|hemolytic|lcl|cTer
FIKRIARLLRKIF
>lcl|4189|lcl|HAPPENN&HAPPENN-hard|lcl|subdataset_2|lcl|non-hemolytic
KKLFKKILKYLAGPAGIGKFLHSAK
>lcl|3264|lcl|HAPPENN|lcl|subdataset_2|lcl|non-hemolytic
KPPPWVPV
>lcl|1634|lcl|HAPPENN&HAPPENN-RR90|lcl|subdataset_2|lcl|non-hemolytic
IVRPPIRCKAAFC
>lcl|3520|lcl|HAPPENN|lcl|subdataset_2|lcl|non-hemolytic|lcl|cTer
SVDMVMKGLKIWPL
>lcl|79|lcl|HAPPENN|lcl|subdataset_2|lcl|hemolytic
GILSSFKGVAKGVAKDLAGKLLETLKCKITGC
>lcl|29|lcl|HAPPENN&HAPPENN-RR90|lcl|subdataset_2|lcl|non-hemolytic|lcl|cTer
ILPLKLPWLPLRR
>lcl|2808|lcl|HAPPENN&HAPPENN-RR90|lcl|subdataset_2|lcl|non-hemolytic|lcl|cTer
RVIEVVQGAYRAIRHIPRRIRQGLERIL
>lcl|848|lcl|HAPPENN|lcl|subdataset_2|lcl|non-hemolytic
FDWDSVLKGVEGFVRGYF
>lcl|64|lcl|HAPPENN|lcl|subdataset_2|lcl|hemolytic
FFHHIFRGIVHVGKTIHRLVTG
>lcl|4282|lcl|HAPPENN&HAPPENN-RR90&HAPPENN-hard|lcl|subdataset_2|lcl|non-hemolytic
KGIRGYKGGYKGAFKQTKY
>lcl|1522|lcl|HAPPENN|lcl|subdataset_2|lcl|hemolytic
GFMKYIKPLIPHAVKAISKLI
>lcl|3104|lcl|HAPPENN&HAPPENN-RR90&HAPPENN-hard|lcl|subdataset_2|lcl|hemolytic|lcl|cTer
GFGMALRLLRRVL
>lcl|1362|lcl|HAPPENN&HAPPENN-RR90|lcl|subdataset_2|lcl|non-hemolytic
SFPFFPPGICKRLKRC
>lcl|1503|lcl|HAPPENN&HAPPENN-RR90&HAPPENN-hard|lcl|subdataset_2|lcl|hemolytic
FLPLVLGALSGILPKILGK
>lcl|2902|lcl|HAPPENN|lcl|subdataset_2|lcl|non-hemolytic
KLKLKFLKKQ
>lcl|3872|lcl|HAPPENN&HAPPENN-hard|lcl|subdataset_2|lcl|non-hemolytic|lcl|cTer
LRIIKKILKKLI
>lcl|4211|lcl|HAPPENN&HAPPENN-RR90&HAPPENN-hard|lcl|subdataset_2|lcl|hemolytic
FLPIVASLAANFLPKIICKITKKC
>lcl|1840|lcl|HAPPENN|lcl|subdataset_2|lcl|hemolytic
EKALEKLIAIQKAIKGMLNGWFTGVGFRRKR
>lcl|916|lcl|HAPPENN|lcl|subdataset_2|lcl|non-hemolytic|lcl|cTer
QLKVDLWGTRSGIQPEQHSSGKSDVRRWRSRY
>lcl|1629|lcl|HAPPENN&HAPPENN-RR90&HAPPENN-hard|lcl|subdataset_2|lcl|non-hemolytic|lcl|cTer
FFPMLADLVSKIF
>lcl|2157|lcl|HAPPENN&HAPPENN-hard|lcl|subdataset_2|lcl|non-hemolytic|lcl|cTer
KFLKKAKKFGKAFVKIL
>lcl|3561|lcl|HAPPENN&HAPPENN-RR90|lcl|subdataset_2|lcl|non-hemolytic
PPCRGIFCRRVGSSSAIARPGKTLSTFITV
>lcl|3714|lcl|HAPPENN&HAPPENN-hard|lcl|subdataset_2|lcl|non-hemolytic|lcl|nTer|lcl|cTer
ILRWPWWPWRRK
>lcl|2277|lcl|HAPPENN&HAPPENN-RR90&HAPPENN-hard|lcl|subdataset_2|lcl|hemolytic|lcl|cTer
FLRSLLRGAKAIYRGARAGWRG
>lcl|3113|lcl|HAPPENN|lcl|subdataset_2|lcl|hemolytic|lcl|cTer
IWSAIWSGIKGLL
>lcl|4729|lcl|HAPPENN&HAPPENN-RR90|lcl|subdataset_2|lcl|non-hemolytic
KWLRRPWRRWR
>lcl|645|lcl|HAPPENN|lcl|subdataset_2|lcl|hemolytic
KNLRRIIRKIIHIIKKYG
>lcl|1805|lcl|HAPPENN|lcl|subdataset_2|lcl|non-hemolytic|lcl|cTer
GRFRRLRKKTRK
```
